# Dormancy as a Signature of Microbial Community Disassembly during Hydrological Collapse in a Desert Oasis

**DOI:** 10.1007/s00248-026-02744-z

**Published:** 2026-04-17

**Authors:** Antonio González-Sánchez, Emmanuel Cordero-Martínez, María Dolores Rodríguez-Torres, África Islas-Robles, Louis Paul Decena-Segarra, Norberto García-Miranda, Marisol Navarro-Miranda, Román Zapién-Campos, Felipe García-Oliva, Luis E. Eguiarte, Valeria Souza, Gabriela Olmedo-Álvarez

**Affiliations:** 1 Department of Genetic Engineering, Cinvestav Unidad Irapuato, Km 9.6 Carretera Irapuato-León, 36824 Guanajuato, Mexico; 2 Instituto Politécnico Nacional, CIIDIR Unidad Michoacán, Justo Sierra Ote. 28, 59510 Jiquilpan de Juárez, Mexico; 3 Instituto de Investigaciones en Ecosistemas y Sustentabilidad, Universidad Nacional Autónoma de México, Antigua Carretera a Pátzcuaro 8701, 58190 Morelia, México; 4 Department of Evolutionary Ecology, Instituto de Ecología, Universidad Nacional Autónoma de México. CU, Coyoacán CdMx 04510 Ciudad de México, Mexico; 5 Department of Genetics, Evolution and Environment, University College London, London, UK

**Keywords:** Wetland Desiccation, Sporulation, Microbial Dormancy, Hydrological Decline, Microbial Biodiversity, Spore-Forming Bacteria, Cuatro Cienegas Basin, Arid Ecosystems, Agricultural Water Extraction, Groundwater Depletion

## Abstract

The Cuatro Ciénegas Basin (CCB) in Coahuila, Mexico, a Ramsar Wetland, is characterized by a unique network of pozas renowned for microbial diversity. Among them, the Churince hydrological system epitomized a delicate balance of endemic species, stable water regimes, and complex microbial assemblages. Over the last five decades, however, the Churince underwent catastrophic ~ 99% water loss, driven by intensive agricultural extraction. This desiccation caused hydrological collapse and reshaped microbial physiology. Longitudinal records from 2007 to 2025, combining satellite imagery, piezometer data, and field observations, documented progressive declines beginning in 2011 and culminating in complete desiccation by 2019. To track microbial responses, we employed a cultivation-based approach distinguishing vegetative cells from dormant spores, quantified as heat-resistant CFU, enabling direct physiological assessment of metabolic activity versus dormancy. As water diminished, spore abundance increased relative to vegetative forms, revealing a shift toward dormancy as a survival strategy under extreme desiccation. Complementary mesocosm experiments showed that reduced diversity further enriched for spores, illustrating how loss of community complexity accelerated dormancy and erosion of active assemblages. Together, these results demonstrated that habitat loss triggered microbial community change, which we frame as a microbial extension of the ecological concept of community disassembly. By combining two decades of hydrological evidence with microbial physiology, this study fills a critical gap in understanding how wetland drying selects for microbial dormancy. These findings underscore the urgent need for sustainable agriculture and integrated water management to preserve biodiversity and ecosystem function in threatened wetlands worldwide.

**Supplementary Information:**

The online version contains supplementary material available at 10.1007/s00248-026-02744-z.

## Introduction

Freshwater scarcity is an escalating global threat, reshaping ecosystems across continents. Microbial communities, as the primary drivers of biogeochemical cycles, are essential to ecosystem stability, nutrient recycling, and planetary resilience. These invisible architects not only sustain critical environmental processes but also represent the evolutionary foundation of biochemical diversity. From large inland lakes to fragile wetlands, the disappearance of water endangers biodiversity, local livelihoods, and ecosystem function.

The Cuatro Ciénegas Basin (CCB), in the Chihuahuan Desert of Coahuila, Mexico, exemplifies this crisis. A globally significant oasis, the CCB is known for extraordinary microbial biodiversity, with mildly saline waters and extreme oligotrophic conditions. Its microbial communities, shaped over geological timescales, provide a unique window into early Earth analogs and microbial adaptation to nutrient limitations. CCB is historically characterized by a unique network of ponds and small lagoons locally called “pozas”, renowned for microbial diversity. Among the aquatic systems in the basin, the Churince hydrological system stood out for its fragile balance and richness in endemic species [1]. Recognizing its significance, it was designated both as a “Protected Area for Flora and Fauna” by the Mexican federal government and a Ramsar Wetland of international importance.

The Churince system, situated at a higher elevation than the rest of the basin and bounded by the Sierra de San Marcos y Pinos, historically maintained a stable hydrological regime influenced by ancient groundwater with magmatic signatures. This environment supported gradients in salinity, oxygen, and pH, fostering a mosaic of microhabitats. Previous studies have identified ancient and deeply branching microbial lineages, including the endemic *Bacillus coahuilensis*, which exemplify adaptations to oligotrophic conditions [2]. Members of the Bacillaceae family, many of which are capable of sporulation, were observed to be abundant in the Churince system [3, 4], serving as models for microbial survival under nutrient stress.

Over the last five decades, however, the Churince system has undergone severe hydrological collapse, losing more than 99% of its surface water. This desiccation has been driven by unchecked agricultural extraction, especially for the irrigation of alfalfa (*Medicago sativa*), a highly water-demanding forage crop. In the region, alfalfa is cultivated through two main irrigation practices: flooding of fields using spring water or by center-pivot irrigation systems supplied by wells. Together, these methods withdraw far more water than is replenished annually by precipitation, creating a chronic imbalance between extraction and natural recharge. This imbalance has directly contributed to aquifer depletion and ecological collapse.

Microbial communities are highly responsive to environmental change and can serve as indicators of ecosystem health. Water availability is central to this responsiveness: without extracellular water, nutrients cannot dissolve for cellular uptake, and microbial cells cannot move toward resources, ultimately leading to starvation [5, 6]. In drylands, microorganisms often endure prolonged periods of desiccation, facing severe energy limitations and stress. Many respond by entering dormancy, a reversible state of reduced metabolic activity that allows them to survive until favorable conditions return. Dormant microorganisms act as microbial “seed banks,” influencing ecological stability and evolutionary dynamics [7]. Dormancy has been defined as “a temporary adaptive state of reduced metabolic activity within an extended period of arrested growth that can enable a microbe to maintain viability under unfavorable conditions” [8].

Dormancy strategies vary. Some taxa form resting structures with thickened cell walls or extracellular polymers, while others produce highly stress-resistant spores. This latter strategy is well documented in Actinobacteria and Bacillota (formerly Firmicutes). Culture-independent methods cannot distinguish dormant from active fractions and systematically underestimate endospore-forming Bacillota because their resistant cell envelopes and spore coats are difficult to lyse with standard DNA extraction protocols [9].

In this study, we operationally define a shift toward dormancy as a quantifiable increase in the proportion of heat-resistant colony-forming units (CFUs) relative to the total culturable community. Within the Bacillaceae family, and particularly in the model bacterium *Bacillus subtilis*, cells can enter a state of non-replicative viability, often referred to as quiescence. The processes of sporulation and the return to vegetative growth during germination have been extensively studied [10, 11]. Bacillota phyla are widely distributed in drylands and become ecologically dominant under conditions of desiccation [12]. Spore-forming bacteria are also widespread in extreme ecosystems, including deserts [13] and endangered salt lakes [14].

Ecological communities are shaped by the dual processes of community assembly, the sequential addition of species over time, and community disassembly, the nonrandom process of progressive species declines and losses [15, 16]. Community disassembly is not simply community assembly in reverse, since disassembly is frequently driven by human-caused stressors that were absent when the community originally formed [15, 17]. This process of species loss follows predictable patterns, or “disassembly rules,” where the sequence of decline is governed by species-specific “response traits” that confer vulnerability to stressors such as habitat fragmentation, climate change, or resource depletion. The specific order in which species are lost is critically important, as it can dramatically alter ecosystem functioning, including productivity, nutrient cycling, and the provisioning of ecosystem services [14, 18]. While community disassembly is frequently studied in the context of macroorganism responses to human impacts, it also occurs as a natural, cyclical process in ephemeral ecosystems that predictably dry up or decay [16, 19].

The sequential disappearance of fish, turtles, and hydrophilic plants in the Intermediate Lagoon of Churince reflects the ecological concept of community disassembly [16]. Our results extend this framework to microbes: natural habitat loss drove disassembly toward dormancy, and the mesocosms provided a simplified experimental view of the same outcome.

This study arose from a rare, unplanned opportunity to observe microbial responses to real-world environmental collapse. As the Churince system desiccated, we integrated ecological, hydrological, and microbiological records across 19 years (2007–2025) to test how communities shift under sustained drying. Using heat sensitivity to distinguish metabolically active (vegetative) from dormant (spore-forming) cells, quantified as heat-resistant CFU, in the culturable aerobic fraction, we tracked a progressive rise in dormancy that paralleled declining water levels documented by piezometer data, satellite imagery, and field photography (including fish and turtle mortality). Critically, we advance the concept of dormancy as a measurable indicator of ecosystem stress, demonstrating that spore prevalence provides a tractable, trait-based signal of community disruption during drying.

To probe mechanism, a two-year mesocosm assay applied an initial heat pulse to remove phototrophs and heat-sensitive heterotrophs; under otherwise shared conditions, these simplified communities rapidly converged on spore enrichment, quantified as heat-resistant CFU/total CFU. Together, the long-term field trajectory and the guild-loss mesocosm support a trait-mediated pathway of community disassembly toward dormancy under a press disturbance. Here we (i) quantify multi-year changes in spore prevalence across shoreline sites, (ii) relate activity to moisture along depth and vegetation gradients and to taxonomic shifts in cultured isolates, and (iii) show that targeted guild loss alone can reproduce the dormancy shift.

## Materials and Methods

### Study Sites, Sampling and Microbial Isolation

The summary of the sampled events for this study is recorded in Supplementary Table 1. The Churince hydrological system, located at 730 m above sea level, comprises a Spring (S), an Intermediate Lagoon (IL), and a Desiccation Lagoon (DL), interconnected by a small river. In the IL, longitudinal sampling was conducted at eight shoreline sites (IL1–IL8, 30–50 m apart) in 2011, 2012, 2019, 2023, and 2025 (Supplementary Table 2). In 2012, a transect across the IL included sediments collected every 50 m along 500 m eastward (grassland) and 550 m westward (*sotol* dominated (*Dasylirion))* from the shore, yielding 40 samples (Supplementary Table 3). In 2014, a 60 cm sediment core was taken at IL7 and sectioned into 2 cm intervals, with 10 subsamples analyzed (Supplementary Tables 4 and 5). GPS coordinates for all sites are provided in Supplementary Tables 2 and 3. All cultivation experiments were conducted using a low-salinity derivative of Marine Agar 2216 (‘Modified Marine Medium’, MMM) containing ~ 11.8 g L⁻¹ mixed salts (~ 1.18% w/v), of which NaCl is 5 g L⁻¹ (0.5%) substantially below seawater salinity. Modified Marine Medium (MMM), composition per liter: NaCl 5 g; MgSO₄ 4.8 g; Na₂SO₄ 1 g; CaCl₂ 0.4 g; KCl 0.2 g (each from separate stock); Na₂CO₃ 0.1 g; ferric citrate 0.1 g; KBr 0.08 g; Na₂HPO₄ 0.08 g; NaF 0.024 g; H₃BO₃ 0.022 g; NH₄NO₃ 0.016 g (combined trace-mineral stock); peptone 5 g; yeast extract 1 g. For solid medium: agar 15–16 g L⁻¹. Our formulation of Marine medium has been standard in our own long-term sampling work [3, 4] enabling consistent recovery of heat-resistant/spore-forming lineages (heat-resistant CFU) and comparability across years.

For longitudinal and transect samples, surface sediments (top ~ 1 cm) were collected with sterile 50 mL tubes. Approximately 0.1 g of sediment was serially diluted (up to 10⁻⁵) in PBS and plated on Modified Marine Medium agar (MMM). Core subsamples (0.5 g mL⁻¹) were resuspended in Tris-HCl buffer (pH 8.4), agitated (600 rpm for 1 h), diluted, and plated similarly. All samples were collected with federal permission (permit SGPA/DGVS/04512) and transported (at 4 °C) either to the CBTA22 laboratory in Cuatro Ciénegas or to the CINVESTAV Irapuato laboratory for plating.

### Mesocosms: Testing Dormancy After Loss of Phototrophs and Heat-Sensitive Heterotrophs

In 2014, while surface water remained, sediments from three Intermediate Lagoon sites (IL4, IL5, IL8) were used to establish laboratory mesocosms. For each site, homogenized sediment was split into two treatments: unheated (intact community) and heat-treated (80 °C, 30 min) to remove phototrophs and heat-sensitive aerobic heterotrophs, enriching for heat-resistant spore formers. Triplicate mesocosms per site and treatment were maintained in glass flasks at 20 °C under a 12 h:12 h light–dark cycle; water was added only to keep sediments covered. At scheduled intervals over two years, subsamples were plated on Modified Marine Medium (MM) to quantify total CFU and the heat-resistant fraction. This design tests whether selective guild loss increases dormancy; it does not test drought, as water was maintained throughout. The 80 °C pretreatment selectively eliminates phototrophs (cutting primary production) and heat-sensitive heterotrophs (reducing consumer diversity and biotic interactions), while leaving heat-resistant spore formers.

### Colony Count and Sporulation Percentage

In most sampling schemes, CFU counts were obtained under two conditions: (1) Direct plating, which recovers colonies arising from both vegetative cells and spores that germinate upon plating (total CFU); and (2) Heat-treated samples, in which sediments were heated at 80 °C for 30 min to inactivate vegetative cells and selectively recover thermotolerant bacteria or spores that readily germinate and grow into colonies (heat-resistant CFU). Heat treatment (80 °C, 30 min) follows standardized protocols for selective recovery of endospore formers [20], consistent with previous studies in this system [3, 4]. The plating workflow and cell cycle are illustrated in Supplementary Fig. 1. Sporulation percentage was determined by: (CFU from spores/Total CFU) × 100. Plates were incubated at 28 °C for 5 days. Heat-treated CFU can occasionally exceed direct CFU, yielding ratios above 100%. The explanation is that (i) heat treatment can activate spore germination and (ii) remove antagonistic vegetative cells allowing growth of previously antagonized cells.

### Satellite Imagery and Water Level Measurements in the Churince Hydrological System

Satellite imagery was obtained from Google Earth (7.3.6.10201) using the time-lapse feature to access historical images. Water levels in the Churince Spring were monitored from 2007 to 2015 using a piezometer installed in a borehole by the Comisión Nacional de Áreas Naturales Protegidas (CONANP). Water level was recorded as the vertical distance between the piezometric sensor at the borehole bottom and the water surface above the sensor. Measurements were taken in different months each year (Supplementary Table 6). In 2012, a temporary disconnection in monitoring occurred. The lowest recorded water level was observed in 2015, marking the end of the measurement period.

### DNA Extraction from Cultivated Strains

A total of 1,419 bacterial isolates were obtained from samples of 2007, 2011, 2012, 2014, 2019, and 2022. Total DNA was extracted for PCR amplification of the 16S rRNA gene using a modified phenol–chloroform method with bead beating (0.1 mm glass beads) to lyse cells. Briefly, overnight cultures were centrifuged (16,000 × g, 5 min), washed with TE buffer, and resuspended in lysis solution (2% Triton X-100, 1% SDS, 100 mM NaCl, 10 mM Tris–HCl pH 8.0, 1 mM EDTA). After the addition of TE buffer and phenol–chloroform, 0.1 mm glass beads were incorporated, and samples were vortexed for 5 min to enhance cell disruption. The aqueous phase obtained after centrifugation was re-extracted with chloroform, and DNA was recovered by ethanol precipitation, washed with 75% ethanol, air-dried, and resuspended in nuclease-free water containing RNase (2 µg/mL).

### Taxonomic Classification and Phylogenetic Analysis

Taxonomic classification was based on 16S rDNA sequences amplified using universal primers 27F (5′-AGAGTTTGATCCTGGCTCAG-3′) and 1492R (5′-TTACGGYTACCTTGTTACGACT-3′). PCR products were sequenced by Sanger technology at LANGEBIO-CINVESTAV, Irapuato, Mexico. A total of 1,419 full-length 16S rDNA sequences were obtained and deposited in the NCBI database; accession numbers are listed in Supplementary Table 7. Taxonomic assignment of each isolate was performed using BLASTn against the NCBI 16S ribosomal RNA database, complemented with SILVA classification for confirmation. For phylogenetic analysis, the 1,419 sequences were trimmed to ~ 350 bp and aligned with the ClustalW algorithm. Phylogenetic reconstruction was conducted in RAxML-GUI using the Maximum Likelihood (ML) method with the K2 + G evolutionary model and 1,000 bootstrap replicates. Fusobacterium was used as the outgroup. The resulting tree was visualized and annotated in the Interactive Tree Of Life (iTOL) platform.

### Statistical Analyses of CFU, Sporulation, and Community Structure

Differences in CFU counts and sporulation percentages among the longitudinal time series, spatial transect, and vertical depth profile datasets were evaluated using one-way analysis of variance (ANOVA), with a significance threshold of α = 0.05. Microbial community analyses were conducted for the classified 1,419 bacterial isolates. For longitudinal comparisons, isolates were grouped into three ecological phases representing distinct hydrological states of the Churince System: With Water (2007), Transition (2011, 2012, 2014), and Desiccation (2019, 2022). Community composition differences were assessed via Permutational Multivariate Analysis of Variance (PERMANOVA, α = 0.05) applied to both alpha and beta diversity indices. Dissimilarity matrices were computed using Bray–Curtis (abundance-based) and Jaccard (presence/absence-based) distances. Beta diversity patterns were visualized using Principal Coordinates Analysis (PCoA). All community-level statistical analyses were performed in R using the vegan package, with visualizations generated in ggplot2.

For genus-level analyses, taxonomic assignments were aggregated at the genus level, and both relative and absolute abundances were calculated as the number of isolates per sampling site within each ecological phase (Supplementary Table 8). Isolates belonging to *Bacillus* were examined separately (Supplementary Table 9). Differences among phases were tested with the Kruskal–Wallis test (α = 0.05), statistic (H), and its associated p-value indicated whether at least one group differed significantly in distribution (Supplementary Table 10). This analysis was performed in Python 3.11 using the kruskal function from scipy.stats module.

## Results

### Hydrological Collapse of the Churince Intermediate Lagoon and Agricultural Drivers

**Fig. 1:**
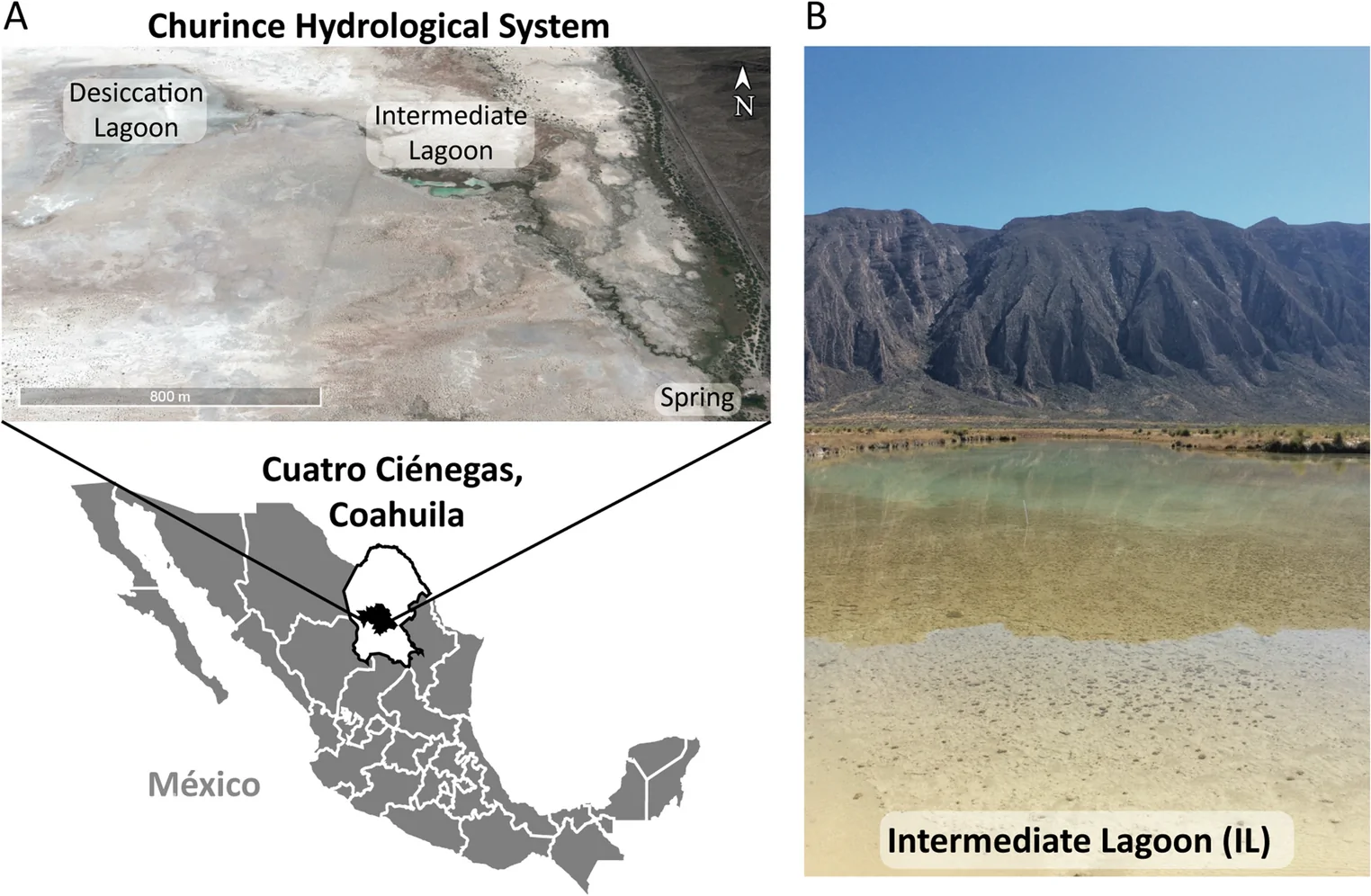
The Churince System in Cuatro Ciénegas, Coahuila, México

The Cuatro Ciénegas Basin (CCB) exemplifies the fragility of desert wetlands. Within it, the Churince hydrological system—comprising the Desiccation Lagoon (DL), the Intermediate Lagoon (IL), and a Spring (WS)—was once an oasis of exceptional biodiversity [1], home to endemic fauna such as *Eleutherodactylus* sp. nov., *Terrapene coahuila*, and *Cyprinella xanthicara* (Supplementary Fig. 2). Photographs from 2001 and satellite imagery from 2009 confirm that the Intermediate Lagoon still retained significant surface water at the beginning of our study (Fig. 1).

(**A**) The Churince system consists of the Desiccation Lagoon (DL), Intermediate Lagoon (IL), and Spring (S), connected by an arroyo. The satellite image was taken from Google Earth from 2009, when the lagoons still held water. (**B**) Photograph of the Intermediate Lagoon in 2001, when the system still retained surface water.

**Fig. 2:**
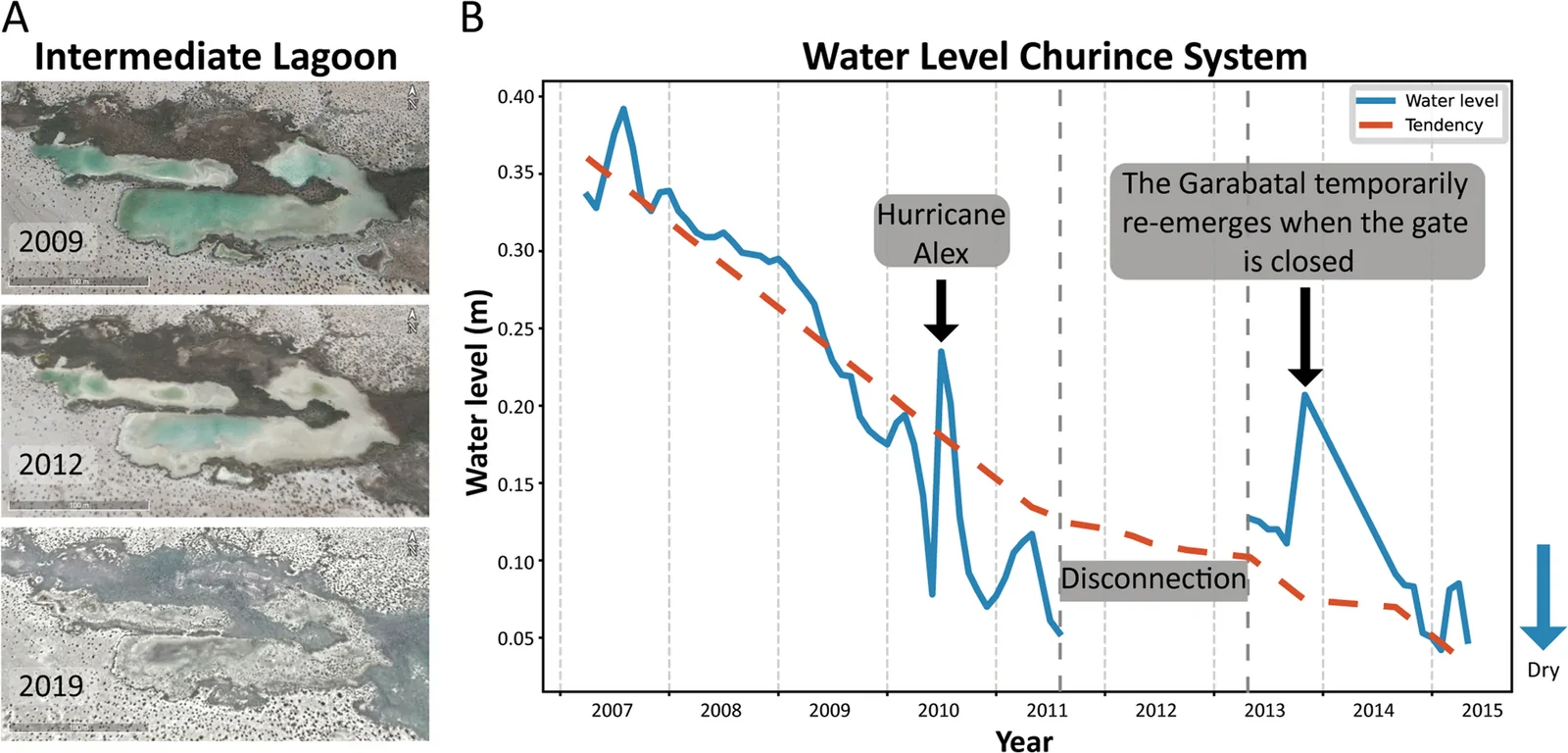
Gradual Desiccation of the Intermediate Lagoon in the Churince system

Multiple lines of evidence converged on a rapid collapse. Satellite images revealed the water loss: abundant water in 2009, drastically reduced by 2012, and gone by 2019 (Fig. 2A). The piezometer records (2007–2015) showed a steady groundwater decline (Fig. 2B), with a sharp drop in 2011 coinciding with observations of dead turtles and fish carpeting exposed sediment (Supplementary Fig. 3). Photographs from 2014 to 2019 further documented receding shorelines and wildlife loss. By 2019, the Intermediate Lagoon had dried completely.

(**A**) Satellite images (Google Earth) showing progressive water loss and increasing aridity in the Intermediate Lagoon and surrounding area: 2009 (with water), 2012 (reduced water), and 2019 (completely dry). (**B**) Yearly average groundwater levels (2007–2015) measured by a piezometer installed at the Churince well. The piezometer records the height of the groundwater table relative to the surface, reflecting the water available to feed the lagoon. A transient rise in 2010 corresponds to Hurricane Alex and the brief re-emergence of the Garabatal spring after the temporary closure of the gate. Piezometer data are provided in Supplementary Table 6.

This collapse coincided with intensified agriculture (Supplementary Fig. 4). Regional water extraction rose from 22 million m³ in 1997 to 88 million m³ in 2022 (public statistics: IMTA, 2023; compiled from CONANP monitoring), driven largely by alfalfa (*Medicago sativa*), a water-demanding forage crop. In the nearby Hundido Valley, alfalfa is cultivated through flood irrigation and center pivots, consuming ~ 15,660 m³/ha. Satellite images show both the reduction of surface water in Laguna Intermedia and expansion of alfalfa fields, from 21 active plots in 2007 to 29 in 2020 (Supplementary Fig. 4B). Poza La Becerra, which likely feeds the Churince aquifer, is continuously pumped for agricultural use; this extraction, combined with numerous wells in the surrounding area supplying alfalfa irrigation, is depleting the regional water table and accelerating the collapse of the Churince system. Despite its Ramsar designation, agricultural expansion strongly overlapped with and likely drove the hydrological collapse of the Intermediate Lagoon.

### Shifts in Dormant vs. Active Bacterial Fractions

Following the disassembly of the macrofaunal community (Supplementary Fig. 2), we analyzed microbial responses to desiccation. Sediments cultured on Modified Marine Medium consistently recovered Bacillaceae. Heat-treated samples revealed the spore-forming fraction, while untreated samples reflected the total culturable pool (vegetative cells plus germinable spores, detailed in Supplementary Fig. 1). The proportion of heat-resistant CFUs served as a proxy for dormancy. To capture ecological patterns, we used three strategies: a vegetation transect, a vertical sediment core, and a shoreline series spanning more than a decade of decline.

**Fig. 3:**
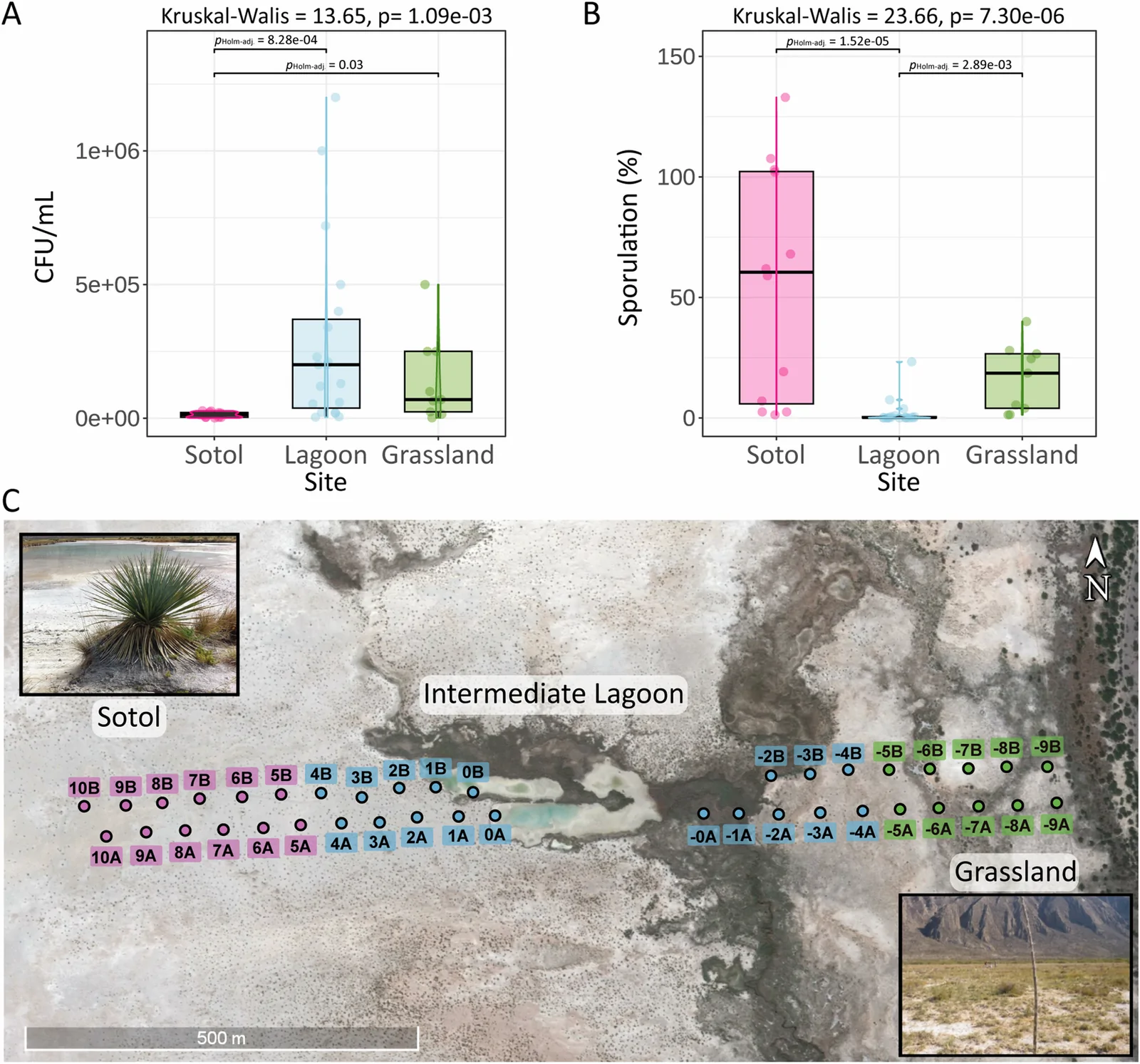
Transect sampling of the Churince System (2012). Transect across the Churince hydrological system (2012). (**A**) Mean colony-forming unit (CFU) per site; highest in grassland. (**B**) Sporulation along the east–west transect: highest at sotol, lowest at lagoon, intermediate at grassland. Statistical differences assessed using Dunn tests with Holm correction (*p* < 0.05). (**C**) Google Earth image (2012) showing 50-m sampling intervals; pink, sotol; blue, lagoon; green, grassland. Each site had a replicate 20 m away (**A**, **B**). Photos of sotol (*Dasylirion*, family Asparagaceae) (left) and grassland (right) provide habitat context. Notice that since (i) heat treatment can activate spore germination and (ii) remove antagonistic vegetative cells, heat-treated CFU can occasionally exceed direct CFU, yielding ratios above 100%

In the transect (2012, Fig. 3C), sporulation co-varied with vegetation and aridity: on the sotol-dominated west side nearly all colonies (~ 100%) originated from spores, whereas the grass-covered east side showed ~ 25% (Fig. 3B). Total CFU counts were ten times higher in grassland soils (~ 25,000 CFU/mL), indicating a larger active fraction in grassland, while spores dominated in arid zones (Fig. 3A; Supplementary Table 3).

**Fig. 4:**
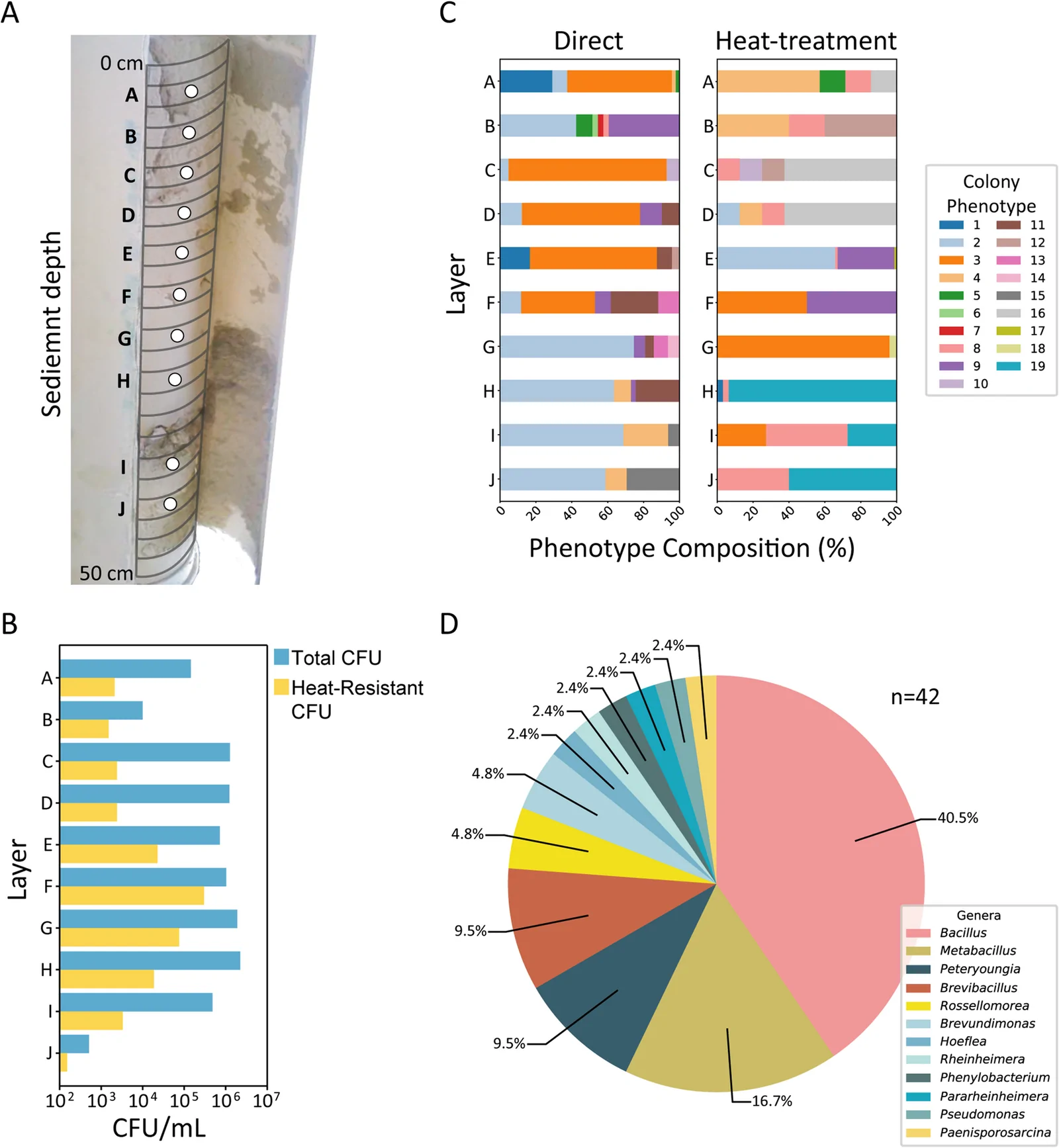
Vertical core sampling at point IL7 in the Intermediate Lagoon

The 2014 sediment core (Fig. 4A) represents decades of deposition. Despite burial, vegetative cells were consistently detected across all layers, where the total CFU always exceeded spore CFU (Fig. 4B). Higher counts were generally observed in the deeper, moister sections (Fig. 4B; Supplementary Table 5). Although the core appeared homogeneous, taxonomic composition and colony morphotypes differed among layers, indicating persistent microbial activity and structuring at depth (Fig. 4C). The relative abundance of the sequenced isolates (*n* = 42) shows that nearly 40% belong to the *Bacillus* genus (Fig. 4D and Supplementary Table 7).

Vertical core sampling at point IL7 in the Intermediate Lagoon. (**A**) Photograph of a PVC core (cut lengthwise) collected at IL7, sectioned into 2 cm segments, with ten sampling points (**A**–**J**) marked by white circles. (**B**) Phenotypic diversity of isolates from each layer: left panels show direct plating (without heat treatment), right panels show phenotypes after heat treatment. We arbitrarily defined colors for 19 morphotypes observed. (**C**) Colony-forming unit (CFU) counts per layer: blue bars, direct plating; yellow bars, heat-treated counts. (**D**) Taxonomic diversity of 41 isolates based on 16 S rDNA sequencing. The pie chart shows genus-level relative abundances, with Bacillus as the predominant genus.

**Fig. 5:**
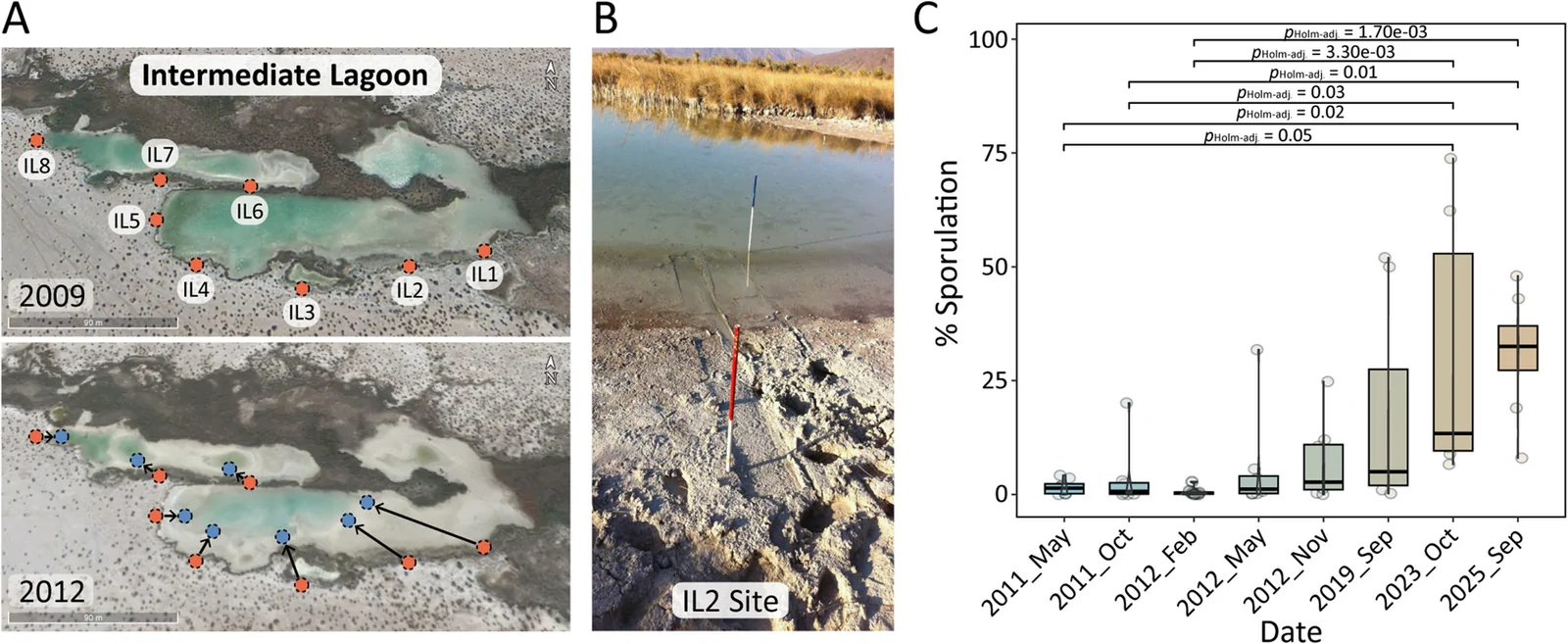
Longitudinal analysis of microbial activity during water loss in the Churince Intermediate Lagoon (2011–2025)

The shoreline series (2011–2025, Fig. 5A) documented a progressive shift toward dormancy. In 2011 and 2012, fewer than 5% of colonies originated from spores. By 2019, sporulation increased to 10–20%. In 2023, it exceeded 20% at some sites, and by 2025, it reached an average above 30% (Fig. 5C; Supplementary Table 2). This trajectory represents a trait-mediated community disassembly, where Bacillaceae shifted from vegetative states into dormancy as surface water disappeared.

(**A**) Google Earth images from 2009 to 2012 showing sampling points (IL1–IL8) established to collect sediments annually along the shoreline while it was submerged. As the water receded, new sampling poles were installed progressively toward the center of the lagoon to continue sampling submerged sediments. (**B**) Photograph of the Intermediate Lagoon 2011 showing a red sampling tube located in now-dry sand near the original shoreline and a blue tube placed farther into the lagoon, indicating pole relocation as water levels declined. (**C**) Sporulation percentages in sediment samples over time (2011, 2012, 2019, 2023 and 2025), showing a steady increase as the lagoon dried. Values represent the average across all sampling points for each date.

### Composition of Culturable Bacteria

**Fig. 6:**
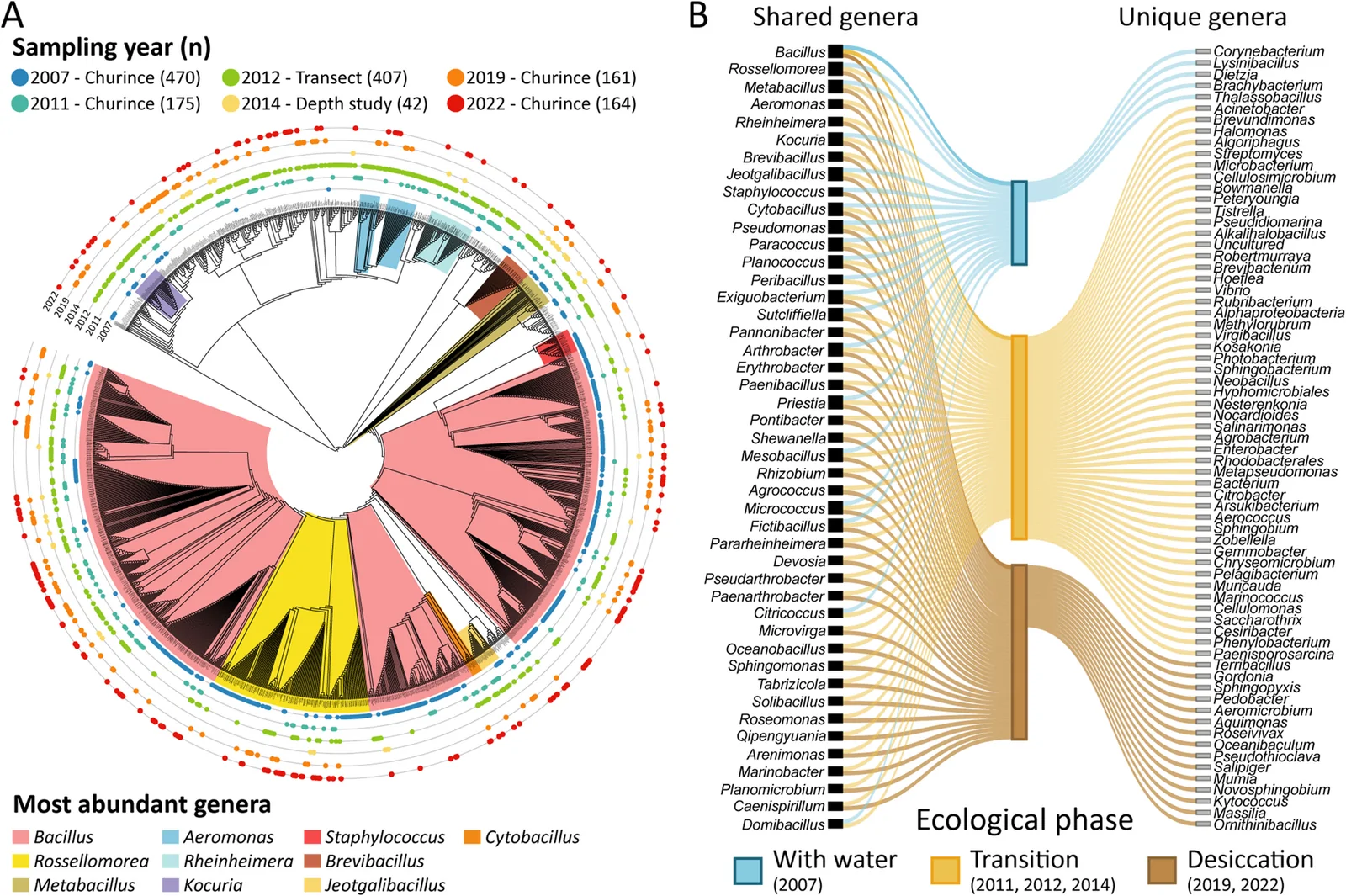
Phylogenetic diversity, taxonomic composition, and ecological phase patterns of bacterial isolates from the Churince System

Drying of the Churince Intermediate Lagoon was reflected in a decline in percent vegetative cells with a concomitant rise in sporulation percentage, alongside shifts in community composition. Across all sampling years, we sequenced and classified 1,419 bacterial isolates from the Intermediate Lagoon (Fig. 6A), of which 682 belonged to the genus *Bacillus* (Supplementary Tables 8 and 9). At the genus level, isolates were grouped into three ecological phases: With Water, Transition, and Desiccation (Fig. 6B), corresponding to different years (see Methods).

(A) Maximum Likelihood phylogenetic tree based on partial 16S rRNA gene sequences (~ 350 bp) from 1,419 isolates, color-coded by taxonomic affiliation and sampling year. Numbers of isolates per year are shown in parentheses. (B) Sankey diagram showing the distribution of genera across three ecological phases corresponding to distinct hydrological states: With Water, Transition, and Desiccation. Grey boxes denote genera unique to a single phase; black boxes denote genera present in at least two phases. The complete distribution of *Bacillus* species and statistical analysis is provided in Supplementary Tables 9 and 10.

From these ecological phases, beta diversity was assessed using PCoA based on Bray–Curtis and Jaccard distances, which showed marginal, non-significant separation among phases (Bray–Curtis: R² = 0.47, *p* = 0.267; Jaccard: R² = 0.42, *p* = 0.267) (Supplementary Fig. 5). Nevertheless, genus-level composition shifted across phases (Fig. 6B).

Alpha diversity revealed an unexpected pattern: the water phase had the lowest diversity, likely due to the 2007 focus on heat-resistant bacteria, which underestimated the number of vegetative cells. The transition phase exhibited the highest Shannon and Simpson values, indicating greater richness and evenness of the species composition. The Desiccation phase maintained high richness, probably due to rare dormant lineages, but with reduced evenness (Supplementary Fig. 6). These results suggest that transitional conditions supported more complex communities than either extreme.

Despite these shifts, a core group of taxa persisted across all phases, including several spore-forming Bacillaceae (*Bacillus*,* Rossellomorea*,* Metabacillus*,* Priestia*,* Sutcliffiella*,* Mesobacillus*,* Cytobacillus*, and *Jeotgalibacillus*) as well as *Micrococcus*,* Arthrobacter*,* Kocuria*,* Pseudomonas*,* Planococcus*, and *Paracoccus* (Fig. 6B and Supplementary Fig. 7). Within this core, *Bacillus* represented 67.45%, 32.37%, and 50.15% of isolates in the water, transition, and desiccation phases, respectively (Supplementary Table 8). The elevated proportion observed in the water phase likely reflects a methodological bias, as isolations at that time relied on heat-treated samples, favoring spore-forming and thermotolerant taxa. Nonetheless, *Bacillus* abundance increased again from the transition to desiccation phases, reaching ~ 50% of isolates.

Species-level analysis showed that *B. licheniformis* rose from ~ 6% in 2007 to ~ 21% in 2022, while species such as *B. aequororis*,* B. paramycoides*, and *B. mesophilum* declined with drying (Supplementary Tables 9 and 10). Thus, surface water loss shifted the culturable community toward spore-forming *Bacillus* species tolerant of desiccation.

### Reduced Community Diversity Drives Microbial Assemblages Toward Dormancy

Field data showed habitat loss, diversity loss, and desiccation occurring simultaneously. To isolate the specific effect of community simplification, we established a two-year mesocosm experiment using lagoon sediments, which were maintained under a light–dark cycle and watered only to offset evaporation. Treatments compared intact communities with those exposed to an initial heat treatment that eliminated non-spore-forming heterotrophs and primary producers (cyanobacteria and algae).

**Fig. 7:**
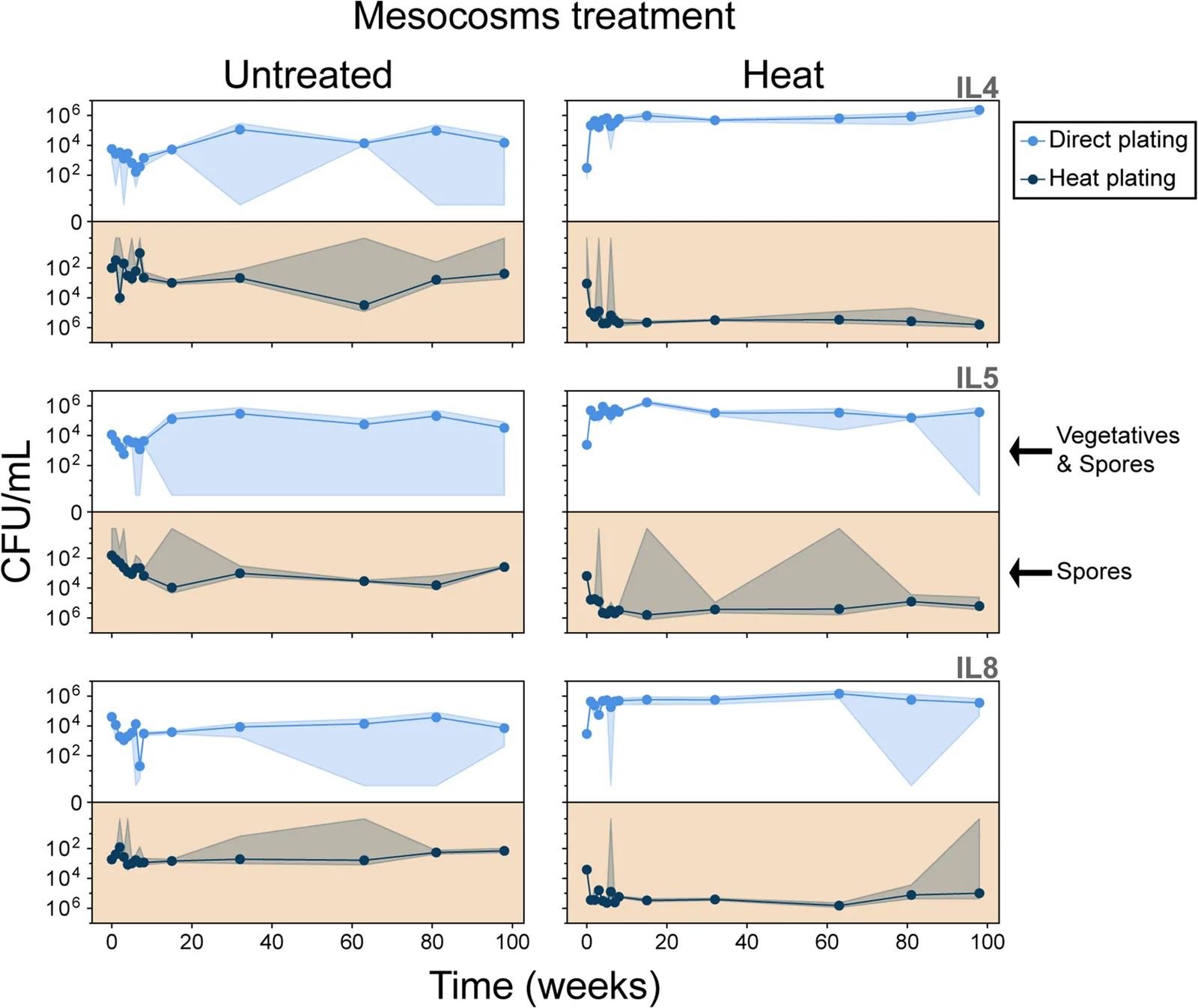
Population dynamics of bacterial phenotypes under direct and heat-treated mesocosm conditions

Our results showed a sharp divergence between treatments. Untreated mesocosms, which retained a complete microbial food web, maintained predominantly active communities, with spores representing only 11–36% of culturable bacteria across the three sites (IL4, IL5, and IL8) (Fig. 7; Supplementary Fig. 8). In contrast, heat-treated mesocosms, reduced to heterotrophs, rapidly shifted toward dormancy: spores accounted for 52–72% of CFU. These differences between treatments were statistically significant (Wilcoxon test, *P* ≤ 0.05; Supplementary Fig. 9). Heat treatment caused substantial mortality, releasing necromass that fueled a short-lived bloom of endospore-forming Bacillaceae, followed by collapse into spore dominance. Colony morphotypes confirmed that spore-former diversity was retained even as autotrophs and competitors were lost. Vegetative and spore counts showed mirror trajectories, with about half of the surviving bacteria sporulating soon after the perturbation (Supplementary Tables 11 and 12).

Mirror plots show temporal changes in colony-forming units per milliliter (CFU/mL). Upper panels display direct plating (vegetative cells + spores), and lower panels (orange background) show heat-treated samples (80 °C, 30 min; spores only). Each line with shaded area denotes a distinct bacterial phenotype, consistently color-coded across treatments. Columns correspond to site samples IL4, IL5, and IL8 (see Fig. 5A), each with three independent replicates. Panel (A) shows mesocosms initiated without prior heat treatment; panel (B) shows mesocosms initiated with heat treatment.

Trait-mediated microbial community disassembly toward dormancy.

**Fig. 8:**
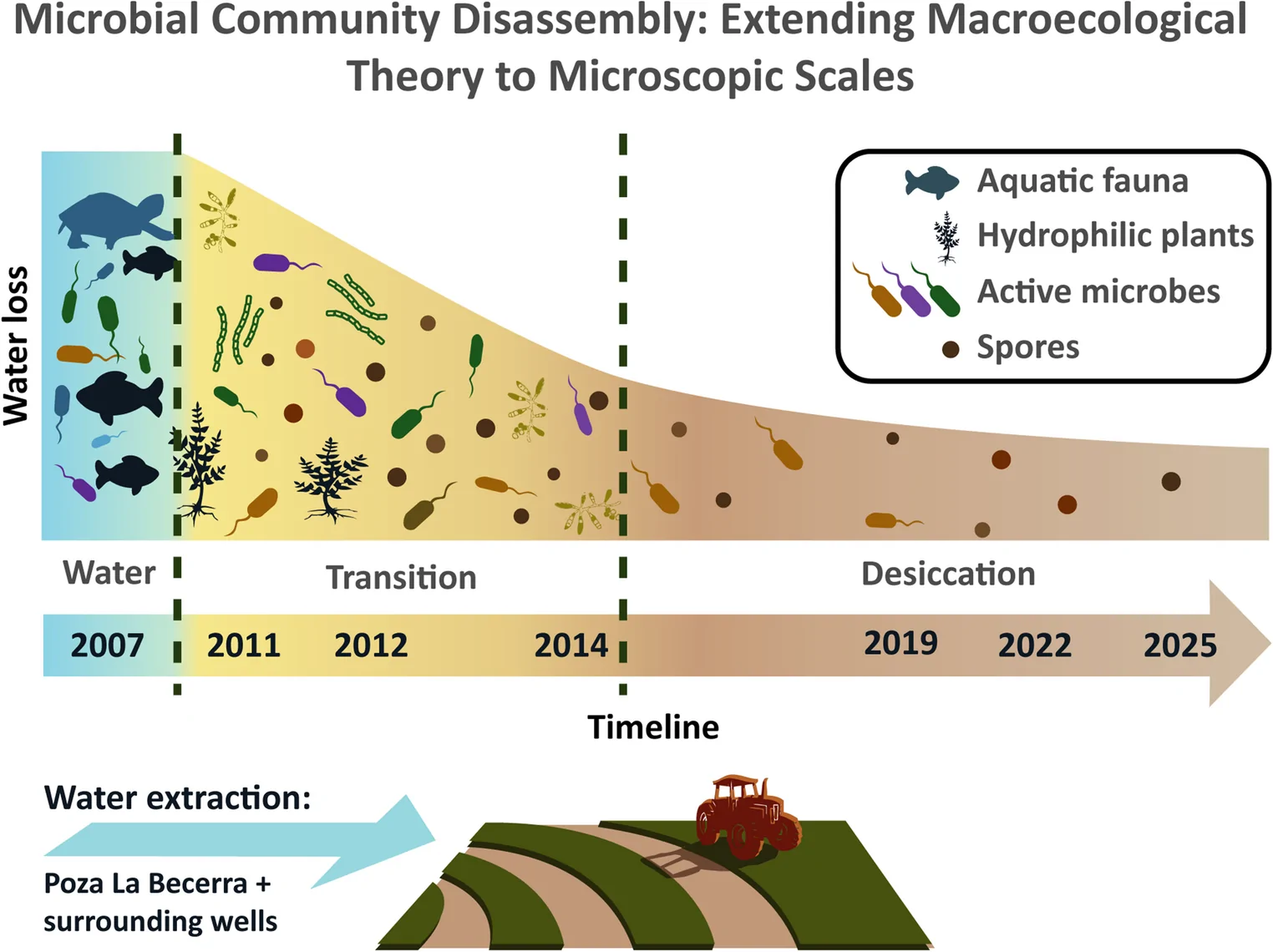
Conceptual model of microbial community disassembly toward dormancy during hydrological collapse. A left axis indicates progressive water loss. The triangle is divided into three phases: blue “with water” block showing turtles, fish and mainly vegetative bacteria; a yellow “transition” block with macrofauna absent and increasing spores alongside motile vegetative cells; and a final narrow block at “desiccation” with few vegetative cells (active fraction) and predominantly spores in multiple colors to indicate diverse spore-forming taxa. Along the base, a timeline indicates 2007 (water), 2012 and 2014 (transition), and 2019, 2022, 2025 (desiccation). The inset illustrates alfalfa irrigation and multiple wells drawing groundwater, noting particular influence from neighboring Poza La Becerra from which water is continuously drawn. The figure summarizes our observation that spore prevalence rises as surface water declines and frames *Bacillus* as a sentinel for dormancy in the culturable aerobic fraction

We integrate field series, depth gradients, taxonomic isolates, and mesocosm outcomes into a conceptual model of microbial community disassembly toward dormancy (Fig. 8), extending macroecological disassembly logic from ordered loss of taxa/functions to a trait-mediated endpoint under drying. Community disassembly typically involves predictable, ordered loss of taxa or functions. The Churince water system was home to endemic fauna such as *Eleutherodactylus* sp. nov., *Terrapene coahuila*, and *Cyprinella xanthicara* (Supplementary Fig. 2). Here we extend this framework to microbes (Fig. 8): the dominant signal is a shift in physiological state rather than clear taxon loss. As water declined, the fraction of heat-resistant colonies increased across shoreline sites, while activity remained higher where moisture persisted. Mesocosms that removed phototrophs and heat-sensitive heterotrophs rapidly enriched for spores under shared conditions. Genus-level culture data shifted in composition but retained a core of spore-forming Bacillaceae that rose in relative dominance under drying. Together, these results support a trait-mediated pathway in which loss of energy-supplying guilds and moisture drives communities toward a dormant end state. Bacillaceae serve as a sentinel clade for dormancy dynamics within the culturable aerobic subset.

## Discussion

The desiccation of the Churince system illustrates the ecological consequences of chronic groundwater overexploitation. Surface water loss closely paralleled groundwater decline (Fig. 2), consistent with global patterns of aquifer depletion in arid regions under irrigation pressure [21]. In the Cuatro Ciénegas Basin, alfalfa cultivation has been a major driver of extraction [22, 23]. Despite its Ramsar designation, the Intermediate Lagoon disappeared entirely by 2019, underscoring both local governance failures and the limits of international protection [24]. Satellite imagery and field photography corroborated this trajectory, documenting shoreline retreat and mass mortality of fish and turtles. The sequence of losses, from macrofauna to microbial shifts favoring spore-formers (heat-resistant CFU/Total CFU), reflects a non-random process shaped by desiccation. Ecologists describe this unraveling as community disassembly [15, 16]. While the system is not naturally ephemeral, anthropogenic stress forced a rapid decline resembling ephemeral habitats. Pisanty et al. [25] similarly documented riparian plants in Churince as “canaries,” foreshadowing collapse. Here, the same logic applies hierarchically, from vertebrate loss to microbial shifts dominated by spore-forming Bacillaceae. Before collapse, the Churince system may have functioned as a source habitat within the Cuatro Ciénegas metacommunity; its loss may have fragmented regional microbial connectivity, though we lack dispersal data to test this hypothesis.

Our results support a hierarchical community disassembly during hydrological collapse at Churince that spans macrofauna to microbes. Macrofaunal loss (dead fish, turtle remains, bird tracks) coincides with a trait-mediated microbial shift: the heat-resistant (spore) fraction rises across sites and years; activity tracks moisture along transects and depth; a Bacillaceae-dominated core persists while increasing in dominance under drying; and mesocosms that remove phototrophs and heat-sensitive heterotrophs rapidly converge on spore enrichment. Together, these patterns extend macroecological disassembly logic to microbes by emphasizing an ordered pathway from energetic support and moisture to dormancy, not just taxon loss.

We interpret dormancy as the prevailing endpoint of microbial disassembly under a sustained drying (press disturbance), while noting that it can be reversible upon rewetting. Inference is confined to the aerobic, culturable fraction recovered on our marine-derived medium (predominantly Bacillaceae); we do not claim whole-community coverage or draw conclusions about unmeasured ecosystem processes. Within these bounds, increased spore prevalence provides a physiological conservative indicator of declining realized microbial activity consistent with functional contraction at the ecosystem level.

Bacillaceae serve as a sentinel clade for dormancy dynamics within the culturable aerobic subset. Across a vegetation transect, shoreline series, and sediment core, the pattern was consistent: increasing aridity coincided with reduced proportions of vegetative cells (active fraction) and enrichment of dormant forms, while residual moisture was associated with higher apparent activity. In the sediment core, subsurface layers retained water and supported higher proportions of vegetative cells (estimated by subtracting heat-resistant CFU from total CFU), paralleling reports of viable and metabolically active cells persisting for decades in lake sediments [26]. These findings indicate that spore banks in Cuatro Ciénegas are not inert repositories but dynamic reservoirs, where water and porosity may allow oxygen and nutrients to sustain metabolic activity even after long-term burial.

Taxonomic shifts reinforce this interpretation. A core group of spore-forming Bacillaceae persisted, and within the genus *Bacillus*, some species such as *B. licheniformis* increased, while others, including *B. aequororis*,* B. mesophilum* and *B. paramycoides*, declined. This pattern highlights how species composition changes across ecological phases. Alpha diversity patterns illustrate the trajectory: stable aquatic conditions supported few dominant taxa, transitional phases maximized richness and evenness, and desiccation maintained high richness—likely due to dormant lineages—although evenness decreased. Alpha diversity peaked during the Transition phase, consistent with the Intermediate Disturbance Hypothesis: moderate disturbance can maximize diversity by preventing competitive exclusion while not eliminating sensitive taxa. Given critiques and mixed support for this hypothesis in aquatic systems [27], we use it as a comparative lens rather than a universal rule. Our primary signal remains trait-mediated disassembly toward dormancy, linking disturbance directly to microbial activity states. In this framework, dormancy represents the endpoint of disassembly.

Similar enrichment of spore-formers has been reported in deserts [13, 28, 29] and endangered salt lakes [14, 30, 31], underscoring global relevance. Our longitudinal approach parallels wet-to-dry transitions documented in Tirez lagoon, where desiccation shifted communities toward Bacillales [32], and responses to desiccation pulses in the Atacama [33], and in Cuatro Ciénegas they may preserve lineages otherwise lost to desiccation.

Drying typically entails loss of diversity [34, 35] and interaction breadth [36]. Our mesocosms tested whether guild loss alone can drive dormancy: heat treatment removed phototrophs and heat-sensitive heterotrophs while water was maintained throughout, separating the effect of losing functional groups from the physical effects of drying. Heat-treated systems showed a brief increase in heterotrophic CFU, consistent with a necromass pulse from the initial die-off, followed by rapid spore enrichment (heat-resistant CFU/total CFU), mirroring the field trajectory. Untreated mesocosms retained autotroph-heterotroph linkages and sustained active communities. This reinforces that community disassembly, the non-random loss of functional guilds, can shift microbial assemblages toward dormancy, independent of water loss per se.

Dormancy provides both refuge and constraint: it preserves rare taxa but reduces contribution to ecosystem processes [7, 37, 38]. The culturable spore-forming fraction represents the system’s resilience potential: dormancy functions as a bet-hedging strategy that maintains biodiversity through unfavorable conditions [7]. In some arid systems, increasing aridity correlates with shifts toward desiccation-resistant Bacillota [34, 39]; our results parallel this pattern, supporting Bacillota as sentinels of ecosystem state under water stress. However, spores alone cannot re-establish complex nutrient flows. Self-sustaining function requires phototrophs, heterotrophs, and higher-order interactions; without diverse guilds, spores remain dormant even when water returns. Thus seed banks preserve diversity but cannot guarantee recovery. Restoration requires addressing the root cause: without hydrological recovery, the seed bank remains dormant and ecosystem function cannot be restored.

Our cultivation-based approach recovered only a subset of microbial diversity, specifically organisms capable of growing aerobically on Modified Marine Medium. Although not selective for Bacillota, this medium has been shown to support their growth and efficiently recover diverse taxa, including Bacillota and Actinomycetota [40], thus enabling detection of both active cells and dormant forms. Nevertheless, many inactive bacteria likely rely on alternative survival strategies that were not captured in this study. Molecular methods offer complementary insights: DNA versus RNA comparisons can distinguish active fractions, and functional markers such as *spo0A* track endospore formers [41]. Future research integrating omics and physiology of Bacillaceae will identify genes critical for survival under desiccation. Continued monitoring will be essential, especially if restoration efforts occur, to evaluate the potential for seed banks to reestablish function. Exploring alternative dormancy strategies, such as the Viable but not Culturable State, will also be key [42]. Additionally, direct tests of functional consequences (e.g., oxygen microprofiles, dissolved organic carbon and nutrient fluxes, extracellular enzyme activities, and necromass turnover assays) will be required to evaluate how dormancy alters biogeochemical processes during collapse.

At a broader scale, the disappearance of the Intermediate lagoon at the Churince provides a rare longitudinal view of microbial responses to wetland drying: desiccation extinguishes macrofauna and reconfigures microbial communities toward dormancy. While our study does not directly measure ecosystem fluxes, the observed biological shifts are consistent with reduced functional connectivity and declining resilience. Sporulation percentage offers a low-cost monitoring tool—rising dormancy signals declining ecosystem health before complete collapse. Such assays may reveal tipping points when resilience erodes, paralleling the theory of critical slowing down [43, 44]. In the Churince water system, they were recognized only after collapse, underscoring the importance of monitoring microbial assays in conjunction with hydrological and chemistry monitoring. The collapse also highlights the importance of incorporating microbes into conservation policy. The launch of the IUCN Microbial Conservation Specialist Group [45] marks a milestone, our results illustrate why microbial communities deserve protection, not only as indicators of ecosystem health, but as reservoirs of unique diversity, including the ancient endemic lineages of Cuatro Ciénegas.

Our findings have direct policy implications for the Cuatro Ciénegas Basin: (i) current agricultural water extraction exceeds sustainable limits and has caused irreversible damage to the Churince system; (ii) remaining water bodies require immediate protection from further extraction.

## Conclusion

Desiccation of the Churince Intermediate Lagoon exemplifies hierarchical community disassembly, from vertebrate loss to trait-mediated microbial reorganization toward dormancy. This collapse highlights the potential of simple microbial assays as low-cost early-warning indicators of ecosystem decline. Longitudinal data indicates that increasing aridity promotes dormancy in endospore-forming Bacillota, quantified as heat-resistant CFU, and reduces surface microbial activity. Spores function both as long-term survival structures and, for the first time, as a measurable proxy for metabolic activity under desiccation. This response combines resilience and loss: seed banks preserve rare taxa yet signal reduced activity and ecosystem function. Although ecosystem functions such as nutrient cycling and energy flow were not directly quantified, the microbial changes documented here are consistent with profound functional constraints. Together, these findings reinforce the need for integrated water governance to safeguard desert wetlands and the microbial processes that underpin their persistence.

## Supplementary Information

Below is the link to the electronic supplementary material.

Supplementary Material 1

Supplementary Material 2

## Data Availability

The 16S rRNA gene sequences generated during this study have been deposited in GenBank (NCBI). The corresponding accession numbers are provided in Supplementary Table 7.
