# Supplementary material for: Dormancy as a Signature of Microbial Community Disassembly during Hydrological Collapse in a Desert Oasis
Source: Microb Ecol. 2026 Apr 17;89:114. doi: 10.1007/s00248-026-02744-z (PMC13212791; doi:10.1007/s00248-026-02744-z)
Supplement: Supplementary file 2 [file 248_2026_2744_MOESM1_ESM.docx]

**Supplementary Tables**

**This document contains 12 Supplementary Tables**

Table 1 - Summary of sampling events, ecological phases, analyses performed, and data location (page 2)

Table 2 - Coordinates, CFU counts, and sporulation percentages from Laguna Intermedia (Churince) sampling sites (page 3)

Table 3 - CFU counts from transect sampling in the Churince system (2012) (page 6)

Table 4 - Morphological characterization of colony phenotypes from a 50-cm sediment core at the LI7 sampling site (page 8)

Table 5 - Colony counts and sporulation percentages from a 50-cm sediment core at the LI7 sampling site (page 9)

Table 6 - Monthly groundwater levels of the Intermediate Lagoon (2007–2025) (page 13)

Table 7 - Taxonomic identification of 1,419 culturable bacterial isolates from the Churince hydrological system (2007–2022) (page 15)

Table 8 - Relative and absolute abundances (n) of culturable bacterial genera isolated from the Churince hydrological system (page 84)

Table 9 - Relative and absolute abundances (n) of Bacillus spp. across ecological phases defined by water availability (page 86)

Table 10 - Kruskal–Wallis test of *Bacillus* spp. across ecological phases defined by water availability (page 88)

Table 11 - Colony counts per phenotype from mesocosm experiments under heat-treated (spore-enriched) and non-heated (intact community) conditions (page 89)

Table 12 - Resume of colony counts and sporulation percentages from mesocosm experiments under heat-treated (spore-enriched) and non-heated (intact community) conditions (page 206)

| **Supplementary Table 1.** Summary of sampling events, ecological phases, analyses performed, and data location. | | | | |
| --- | --- | --- | --- | --- |
| **Year** | **Sampling site** | **Ecological phase** | **Analyses performed** | **Data location** |
| 2007 | Intermediate Lagoon (IL) | With Water (surface water present, sediments submerged) | Heat-resistant isolates only (no total CFU counts); 16S rDNA sequencing | Supplementary Table 2, 7; Fig. 6A |
| 2011 | Intermediate Lagoon shoreline (IL1-IL8) | Transition (receding shoreline, declining piezometer) | Total CFU and heat-resistant CFU (sporulation %); 16S rDNA sequencing | Supplementary Table 2, 7; Fig. 5C, 6A |
| 2012 | Transect sotol-lagoon-grassland | Transition (receding shoreline, declining piezometer) | Total CFU and heat-resistant CFU (sporulation %); 16S rDNA sequencing | Supplementary Table 2, 7; Fig. 3A-C, 6A |
| 2012 | Intermediate Lagoon shoreline (IL1-IL8) | Transition (receding shoreline, declining piezometer) | Total CFU and heat-resistant CFU (sporulation %); 16S rDNA sequencing | Supplementary Table 2, 7; Fig. 5C, 6A |
| 2014 | Vertical depth core (IL7) | Transition (surface water present, shoreline retreating) | Total CFU and heat-resistant CFU; 19 morphotypes classified; 16S rDNA sequencing (n=42) | Supplementary Table 4, 5, 7; Fig. 5A-D |
| 2014 | Mesocosm establishment (IL4, IL5, IL8) | Transition (surface water present at collection) | Total CFU and heat-resistant CFU over 2 years; morphotype classification | Supplementary Table 11; Fig. 7, Supplementary Fig. 8 |
| 2019 | Intermediate Lagoon shoreline (IL1-IL8) | Desiccation (no surface water, dry sediments) | Total CFU and heat-resistant CFU (sporulation %); 16S rDNA sequencing | Supplementary Table 2, 7; Fig. 5C, 6A |
| 2022 | Intermediate Lagoon (dry sediments) | Desiccation (no surface water, dry sediments) | Total CFU and heat-resistant CFU (sporulation %); 16S rDNA sequencing | Supplementary Table 2, 7; Fig. 6A |
| 2025 | Intermediate Lagoon (dry sediments) | Desiccation (no surface water, dry sediments) | Total CFU and heat-resistant CFU (sporulation %) | Supplementary Table 2; Fig. 5C |
| Ecological phases defined operationally: With Water = surface water visible in satellite imagery, piezometer >0.3 m, sediments submerged; Transition = receding shoreline visible in satellite imagery, declining piezometer levels, sampling poles relocated toward remaining water; Desiccation = no surface water in satellite imagery, sediments fully exposed and dry. CFU = colony-forming units. Sporulation % = (heat-resistant CFU / total CFU) x 100. All 16S rDNA sequences deposited in GenBank; accession numbers in Supplementary Table 7. | | | | |

| **Supplementary Table 2**. Coordinates, CFU counts, and sporulation percentages from Laguna Intermedia (Churince) sampling sites. | | | | | | | |
| --- | --- | --- | --- | --- | --- | --- | --- |
| **Date** | **Site** | **UFC total** | **Estimated vegetative UFC*** | **UFC from spores**** | **% Sporulation***** | **Latitude (GMS)** | **Longitude (GMS)** |
| 2011_May | LI1 | 660 | 660 | 0 | 0 | 26◦50’52.9’’N | 102◦08’24.1’’W |
| 2011_May | LI2 | 230,303 | 226,000 | 4,303 | 1.9 | 26◦50’51.9’’N | 102◦08’30.2’’W |
| 2011_May | LI3 | 95,151 | 94,939 | 212 | 0.2 | 26◦50’52.2’’N | 102◦08’28.4’’W |
| 2011_May | LI4 | 512,121 | 503,273 | 8,848 | 1.7 | 26◦50’51.9’’N | 102◦08’30.2’’W |
| 2011_May | LI5 | 209,090 | 200,242 | 8,848 | 4.2 | 26◦84’84.1’’N | 102◦14’25.9’’W |
| 2011_May | LI6 | 1,903,030 | 1,901,030 | 2,000 | 0.1 | 26◦50’53.9’’N | 102◦08’29.8’’W |
| 2011_May | LI7 | 1,536,363 | 1,520,091 | 16,272 | 1.1 | 26◦50’54.1’’N | 102◦08’32.3’’W |
| 2011_May | LI8 | 2,424,242 | 2,336,061 | 88,181 | 3.6 | 26◦50’55.2’’N | 102◦08’34.9’’W |
| 2011_Oct | LI1 | 25,000 | 24,404 | 596 | 2.4 | 26◦50’52.9’’N | 102◦08’24.1’’W |
| 2011_Oct | LI2 | 720 | 575 | 145 | 20.1 | 26◦50’51.9’’N | 102◦08’30.2’’W |
| 2011_Oct | LI3 | 63,820 | 63,820 | 0 | 0 | 26◦50’52.2’’N | 102◦08’28.4’’W |
| 2011_Oct | LI4 | 51,400 | 51,355 | 45 | 0.1 | 26◦50’51.9’’N | 102◦08’30.2’’W |
| 2011_Oct | LI5 | 105,694 | 104,594 | 1,100 | 1 | 26◦84’84.1’’N | 102◦14’25.9’’W |
| 2011_Oct | LI6 | 30,300 | 30,189 | 111 | 0.4 | 26◦50’53.9’’N | 102◦08’29.8’’W |
| 2011_Oct | LI7 | 11,900 | 11,546 | 354 | 3 | 26◦50’54.1’’N | 102◦08’32.3’’W |
| 2011_Oct | LI8 | 91,694 | 91,671 | 23 | 0 | 26◦50’55.2’’N | 102◦08’34.9’’W |
| 2012_Feb | LI1 | 8,200 | 8,193 | 7 | 0.1 | 26◦50’52.9’’N | 102◦08’24.1’’W |
| 2012_Feb | LI2 | 139,400 | 138,999 | 401 | 0.3 | 26◦50’51.9’’N | 102◦08’30.2’’W |
| 2012_Feb | LI3 | 253,000 | 252,997 | 3 | 0 | 26◦50’52.2’’N | 102◦08’28.4’’W |
| 2012_Feb | LI4 | 34,600 | 34,476 | 124 | 0.4 | 26◦50’51.9’’N | 102◦08’30.2’’W |
| 2012_Feb | LI5 | 12,280 | 12,180 | 100 | 0.8 | 26◦84’84.1’’N | 102◦14’25.9’’W |
| 2012_Feb | LI6 | 299,400 | 290,988 | 8,412 | 2.8 | 26◦50’53.9’’N | 102◦08’29.8’’W |
| 2012_Feb | LI7 | 40,540 | 40,388 | 152 | 0.4 | 26◦50’54.1’’N | 102◦08’32.3’’W |
| 2012_Feb | LI8 | 2,130,600 | 2,130,277 | 323 | 0 | 26◦50’55.2’’N | 102◦08’34.9’’W |
| 2012_May | LI1 | 23,010 | 22,967 | 43 | 0.2 | 26◦50’52.9’’N | 102◦08’24.1’’W |
| 2012_May | LI2 | 20,760 | 20,619 | 141 | 0.7 | 26◦50’51.9’’N | 102◦08’30.2’’W |
| 2012_May | LI3 | 39,800 | 39,770 | 30 | 0.1 | 26◦50’52.2’’N | 102◦08’28.4’’W |
| 2012_May | LI4 | 8,490 | 8,195 | 295 | 3.5 | 26◦50’51.9’’N | 102◦08’30.2’’W |
| 2012_May | LI5 | 3,670 | 3,465 | 205 | 5.6 | 26◦84’84.1’’N | 102◦14’25.9’’W |
| 2012_May | LI6 | 83,630 | 83,486 | 144 | 0.2 | 26◦50’53.9’’N | 102◦08’29.8’’W |
| 2012_May | LI7 | 23,080 | 22,717 | 363 | 1.6 | 26◦50’54.1’’N | 102◦08’32.3’’W |
| 2012_May | LI8 | 2,720 | 1,855 | 865 | 31.8 | 26◦50’55.2’’N | 102◦08’34.9’’W |
| 2012_Nov | LI1 | 190,100 | 189,549 | 551 | 0.3 | 26◦50’52.9’’N | 102◦08’24.1’’W |
| 2012_Nov | LI2 | 9,520 | 8,514 | 1,006 | 10.6 | 26◦50’51.9’’N | 102◦08’30.2’’W |
| 2012_Nov | LI3 | 243,041 | 242,963 | 78 | 0 | 26◦50’52.2’’N | 102◦08’28.4’’W |
| 2012_Nov | LI4 | 22,330 | 16,798 | 5,532 | 24.8 | 26◦50’51.9’’N | 102◦08’30.2’’W |
| 2012_Nov | LI5 | 166,750 | 164,527 | 2,223 | 1.3 | 26◦84’84.1’’N | 102◦14’25.9’’W |
| 2012_Nov | LI6 | 62,000 | 59,892 | 2,108 | 3.4 | 26◦50’53.9’’N | 102◦08’29.8’’W |
| 2012_Nov | LI7 | 162,000 | 142,576 | 19,424 | 12 | 26◦50’54.1’’N | 102◦08’32.3’’W |
| 2012_Nov | LI8 | 173,900 | 170,436 | 3,464 | 2 | 26◦50’55.2’’N | 102◦08’34.9’’W |
| 2019_Sep | LI1 | 118,000 | 59,000 | 59,000 | 50 | 26◦50’52.9’’N | 102◦08’24.1’’W |
| 2019_Sep | LI2 | 5,990 | 2,880 | 3,110 | 52 | 26◦50’51.9’’N | 102◦08’30.2’’W |
| 2019_Sep | LI3 | 360,000 | 343,500 | 16,500 | 5 | 26◦50’52.2’’N | 102◦08’28.4’’W |
| 2019_Sep | LI4 | 280,000 | 265,300 | 14,700 | 5 | 26◦50’51.9’’N | 102◦08’30.2’’W |
| 2019_Sep | LI5 | 864,640 | 852,640 | 12,000 | 1 | 26◦84’84.1’’N | 102◦14’25.9’’W |
| 2019_Sep | LI6 | 116,000 | 112,600 | 3,400 | 3 | 26◦50’53.9’’N | 102◦08’29.8’’W |
| 2019_Sep | LI8 | 2,110,000 | 2,105,000 | 5,000 | 0.2 | 26◦50’55.2’’N | 102◦08’34.9’’W |
| 2023_Oct | LI1 | 1,230,000 | 1,064,300 | 165,700 | 13.4 | 26◦50’52.9’’N | 102◦08’24.1’’W |
| 2023_Oct | LI2 | 58,000 | 18,900 | 39,100 | 73.8 | 26◦50’51.9’’N | 102◦08’30.2’’W |
| 2023_Oct | LI3 | 69,000 | 61,900 | 7,100 | 10.3 | 26◦50’52.2’’N | 102◦08’28.4’’W |
| 2023_Oct | LI5 | 13,200 | 12,020 | 1,180 | 8.9 | 26◦84’84.1’’N | 102◦14’25.9’’W |
| 2023_Oct | LI6 | 122,000 | 46,000 | 76,000 | 62.3 | 26◦50’53.9’’N | 102◦08’29.8’’W |
| 2023_Oct | LI7 | 460,000 | 260,000 | 200,000 | 43.5 | 26◦50’54.1’’N | 102◦08’32.3’’W |
| 2023_Oct | LI8 | 77,000 | 25,995 | 51,005 | 6.6 | 26◦50’55.2’’N | 102◦08’34.9’’W |
| 2025_Sep | LI1 | 203000 | 134000 | 69000 | 34 | 26◦50’52.9’’N | 102◦08’24.1’’W |
| 2025_Sep | LI2 | 119000 | 68000 | 51000 | 43 | 26◦50’51.9’’N | 102◦08’30.2’’W |
| 2025_Sep | LI3 | 110000 | 101400 | 8600 | 8 | 26◦50’52.2’’N | 102◦08’28.4’’W |
| 2025_Sep | LI4 | 54000 | 28000 | 26000 | 48 | 26◦50’51.9’’N | 102◦08’30.2’’W |
| 2025_Sep | LI5 | 21800 | 15000 | 6800 | 31 | 26◦84’84.1’’N | 102◦14’25.9’’W |
| 2025_Sep | LI6 | 23100 | 14900 | 8200 | 35 | 26◦50’53.9’’N | 102◦08’29.8’’W |
| 2025_Sep | LI7 | 167000 | 117000 | 50000 | 30 | 26◦50’54.1’’N | 102◦08’32.3’’W |
| 2025_Sep | LI8 | 370000 | 298000 | 72000 | 19 | 26◦50’55.2’’N | 102◦08’34.9’’W |
| * * Estimated vegetative CFU count was obtained by subtracting spore-derived CFUs from the total CFU count. | | | | | | | |
| ** Spore-forming CFUs correspond to colonies recovered after heat treatment, which inactivates vegetative cells. | | | | | | | |
| *** Sporulation percentage was calculated as the proportion of spore-forming CFUs relative to the total CFU count, multiplied by 100. | | | | | | | |

| **Supplementary Table 3.** CFU counts from transect sampling in the Churince system (2011). | | | | | |
| --- | --- | --- | --- | --- | --- |
| **Sitie** | **Sample site** | **Geographic coordinates** | | **CFU from Spores** | **Sporulation (%)** |
| Grassland | –6A | 26 50.912N | 102 08.206W | 10,000 | 4 |
| Grassland | –7A | 26 50.913N | 102 08.175W | 7,000 | 28 |
| Grassland | –8A | 26 50.914N | 102 08.145W | 3,300 | 1.3 |
| Grassland | –9A | 26 50.916N | 102 08.114W | 27,000 | 5.4 |
| Grassland | –5B | 26 50.939N | 102 08.244W | 13,000 | 18.6 |
| Grassland | –6B | 26 50.939N | 102 08.213W | 1,400 | 1.4 |
| Grassland | –7B | 26 50.940N | 102 08.183W | 600 | 40 |
| Grassland | –8B | 26 50.941N | 102 08.153W | 16,000 | 24.6 |
| Grassland | –9B | 26 50.941N | 102 08.122W | 4,000 | 26.6 |
| Lagoon | 0A | 26 50.906N | 102 08.546W | 0 | 0 |
| Lagoon | 1A | 26 50.906N | 102 08.576W | 180 | 0.025 |
| Lagoon | 2A | 26 50.904N | 102 08.607W | 1,400 | 23.3 |
| Lagoon | 3A | 26 50.902N | 102 08.637W | 700 | 0.3 |
| Lagoon | 4A | 26 50.901N | 102 08.667W | 1,200 | 1 |
| Lagoon | 0B | 26 50.923N | 102 08.565W | 0 | 0 |
| Lagoon | 1B | 26 50.927N | 102 08.594W | 100 | 0.18 |
| Lagoon | 2B | 26 50.927N | 102 08.622W | 300 | 0.5 |
| Lagoon | 3B | 26 50.920N | 102 08.651W | 1,500 | 7.5 |
| Lagoon | 4B | 26 50.923N | 102 08.682W | 90 | 0.04 |
| Lagoon | –0A | 26 50.908N | 102 08.387W | 1,500 | 0.2 |
| Lagoon | –1A | 26 50.908N | 102 08.360W | 1,500 | 0.6 |
| Lagoon | –2A | 26 50.908N | 102 08.329W | 13,000 | 3.8 |
| Lagoon | –3A | 26 50.909N | 102 08.297W | 7 | 0.003 |
| Lagoon | –4A | 26 50.910N | 102 08.265W | 50 | 0.005 |
| Lagoon | –5A | 26 50.911N | 102 08.236W | 30 | 0.02 |
| Lagoon | –2B | 26 50.935N | 102 08.335W | 1,200 | 0.3 |
| Lagoon | –3B | 26 50.936N | 102 08.305W | 80 | 0.0016 |
| Lagoon | –4B | 26 50.938N | 102 08.275W | 90 | 0.0045 |
| Sotol | 5A | 26 50.900N | 102 08.698W | 310 | 1.3 |
| Sotol | 6A | 26 50.898N | 102 08.727W | 1,600 | 59 |
| Sotol | 7A | 26 50.897N | 102 08.758W | 1,300 | 61.9 |
| Sotol | 8A | 26 50.896N | 102 08.787W | 14,000 | 107.6 |
| Sotol | 9A | 26 50.895N | 102 08.817W | 1,400 | 7 |
| Sotol | 10A | 26 50.893N | 102 08.848W | 1,600 | 133 |
| Sotol | 5B | 26 50.922N | 102 08.713W | 3,700 | 103 |
| Sotol | 6B | 26 50.920N | 102 08.743W | 3,000 | 68 |
| Sotol | 7B | 26 50.919N | 102 08.776W | 80 | 2.5 |
| Sotol | 8B | 26 50.916N | 102 08.804W | 2,500 | 19.2 |
| Sotol | 9B | 26 50.914N | 102 08.833W | 700 | 2.5 |
| Sotol | 10B | 26 50.913N | 102 08.864W | 12,000 | 102 |
| Transect sampling was performed 550 m east (−10) in grassland and 550 m west (+10) in sotol (*Dasylirio*). -dominated habitat. At each distance, two samples (replicates A and B) were collected, with points placed every 50 m starting from the shore of the Intermediate Lagoon. | | | | | |

| **Supplementary Table 4.** Morphological characterization of colony phenotypes from a 50-cm sediment core at the LI7 sampling site. | | | | | |
| --- | --- | --- | --- | --- | --- |
| **Phenotype number** | **Form** | **Margin** | **Elevation** | **Appearance** | **Color** |
| 1 | Circular | Full | Flat | Smooth | Orange |
| 2 | Circular | Wavy | Umbilicate | Rough | Beige |
| 3 | Circular | Full | Flat | Smooth | Cream |
| 4 | Circular | Wavy | Raised | Smooth | Yellow |
| 5 | Circular | Full | Flat | Smooth | Brown |
| 6 | Circular | Wavy | Flat | Smooth | Brown |
| 7 | Circular | Full | Flat | Smooth | Yellow |
| 8 | Irregular | Wavy | Umbilicate | Rough | Red |
| 9 | Circular | Full | Umbilicate | Smooth | Translucent |
| 10 | Circular | Wavy | Raised | Smooth | Beige |
| 11 | Circular | Full | Umbilicate | Smooth | Pink |
| 12 | Circular | Full | Raised | Rough | Beige |
| 13 | Circular | Full | Raised | Rough | Yellow |
| 14 | Circular | Wavy | Umbilicate | Smooth | Cream |
| 15 | Irregular | Lobed | Convex | Rough | Beige |
| 16 | Circular | Wavy | Raised | Rough | Brown |
| 17 | Circular | Wavy | Raised | Rough | Beige |
| 18 | Circular | Wavy | Flat | Smooth | White |
| 19 | Circular | Full | Flat | Smooth | Beige |
| The morphological features of the colonies were examined under a stereoscope | | | | | |

| **Supplementary Table 5.** Colony counts and sporulation percentages from a 50-cm sediment core at the LI7 sampling site. | | | | | | | | |
| --- | --- | --- | --- | --- | --- | --- | --- | --- |
| **Layer** | **Phenotype** | **Count** | **Treatment** | **Dilution** | **UFC/ml** | **Percentage of colonies in the layer** | **TotalCount_per_layer** | **% Total** |
| 1 | 1 | 14 | Direct | 2 | 4.24E+04 | 29.2 | 48 | 100 |
| 1 | 2 | 4 | Direct | 2 | 1.21E+04 | 8.3 | 48 |  |
| 1 | 3 | 28 | Direct | 2 | 8.48E+04 | 58.3 | 48 |  |
| 1 | 4 | 1 | Direct | 2 | 3.03E+03 | 2.1 | 48 |  |
| 1 | 5 | 1 | Direct | 2 | 3.03E+03 | 2.1 | 48 |  |
| 2 | 2 | 14 | Direct | 1 | 4.24E+03 | 42.4 | 33 | 100 |
| 2 | 5 | 3 | Direct | 1 | 9.09E+02 | 9.1 | 33 |  |
| 2 | 6 | 1 | Direct | 1 | 3.03E+02 | 3 | 33 |  |
| 2 | 7 | 1 | Direct | 1 | 3.03E+02 | 3 | 33 |  |
| 2 | 8 | 1 | Direct | 1 | 3.03E+02 | 3 | 33 |  |
| 2 | 9 | 13 | Direct | 1 | 3.94E+03 | 39.4 | 33 |  |
| 3 | 2 | 2 | Direct | 3 | 6.06E+04 | 4.8 | 42 | 100 |
| 3 | 3 | 37 | Direct | 3 | 1.12E+06 | 88.1 | 42 |  |
| 3 | 10 | 3 | Direct | 3 | 9.09E+04 | 7.1 | 42 |  |
| 4 | 2 | 5 | Direct | 3 | 1.52E+05 | 12.2 | 41 | 100 |
| 4 | 9 | 5 | Direct | 3 | 1.52E+05 | 12.2 | 41 |  |
| 4 | 11 | 4 | Direct | 3 | 1.21E+05 | 9.8 | 41 |  |
| 4 | 3 | 27 | Direct | 3 | 8.18E+05 | 65.9 | 41 |  |
| 5 | 1 | 4 | Direct | 3 | 1.21E+05 | 16.7 | 24 | 100 |
| 5 | 3 | 17 | Direct | 3 | 5.15E+05 | 70.8 | 24 |  |
| 5 | 11 | 2 | Direct | 3 | 6.06E+04 | 8.3 | 24 |  |
| 5 | 12 | 1 | Direct | 3 | 3.03E+04 | 4.2 | 24 |  |
| 6 | 11 | 9 | Direct | 3 | 2.73E+05 | 26.5 | 34 | 100 |
| 6 | 2 | 4 | Direct | 3 | 1.21E+05 | 11.8 | 34 |  |
| 6 | 3 | 14 | Direct | 3 | 4.24E+05 | 41.2 | 34 |  |
| 6 | 9 | 3 | Direct | 3 | 9.09E+04 | 8.8 | 34 |  |
| 6 | 13 | 4 | Direct | 3 | 1.21E+05 | 11.8 | 34 |  |
| 7 | 11 | 3 | Direct | 3 | 9.09E+04 | 4.8 | 63 | 100 |
| 7 | 9 | 4 | Direct | 3 | 1.21E+05 | 6.3 | 63 |  |
| 7 | 13 | 5 | Direct | 3 | 1.52E+05 | 7.9 | 63 |  |
| 7 | 2 | 47 | Direct | 3 | 1.42E+06 | 74.6 | 63 |  |
| 7 | 14 | 4 | Direct | 3 | 1.21E+05 | 6.3 | 63 |  |
| 8 | 11 | 18 | Direct | 3 | 5.45E+05 | 24.3 | 74 | 100 |
| 8 | 9 | 2 | Direct | 3 | 6.06E+04 | 2.7 | 74 |  |
| 8 | 4 | 7 | Direct | 3 | 2.12E+05 | 9.5 | 74 |  |
| 8 | 2 | 47 | Direct | 3 | 1.42E+06 | 63.5 | 74 |  |
| 9 | 4 | 4 | Direct | 3 | 1.21E+05 | 25 | 16 | 100 |
| 9 | 2 | 11 | Direct | 3 | 3.33E+05 | 68.8 | 16 |  |
| 9 | 15 | 1 | Direct | 3 | 3.03E+04 | 6.3 | 16 |  |
| 10 | 15 | 5 | Direct | 0 | 1.52E+02 | 29.4 | 17 | 100 |
| 10 | 4 | 2 | Direct | 0 | 6.06E+01 | 11.8 | 17 |  |
| 10 | 2 | 10 | Direct | 0 | 3.03E+02 | 58.8 | 17 |  |
| 1 | 8 | 1 | Heated | 1 | 3.03E+02 | 14.3 | 7 | 100 |
| 1 | 5 | 1 | Heated | 1 | 3.03E+02 | 14.3 | 7 |  |
| 1 | 4 | 4 | Heated | 1 | 1.21E+03 | 57.1 | 7 |  |
| 1 | 16 | 1 | Heated | 1 | 3.03E+02 | 14.3 | 7 |  |
| 2 | 8 | 1 | Heated | 1 | 3.03E+02 | 20 | 5 | 100 |
| 2 | 12 | 2 | Heated | 1 | 6.06E+02 | 40 | 5 |  |
| 2 | 4 | 2 | Heated | 1 | 6.06E+02 | 40 | 5 |  |
| 3 | 8 | 1 | Heated | 1 | 3.03E+02 | 12.5 | 8 | 100 |
| 3 | 16 | 5 | Heated | 1 | 1.52E+03 | 62.5 | 8 |  |
| 3 | 10 | 1 | Heated | 1 | 3.03E+02 | 12.5 | 8 |  |
| 3 | 12 | 1 | Heated | 1 | 3.03E+02 | 12.5 | 8 |  |
| 4 | 8 | 1 | Heated | 1 | 3.03E+02 | 12.5 | 8 | 100 |
| 4 | 16 | 5 | Heated | 1 | 1.52E+03 | 62.5 | 8 |  |
| 4 | 4 | 1 | Heated | 1 | 3.03E+02 | 12.5 | 8 |  |
| 4 | 2 | 1 | Heated | 1 | 3.03E+02 | 12.5 | 8 |  |
| 5 | 2 | 50 | Heated | 1 | 1.52E+04 | 65.8 | 76 | 100 |
| 5 | 17 | 1 | Heated | 1 | 3.03E+02 | 1.3 | 76 |  |
| 5 | 8 | 1 | Heated | 1 | 3.03E+02 | 1.3 | 76 |  |
| 5 | 9 | 24 | Heated | 1 | 7.27E+03 | 31.6 | 76 |  |
| 6 | 3 | 50 | Heated | 2 | 1.52E+05 | 50 | 100 | 100 |
| 6 | 9 | 50 | Heated | 2 | 1.52E+05 | 50 | 100 |  |
| 7 | 3 | 24 | Heated | 2 | 7.27E+04 | 96 | 25 | 100 |
| 7 | 18 | 1 | Heated | 2 | 3.03E+03 | 4 | 25 |  |
| 8 | 8 | 2 | Heated | 1 | 6.06E+02 | 3.2 | 62 | 100 |
| 8 | 1 | 2 | Heated | 1 | 6.06E+02 | 3.2 | 62 |  |
| 8 | 19 | 58 | Heated | 1 | 1.76E+04 | 93.5 | 62 |  |
| 9 | 8 | 5 | Heated | 1 | 1.52E+03 | 45.5 | 11 | 100 |
| 9 | 19 | 3 | Heated | 1 | 9.09E+02 | 27.3 | 11 |  |
| 9 | 3 | 3 | Heated | 1 | 9.09E+02 | 27.3 | 11 |  |
| 10 | 8 | 2 | Heated | 0 | 6.06E+01 | 40 | 5 | 100 |
| 10 | 19 | 3 | Heated | 0 | 9.09E+01 | 60 | 5 |  |

| **Supplementary Table 6.** Monthly groundwater levels of the Intermediate Lagoon (2007–2015). | | |
| --- | --- | --- |
| **Year** | **Month** | **Level (meters)** |
| 2007 | Apr | 0.336 |
| 2007 | May | 0.328 |
| 2007 | June | 0.352 |
| 2007 | Jul | 0.376 |
| 2007 | Aug | 0.392 |
| 2007 | Sep | 0.367 |
| 2007 | Oct | 0.333 |
| 2007 | Nov | 0.326 |
| 2007 | Dec | 0.338 |
| 2008 | Jan | 0.339 |
| 2008 | Feb | 0.326 |
| 2008 | Mar | 0.32 |
| 2008 | Apr | 0.312 |
| 2008 | May | 0.309 |
| 2008 | Jun | 0.309 |
| 2008 | Jul | 0.312 |
| 2008 | Aug | 0.306 |
| 2008 | Sep | 0.299 |
| 2008 | Oct | 0.298 |
| 2008 | Nov | 0.297 |
| 2008 | Dec | 0.293 |
| 2009 | Jan | 0.295 |
| 2009 | Feb | 0.289 |
| 2009 | Mar | 0.281 |
| 2009 | Apr | 0.274 |
| 2009 | May | 0.266 |
| 2009 | Jun | 0.245 |
| 2009 | Jul | 0.229 |
| 2009 | Aug | 0.22 |
| 2009 | Sep | 0.219 |
| 2009 | Oct | 0.193 |
| 2009 | Nov | 0.184 |
| 2009 | Dec | 0.179 |
| 2010 | Jan | 0.175 |
| 2010 | Feb | 0.189 |
| 2010 | Mar | 0.194 |
| 2010 | Apr | 0.175 |
| 2010 | May | 0.142 |
| 2010 | Jun | 0.078 |
| 2010 | Jul | 0.235 |
| 2010 | Aug | 0.202 |
| 2010 | Sep | 0.128 |
| 2010 | Oct | 0.092 |
| 2010 | Nov | 0.08 |
| 2010 | Dec | 0.07 |
| 2011 | Jan | 0.077 |
| 2011 | Feb | 0.089 |
| 2011 | Mar | 0.105 |
| 2011 | Apr | 0.112 |
| 2011 | May | 0.117 |
| 2011 | Jul | 0.061 |
| 2011 | Aug | 0.053 |
| 2012 | Jan–mar | 0 |
| 2012 | Apr–jun | 0 |
| 2012 | Jun–sep | 0 |
| 2012 | Oct–dec | 0 |
| 2013 | May | 0.127 |
| 2013 | Jun | 0.125 |
| 2013 | Jul | 0.12 |
| 2013 | Aug | 0.12 |
| 2013 | Sep | 0.111 |
| 2013 | Oct | 0.157 |
| 2013 | Nov | 0.207 |
| 2014 | Sep | 0.091 |
| 2014 | Oct | 0.084 |
| 2014 | Nov | 0.083 |
| 2014 | Dec | 0.053 |
| 2015 | Jan | 0.05 |
| 2015 | Feb | 0.042 |
| 2015 | Mar | 0.081 |
| 2015 | Apr | 0.085 |
| 2015 | May | 0.048 |
| Groundwater levels were measured using a piezometer installed in the Churince well (Data from Conanp and IMTA) | | |

| **Supplementary Table 7.** Taxonomic identification of 1,419 culturable bacterial isolates from the Churince hydrological system (2007–2022). | | | | | |
| --- | --- | --- | --- | --- | --- |
| **Strain number** | **Sample-ID** | **Year** | **Sampling Site** | **NCBI accession number** | **Taxonomy** |
| 1 | CH100A_3T | 2007 | LI8 | KF966517 | *Bacillus zhangzhouensis* |
| 2 | CH101A_3T | 2007 | LI8 | KF966290 | *Bacillus paramycoides* |
| 3 | CH102_3T | 2007 | LI8 | KF966315 | *Bacillus thuringiensis* |
| 4 | CH103A_3T | 2007 | LI8 | KF966254 | *Peribacillus simplex* |
| 5 | CH103B_3T | 2007 | LI8 | KF966244 | *Bacillus aequororis* |
| 6 | CH104A_3D | 2007 | LI8 | KF966478 | *Rossellomorea marisflavi* |
| 7 | CH104B_3D | 2007 | LI8 | KF966512 | *Bacillus zhangzhouensis* |
| 8 | CH105_3D | 2007 | LI8 | KF966245 | *Bacillus aequororis* |
| 9 | CH107_3D | 2007 | LI8 | KF966476 | *Rossellomorea marisflavi* |
| 10 | CH108_3D_2 | 2007 | LI8 | PQ578877 | *Rossellomorea marisflavi* |
| 11 | CH108_3D_1 | 2007 | LI8 | KF966477 | *Bacillus aequororis* |
| 12 | CH109A_4T | 2007 | LI7 | JF951292 | *Bacillus inaquosorum* |
| 13 | CH109B_4T | 2007 | LI7 | KF966415 | *Bacillus inaquosorum* |
| 14 | CH110_4T | 2007 | LI7 | KF966414 | *Bacillus inaquosorum* |
| 15 | CH111_4T | 2007 | LI7 | JF951232 | *Bacillus thuringiensis* |
| 16 | CH111b_4T | 2007 | LI7 | KF966332 | *Bacillus thuringiensis* |
| 17 | CH112A_4T | 2007 | LI7 | JF951255 | *Bacillus zhangzhouensis* |
| 18 | CH112b_4T | 2007 | LI7 | PV272375 | *Bacillus pakistanensis* |
| 19 | CH113A_4T | 2007 | LI7 | JF951228 | *Metabacillus flavus* |
| 20 | CH113B_4T | 2007 | LI7 | KF966534 | *Metabacillus flavus* |
| 21 | CH115B_4C | 2007 | LI7 | KF966168 | *Lysinibacillus macroides* |
| 22 | CH116_4C | 2007 | LI7 | KF966246 | *Bacillus aequororis* |
| 23 | CH117_4C | 2007 | LI7 | KF966407 | *Bacillus mesophilum* |
| 24 | CH118_4C | 2007 | LI7 | KF966513 | *Bacillus zhangzhouensis* |
| 25 | CH119_4C | 2007 | LI7 | KF966505 | *Bacillus zhangzhouensis* |
| 26 | CH11A | 2007 | Out | KF966522 | *Bacillus stratosphericus* |
| 27 | CH11B | 2007 | Out | KF966532 | *Metabacillus flavus* |
| 28 | CH120A_4D_2 | 2007 | LI7 | PQ578878 | *Bacillus licheniformis* |
| 29 | CH120A_4D_1 | 2007 | LI7 | KF966431 | *Bacillus licheniformis* |
| 30 | CH120B_4D_2 | 2007 | LI7 | PQ578879 | *Bacillus licheniformis* |
| 31 | CH120B_4D_1 | 2007 | LI7 | KF966429 | *Bacillus licheniformis* |
| 32 | CH121A_4D | 2007 | LI7 | KF966316 | *Bacillus proteolyticus* |
| 33 | CH121B_4D | 2007 | LI7 | KF966317 | *Bacillus proteolyticus* |
| 34 | CH122A_4D | 2007 | LI7 | KF966343 | *Bacillus proteolyticus* |
| 35 | CH122B_4D | 2007 | LI7 | KF966351 | *Priestia flexa* |
| 36 | CH123A_4D | 2007 | LI7 | KF966199 | *Sutcliffiella zhanjiangensis* |
| 37 | CH124A_4D | 2007 | LI7 | KF966329 | *Bacillus thuringiensis* |
| 38 | CH126_4D | 2007 | LI7 | KF966310 | *Bacillus wiedmannii* |
| 39 | CH127A_4D | 2007 | LI7 | KF966247 | *Bacillus aequororis* |
| 40 | CH127B_4D | 2007 | LI7 | KF966236 | *Bacillus aequororis* |
| 41 | CH128A_4D | 2007 | LI7 | KF966387 | *Bacillus mesophilum* |
| 42 | CH128B_4D | 2007 | LI7 | KF966455 | *Bacillus pakistanensis* |
| 43 | CH129A_4D_2 | 2007 | LI7 | PQ578880 | *Bacillus aequororis* |
| 44 | CH129A_4D_1 | 2007 | LI7 | KF966226 | *Bacillus aequororis* |
| 45 | CH130A_4D | 2007 | LI7 | KF966434 | *Bacillus licheniformis* |
| 46 | CH130B_4D | 2007 | LI7 | KF966428 | *Bacillus licheniformis* |
| 47 | CH131A_4D | 2007 | LI7 | KF966425 | *Bacillus licheniformis* |
| 48 | CH131B_4D | 2007 | LI7 | KF966426 | *Bacillus licheniformis* |
| 49 | CH132_4D | 2007 | LI7 | KF966384 | *Bacillus mesophilum* |
| 50 | CH133_14D | 2007 | LI7 | KF966386 | *Bacillus mesophilum* |
| 51 | CH134A_4D | 2007 | LI7 | KF966504 | *Bacillus zhangzhouensis* |
| 52 | CH134B_4D | 2007 | LI7 | KF966450 | *Bacillus pakistanensis* |
| 53 | CH134C_4D | 2007 | LI7 | KF966427 | *Bacillus licheniformis* |
| 54 | CH135A_4T | 2007 | LI7 | JF951277 | *Bacillus aequororis* |
| 55 | CH135B_4T | 2007 | LI7 | KF966230 | *Bacillus aequororis* |
| 56 | CH136A_4T | 2007 | LI7 | KF966250 | *Bacillus aequororis* |
| 57 | CH139B_4T | 2007 | LI7 | KF966327 | *Bacillus thuringiensis* |
| 58 | CH140A_4T | 2007 | LI7 | JF951276 | *Bacillus thuringiensis* |
| 59 | CH141A_4T | 2007 | LI7 | KF966248 | *Bacillus aequororis* |
| 60 | CH141B_4T | 2007 | LI7 | KF966463 | *Rossellomorea vietnamensis* |
| 61 | CH142_4T | 2007 | LI7 | JF951263 | *Bacillus mesophilum* |
| 62 | CH143_4T | 2007 | LI7 | KF966223 | *Bacillus aequororis* |
| 63 | CH144A_4T_2 | 2007 | LI7 | PQ578881 | *Bacillus zhangzhouensis* |
| 64 | CH144A_4T_1 | 2007 | LI7 | JF951239 | *Bacillus zhangzhouensis* |
| 65 | CH144B_4T | 2007 | LI7 | JF951283 | *Bacillus zhangzhouensis* |
| 66 | CH145_4T | 2007 | LI7 | JF951259 | *Bacillus zhangzhouensis* |
| 67 | CH145B_4T | 2007 | LI7 | JF951273 | *Bacillus aequororis* |
| 68 | CH146A_4T | 2007 | LI7 | KF966449 | *Bacillus pakistanensis* |
| 69 | CH146B_4T | 2007 | LI7 | KF966454 | *Bacillus pakistanensis* |
| 70 | CH147A_4T | 2007 | LI7 | KF966208 | *Bacillus aequororis* |
| 71 | CH147B_4T | 2007 | LI7 | KF966339 | *Bacillus thuringiensis* |
| 72 | CH148_4T | 2007 | LI7 | JF951287 | *Priestia filamentosa* |
| 73 | CH149a_4T | 2007 | LI7 | JF951304 | *Micrococcus luteus* |
| 74 | CH149B_4T | 2007 | LI7 | KF966154 | *Dietzia cinnamea* |
| 75 | CH150A_4T | 2007 | LI7 | JF951229 | *Bacillus zhangzhouensis* |
| 76 | CH150B_4T | 2007 | LI7 | KF966514 | *Bacillus zhangzhouensis* |
| 77 | CH150B_A_4T | 2007 | LI7 | PQ578882 | *Bacillus zhangzhouensis* |
| 78 | CH150B_B_4T | 2007 | LI7 | KF966515 | *Bacillus safensis* |
| 79 | CH151B_5R | 2007 | LI6 | KF966314 | *Bacillus proteolyticus* |
| 80 | CH152A_5R | 2007 | LI6 | KF966499 | *Rossellomorea aquimaris* |
| 81 | CH153A_5R | 2007 | LI6 | JF951264 | *Bacillus mesophilum* |
| 82 | CH153B_5R | 2007 | LI6 | KF966318 | *Bacillus proteolyticus* |
| 83 | CH154A_4T | 2007 | LI6 | JF951274 | *Staphylococcus pseudoxylosus* |
| 84 | CH155A_5T | 2007 | LI6 | JF951302 | *Bacillus proteolyticus* |
| 85 | CH155B_5T | 2007 | LI6 | KF966311 | *Bacillus hominis* |
| 86 | CH156_5T | 2007 | LI6 | KF966313 | *Bacillus stratosphericus* |
| 87 | CH156B_5T | 2007 | LI6 | KF966373 | *Bacillus proteolyticus* |
| 88 | CH157A_5T | 2007 | LI6 | JF951235 | *Bacillus mesophilum* |
| 89 | CH157B_5T | 2007 | LI6 | KF966397 | *Bacillus thuringiensis* |
| 90 | CH158A_5T | 2007 | LI6 | JF951248 | *Bacillus mesophilum* |
| 91 | CH158B_4T | 2007 | LI6 | PQ578883 | *Bacillus aequororis* |
| 92 | CH159A | 2007 | LI6 | JF951245 | *Rossellomorea aquimaris* |
| 93 | CH159B_5T_2 | 2007 | LI6 | PQ578884 | *Bacillus thuringiensis* |
| 94 | CH159B_5T_1 | 2007 | LI6 | JF951266 | *Bacillus thuringiensis* |
| 95 | CH160A_5T | 2007 | LI6 | JF951227 | *Bacillus zhangzhouensis* |
| 96 | CH160B_5T | 2007 | LI6 | KF966291 | *Bacillus paramycoides* |
| 97 | CH160C_5T | 2007 | LI6 | JF951265 | *Bacillus mobilis* |
| 98 | CH161A_5T | 2007 | LI6 | KF966319 | *Bacillus proteolyticus* |
| 99 | CH161B_5T | 2007 | LI6 | KF966292 | *Bacillus paramycoides* |
| 100 | CH161d_5T_2 | 2007 | LI6 | PQ578885 | *Bacillus mesophilum* |
| 101 | CH161d_5T_1 | 2007 | LI6 | JF951254 | *Bacillus mesophilum* |
| 102 | CH162_5T | 2007 | LI6 | KP340489 | *Bacillus mesophilum* |
| 103 | CH163A_5T | 2007 | LI6 | KF966405 | *Bacillus mesophilum* |
| 104 | CH163B_5T | 2007 | LI6 | KF966383 | *Bacillus mesophilum* |
| 105 | CH164A_5T | 2007 | LI6 | PV272002 | *Bacillus mesophilum* |
| 106 | CH164B_5T | 2007 | LI6 | KF966269 | *Bacillus paramycoides* |
| 107 | CH165A_5C | 2007 | LI6 | KF966169 | *Bacillus paranthracis* |
| 108 | CH165B_5T | 2007 | LI6 | KF966293 | *Lysinibacillus macroides* |
| 109 | CH167_5R | 2007 | LI6 | KF966406 | *Bacillus paramycoides* |
| 110 | CH168A_5R | 2007 | LI6 | KF966382 | *Bacillus mesophilum* |
| 111 | CH168B_5R | 2007 | LI6 | KF966375 | *Bacillus mesophilum* |
| 112 | CH169A_5R | 2007 | LI6 | KF966298 | *Bacillus mesophilum* |
| 113 | CH169B_5R | 2007 | LI6 | KF966406 | *Bacillus paramycoides* |
| 114 | CH16A_1C | 2007 | LI9 | KF966501 | *Rossellomorea aquimaris* |
| 115 | CH16B_1C | 2007 | LI9 | KF966350 | *Thalassobacillus hwangdonensis* |
| 116 | CH170_5R | 2007 | LI6 | KF966375 | *Bacillus mesophilum* |
| 117 | CH170A_5R | 2007 | LI6 | KF966398 | *Bacillus mesophilum* |
| 118 | CH170B_5R | 2007 | LI6 | KF966399 | *Bacillus mesophilum* |
| 119 | CH171A_5R | 2007 | LI6 | KF966353 | *Mesobacillus jeotgali* |
| 120 | CH171B_5R | 2007 | LI6 | KF966165 | *Domibacillus robiginosus* |
| 121 | CH172_5R | 2007 | LI6 | KF966340 | *Bacillus thuringiensis* |
| 122 | CH173A_5R | 2007 | LI6 | KF966320 | *Bacillus proteolyticus* |
| 123 | CH173B_5R | 2007 | LI6 | KF966238 | *Bacillus aequororis* |
| 124 | CH174_5R | 2007 | LI6 | KF966307 | *Bacillus paramycoides* |
| 125 | CH175_5R | 2007 | LI6 | KF966309 | *Bacillus proteolyticus* |
| 126 | CH176A_5C | 2007 | LI6 | KF966346 | *Bacillus thuringiensis* |
| 127 | CH176B_5C | 2007 | LI6 | KF966420 | *Bacillus licheniformis* |
| 128 | CH177_5C | 2007 | LI6 | KF966294 | *Bacillus paramycoides* |
| 129 | CH178_5C | 2007 | LI6 | KF966503 | *Metabacillus indicus* |
| 130 | CH179_5D | 2007 | LI6 | KF966328 | *Bacillus thuringiensis* |
| 131 | CH17A_1C | 2007 | LI9 | KF966502 | *Rossellomorea aquimaris* |
| 132 | CH17B_1C | 2007 | LI9 | PV272190 | *Corynebacterium confusum* |
| 133 | CH180B_5D | 2007 | LI6 | KF966448 | *Bacillus pakistanensis* |
| 134 | CH181_5D | 2007 | LI6 | KF966408 | *Bacillus mesophilum* |
| 135 | CH181B_5D | 2007 | LI6 | KF966295 | *Bacillus paramycoides* |
| 136 | CH182B_5D | 2007 | LI6 | KF966312 | *Bacillus proteolyticus* |
| 137 | CH183_5D | 2007 | LI6 | KF966413 | *Bacillus inaquosorum* |
| 138 | CH184A_5D | 2007 | LI6 | KF966378 | *Bacillus mesophilum* |
| 139 | CH18B_1C | 2007 | LI9 | KF966170 | *Staphylococcus borealis* |
| 140 | CH190A_5D | 2007 | LI6 | KF966500 | *Rossellomorea aquimaris* |
| 141 | CH190B_5D | 2007 | LI6 | KF966263 | *Bacillus thuringiensis* |
| 142 | CH19A_1T | 2007 | LI9 | JF951288 | *Rossellomorea aquimaris* |
| 143 | CH19b_1T | 2007 | LI9 | PV272291 | *Rossellomorea aquimaris* |
| 144 | CH1A | 2007 | In | KF966157 | *Arthrobacter liuii* |
| 145 | CH1B | 2007 | In | KF966158 | *Arthrobacter liuii* |
| 146 | CH2 | 2007 | In | KF966163 | *Kocuria salina* |
| 147 | CH20A_1T | 2007 | LI9 | JF951250 | *Rossellomorea aquimaris* |
| 148 | CH20B_1T | 2007 | LI10 | JF951251 | *Rossellomorea aquimaris* |
| 149 | CH21_1T | 2007 | LI11 | JF951275 | *Bacillus licheniformis* |
| 150 | CH22_1T | 2007 | LI12 | JF951240 | *Bacillus stratosphericus* |
| 151 | CH23_1T | 2007 | LI13 | PQ578887 | *Bacillus stratosphericus* |
| 152 | CH24_1T | 2007 | LI14 | PQ578888 | *Bacillus stratosphericus* |
| 153 | CH25_1T | 2007 | LI15 | PQ578889 | *Bacillus thuringiensis* |
| 154 | CH26A_1T | 2007 | LI16 | PQ578890 | *Citricoccus zhacaiensis* |
| 155 | CH26B_1T | 2007 | LI17 | PQ578891 | *Bacillus thuringiensis* |
| 156 | CH28_1T | 2007 | LI18 | PQ578892 | *Bacillus aequororis* |
| 157 | CH29_1T | 2007 | LI19 | PQ578893 | *Corynebacterium senegalense* |
| 158 | CH3 | 2007 | In | KF966164 | *Kocuria sediminis* |
| 159 | CH30_1T | 2007 | LI9 | PQ578894 | *Rossellomorea aquimaris* |
| 160 | CH311a_11T | 2007 | LI5 | KF966410 | *Bacillus inaquosorum* |
| 161 | CH311B_11T | 2007 | LI5 | KF966348 | *Bacillus proteolyticus* |
| 162 | CH311c_11T | 2007 | LI5 | KF966161 | *Kocuria assamensis* |
| 163 | CH312c_11T | 2007 | LI5 | KF966445 | *Bacillus pakistanensis* |
| 164 | CH313A_11T | 2007 | LI5 | PV272374 | *Bacillus proteolyticus* |
| 165 | CH313b_11T | 2007 | LI5 | KF966412 | *Bacillus stercoris* |
| 166 | CH314_11T | 2007 | LI5 | KF966225 | *Bacillus aequororis* |
| 167 | CH315_11T | 2007 | LI5 | KF966479 | *Rossellomorea marisflavi* |
| 168 | CH316_11T | 2007 | LI5 | KF966270 | *Bacillus paranthracis* |
| 169 | CH317a_11T | 2007 | LI5 | KF966440 | *Bacillus pakistanensis* |
| 170 | CH318_11T | 2007 | LI5 | KF966400 | *Cytobacillus oceanisediminis* |
| 171 | CH319A_11T | 2007 | LI5 | KF966409 | *Cytobacillus oceanisediminis* |
| 172 | CH319b_11T | 2007 | LI5 | KF966272 | *Bacillus paramycoides* |
| 173 | CH32_1T | 2007 | LI9 | KF966464 | *Rossellomorea vietnamensis* |
| 174 | CH320a_11T | 2007 | LI5 | KF966219 | *Bacillus aequororis* |
| 175 | CH320b_11T | 2007 | LI5 | KF966497 | *Rossellomorea aquimaris* |
| 176 | CH321_11T | 2007 | LI5 | KF966284 | *Bacillus paramycoides* |
| 177 | CH322a_11C | 2007 | LI5 | KF966262 | *Bacillus proteolyticus* |
| 178 | CH322b_11C | 2007 | LI5 | KF966331 | *Bacillus thuringiensis* |
| 179 | CH323A_11C | 2007 | LI5 | KF966349 | *Bacillus proteolyticus* |
| 180 | CH323b_11C | 2007 | LI5 | KF966178 | *Staphylococcus ureilyticus* |
| 181 | CH324a_11C | 2007 | LI5 | KF966274 | *Bacillus paramycoides* |
| 182 | CH324B_11C | 2007 | LI5 | KF966176 | *Staphylococcus ureilyticus* |
| 183 | CH324C_11C | 2007 | LI5 | KF966473 | *Bacillus pakistanensis* |
| 184 | CH325a_11C | 2007 | LI5 | KF966283 | *Bacillus paramycoides* |
| 185 | CH325B_11C | 2007 | LI5 | KF966416 | *Bacillus inaquosorum* |
| 186 | CH326a_11D | 2007 | LI5 | KF966200 | *Bacillus aequororis* |
| 187 | CH326b_11D | 2007 | LI5 | KF966510 | *Bacillus safensis* |
| 188 | CH327a_11D | 2007 | LI5 | KF966421 | *Bacillus licheniformis* |
| 189 | CH327B_11D | 2007 | LI5 | KF966275 | *Bacillus paramycoides* |
| 190 | CH328_11D | 2007 | LI5 | KF966335 | *Bacillus thuringiensis* |
| 191 | CH329a_11D | 2007 | LI5 | KF966203 | *Bacillus aequororis* |
| 192 | CH329b_11D | 2007 | LI5 | KF966279 | *Bacillus paramycoides* |
| 193 | CH329c_11D | 2007 | LI5 | KF966215 | *Bacillus aequororis* |
| 194 | CH33_1T | 2007 | LI9 | JF951278 | *Bacillus pakistanensis* |
| 195 | CH330a_11D | 2007 | LI5 | KF966287 | *Bacillus paramycoides* |
| 196 | CH330b_11D | 2007 | LI5 | KF966251 | *Bacillus aequororis* |
| 197 | CH331a_11D | 2007 | LI5 | KF966404 | *Bacillus mesophilum* |
| 198 | CH331b_11D | 2007 | LI5 | KF966533 | *Metabacillus flavus* |
| 199 | CH331c_11D | 2007 | LI5 | KF966286 | *Bacillus paramycoides* |
| 200 | CH332a_11D | 2007 | LI5 | KF966519 | *Bacillus pumilus* |
| 201 | CH332B_11D | 2007 | LI5 | KF966508 | *Bacillus pumilus* |
| 202 | CH333_11D | 2007 | LI5 | KF966451 | *Bacillus pakistanensis* |
| 203 | CH334A_11D | 2007 | LI5 | KF966377 | *Cytobacillus oceanisediminis* |
| 204 | CH334B_11D | 2007 | LI5 | KF966391 | *Cytobacillus oceanisediminis* |
| 205 | CH335A_11D | 2007 | LI5 | KF966393 | *Cytobacillus depressus* |
| 206 | CH335B_11D | 2007 | LI5 | KF966395 | *Cytobacillus depressus* |
| 207 | CH336A_11D | 2007 | LI5 | KF966370 | *Cytobacillus depressus* |
| 208 | CH336B_11D | 2007 | LI5 | KF966167 | *Exiguobacterium profundum* |
| 209 | CH336c_11D | 2007 | LI5 | KF966371 | *Bacillus mesophilum* |
| 210 | CH336D_11D | 2007 | LI5 | KF966344 | *Bacillus proteolyticus* |
| 211 | CH337A_11D | 2007 | LI5 | KF966273 | *Bacillus paramycoides* |
| 212 | CH337b_11D | 2007 | LI5 | KF966466 | *Rossellomorea vietnamensis* |
| 213 | CH338_11D | 2007 | LI5 | KF966341 | *Bacillus proteolyticus* |
| 214 | CH339_11D | 2007 | LI5 | KF966424 | *Bacillus licheniformis* |
| 215 | CH34 | 2007 | LI9 | JF951291 | *Rossellomorea vietnamensis* |
| 216 | CH340_11D | 2007 | LI5 | KF966401 | *Bacillus mesophilum* |
| 217 | CH341b_12C | 2007 | LI4 | KF966277 | *Bacillus paramycoides* |
| 218 | CH342_12C | 2007 | LI4 | KF966186 | *Planococcus antioxidans* |
| 219 | CH343a_12C | 2007 | LI4 | KF966187 | *Planococcus antioxidans* |
| 220 | CH343b_12C | 2007 | LI4 | KF966162 | *Kocuria assamensis* |
| 221 | CH344b_12C | 2007 | LI4 | KF966185 | *Planococcus donghaensis* |
| 222 | CH344c_12C | 2007 | LI4 | KF966369 | *Bacillus mesophilum* |
| 223 | CH345_12C | 2007 | LI4 | KF966188 | *Planococcus antioxidans* |
| 224 | CH346a_12C | 2007 | LI4 | KF966498 | *Rossellomorea aquimaris* |
| 225 | CH346b_12C | 2007 | LI4 | KF966417 | *Bacillus stercoris* |
| 226 | CH347a_12T | 2007 | LI4 | KF966469 | *Rossellomorea vietnamensis* |
| 227 | CH347b_12T | 2007 | LI4 | KF966470 | *Rossellomorea vietnamensis* |
| 228 | CH349a_12T | 2007 | LI4 | KF966418 | *Bacillus stercoris* |
| 229 | CH349b_12T | 2007 | LI4 | KF966439 | *Bacillus pakistanensis* |
| 230 | CH35_1T | 2007 | LI9 | KF966475 | *Rossellomorea vietnamensis* |
| 231 | CH350_12T | 2007 | LI4 | KF966218 | *Bacillus aequororis* |
| 232 | CH351b_12D | 2007 | LI4 | KF966233 | *Bacillus aequororis* |
| 233 | CH351c_12D | 2007 | LI4 | KF966520 | *Bacillus zhangzhouensis* |
| 234 | CH352a_12D | 2007 | LI4 | KF966220 | *Bacillus aequororis* |
| 235 | CH352b_12D | 2007 | LI4 | KF966280 | *Bacillus paramycoides* |
| 236 | CH353_12D | 2007 | LI4 | KF966184 | *Planococcus antioxidans* |
| 237 | CH354_12D | 2007 | LI4 | KF966403 | *Bacillus mesophilum* |
| 238 | CH355a_12D | 2007 | LI4 | KF966438 | *Rossellomorea vietnamensis* |
| 239 | CH355c_12D | 2007 | LI4 | KF966402 | *Bacillus mesophilum* |
| 240 | CH356a_12D | 2007 | LI4 | KF966249 | *Bacillus aequororis* |
| 241 | CH356b_12D | 2007 | LI4 | KF966282 | *Bacillus paramycoides* |
| 242 | CH357_12T | 2007 | LI4 | KF966419 | *Bacillus stercoris* |
| 243 | CH358_12T | 2007 | LI4 | KF966259 | *Metabacillus crassostreae* |
| 244 | CH359a_12T | 2007 | LI4 | KF966411 | *Bacillus spizizenii* |
| 245 | CH36_1T | 2007 | LI9 | JF951269 | *Rossellomorea vietnamensis* |
| 246 | CH360_12T | 2007 | LI4 | KF966472 | *Rossellomorea vietnamensis* |
| 247 | CH361b_12T | 2007 | LI4 | KF966252 | *Peribacillus simplex* |
| 248 | CH362a_12T | 2007 | LI4 | KF966465 | *Rossellomorea vietnamensis* |
| 249 | CH363a_12T | 2007 | LI4 | KF966257 | *Peribacillus simplex* |
| 250 | CH363b_12T | 2007 | LI4 | KF966258 | *Peribacillus simplex* |
| 251 | CH364_12T | 2007 | LI4 | KF966437 | *Rossellomorea vietnamensis* |
| 252 | CH365_12T | 2007 | LI4 | KF966444 | *Bacillus pakistanensis* |
| 253 | CH366_12T | 2007 | LI4 | KF966535 | *Metabacillus flavus* |
| 254 | CH367a_12T | 2007 | LI4 | KF966388 | *Cytobacillus eiseniae* |
| 255 | CH367b_12T | 2007 | LI4 | KF966436 | *Rossellomorea vietnamensis* |
| 256 | CH368_12T | 2007 | LI4 | KF966376 | *Bacillus mesophilum* |
| 257 | CH369_12T | 2007 | LI4 | KF966221 | *Bacillus aequororis* |
| 258 | CH37_1T | 2007 | LI9 | JF951234 | *Bacillus mesophilum* |
| 259 | CH370a_12T | 2007 | LI4 | KF966336 | *Bacillus thuringiensis* |
| 260 | CH370b_12T | 2007 | LI4 | KF966264 | *Bacillus thuringiensis* |
| 261 | CH370c_12T | 2007 | LI4 | KF966193 | *Jeotgalibacillus marinus* |
| 262 | CH371_12T | 2007 | LI4 | KF966195 | *Jeotgalibacillus marinus* |
| 263 | CH371b_12T | 2007 | LI4 | KF966347 | *Bacillus proteolyticus* |
| 264 | CH372_12D | 2007 | LI4 | KF966334 | *Bacillus thuringiensis* |
| 265 | CH373a_12D | 2007 | LI4 | KF966496 | *Rossellomorea aquimaris* |
| 266 | CH373b_12D | 2007 | LI4 | KF966483 | *Rossellomorea aquimaris* |
| 267 | CH374a_12D | 2007 | LI4 | KF966227 | *Bacillus aequororis* |
| 268 | CH374b_12D | 2007 | LI4 | KF966150 | *Pseudomonas nitrititolerans* |
| 269 | CH374c_12D | 2007 | LI4 | KF966155 | *Brachybacterium paraconglomeratum* |
| 270 | CH375a_12D | 2007 | LI4 | KF966372 | *Bacillus mesophilum* |
| 271 | CH375b_12D | 2007 | LI4 | KF966266 | *Bacillus paramycoides* |
| 272 | CH376a_12D | 2007 | LI4 | KF966381 | *Bacillus mesophilum* |
| 273 | CH376B_12D | 2007 | LI4 | KF966217 | *Bacillus aequororis* |
| 274 | CH377_12D | 2007 | LI4 | KF966394 | *Bacillus mesophilum* |
| 275 | CH378_12D | 2007 | LI4 | KF966192 | *Jeotgalibacillus marinus* |
| 276 | CH379a_12D | 2007 | LI4 | KF966210 | *Bacillus aequororis* |
| 277 | CH379b_12D | 2007 | LI4 | KF966490 | *Rossellomorea aquimaris* |
| 278 | CH379c_12D | 2007 | LI4 | KF966202 | *Bacillus aequororis* |
| 279 | CH380b_12D | 2007 | LI4 | KF966493 | *Rossellomorea aquimaris* |
| 280 | CH380c_12D | 2007 | LI4 | KF966271 | *Bacillus paramycoides* |
| 281 | CH381a_12D | 2007 | LI4 | KF966149 | *Paracoccus carotinifaciens* |
| 282 | CH381B_12D | 2007 | LI4 | KF966182 | *Staphylococcus ureilyticus* |
| 283 | CH382A_12D | 2007 | LI4 | PV272376 | *Rossellomorea aquimaris* |
| 284 | CH382b_12D | 2007 | LI4 | KF966482 | *Rossellomorea aquimaris* |
| 285 | CH383a_12D | 2007 | LI4 | KF966389 | *Bacillus mesophilum* |
| 286 | CH383b_12D | 2007 | LI4 | KF966392 | *Bacillus mesophilum* |
| 287 | CH384a_12D | 2007 | LI4 | KF966390 | *Bacillus mesophilum* |
| 288 | CH384b_12D | 2007 | LI4 | KF966285 | *Bacillus paramycoides* |
| 289 | CH385a_12D | 2007 | LI4 | KF966172 | *Staphylococcus casei* |
| 290 | CH385b_12D | 2007 | LI4 | KF966260 | *Bacillus paramycoides* |
| 291 | CH38A_1T | 2007 | LI9 | KF966435 | *Bacillus pakistanensis* |
| 292 | CH38B_1T | 2007 | LI9 | KF966471 | *Rossellomorea vietnamensis* |
| 293 | CH38C_1T | 2007 | LI9 | JF951285 | *Bacillus thuringiensis* |
| 294 | CH39_1T | 2007 | LI9 | KF966160 | *Kocuria assamensis* |
| 295 | CH40_1T | 2007 | LI9 | JF951296 | *Rossellomorea vietnamensis* |
| 296 | CH402_13C | 2007 | LI3 | KF966433 | *Bacillus licheniformis* |
| 297 | CH403b_13C | 2007 | LI3 | KF966521 | *Bacillus stratosphericus* |
| 298 | CH404a_13C | 2007 | LI3 | KF966468 | *Rossellomorea vietnamensis* |
| 299 | CH404B_13C | 2007 | LI3 | KF966153 | *Dietzia cinnamea* |
| 300 | CH405a_13C | 2007 | LI3 | KF966364 | *Bacillus mesophilum* |
| 301 | CH405b_13C | 2007 | LI3 | KF966235 | *Bacillus aequororis* |
| 302 | CH406a_13C | 2007 | LI3 | KF966365 | *Bacillus mesophilum* |
| 303 | CH406b_13C | 2007 | LI3 | KF966302 | *Bacillus paramycoides* |
| 304 | CH406c_13C | 2007 | LI3 | KF966356 | *Bacillus mesophilum* |
| 305 | CH407a_13C | 2007 | LI3 | KF966306 | *Bacillus paramycoides* |
| 306 | CH407B_13C | 2007 | LI3 | KF966484 | *Rossellomorea aquimaris* |
| 307 | CH408a_13C | 2007 | LI3 | KF966177 | *Staphylococcus ureilyticus* |
| 308 | CH408b_13C | 2007 | LI3 | KF966156 | *Brachybacterium paraconglomeratum* |
| 309 | CH408c_13C | 2007 | LI3 | KF966180 | *Staphylococcus ureilyticus* |
| 310 | CH408e_13C | 2007 | LI3 | KF966198 | *Priestia filamentosa* |
| 311 | CH409a_13C | 2007 | LI3 | KF966308 | *Bacillus paramycoides* |
| 312 | CH41A_1T | 2007 | Li9 | KF966190 | *Fictibacillus nanhaiensis* |
| 313 | CH410a_13C | 2007 | LI3 | KF966278 | *Bacillus paramycoides* |
| 314 | CH410b_13C | 2007 | LI3 | KF966360 | *Bacillus mesophilum* |
| 315 | CH411_13C | 2007 | LI3 | KF966354 | *Cytobacillus gottheilii* |
| 316 | CH413_13T | 2007 | LI3 | KF966422 | *Bacillus licheniformis* |
| 317 | CH414A_13T | 2007 | LI3 | KF966175 | *Staphylococcus ureilyticus* |
| 318 | CH414B_13T | 2007 | LI3 | KF966181 | *Staphylococcus ureilyticus* |
| 319 | CH415B_13T | 2007 | LI3 | KF966201 | *Bacillus aequororis* |
| 320 | CH416a_13T | 2007 | LI3 | KF966304 | *Bacillus paramycoides* |
| 321 | CH416B_13T | 2007 | LI3 | KF966214 | *Bacillus aequororis* |
| 322 | CH416c_13T | 2007 | LI3 | KF966206 | *Bacillus aequororis* |
| 323 | CH417_13T | 2007 | LI3 | KF966380 | *Bacillus mesophilum* |
| 324 | CH418b_13T | 2007 | LI3 | PV272377 | *Bacillus aequororis* |
| 325 | CH419a_13T | 2007 | LI3 | KF966452 | *Bacillus pakistanensis* |
| 326 | CH419b_13T | 2007 | LI3 | KF966447 | *Rossellomorea vietnamensis* |
| 327 | CH41B_1T | 2007 | LI9 | JF951256 | *Fictibacillus nanhaiensis* |
| 328 | CH42_1T | 2007 | LI9 | KF966488 | *Rossellomorea aquimaris* |
| 329 | CH420a_13T | 2007 | LI3 | KF966276 | *Bacillus paramycoides* |
| 330 | CH421b_13T | 2007 | LI3 | KF966358 | *Bacillus mesophilum* |
| 331 | CH422a_13T | 2007 | LI3 | KF966443 | *Rossellomorea vietnamensis* |
| 332 | CH423_13T | 2007 | LI3 | KF966194 | *Jeotgalibacillus marinus* |
| 333 | CH423b_13T | 2007 | LI3 | KF966379 | *Bacillus mesophilum* |
| 334 | CH424_13T | 2007 | LI3 | KF966216 | *Bacillus aequororis* |
| 335 | CH425b_13T | 2007 | LI3 | KF966337 | *Bacillus thuringiensis* |
| 336 | CH425c_13T | 2007 | LI3 | KF966467 | *Rossellomorea vietnamensis* |
| 337 | CH426a_13T | 2007 | LI3 | KF966303 | *Bacillus paramycoides* |
| 338 | CH426b_13T | 2007 | LI3 | KF966281 | *Bacillus paramycoides* |
| 339 | CH427_13T | 2007 | LI3 | KF966432 | *Bacillus licheniformis* |
| 340 | CH428a_13T | 2007 | LI3 | KF966305 | *Bacillus paramycoides* |
| 341 | CH428b_13T | 2007 | LI3 | KF966229 | *Bacillus aequororis* |
| 342 | CH428c_13T | 2007 | LI3 | KF966441 | *Bacillus pakistanensis* |
| 343 | CH429a_13T_2 | 2007 | LI3 | KF966485 | *Rossellomorea aquimaris* |
| 344 | CH429a_13T_1 | 2007 | LI3 | KF966485 | *Bacillus aequororis* |
| 345 | CH429b_13T | 2007 | LI3 | KF966486 | *Rossellomorea aquimaris* |
| 346 | CH43_1T | 2007 | LI9 | JF951231 | *Bacillus licheniformis* |
| 347 | CH431a_13D | 2007 | LI3 | KF966366 | *Bacillus mesophilum* |
| 348 | CH431b_13D | 2007 | LI3 | KF966209 | *Bacillus aequororis* |
| 349 | CH434a_13D | 2007 | LI3 | KF966359 | *Bacillus mesophilum* |
| 350 | CH435a_13D | 2007 | LI3 | KF966509 | *Bacillus safensis* |
| 351 | CH435b_13D | 2007 | LI3 | KF966267 | *Bacillus paramycoides* |
| 352 | CH435c_13D | 2007 | LI3 | KF966256 | *Peribacillus simplex* |
| 353 | CH436a_13D | 2007 | LI3 | KF966228 | *Bacillus aequororis* |
| 354 | CH436b_13D | 2007 | LI3 | KF966338 | *Bacillus thuringiensis* |
| 355 | CH437b_13D | 2007 | LI3 | KF966507 | *Bacillus safensis* |
| 356 | CH438b_13D | 2007 | LI3 | KF966253 | *Peribacillus simplex* |
| 357 | CH438c_13D | 2007 | LI3 | KF966330 | *Bacillus thuringiensis* |
| 358 | CH439a_13D | 2007 | LI3 | KF966321 | *Bacillus thuringiensis* |
| 359 | CH44_1T | 2007 | LI9 | JF951257 | *Bacillus aequororis* |
| 360 | CH440a_13D | 2007 | LI3 | KF966211 | *Bacillus aequororis* |
| 361 | CH440b_13D | 2007 | LI3 | KF966357 | *Bacillus mesophilum* |
| 362 | CH441c_13D | 2007 | LI3 | KF966355 | *Bacillus mesophilum* |
| 363 | CH442a_13D | 2007 | LI3 | KF966255 | *Peribacillus simplex* |
| 364 | CH442b_13D | 2007 | LI3 | KF966367 | *Bacillus mesophilum* |
| 365 | CH445_14T | 2007 | LI7 | JF951270 | *Sutcliffiella zhanjiangensis* |
| 366 | CH446_14T | 2007 | LI7 | JF951293 | *Bacillus mesophilum* |
| 367 | CH447_14T | 2007 | LI7 | JF951294 | *Peribacillus acanthi* |
| 368 | CH448A_14T | 2007 | LI7 | JF951281 | *Bacillus aequororis* |
| 369 | CH448B_14T | 2007 | LI7 | JF951268 | *Bacillus paramycoides* |
| 370 | CH449a1_14T | 2007 | LI7 | JF951303 | *Bacillus mesophilum* |
| 371 | CH449a2_14T | 2007 | LI7 | JF951298 | *Bacillus aequororis* |
| 372 | CH449b_14T | 2007 | LI7 | JF951290 | *Bacillus thuringiensis* |
| 373 | CH45_1T | 2007 | LI9 | JF951280 | *Bacillus aequororis* |
| 374 | CH450_14T | 2007 | LI7 | JF951286 | *Metabacillus indicus* |
| 375 | CH451A_14T | 2007 | LI7 | JF951258 | *Rossellomorea vietnamensis* |
| 376 | CH451B_14T | 2007 | LI7 | JF951267 | *Rossellomorea vietnamensis* |
| 377 | CH452A_14T | 2007 | LI7 | JF951244 | *Bacillus mesophilum* |
| 378 | CH452B_14D | 2007 | LI7 | JF951260 | *Bacillus paramycoides* |
| 379 | CH453A_14D | 2007 | LI7 | KF966159 | *Micrococcus luteus* |
| 380 | CH453B_14D | 2007 | LI7 | KF966296 | *Bacillus paramycoides* |
| 381 | CH453C_14D | 2007 | LI7 | KF966495 | *Rossellomorea aquimaris* |
| 382 | CH454A_14D | 2007 | LI7 | KF966524 | *Bacillus stratosphericus* |
| 383 | CH454B_14D | 2007 | LI7 | KF966525 | *Bacillus stratosphericus* |
| 384 | CH455A_14D | 2007 | LI7 | KF966446 | *Bacillus pakistanensis* |
| 385 | CH455B1_14D | 2007 | LI7 | KF966261 | *Bacillus paramycoides* |
| 386 | CH455B2_14D | 2007 | LI7 | KF966300 | *Bacillus paramycoides* |
| 387 | CH456_14D_2 | 2007 | LI7 | KF966323 | *Bacillus thuringiensis* |
| 388 | CH456_14D_1 | 2007 | LI7 | KF966323 | *Bacillus thuringiensis* |
| 389 | CH457A_14D | 2007 | LI7 | KF966363 | *Bacillus mesophilum* |
| 390 | CH457B_14D | 2007 | LI7 | KF966322 | *Bacillus thuringiensis* |
| 391 | CH458A_14D | 2007 | LI7 | KF966205 | *Bacillus aequororis* |
| 392 | CH458B_14D | 2007 | LI7 | KF966324 | *Bacillus thuringiensis* |
| 393 | CH459A_14D | 2007 | LI7 | KF966173 | *Staphylococcus casei* |
| 394 | CH460A_14D | 2007 | LI7 | KF966374 | *Bacillus mesophilum* |
| 395 | CH460B_14D_2 | 2007 | LI7 | PQ578895 | *Bacillus paramycoides* |
| 396 | CH460B_14D_1 | 2007 | LI7 | KF966297 | *Bacillus paramycoides* |
| 397 | CH461A_14D | 2007 | LI7 | KF966352 | *Mesobacillus jeotgali* |
| 398 | CH461B_14D | 2007 | LI7 | KF966494 | *Rossellomorea aquimaris* |
| 399 | CH462A_14D | 2007 | LI7 | KF966191 | *Jeotgalibacillus alimentarius* |
| 400 | CH462B_14D | 2007 | LI7 | KF966362 | *Bacillus mesophilum* |
| 401 | CH463A_14D | 2007 | LI7 | KF966361 | *Bacillus mesophilum* |
| 402 | CH463B_14D | 2007 | LI7 | KF966301 | *Bacillus paramycoides* |
| 403 | CH46A_2A | 2007 | LI2 | KF966299 | *Bacillus paramycoides* |
| 404 | CH46B_2B | 2007 | LI2 | KF966491 | *Rossellomorea aquimaris* |
| 405 | CH47_2B | 2007 | LI2 | KF966189 | *Fictibacillus nanhaiensis* |
| 406 | CH48_2B | 2007 | LI2 | KF966430 | *Bacillus licheniformis* |
| 407 | CH49A_2B | 2007 | LI2 | KF966442 | *Rossellomorea vietnamensis* |
| 408 | CH49B_2B | 2007 | LI2 | KF966456 | *Rossellomorea vietnamensis* |
| 409 | CH50A_2B | 2007 | LI2 | KF966453 | *Rossellomorea vietnamensis* |
| 410 | CH50B_2B | 2007 | LI2 | KF966457 | *Rossellomorea vietnamensis* |
| 411 | CH51A_2B | 2007 | LI2 | KF966458 | *Rossellomorea vietnamensis* |
| 412 | CH51B_2B | 2007 | LI2 | KF966239 | *Bacillus aequororis* |
| 413 | CH51C_2B | 2007 | LI2 | KF966459 | *Rossellomorea vietnamensis* |
| 414 | CH52_2C | 2007 | LI2 | KF966462 | *Rossellomorea vietnamensis* |
| 415 | CH54A_2C | 2007 | LI2 | KF966536 | *Metabacillus flavus* |
| 416 | CH54B_2C | 2007 | LI2 | KF966537 | *Metabacillus flavus* |
| 417 | CH55_2C | 2007 | LI2 | KF966240 | *Bacillus aequororis* |
| 418 | CH57A_2C | 2007 | LI2 | KF966528 | *Bacillus stratosphericus* |
| 419 | CH57B_2C | 2007 | LI2 | KF966527 | *Bacillus stratosphericus* |
| 420 | CH58A_2C | 2007 | LI2 | KF966523 | *Bacillus stratosphericus* |
| 421 | CH59A_2C | 2007 | LI2 | KF966268 | *Bacillus paranthracis* |
| 422 | CH59B_2C | 2007 | LI2 | KF966166 | *Exiguobacterium profundum* |
| 423 | CH6 | 2007 | In | KF966506 | *Bacillus safensis* |
| 424 | CH60A_2C | 2007 | LI2 | KF966326 | *Bacillus thuringiensis* |
| 425 | CH60B_2C | 2007 | LI2 | KF966531 | *Bacillus stratosphericus* |
| 426 | CH61A_2B | 2007 | LI2 | KF966342 | *Bacillus proteolyticus* |
| 427 | CH61B_2B | 2007 | LI2 | KF966212 | *Bacillus aequororis* |
| 428 | CH62_2B | 2007 | LI2 | KF966461 | *Rossellomorea vietnamensis* |
| 429 | CH64A_2B | 2007 | LI2 | KF966183 | *Staphylococcus pseudoxylosus* |
| 430 | CH64B_2B | 2007 | LI2 | KF966474 | *Rossellomorea marisflavi* |
| 431 | CH65_2B | 2007 | LI2 | KF966385 | *Bacillus mesophilum* |
| 432 | CH66_2B | 2007 | LI2 | KF966288 | *Bacillus paramycoides* |
| 433 | CH67_2B | 2007 | LI2 | KF966222 | *Bacillus aequororis* |
| 434 | CH69B_2B | 2007 | LI2 | KF966481 | *Rossellomorea aquimaris* |
| 435 | CH6B | 2007 | In | KF966492 | *Rossellomorea aquimaris* |
| 436 | CH7 | 2007 | In | KF966325 | *Bacillus thuringiensis* |
| 437 | CH70_2B | 2007 | LI2 | KF966174 | *Staphylococcus ureilyticus* |
| 438 | CH71_2B | 2007 | LI2 | KF966368 | *Bacillus mesophilum* |
| 439 | CH72A_2B | 2007 | LI2 | KF966511 | *Bacillus zhangzhouensis* |
| 440 | CH72B_2B | 2007 | LI2 | KF966489 | *Rossellomorea aquimaris* |
| 441 | CH73B_2B | 2007 | LI2 | KF966423 | *Bacillus licheniformis* |
| 442 | CH74B_2B | 2007 | LI2 | KF966529 | *Bacillus stratosphericus* |
| 443 | CH76_3T | 2007 | LI8 | KF966480 | *Rossellomorea aquimaris* |
| 444 | CH77_3T | 2007 | LI8 | KF966179 | *Staphylococcus ureilyticus* |
| 445 | CH78_3T | 2007 | LI8 | KF966232 | *Bacillus aequororis* |
| 446 | CH78A_3T | 2007 | LI8 | KF966237 | *Bacillus aequororis* |
| 447 | CH79_3T | 2007 | LI8 | KF966196 | *Jeotgalibacillus marinus* |
| 448 | CH80_3T | 2007 | LI8 | KF966241 | *Bacillus aequororis* |
| 449 | CH81A_3T | 2007 | LI8 | JF951289 | *Bacillus aequororis* |
| 450 | CH81B_3T | 2007 | LI8 | KF966197 | *Jeotgalibacillus marinus* |
| 451 | CH82A_3T | 2007 | LI8 | KF966530 | *Bacillus stratosphericus* |
| 452 | CH82B_3T | 2007 | LI8 | KF966152 | *Corynebacterium confusum* |
| 453 | CH83A_3T | 2007 | LI8 | KF966231 | *Bacillus aequororis* |
| 454 | CH83B_3T | 2007 | LI8 | KF966242 | *Bacillus aequororis* |
| 455 | CH84_3T | 2007 | LI8 | JF951246 | *Bacillus aequororis* |
| 456 | CH85_3T | 2007 | LI8 | KF966207 | *Bacillus aequororis* |
| 457 | CH87B_3T | 2007 | LI8 | PQ578896 | *Rossellomorea aquimaris* |
| 458 | CH88_3T | 2007 | LI8 | JF951301 | *Bacillus aequororis* |
| 459 | CH89_3T | 2007 | LI8 | JF951300 | *Bacillus aequororis* |
| 460 | CH90_3T | 2007 | LI8 | KF966243 | *Bacillus vallismortis* |
| 461 | CH91B_3T | 2007 | LI8 | JF951271 | *Bacillus stratosphericus* |
| 462 | CH92_3T | 2007 | LI8 | KF966518 | *Bacillus zhangzhouensis* |
| 463 | CH93_3T | 2007 | LI8 | JF951261 | *Bacillus stratosphericus* |
| 464 | CH94_3T | 2007 | LI8 | KF966265 | *Bacillus paranthracis* |
| 465 | CH95A_3T | 2007 | LI8 | JF951272 | *Bacillus zhangzhouensis* |
| 466 | CH95B_3T | 2007 | LI8 | KF966289 | *Bacillus paramycoides* |
| 467 | CH98A_3T | 2007 | LI8 | KF966526 | *Bacillus stratosphericus* |
| 468 | CH98B_3T | 2007 | LI8 | JF951297 | *Bacillus stratosphericus* |
| 469 | CH99A_3T | 2007 | LI8 | KF966224 | *Bacillus aequororis* |
| 470 | CH99B_3T | 2007 | LI8 | JF951282 | *Staphylococcus ureilyticus* |
| 471 | 126_2 | 2011 | e1 | MG896977 | *Pseudomonas sesami* |
| 472 | 1 | 2011 | tular1 | PV272378 | *Paenarthrobacter nitroguajacolicus* |
| 473 | 2 | 2011 | foothill3 | MG896883 | *Bacillus licheniformis* |
| 474 | 3 | 2011 | mezquIte1 | MG896887 | *Bacillus licheniformis* |
| 475 | 4 | 2011 | foothill1 | PV272379 | *Metabacillus niabensis* |
| 476 | 5 | 2011 | foothill1 | MG896795 | *Mesobacillus zeae* |
| 477 | 6 | 2011 | s6 | MG896923 | *Priestia aryabhattai B8W22* |
| 478 | 7 | 2011 | s6 | MG896902 | *Bacillus licheniformis* |
| 479 | 11 | 2011 | mezquIte6 | PV272380 | *Bacillus yapensis* |
| 480 | 12 | 2011 | tular3 | MG896885 | *Bacillus aequororis* |
| 481 | 13 | 2011 | mezquIte6 | MG896881 | *Virgibacillus salarius* |
| 482 | 14 | 2011 | mezquIte1 | MG896839 | *Bacillus spizizenii* |
| 483 | 15 | 2011 | foothill3 | MG896840 | *Bacillus inaquosorum* |
| 484 | 16 | 2011 | tular6 | PV272381 | *Cytobacillus firmus* |
| 485 | 18 | 2011 | s6 | MG896835 | *Bacillus paramycoides* |
| 486 | 19 | 2011 | foothill2 | MG896832 | *Bacillus inaquosorum* |
| 487 | 21 | 2011 | tular6 | PV272382 | *Cytobacillus oceanisediminis* |
| 488 | 22 | 2011 | mezquIte6 | MG896882 | *Bacillus spizizenii* |
| 489 | 24 | 2011 | mezquIte6 | PV272383 | *Bacillus licheniformis* |
| 490 | 25 | 2011 | foothill1 | MG896763 | *Bacillus spizizenii* |
| 491 | 26 | 2011 | tular2 | PV272384 | *Neobacillus niacini* |
| 492 | 28 | 2011 | tular6 | PV272385 | *Bacillus mesophilum* |
| 493 | 29 | 2011 | mezquIte6 | MG896762 | *Bacillus spizizenii* |
| 494 | 31 | 2011 | tular3 | PV272386 | *Kosakonia oryzendophytica* |
| 495 | 32 | 2011 | mezquIte6 | MG896777 | *Bacillus aequororis* |
| 496 | 33 | 2011 | foothill1 | MG896886 | *Bacillus inaquosorum* |
| 497 | 34 | 2011 | foothill1 | MG896767 | *Brevibacterium celere* |
| 498 | 35 | 2011 | tular6 | MG896768 | *Bacillus paramycoides* |
| 499 | 36 | 2011 | s1 | MG896770 | *Fictibacillus phosphorivorans* |
| 500 | 37 | 2011 | s3 | MG896772 | *Sutcliffiella catenulata* |
| 501 | 40 | 2011 | mezquIte1 | MG896790 | *Metabacillus niabensis* |
| 502 | 41 | 2011 | foothill1 | MG896955 | *Pseudomonas sesami* |
| 503 | 43 | 2011 | foothill1 | MG896789 | *Bacillus paramycoides* |
| 504 | 45 | 2011 | foothill3 | MG896787 | *Bacillus inaquosorum* |
| 505 | 46 | 2011 | s6 | PQ578898 | *Bacillus licheniformis* |
| 506 | 47 | 2011 | foothill2 | MG896786 | *Bacillus stercoris* |
| 507 | 48 | 2011 | foothill2 | MG896785 | *Peribacillus frigoritolerans* |
| 508 | 49 | 2011 | mezquIte1 | MG896895 | *Paenibacillus amylolyticus* |
| 509 | 52 | 2011 | foothill1 | MG896896 | *Microbacterium barkeri* |
| 510 | 53 | 2011 | foothill1 | MG896893 | *Sphingobacterium lumbrici* |
| 511 | 56 | 2011 | foothill2 | MG896799 | *Microbacterium arabinogalactanolyticum* |
| 512 | 58 | 2011 | sotolar1 | MG896967 | *Bacillus mesophilum* |
| 513 | 61 | 2011 | foothill1 | MG896791 | *Metabacillus niabensis* |
| 514 | 62 | 2011 | foothill1 | MG896793 | *Bacillus tropicus* |
| 515 | 63 | 2011 | s3 | MG896894 | *Bacillus licheniformis* |
| 516 | 64 | 2011 | s1 | MG896898 | *Robertmurraya siralis* |
| 517 | 65 | 2011 | foothill1 | MG896741 | *Kocuria polaris* |
| 518 | 66 | 2011 | mezquIte6 | MG896740 | *Paenibacillus seodonensis* |
| 519 | 68 | 2011 | tular3 | MG896899 | *Bacillus licheniformis* |
| 520 | 69 | 2011 | tular3 | MG896742 | *Photobacterium arenosum* |
| 521 | 70 | 2011 | s1 | MG896924 | *Bacillus spizizenii* |
| 522 | 71 | 2011 | s1 | MG896897 | *Bacillus spizizenii* |
| 523 | 72 | 2011 | foothill1 | MG896922 | *Brevibacterium celere* |
| 524 | 73 | 2011 | sotolar2 | PV272387 | *Brevibacillus formosus* |
| 525 | 76 | 2011 | s2 | MG896962 | *Cytobacillus firmus* |
| 526 | 77 | 2011 | e2 | PV272388 | *Bacillus zhangzhouensis* |
| 527 | 78 | 2011 | s1 | MG896834 | *Bacillus mesophilum* |
| 528 | 81 | 2011 | e2 | PV272389 | *Bacillus vallismortis* |
| 529 | 83 | 2011 | LI3 | MG896906 | *Citricoccus alkalitolerans* |
| 530 | 86 | 2011 | e2 | PV272390 | *Bacillus inaquosorum* |
| 531 | 87 | 2011 | LI2 | MG896890 | *Jeotgalibacillus marinus* |
| 532 | 90_1 | 2011 | s3 | PV272391 | *Bacillus* |
| 533 | 92 | 2011 | s2 | MG896919 | *Rossellomorea aquimaris* |
| 534 | 94 | 2011 | s2 | MG896820 | *Rheinheimera mangrovi* |
| 535 | 95 | 2011 | s3 | MG896976 | *Exiguobacterium acetylicum* |
| 536 | 97 | 2011 | s2 | PV272392 | *Aeromonas veronii* |
| 537 | 101 | 2011 | s3 | MG896935 | *Brevibacillus formosus* |
| 538 | 104 | 2011 | e2 | MG896957 | *Bacillus zhangzhouensis* |
| 539 | 107 | 2011 | e2 | MG896956 | *Bacillus inaquosorum* |
| 540 | 110 | 2011 | e2 | MG896960 | *Bacillus mesophilum* |
| 541 | 112 | 2011 | LI2 | MG896984 | *Aeromonas veronii* |
| 542 | 115 | 2011 | LI1 | MG896909 | *Solibacillus silvestris* |
| 543 | 116 | 2011 | LI1 | MG896831 | *Aeromonas veronii* |
| 544 | 117 | 2011 | LI7 | MG896930 | *Shewanella putrefaciens* |
| 545 | 119 | 2011 | LI5 | MG896965 | *Brevundimonas subvibrioides* |
| 546 | 120 | 2011 | LI3 | PV272393 | *Rubribacterium polymorphum* |
| 547 | 122 | 2011 | LI6 | MG896973 | *Jeotgalibacillus marinus* |
| 548 | 124 | 2011 | LI4 | MG896963 | *Rubribacterium polymorphum* |
| 549 | 125 | 2011 | e1 | MG896867 | *Pseudomonas sesami* |
| 550 | 126_1 | 2011 | e1 | MG896954 | *Exiguobacterium profundum* |
| 551 | 127 | 2011 | LI5 | MG896814 | *Brevundimonas subvibrioides* |
| 552 | 131 | 2011 | LI5 | MG896889 | *Tabrizicola sediminis* |
| 553 | 133_1 | 2011 | LI6 | MG896904 | *Bacillus gaemokensis* |
| 554 | 134 | 2011 | s3 | PQ578897 | *Planococcus salinus* |
| 555 | 135 | 2011 | s3 | MG896870 | *Bacillus licheniformis* |
| 556 | 137 | 2011 | LI8 | MG896908 | *Pseudomonas sesami* |
| 557 | 138 | 2011 | s3 | MG896934 | *Marinobacter lipolyticus* |
| 558 | 139 | 2011 | traductor | PV272394 | *Jeotgalibacillus marinus* |
| 559 | 140 | 2011 | LI6 | MG896953 | *Pannonibacter phragmitetus* |
| 560 | 142 | 2011 | e2 | PV272395 | *Pseudomonas sesami* |
| 561 | 146 | 2011 | LI7 | MG896812 | *Metabacillus sediminilitoris* |
| 562 | 147 | 2011 | e1 | MG896813 | *Pseudomonas sesami* |
| 563 | 149 | 2011 | LI4 | PV272396 | *Sphingomonas suaedae* |
| 564 | 150 | 2011 | s1 | MG896891 | *Bacillus licheniformis* |
| 565 | 151 | 2011 | LI4 | MG896828 | *Acinetobacter oryzae* |
| 566 | 190 | 2011 | LI4 | PV272397 | *Bacillus* |
| 567 | 192_1 | 2011 | s2 | MG896829 | *Exiguobacterium acetylicum* |
| 568 | 196 | 2011 | LI3 | MG896830 | *Rossellomorea vietnamensis* |
| 569 | 197 | 2011 | LI2 | MG896941 | *Jeotgalibacillus marinus* |
| 570 | 198 | 2011 | LI1 | MG896958 | *Bacillus paramycoides* |
| 571 | 200 | 2011 | s2 | MG896868 | *Aeromonas veronii* |
| 572 | 60a | 2011 | foothill1 | MG896797 | *Bacillus spizizenii* |
| 573 | 09_2011_1_de2_sed_hot_B07.ab1 | 2011 | de2 | PV272398 | *Paenibacillus alvei DSM* |
| 574 | 09_2011_1_de2_sed_hot_C07.ab1 | 2011 | de2 | PV272399 | *Paenibacillus alvei DSM* |
| 575 | 09_2011_2_l5_sed_dir_B07.ab1 | 2011 | LI5 | PV272400 | *Rheinheimera aquimaris* |
| 576 | 09_2011_3_s1_soil_dir_E02.ab1 | 2011 | s1 | MG896842 | *Robertmurraya crescens* |
| 577 | 09_2011_6_l9_soil_hot_C04.ab1 | 2011 | LI9 | PV272401 | *Rossellomorea aquimaris* |
| 578 | 09_2011_7_e1_soil_hot_E05.ab1 | 2011 | e1 | MG896880 | *Bacillus aerophilus* |
| 579 | 09_2011_8_l8_soil_dir_H06.ab1 | 2011 | LI8 | PQ578920 | *Bacillus aequororis* |
| 580 | 09_2011_9_l8_soil_hot_F01.ab1 | 2011 | LI8 | MG896884 | *Metabacillus flavus* |
| 581 | 09_2011_10_l8_soil_hot_G12.ab1 | 2011 | LI8 | MG896928 | *Bacillus coahuilensis m4-4* |
| 582 | 09_2011_11_l1_soil_hot_A06.ab1 | 2011 | LI1 | MG896929 | *Rossellomorea vietnamensis* |
| 583 | 09_2011_11_l1_soil_hot_G05.ab1 | 2011 | LI1 | PQ578900 | *Rossellomorea vietnamensis* |
| 584 | 09_2011_13_d8_sed_hot_E07.ab1 | 2011 | d8 | MG896927 | *Bacillus licheniformis* |
| 585 | 09_2011_14_d8_sed_dir_A05.ab1 | 2011 | d8 | PQ578901 | *Kocuria assamensis* |
| 586 | 09_2011_15_d8_sed_dir_B06.ab1 | 2011 | d8 | PQ578902 | *Bacillus coahuilensis m4-4* |
| 587 | 09_2011_16_l5_soil_hot_A01.ab1 | 2011 | LI5 | PQ578903 | *Bacillus licheniformis* |
| 588 | 09_2011_18_de2_sed_dir_A01.ab1 | 2011 | de2 | MG896852 | *Microbacterium maritypicum* |
| 589 | 09_2011_20_de2_sed_dir_E04.ab1 | 2011 | de2 | PV272402 | *Algoriphagus aquatilis* |
| 590 | 09_2011_21_de2_sed_dir_C05.ab1 | 2011 | de2 | PQ578904 | *Bacillus mojavensis* |
| 591 | 09_2011_21_de2_sed_dir_F04.ab1 | 2011 | de2 | PQ578905 | *Algoriphagus hitonicola* |
| 592 | 09_2011_22_de2_sed_dir_H03.ab1 | 2011 | de2 | PQ578906 | *Algoriphagus taeanensis* |
| 593 | 09_2011_23_de2_sed_dir_E05.ab1 | 2011 | de2 | PV272403 | *Exiguobacterium aurantiacum* |
| 594 | 09_2011_25_l7_soil_dir_H05.ab1 | 2011 | LI7 | PQ578907 | *Exiguobacterium aurantiacum* |
| 595 | 09_2011_26_l7_soil_dir_F12.ab1 | 2011 | LI7 | MG896821 | *Planococcus koreensis* |
| 596 | 09_2011_26_l7_soil_dir_C06.ab1 | 2011 | LI7 | MG896819 | *Algoriphagus aquatilis* |
| 597 | 09_2011_26_l7_soil_dir_C01.ab1 | 2011 | LI7 | MG896818 | *Planococcus koreensis* |
| 598 | 09_2011_27_d9_sed_hot_C02.ab1 | 2011 | d9 | MG896817 | *Metabacillus flavus* |
| 599 | 09_2011_28_d6_H2O_dir_C04.ab1 | 2011 | d5 | MG896815 | *Pararheinheimera chironomi* |
| 600 | 09_2011_29_d5_sed_dir_B05.ab1 | 2011 | d5 | MG896827 | *Rossellomorea vietnamensis* |
| 601 | 09_2011_29_d5_sed_dir_G04.ab1 | 2011 | d5 | PQ578908 | *Bacillus pakistanensis* |
| 602 | 09_2011_30_e2_sed_dir_C07.ab1 | 2011 | e2 | MG896826 | *Rheinheimera pleomorphica* |
| 603 | 09_2011_31_l8_soil_dir_G01.ab1 | 2011 | LI8 | PV272404 | *Bacillus* |
| 604 | 09_2011_32_e2_soil_dir_D01.ab1 | 2011 | e2 | MG896823 | *Rheinheimera pleomorphica* |
| 605 | 09_2011_33_l2_soil_hot_E03.ab1 | 2011 | LI2 | MG896845 | *Brevibacillus formosus* |
| 606 | 09_2011_34_s1_soil_dir_F07.ab1 | 2011 | s1 | MG896825 | *Bacillus aerophilus* |
| 607 | 09_2011_36_l6_soil_dir_E06.ab1 | 2011 | LI6 | PQ578909 | *Exiguobacterium aurantiacum* |
| 608 | 09_2011_38_l4_soil_dir_H12.ab1 | 2011 | LI4 | MG896838 | *Tistrella mobilis* |
| 609 | 09_2011_38_l4_soil_dir_D05.ab1 | 2011 | LI4 | MG896833 | *Tistrella mobilis* |
| 610 | 09_2011_39_s3_soil_dir_G07.ab1 | 2011 | s3 | MG896837 | *Caenispirillum humi* |
| 611 | 09_2011_40_e1_soil_dir_H03.ab1 | 2011 | e1 | MG896841 | *Rheinheimera aquimaris* |
| 612 | 09_2011_43_d6_sed_dir_A02.ab1 | 2011 | d6 | MG896847 | *Rheinheimera sediminis* |
| 613 | 09_2011_45_d6_sed_dir_B05.ab1 | 2011 | d6 | PV272405 | *Erythrobacter donghaensis* |
| 614 | 09_2011_46_d6_sed_dir_B04.ab1 | 2011 | d6 | PV272406 | *Halomonas lionensis* |
| 615 | 09_2011_47_d6_sed_dir_D06.ab1 | 2011 | d6 | PV272407 | *Bacillus* |
| 616 | 09_2011_49_d7_sed_dir_C03.ab1 | 2011 | d7 | MG896851 | *Exiguobacterium mexicanum* |
| 617 | 09_2011_50_d6_H2O_dir_D04.ab1 | 2011 | d6 | MG896844 | *Pararheinheimera chironomi* |
| 618 | 09_2011_51_d9_sed_dir_F06.ab1 | 2011 | d9 | MG896850 | *Bacillus coahuilensis m4-4* |
| 619 | 09_2011_52_d6_H2O_dir_D05.ab1 | 2011 | d6 | PQ578910 | *Rheinheimera pleomorphica* |
| 620 | 09_2011_52_d6_H2O_dir_A04.ab1 | 2011 | d6 | MG896843 | *Rheinheimera aquimaris* |
| 621 | 09_2011_53_d6_H2O_dir_G04.ab1 | 2011 | d6 | MG896864 | *Rheinheimera muenzenbergensis* |
| 622 | 09_2011_54_s3_soil_hot_A04.ab1 | 2011 | s3 | MG896866 | *Bacillus aerophilus* |
| 623 | 09_2011_55_s3_soil_dir_hot_D04.ab1 | 2011 | s3 | PQ578911 | *Rossellomorea aquimaris* |
| 624 | 09_2011_56_d9_sed_dir_F05.ab1 | 2011 | d9 | MG896854 | *Brevundimonas variabilis* |
| 625 | 09_2011_56_d9_sed_hot_G05.ab1 | 2011 | d9 | MG896865 | *Brevundimonas bacteroides* |
| 626 | 09_2011_58_l4_soil_hot_H04.ab1 | 2011 | LI4 | PQ578912 | *Bacillus licheniformis* |
| 627 | 09_2011_59_l4_soil_hot_B03.ab1 | 2011 | LI4 | MG896856 | *Bacillus thuringiensis* |
| 628 | 09_2011_60_d9_sed_dir_A07.ab1 | 2011 | d9 | PV272408 | *Jeotgalibacillus marinus* |
| 629 | 09_2011_61_d5_sed_hot_B02.ab1 | 2011 | d5 | MG896860 | *Rossellomorea vietnamensis* |
| 630 | 09_2011_62_d5_sed_dir_D02.ab1 | 2011 | d5 | MG896859 | *Pannonibacter carbonis* |
| 631 | 09_2011_64_l6_soil_hot_H01.ab1 | 2011 | LI6 | PV272409 | *Bacillus* |
| 632 | 09_2011_64_l6_soil_hot_C05.ab1 | 2011 | LI6 | PQ578913 | *Metabacillus indicus* |
| 633 | 09_2011_67_d3_sed_dir_H07.ab1 | 2011 | d3 | PQ578914 | *Bacillus aerophilus* |
| 634 | 09_2011_68_d3_sed_dir_F05.ab1 | 2011 | d3 | MG896863 | *Rheinheimera riviphila* |
| 635 | 09_2011_70_d9_sed_hot_G03.ab1 | 2011 | d9 | PQ578915 | *Bacillus songklensis* |
| 636 | 09_2011_71_d7_H2O_dir_G02.ab1 | 2011 | d7 | PQ578916 | *Rheinheimera muenzenbergensis* |
| 637 | 09_2011_71_d7_H2O_dir_E04.ab1 | 2011 | d7 | MG896875 | *Rheinheimera aquimaris* |
| 638 | 09_2011_72_d7_H2O_dir_G03.ab1 | 2011 | d7 | PQ578917 | *Rheinheimera riviphila* |
| 639 | 09_2011_74_d9_sed_hot_B04.ab1 | 2011 | d9 | MG896857 | *Bacillus licheniformis* |
| 640 | 09_2011_75_d9_sed_hot_G06.ab1 | 2011 | d9 | PQ578918 | *Bacillus licheniformis* |
| 641 | 09_2011_76_d3_sed_hot_A03.ab1 | 2011 | d3 | PV272410 | *Metabacillus crassostreae* |
| 642 | 09_2011_77_l3_soil_hot_A02.ab1 | 2011 | LI3 | PQ578919 | *Bacillus* |
| 643 | 09_2011_77_l3_soil_hot_B01.ab1 | 2011 | LI3 | PV272411 | *Metabacillus crassostreae* |
| 644 | 09_2011_78_l4_soil_dir_E03.ab1 | 2011 | LI4 | PV272412 | *Bacillus* |
| 645 | 09_2011_78_l4_soil_dir_A05.ab1 | 2011 | LI4 | MG896873 | *Bacillus licheniformis* |
| 646 | 02_2012_16_l5_sed_dir_D10.ab1 | 2012 | LI5 | MG896869 | *Alphaproteobacteria* |
| 647 | 02_2012_17_l5_sed_dir_B09.ab1 | 2012 | LI5 | PV272413 | *Brevundimonas* |
| 648 | 02_2012_20_s2_sed_dir_B11.ab1 | 2012 | s2 | MG896892 | *Sutcliffiella* |
| 649 | 02_2012_25_l4_sed_dir_F07.ab1 | 2012 | LI4 | MG896979 | *Rheinheimera* |
| 650 | 02_2012_26_l4_sed_dir_E08.ab1 | 2012 | LI4 | MG896932 | *Rheinheimera* |
| 651 | 02_2012_3_l8_sed_dir_C09.ab1 | 2012 | LI8 | PV272414 | *Bacillus* |
| 652 | 02_2012_32_l6_sed_dir_A11.ab1 | 2012 | LI6 | MG896822 | *Aeromonas* |
| 653 | 02_2012_33_l6_sed_dir_F10.ab1 | 2012 | LI6 | MG896983 | *Hyphomicrobiales* |
| 654 | 02_2012_35_e2_sed_dir_H09.ab1 | 2012 | e2 | PV272415 | *Rheinheimera* |
| 655 | 02_2012_39_e2_sed_dir_A08.ab1 | 2012 | e2 | MG896816 | *Rhodobacterales* |
| 656 | 02_2012_40_e1_sed_dir_H07.ab1 | 2012 | e1 | PV272416 | *Jeotgalibacillus* |
| 657 | 02_2012_44_e1_sed_dir_A09.ab1 | 2012 | e1 | MG896810 | *Arenimonas* |
| 658 | 02_2012_46_e1_sed_dir_C11.ab1 | 2012 | e1 | MG896907 | *Alkalihalobacillus* |
| 659 | 02_2012_47_l7_sed_dir_D09.ab1 | 2012 | LI7 | MG896920 | *Bacterium* |
| 660 | 02_2012_50_l7_sed_dir_G08.ab1 | 2012 | LI7 | PV272417 | *Bacillus* |
| 661 | 02_2012_51_l7_sed_dir_C08.ab1 | 2012 | LI7 | PV272418 | *Bacillus* |
| 662 | 02_2012_58_PR_sed_dir_D11.ab1 | 2012 | PR | PV272419 | *Bacillus* |
| 663 | 02_2012_60_PR_sed_dir_B08.ab1 | 2012 | PR | MG896824 | *Pseudidiomarina* |
| 664 | 02_2012_62_PR_sed_dir_A10.ab1 | 2012 | PR | PV272420 | *Rossellomorea* |
| 665 | 02_2012_63_PR_sed_dir_C07.ab1 | 2012 | PR | MG896888 | *Bacillus* |
| 666 | 02_2012_64_PR_sed_dir_C10.ab1 | 2012 | PR | MG896903 | *Alkalihalobacillus* |
| 667 | 02_2012_66_PRC_sed_dir_H08.ab1 | 2012 | PRC | PV272421 | *Bacillus* |
| 668 | 02_2012_70_PRC_sed_dir_B07.ab1 | 2012 | PRC | PV272422 | *Bacillus* |
| 669 | 02_2012_71_PRC_sed_dir_G07.ab1 | 2012 | PRC | PV272423 | *Bacillus* |
| 670 | 02_2012_75_l3_sed_dir_G09.ab1 | 2012 | LI3 | MG896969 | *Bowmanella* |
| 671 | 02_2012_76_l3_sed_dir_E11.ab1 | 2012 | LI3 | MG896931 | *Rheinheimera* |
| 672 | 02_2012_79_l1_sed_dir_F09.ab1 | 2012 | LI1 | MG896982 | *Exiguobacterium* |
| 673 | 02_2012_83_l1_sed_dir_E07.ab1 | 2012 | LI1 | MG896933 | *Bacillus* |
| 674 | 02_2012_84_s1_sed_dir_D07.ab1 | 2012 | s1 | MG896918 | *Rheinheimera* |
| 675 | 02_2012_86_s1_sed_dir_F08.ab1 | 2012 | s1 | MG896981 | *Bacillus* |
| 676 | 02_2012_9_s3_sed_dir_D08.ab1 | 2012 | s3 | MG896921 | *Pseudomonas* |
| 677 | 05_2012_101_l2_sed_dir__A11.seq | 2012 | LI2 | MG897011 | *Pseudomonas* |
| 678 | 05_2012_104_l3-2_sed_dir_H01.seq | 2012 | LI3 | MG897009 | *Bowmanella* |
| 679 | 05_2012_106_l3-2_sed_dir__C07.seq | 2012 | LI3 | PV272424 | *Pseudomonas* |
| 680 | 05_2012_107_l3-2_sed_dir__C12.seq | 2012 | LI3 | MG897010 | *Acinetobacter* |
| 681 | 05_2012_112_l3-2_sed_dir_F07.seq | 2012 | LI3 | MG897014 | *Acinetobacter* |
| 682 | 05_2012_117_e1_sed_dir__B10.seq | 2012 | e1 | MG897013 | *Aeromonas* |
| 683 | 05_2012_118_e1_sed_dir_B05.seq | 2012 | e1 | PV272425 | *Paracoccus* |
| 684 | 05_2012_120_e1_sed_dir_F06.seq | 2012 | e1 | PV272426 | *Bacillus* |
| 685 | 05_2012_121_e1_sed_dir_E07.seq | 2012 | e1 | PV272427 | *Aeromonas* |
| 686 | 05_2012_122_e1_sed_dir_D10.seq | 2012 | e1 | MG897015 | *Bacillus* |
| 687 | 05_2012_123_e1_sed_dir_G03.seq | 2012 | e1 | PV272428 | *Pseudomonas* |
| 688 | 05_2012_124_e1_sed_dir_E09.seq | 2012 | e1 | PV272429 | *Cytobacillus* |
| 689 | 05_2012_126_l4_sed_dir__C11.seq | 2012 | LI4 | PV272430 | *Bacillus* |
| 690 | 05_2012_127_l4_sed_dir__A07.seq | 2012 | LI4 | MG896989 | *Bacillus* |
| 691 | 05_2012_129_l4_sed_dir_E11.seq | 2012 | LI4 | PV272431 | *Bacillus* |
| 692 | 05_2012_13_l5_sed_dir_E02.seq | 2012 | LI5 | MG896993 | *Bacillus* |
| 693 | 05_2012_131_l4_sed_dir_D09.seq | 2012 | LI4 | MG896985 | *Bacillus* |
| 694 | 05_2012_131_l4_sed_dir_F04.seq | 2012 | LI4 | PV272432 | *Bacillus* |
| 695 | 05_2012_134_l4_sed_dir_D07.seq | 2012 | LI4 | MG896987 | *Rossellomorea* |
| 696 | 05_2012_134_l4_sed_dir_E08.seq | 2012 | LI4 | MG896986 | *Rossellomorea* |
| 697 | 05_2012_135_l4_sed_dir__C06.seq | 2012 | LI4 | MG896988 | *Bacillus* |
| 698 | 05_2012_136_l4_sed_dir__B04.seq | 2012 | LI4 | PV272433 | *Shewanella* |
| 699 | 05_2012_139_l4_sed_dir_D06.seq | 2012 | LI4 | PV272434 | *Bacillus* |
| 700 | 05_2012_140_l4_sed_dir__C10.seq | 2012 | LI4 | MG896992 | *Aeromonas* |
| 701 | 05_2012_140_s1_sed_dir_H03.seq | 2012 | s1 | PV272435 | *Aeromonas* |
| 702 | 05_2012_141_s1_sed_dir_G02.seq | 2012 | s1 | MG896991 | *Aeromonas* |
| 703 | 05_2012_142_s1_sed_dir_E12.seq | 2012 | s1 | MG896990 | *Bacillus* |
| 704 | 05_2012_144_s1_sed_dir__C01.seq | 2012 | s1 | PV272436 | *Metapseudomonas* |
| 705 | 05_2012_145_s1_sed_dir__C09.seq | 2012 | s1 | PV272437 | *Pannonibacter* |
| 706 | 05_2012_15_l5_sed_dir_D08.seq | 2012 | LI5 | MG896996 | *Bacillus* |
| 707 | 05_2012_150_s1_sed_dir_D11.seq | 2012 | s1 | PV272438 | *Bacillus* |
| 708 | 05_2012_158_l3-1_sed_dir__C05.seq | 2012 | LI3 | PV272439 | *Bacillus* |
| 709 | 05_2012_158_l3-1_sed_dir_F09.seq | 2012 | LI3 | PV272440 | *Bacillus* |
| 710 | 05_2012_16_s3_sed_dir_A01.seq | 2012 | s3 | PV272441 | *Aeromonas* |
| 711 | 05_2012_160_l3-1_sed_dir__B09.seq | 2012 | LI3 | PV272442 | *Uncultured* |
| 712 | 05_2012_161_13-1_sed_dir_F05.seq | 2012 | L3 | PV272443 | *Alphaproteobacteria* |
| 713 | 05_2012_163_l3-1_sed_dir__C04.seq | 2012 | LI3 | PV272444 | *Bowmanella* |
| 714 | 05_2012_165_l3-1_sed_dir_F10.seq | 2012 | LI3 | PV272445 | *Pseudomonas* |
| 715 | 05_2012_166_l3-1_sed_dir_H05.seq | 2012 | LI3 | MG896995 | *Pseudomonas* |
| 716 | 05_2012_17_s3_sed_dir_H11.seq | 2012 | s3 | MG896998 | *Pseudomonas* |
| 717 | 05_2012_171_l7_sed_dir_A03.seq | 2012 | LI7 | PV272446 | *Agrobacterium* |
| 718 | 05_2012_175_l7_sed_dir_A02.seq | 2012 | LI7 | PV272447 | *Paracoccus* |
| 719 | 05_2012_176_l7_sed_dir__A09.seq | 2012 | LI7 | PV272448 | *Tistrella* |
| 720 | 05_2012_177_l7_sed_dir__B12.seq | 2012 | LI7 | PV272449 | *Jeotgalibacillus* |
| 721 | 05_2012_178_l7_sed_dir_H09.seq | 2012 | LI7 | PV272450 | *Uncultured* |
| 722 | 05_2012_18_s3_sed_dir__A04.seq | 2012 | s3 | PV272451 | *Aeromonas* |
| 723 | 05_2012_184_l7_sed_dir__B01.seq | 2012 | LI7 | PV272452 | *Aeromonas* |
| 724 | 05_2012_19_s3_sed_dir_E01.seq | 2012 | s3 | PV272453 | *Acinetobacter* |
| 725 | 05_2012_191_l1_sed_dir_G05.seq | 2012 | LI1 | MG896997 | *Jeotgalibacillus* |
| 726 | 05_2012_192_l1_sed_dir__D01.seq | 2012 | LI1 | PV272454 | *Aeromonas* |
| 727 | 05_2012_193_l1_sed_dir_F01.seq | 2012 | LI1 | PV272455 | *Tistrella* |
| 728 | 05_2012_194_l1_sed_dir_H04.seq | 2012 | LI1 | PV272456 | *Bacillus* |
| 729 | 05_2012_2_s2_sed_dir_F11.seq | 2012 | s2 | MG896804 | *Aeromonas* |
| 730 | 05_2012_22_s3_sed_dir_G01.seq | 2012 | s3 | MG896806 | *Alkalihalobacillus* |
| 731 | 05_2012_24_s3_sed_dir_G06.seq | 2012 | s3 | MG896807 | *Bacillus* |
| 732 | 05_2012_27_s2_sed_dir__C02.seq | 2012 | s2 | PV272457 | *Bacillus* |
| 733 | 05_2012_3_l6_sed_dir__B08.seq | 2012 | LI6 | PV272458 | *Rossellomorea* |
| 734 | 05_2012_31_s2_sed_dir_G11.seq | 2012 | s2 | PV272459 | *Bacillus* |
| 735 | 05_2012_32_s2_sed_dir_G09.seq | 2012 | s2 | MG896802 | *Aeromonas* |
| 736 | 05_2012_33_s2_sed_dir_A05.seq | 2012 | s2 | MG896784 | *Bacillus* |
| 737 | 05_2012_39_e2_sed_dir__B11.seq | 2012 | e2 | MG896781 | *Bacillus* |
| 738 | 05_2012_45_e2_sed_dir__A12.seq | 2012 | e2 | MG896782 | *Acinetobacter* |
| 739 | 05_2012_45_e2_sed_dir_G04.seq | 2012 | e2 | MG896783 | *Acinetobacter* |
| 740 | 05_2012_47_l8_sed_dir_G08.seq | 2012 | LI8 | MG896778 | *Aeromonas* |
| 741 | 05_2012_48_e2_sed_dir_D12.seq | 2012 | e2 | MG896779 | *Uncultured* |
| 742 | 05_2012_5_l6_sed_dir__B06.seq | 2012 | LI6 | PV272460 | *Bacillus* |
| 743 | 05_2012_53_l8_sed_dir__D03.seq | 2012 | LI8 | MG896780 | *Aeromonas* |
| 744 | 05_2012_56_l8_sed_dir_G10.seq | 2012 | LI8 | PV272461 | *Aeromonas* |
| 745 | 05_2012_58_l8_sed_dir__A08.seq | 2012 | LI8 | PV272462 | *Bacillus* |
| 746 | 05_2012_59_l8_sed_dir_E10.seq | 2012 | LI8 | PV272463 | *Bacillus* |
| 747 | 05_2012_6_l5_sed_dir_E05.seq | 2012 | LI5 | MG896760 | *Aeromonas* |
| 748 | 05_2012_63_l8_sed_dir_H08.seq | 2012 | LI8 | PV272464 | *Bacillus* |
| 749 | 05_2012_66_l6a_sed_dir__A10.seq | 2012 | LI6a | MG896758 | *Aeromonas* |
| 750 | 05_2012_67_l6a_sed_dir_H06.seq | 2012 | LI6a | PV272465 | *Bacillus* |
| 751 | 05_2012_69_l6a_sed_dir_G12.seq | 2012 | LI6a | PV272466 | *Acinetobacter* |
| 752 | 05_2012_7_l5_sed_dir__D02.seq | 2012 | LI5 | PV272467 | *Aeromonas* |
| 753 | 05_2012_72_l6a_sed_dir__B02.seq | 2012 | LI6a | PV272468 | *Aeromonas* |
| 754 | 05_2012_76_l6a_sed_dir_H12.seq | 2012 | LI6a | PV272469 | *Bacillus* |
| 755 | 05_2012_78_l3_sed_dir__C08.seq | 2012 | LI3 | MG896937 | *Bacillus* |
| 756 | 05_2012_86_l3_sed_dir_H07.seq | 2012 | LI3 | PV272470 | *Jeotgalibacillus* |
| 757 | 05_2012_89_l2_sed_dir_D05.seq | 2012 | LI2 | PV272471 | *Pseudomonas* |
| 758 | 05_2012_9_l5_sed_dir__B07.seq | 2012 | LI5 | PV272472 | *Aeromonas* |
| 759 | 05_2012_92_l2_sed_dir_D04.seq | 2012 | LI2 | PV272473 | *Acinetobacter* |
| 760 | 05_2012_95_l2_sed_dir_F08.seq | 2012 | LI2 | MG896936 | *Pseudomonas* |
| 761 | b_G07.ab1 | 2012 | LI2 | PV272474 | *Bacillus* |
| 762 | 11_2012_1_d5_sed_dir_A10 | 2012 | D5 | PV272475 | *Aeromonas veronii* |
| 763 | 11_2012_5_d5_sed_dir_A09 | 2012 | D5 | PV272476 | *Erythrobacter donghaensis* |
| 764 | 11_2012_6_d5_sed_dir_E05 | 2012 | D5 | PV272477 | *Aeromonas veronii* |
| 765 | 11_2012_10_d5_sed_dir_H06 | 2012 | D5 | PV272478 | *Erythrobacter donghaensis* |
| 766 | 11_2012_12_e1_sed_dir_F09 | 2012 | E1 | PV272479 | *Jeotgalibacillus marinus* |
| 767 | 11_2012_13_e1_sed_dir_H04 | 2012 | E1 | PV272480 | *Pseudidiomarina taiwanensis* |
| 768 | 11_2012_14_e1_sed_dir_G08 | 2012 | E1 | PV272481 | *Brevibacillus choshinensis* |
| 769 | 11_2012_15_e1_sed_dir_C04 | 2012 | E1 | PV272482 | *Algoriphagus faecimaris* |
| 770 | 11_2012_17_l6_sed_dir_A10 | 2012 | LI6 | PV272483 | *Exiguobacterium aurantiacum* |
| 771 | 11_2012_18_l6_sed_dir_H09 | 2012 | LI6 | PV272484 | *Bacillus inaquosorum* |
| 772 | 11_2012_19_d3_sed_dir_E08 | 2012 | D3 | PV272485 | *Bacillus thuringiensis* |
| 773 | 11_2012_20_d3_sed_dir_C06 | 2012 | D3 | PV272486 | *Enterobacter soli* |
| 774 | 11_2012_22_l6_sed_dir_B07 | 2012 | LI6 | PV272487 | *Rheinheimera aquimaris* |
| 775 | 11_2012_23_l6_sed_dir_B05 | 2012 | LI6 | PV272488 | *Bacillus paramycoides* |
| 776 | 11_2012_24_l6_sed_dir_C07 | 2012 | LI6 | PV272489 | *Bacillus paramycoides* |
| 777 | 11_2012_25_l6_sed_dir_F01 | 2012 | LI6 | PV272490 | *Exiguobacterium alkaliphilum* |
| 778 | 11_2012_26_d7_sed_dir_H08 | 2012 | D7 | PV272491 | *Metabacillus indicus* |
| 779 | 11_2012_27_d7_sed_dir_B04 | 2012 | D7 | PV272492 | *Qipengyuania intermedia* |
| 780 | 11_2012_28_d7_sed_dir_D04 | 2012 | D7 | PV272493 | *Hoeflea alexandrii* |
| 781 | 11_2012_29_d7_sed_dir_E09 | 2012 | D7 | PV272494 | *Erythrobacter donghaensis* |
| 782 | 11_2012_30_d7_sed_dir_F11 | 2012 | D7 | PV272495 | *Halomonas lionensis* |
| 783 | 11_2012_33_d7_sed_dir_D07 | 2012 | D7 | PV272496 | *Brevundimonas fluminis* |
| 784 | 11_2012_35_l5_sed_dir_A08 | 2012 | LI1 | PV272497 | *Rheinheimera pleomorphica* |
| 785 | 11_2012_36_l2_sed_dir_B10 | 2012 | LI2 | PV272498 | *Rossellomorea vietnamensis* |
| 786 | 11_2012_39_l5_sed_dir_G03 | 2012 | LI5 | PV272499 | *Exiguobacterium himgiriensis* |
| 787 | 11_2012_40_l6_sed_dir_E11 | 2012 | LI6 | PV272500 | *Rheinheimera sediminis* |
| 788 | 11_2012_41_l7_sed_dir_G04 | 2012 | LI7 | PV272501 | *Rheinheimera aquimaris* |
| 789 | 11_2012_44_l5_sed_dir_H03 | 2012 | LI5 | PV272502 | *Rheinheimera aquimaris* |
| 790 | 11_2012_45_l4_sed_dir_E09 | 2012 | LI4 | PV272503 | *Salinarimonas ramus* |
| 791 | 11_2012_46_l4_sed_dir_E07 | 2012 | LI4 | PV272504 | *Kocuria rosea* |
| 792 | 11_2012_48_l4_sed_dir_B06 | 2012 | LI4 | PV272505 | *Microbacterium ginsengisoli* |
| 793 | 11_2012_49_d3_sed_dir_A11 | 2012 | LI4 | PV272506 | *Jeotgalibacillus marinus* |
| 794 | 11_2012_50_l4_sed_dir_C09 | 2012 | LI4 | PV272507 | *Bacillus cabrialesii* |
| 795 | 11_2012_51_l4_sed_dir_D03 | 2012 | LI4 | PV272508 | *Nocardioides szechwanensis* |
| 796 | 11_2012_54_s3_sed_dir_A09 | 2012 | S3 | PV272509 | *Aeromonas veronii* |
| 797 | 11_2012_56_s3_sed_dir_D11 | 2012 | S3 | PV272510 | *Shewanella putrefaciens* |
| 798 | 11_2012_57_s3_sed_dir_G11 | 2012 | S3 | PV272511 | *Erythrobacter tepidarius* |
| 799 | 11_2012_58_s3_sed_dir_A07 | 2012 | S3 | PV272512 | *Jeotgalibacillus malaysiensis* |
| 800 | 11_2012_58_s3_sed_dir_A07 | 2012 | S3 | PV272512 | *Jeotgalibacillus malaysiensis* |
| 801 | 11_2012_59_l5_sed_dir_D04 | 2012 | LI5 | PV272513 | *Paracoccus carotinifaciens* |
| 802 | 11_2012_60_l5_sed_dir_G12 | 2012 | LI5 | PV272514 | *Rheinheimera aquimaris* |
| 803 | 11_2012_61_l5_sed_dir_E02 | 2012 | LI5 | PV272515 | *Nesterenkonia jeotgali* |
| 804 | 11_2012_62_l5_sed_dir_A05 | 2012 | LI5 | PV272516 | *Rheinheimera pleomorphica* |
| 805 | 11_2012_63_l5_sed_dir_A11 | 2012 | LI5 | PV272517 | *Zobellella taiwanensis* |
| 806 | 11_2012_64_l5_sed_dir_A06 | 2012 | LI5 | PV272518 | *Pannonibacter indicus* |
| 807 | 11_2012_65_d3_sed_dir_A04 | 2012 | D3 | PV272519 | *Bacillus inaquosorum* |
| 808 | 11_2012_69_d3_sed_dir_E07 | 2012 | D3 | PV272520 | *Jeotgalibacillus marinus* |
| 809 | 11_2012_70_d3_sed_dir_H05 | 2012 | D3 | PV272521 | *Rossellomorea vietnamensis* |
| 810 | 11_2012_73_d8_sed_dir_A05 | 2012 | D8 | PV272522 | *Bacillus licheniformis* |
| 811 | 11_2012_74_d8_sed_dir_G05 | 2012 | D8 | PV272523 | *Citrobacter arsenatis* |
| 812 | 11_2012_75_d8_sed_dir_A02 | 2012 | D8 | PV272524 | *Brevundimonas bacteroides* |
| 813 | 11_2012_76_d8_sed_dir_F04 | 2012 | D8 | PV272525 | *Aerococcus viridans* |
| 814 | 11_2012_77_d8_sed_dir_F11 | 2012 | D8 | PV272526 | *Aeromonas veronii* |
| 815 | 11_2012_78_d8_sed_dir_C12 | 2012 | D8 | PV272527 | *Sphingobium abikonense* |
| 816 | 11_2012_81_l2_sed_dir_D08 | 2012 | LI2 | PV272528 | *Aeromonas veronii* |
| 817 | 11_2012_82_l2_sed_dir_D09 | 2012 | LI2 | PV272529 | *Exiguobacterium himgiriensis* |
| 818 | 11_2012_88_l2_sed_dir_C10 | 2012 | LI2 | PV272530 | *Aeromonas veronii* |
| 819 | 11_2012_89_l2_sed_dir_H05 | 2012 | LI2 | PV272531 | *Bacillus pakistanensis* |
| 820 | 11_2012_90_s2_sed_dir_G08 | 2012 | S2 | PV272532 | *Aeromonas veronii* |
| 821 | 11_2012_91_l7_sed_dir_C08 | 2012 | LI7 | PV272533 | *Pontibacter lucknowensis* |
| 822 | 11_2012_92_l7_sed_dir_B01 | 2012 | LI7 | PV272534 | *Acinetobacter bereziniae* |
| 823 | 11_2012_93_s1_sed_dir_B08 | 2012 | S1 | PV272535 | *Streptomyces sindenensis* |
| 824 | 11_2012_94_s1_sed_dir_D09 | 2012 | S1 | PV272536 | *Paracoccus amoyensis* |
| 825 | 11_2012_96_s1_sed_dir_C01 | 2012 | S1 | PV272537 | *Paracoccus carotinifaciens* |
| 826 | 11_2012_97_s1_sed_dir_H11 | 2012 | S1 | PV272538 | *Rheinheimera aquimaris* |
| 827 | 11_2012_98_s1_sed_dir_G06 | 2012 | S1 | PV272539 | *Rheinheimera aquimaris* |
| 828 | 11_2012_99_s1_sed_dir_F03 | 2012 | S1 | PV272540 | *Streptomyces bacillaris* |
| 829 | 11_2012_101_e2_sed_dir_F03 | 2012 | E2 | PV272541 | *Arsukibacterium perlucidum* |
| 830 | 11_2012_102_e2_sed_dir_C09 | 2012 | E2 | PV272542 | *Kocuria polaris* |
| 831 | 11_2012_103_e1_sed_dir_C06 | 2012 | E2 | PV272543 | *Halomonas janggokensis* |
| 832 | 11_2012_104_e2_sed_dir_D11 | 2012 | E2 | PV272544 | *Rheinheimera aquimaris* |
| 833 | 11_2012_105_e2_sed_dir_C11 | 2012 | E2 | PV272545 | *Rheinheimera aquimaris* |
| 834 | 11_2012_107_e2_sed_dir_D10 | 2012 | E2 | PV272546 | *Halomonas subterranea* |
| 835 | 11_2012_115_l5_sed_dir_A03 | 2012 | LI5 | PV272547 | *Erythrobacter tepidarius* |
| 836 | 11_2012_117_l7_sed_dir_H06 | 2012 | LI7 | PV272548 | *Vibrio cholerae* |
| 837 | 11_2012_118_l7_sed_dir_B10 | 2012 | LI7 | PV272549 | *Rheinheimera pleomorphica* |
| 838 | 11_2012_119_l7_sed_dir_C08 | 2012 | LI7 | PV272550 | *Planococcus salinarum* |
| 839 | 11_2012_121_l7_sed_dir_D07 | 2012 | LI7 | PV272551 | *Paracoccus carotinifaciens* |
| 840 | 11_2012_122_l7_sed_dir_H07 | 2012 | LI7 | PV272552 | *Arthrobacter subterraneus* |
| 841 | 11_2012_123_d7a_sed_dir_H04 | 2012 | D7 | PV272553 | *Bacillus proteolyticus* |
| 842 | 11_2012_125_d7a_sed_dir_C11 | 2012 | D7 | PV272554 | *Vibrio cholerae* |
| 843 | 11_2012_126_d7a_sed_dir_E10 | 2012 | D7 | PV272555 | *Bacillus coahuilensis m4-4* |
| 844 | 11_2012_127_d7a_sed_dir_H09 | 2012 | D7 | PV272556 | *Aeromonas veronii* |
| 845 | 11_2012_128_d7a_sed_dir_E04 | 2012 | D7 | PV272557 | *Bacillus thuringiensis* |
| 846 | 11_2012_129_d7a_sed_dir_B09 | 2012 | D7 | PV272558 | *Bacillus thuringiensis* |
| 847 | 11_2012_132_s2_sed_dir_C10 | 2012 | S2 | PV272559 | *Aeromonas veronii* |
| 848 | 11_2012_135_d6_sed_dir_H12 | 2012 | D6 | PV272560 | *Bacillus pakistanensis* |
| 849 | 11_2012_136_s2_sed_dir_H01 | 2012 | S2 | PV272561 | *Bacillus proteolyticus* |
| 850 | 11_2012_138_d6_sed_dir_F12 | 2012 | D6 | PV272562 | *Aeromonas veronii* |
| 851 | 11_2012_139_s2_sed_dir_E12 | 2012 | S2 | PV272563 | *Aeromonas veronii* |
| 852 | 11_2012_142_l3_sed_dir | 2012 | LI3 | PV272564 | *Pseudidiomarina salinarum* |
| 853 | 11_2012_144_l3_sed_dir_A12 | 2012 | LI3 | PV272565 | *Pelagibacterium halotolerans B2* |
| 854 | 11_2012_145_l3_sed_dir | 2012 | LI3 | PV272566 | *Bowmanella yangjiangensis* |
| 855 | 11_2012_146_l3_sed_dir_E10 | 2012 | LI3 | PV272567 | *Halomonas piezotolerans* |
| 856 | 11_2012_147_l3_sed_dir | 2012 | LI3 | PV272568 | *Muricauda aquimarina* |
| 857 | 11_2012_149_l8_sed_dir | 2012 | LI8 | PV272569 | *Streptomyces sindenensis* |
| 858 | 11_2012_150_l8_sed_dir_H10 | 2012 | LI8 | PV272570 | *Jeotgalibacillus alimentarius* |
| 859 | 11_2012_151_l8_sed_dir_G12 | 2012 | LI8 | PV272571 | *Jeotgalibacillus marinus* |
| 860 | 11_2012_152_l8_sed_dir | 2012 | LI8 | PV272572 | *Halomonas arcis* |
| 861 | 11_2012_153_l8_sed_dir | 2012 | LI8 | PV272573 | *Sphingomonas aestuarii* |
| 862 | 11_2012_154_d6_sed_dir_H12 | 2012 | D6 | PV272574 | *Aeromonas veronii* |
| 863 | 11_2012_155_d6_sed_dir | 2012 | D6 | PV272575 | *Bacillus proteolyticus* |
| 864 | 11_2012_156_s2_sed_dir_F12 | 2012 | S2 | PV272576 | *Aeromonas veronii* |
| 865 | 11_2012_157_s2_sed_dir_B12 | 2012 | S2 | PV272577 | *Shewanella putrefaciens* |
| 866 | 0A_1 | 2012 | Transect_0A_1 | PV272578 | *Bacillus thuringiensis* |
| 867 | 0A_10 | 2012 | Transect_0A_10 | MG897090 | *Cytobacillus firmus* |
| 868 | 0A_11 | 2012 | Transect_0A_11 | MG897068 | *Rheinheimera lutimaris* |
| 869 | 0A_12 | 2012 | Transect_0A_12 | MG897074 | *Paracoccus carotinifaciens* |
| 870 | 0A_2 | 2012 | Transect_0A_2 | PV272579 | *Chryseomicrobium palamuruense* |
| 871 | 0A_3 | 2012 | Transect_0A_3 | PV272580 | *Rossellomorea vietnamensis* |
| 872 | 0A_4 | 2012 | Transect_0A_4 | MG897119 | *Aeromonas allosaccharophila* |
| 873 | 0A_5 | 2012 | Transect_0A_5 | PV272581 | *Rheinheimera riviphila* |
| 874 | 0A_6 | 2012 | Transect_0A_6 | PV272582 | *Bacillus songklensis* |
| 875 | 0A_7 | 2012 | Transect_0A_7 | PV272583 | *Erythrobacter tepidarius* |
| 876 | 0A_8 | 2012 | Transect_0A_8 | PV272584 | *Gemmobacter lutimaris* |
| 877 | 0B_1 | 2012 | Transect_0B_1 | PV272585 | *Citricoccus yambaruensis* |
| 878 | 0B_2 | 2012 | Transect_0B_2 | PV272586 | *Bacillus aerophilus* |
| 879 | 0B_3 | 2012 | Transect_0B_3 | PV272587 | *Paenibacillus thiaminolyticus* |
| 880 | 0B_4 | 2012 | Transect_0B_4 | PV272588 | *Rheinheimera riviphila* |
| 881 | 0B_5 | 2012 | Transect_0B_5 | MG897092 | *Paracoccus chinensis* |
| 882 | 0B_6 | 2012 | Transect_0B_6 | MG897096 | *Bacillus paramycoides* |
| 883 | 10A_1 | 2012 | Transect_10A_1 | PV272589 | *Bacillus cabrialesii* |
| 884 | 10A_10 | 2012 | Transect_10A_10 | MG897103 | *Paenibacillus popilliae* |
| 885 | 10A_11 | 2012 | Transect_10A_11 | MG897105 | *Sutcliffiella zhanjiangensis* |
| 886 | 10A_2 | 2012 | Transect_10A_2 | PV272590 | *Planococcus halotolerans* |
| 887 | 10A_3 | 2012 | Transect_10A_3 | MG897123 | *Planococcus halotolerans* |
| 888 | 10A_4 | 2012 | Transect_10A_4 | PV272591 | *Micrococcus yunnanensis* |
| 889 | 10A_5 | 2012 | Transect_10A_5 | PV272592 | *Bacillus licheniformis* |
| 890 | 10A_6 | 2012 | Transect_10A_6 | MG897117 | *Bacillus licheniformis* |
| 891 | 10A_8 | 2012 | Transect_10A_8 | PV272593 | *Planococcus antioxidans* |
| 892 | 10A_9 | 2012 | Transect_10A_9 | PV272594 | *Pontibacter salisaro* |
| 893 | 10B_1 | 2012 | Transect_10B_1 | PV272595 | *Bacillus tianshenii* |
| 894 | 10B_2 | 2012 | Transect_10B_2 | PV272596 | *Bacillus licheniformis* |
| 895 | 10B_3 | 2012 | Transect_10B_3 | PV272597 | *Bacillus licheniformis* |
| 896 | 10B_4 | 2012 | Transect_10B_4 | PV272598 | *Brevibacillus formosus* |
| 897 | 10B_5 | 2012 | Transect_10B_5 | MG897082 | *Oceanobacillus picturae* |
| 898 | 10B_6 | 2012 | Transect_10B_6 | MG897104 | *Paenibacillus tezpurensis* |
| 899 | 10B_7_1 | 2012 | Transect_10B_7_1 | MG897080 | *Brevibacillus formosus* |
| 900 | 10B_7_2 | 2012 | Transect_10B_7_2 | MG897081 | *Brevibacillus formosus* |
| 901 | 10C_1 | 2012 | Transect_10C_1 | PV272599 | *Agrococcus sediminis* |
| 902 | 10C_10 | 2012 | Transect_10C_10 | PV272600 | *Bacillus spizizenii* |
| 903 | 10C_11 | 2012 | Transect_10C_11 | MG897073 | *Kocuria polaris* |
| 904 | 10C_13 | 2012 | Transect_10C_13 | MG897072 | *Paracoccus zeaxanthinifaciens* |
| 905 | 10C_14 | 2012 | Transect_10C_14 | MG897071 | *Paracoccus zeaxanthinifaciens* |
| 906 | 10C_15 | 2012 | Transect_10C_15 | MG897070 | *Pontibacter virosus* |
| 907 | 10C_16 | 2012 | Transect_10C_16 | MG897069 | *Microvirga soli* |
| 908 | 10C_2 | 2012 | Transect_10C_2 | PV272601 | *Bacillus spizizenii* |
| 909 | 10C_3 | 2012 | Transect_10C_3 | PV272602 | *Bacillus coreaensis* |
| 910 | 10C_4 | 2012 | Transect_10C_4 | PV272603 | *Bacillus spizizenii* |
| 911 | 10C_6 | 2012 | Transect_10C_6 | PV272604 | *Bacillus spizizenii* |
| 912 | 10C_9 | 2012 | Transect_10C_9 | PV272605 | *Bacillus spizizenii* |
| 913 | 1A_1 | 2012 | Transect_1A_1 | PV272606 | *Metabacillus sediminilitoris* |
| 914 | 1A_10 | 2012 | Transect_1A_10 | MG897112 | *Brevibacillus formosus* |
| 915 | 1A_11 | 2012 | Transect_1A_11 | MG897115 | *Planococcus antarcticus* |
| 916 | 1A_12 | 2012 | Transect_1A_12 | MG897113 | *Kocuria polaris* |
| 917 | 1A_13 | 2012 | Transect_1A_13 | MG897108 | *Metabacillus bambusae* |
| 918 | 1A_14 | 2012 | Transect_1A_14 | MG897110 | *Brevibacillus formosus* |
| 919 | 1A_15 | 2012 | Transect_1A_15 | MG897111 | *Cytobacillus oceanisediminis* |
| 920 | 1A_2 | 2012 | Transect_1A_2 | PV272607 | *Bacillus licheniformis* |
| 921 | 1A_3 | 2012 | Transect_1A_3 | MG897121 | *Peribacillus simplex* |
| 922 | 1A_4 | 2012 | Transect_1A_4 | PV272608 | *Bacillus licheniformis* |
| 923 | 1A_7 | 2012 | Transect_1A_7 | PV272609 | *Bacillus zanthoxyli* |
| 924 | 1A_8 | 2012 | Transect_1A_8 | PV272610 | *Bacillus licheniformis* |
| 925 | 1A_9 | 2012 | Transect_1A_9 | PV272611 | *Bacillus spizizenii* |
| 926 | 1B_1 | 2012 | Transect_1B_1 | PV272612 | *Domibacillus iocasae* |
| 927 | 1B_10 | 2012 | Transect_1B_10 | MG897091 | *Brevibacillus formosus* |
| 928 | 1B_11 | 2012 | Transect_1B_11 | MG897087 | *Cytobacillus firmus* |
| 929 | 1B_12 | 2012 | Transect_1B_12 | MG897088 | *Cytobacillus firmus* |
| 930 | 1B_14 | 2012 | Transect_1B_14 | MG897094 | *Kocuria salina* |
| 931 | 1B_15 | 2012 | Transect_1B_15 | MG897093 | *Bacillus licheniformis* |
| 932 | 1B_2 | 2012 | Transect_1B_2 | PV272613 | *Priestia aryabhattai B8W22* |
| 933 | 1B_3 | 2012 | Transect_1B_3 | PV272614 | *Cytobacillus firmus* |
| 934 | 1B_4 | 2012 | Transect_1B_4 | PV272615 | *Bacillus licheniformis* |
| 935 | 1B_5 | 2012 | Transect_1B_5 | PV272616 | *Kocuria rosea* |
| 936 | 1B_7 | 2012 | Transect_1B_7 | PV272617 | *Agrococcus jenensis* |
| 937 | 1B_8 | 2012 | Transect_1B_8 | PV272618 | *Brevibacillus formosus* |
| 938 | 1B_9 | 2012 | Transect_1B_9 | MG897095 | *Agrococcus lahaulensis* |
| 939 | 2A_1 | 2012 | Transect_2A_1 | MG897118 | *Brevibacillus formosus* |
| 940 | 2A_10 | 2012 | Transect_2A_10 | MG897109 | *Bacillus licheniformis* |
| 941 | 2A_11 | 2012 | Transect_2A_11 | MG897084 | *Bacillus licheniformis* |
| 942 | 2A_2 | 2012 | Transect_2A_2 | MG897120 | *Bacillus licheniformis* |
| 943 | 2A_3 | 2012 | Transect_2A_3 | PV271959 | *Metabacillus crassostreae* |
| 944 | 2A_5 | 2012 | Transect_2A_5 | MG897107 | *Sutcliffiella deserti* |
| 945 | 2A_7 | 2012 | Transect_2A_7 | MG897086 | *Marinococcus tarijensis* |
| 946 | 2A_9 | 2012 | Transect_2A_9 | MG897106 | *Brevibacillus formosus* |
| 947 | 2B_1 | 2012 | Transect_2B_1 | PV271960 | *Metabacillus niabensis* |
| 948 | 2B_10 | 2012 | Transect_2B_10 | MG897077 | *Rhizobium cellulosilyticum* |
| 949 | 2B_11 | 2012 | Transect_2B_11 | MG897085 | *Devosia submarina* |
| 950 | 2B_12 | 2012 | Transect_2B_12 | MG897079 | *Brevibacillus formosus* |
| 951 | 2B_13 | 2012 | Transect_2B_13 | MG897078 | *Kocuria rosea* |
| 952 | 2B_2 | 2012 | Transect_2B_2 | PV271961 | *Halomonas saudii* |
| 953 | 2B_3 | 2012 | Transect_2B_3 | PV271962 | *Metabacillus litoralis* |
| 954 | 2B_4 | 2012 | Transect_2B_4 | PV271963 | *Peribacillus frigoritolerans* |
| 955 | 2B_5 | 2012 | Transect_2B_5 | PV271964 | *Bacillus cabrialesii* |
| 956 | 2B_6 | 2012 | Transect_2B_6 | PV271965 | *Bacillus licheniformis* |
| 957 | 2B_7 | 2012 | Transect_2B_7 | PV271966 | *Bacillus inaquosorum* |
| 958 | 2B_8 | 2012 | Transect_2B_8 | PV271967 | *Bacillus inaquosorum* |
| 959 | 2B_9 | 2012 | Transect_2B_9 | PV271968 | *Kocuria polaris* |
| 960 | 5C_1 | 2012 | Transect_5C_1 | MG897124 | *Planomicrobium okeanokoites* |
| 961 | 5C_11 | 2012 | Transect_5C_11 | PV271969 | *Bacillus inaquosorum* |
| 962 | 5C_12 | 2012 | Transect_5C_12 | MG897097 | *Arthrobacter subterraneus* |
| 963 | 5C_13 | 2012 | Transect_5C_13 | MG897098 | *Streptomyces lavendofoliae* |
| 964 | 5C_2 | 2012 | Transect_5C_2 | PV271970 | *Bacillus cabrialesii* |
| 965 | 5C_3 | 2012 | Transect_5C_3 | PV271971 | *Paracoccus speluncae* |
| 966 | 5C_4 | 2012 | Transect_5C_4 | PV271972 | *Arthrobacter bussei* |
| 967 | 5C_5 | 2012 | Transect_5C_5 | PV271973 | *Kocuria turfanensis* |
| 968 | 5C_7 | 2012 | Transect_5C_7 | PV271974 | *Bacillus licheniformis* |
| 969 | 5C_7_1 | 2012 | Transect_5C_7_1 | PV271975 | *Metabacillus niabensis* |
| 970 | 5D_1 | 2012 | Transect_5D_1 | PV271976 | *Bacillus spizizenii* |
| 971 | 5D_10 | 2012 | Transect_5D_10 | PV271977 | *Bacillus inaquosorum* |
| 972 | 5D_12 | 2012 | Transect_5D_12 | PV271978 | *Bacillus inaquosorum* |
| 973 | 5D_13 | 2012 | Transect_5D_13 | PV271979 | *Bacillus inaquosorum* |
| 974 | 5D_14 | 2012 | Transect_5D_14 | PV271980 | *Peribacillus frigoritolerans* |
| 975 | 5D_17 | 2012 | Transect_5D_17 | PV271981 | *Saccharothrix saharensis* |
| 976 | 5D_18 | 2012 | Transect_5D_18 | PV271982 | *Bacillus inaquosorum* |
| 977 | 5D_19 | 2012 | Transect_5D_19 | PV271983 | *Bacillus inaquosorum* |
| 978 | 5D_20 | 2012 | Transect_5D_20 | MG897075 | *Paenarthrobacter nitroguajacolicus* |
| 979 | 5D_3 | 2012 | Transect_5D_3 | PV271984 | *Bacillus cabrialesii* |
| 980 | 5D_4 | 2012 | Transect_5D_4 | PV271985 | *Bacillus inaquosorum* |
| 981 | 5D_5 | 2012 | Transect_5D_5 | PV271986 | *Bacillus inaquosorum* |
| 982 | 5D_6 | 2012 | Transect_5D_6 | PV271987 | *Bacillus licheniformis* |
| 983 | 5D_8 | 2012 | Transect_5D_8 | PV271988 | *Kocuria turfanensis* |
| 984 | 5D_8_1 | 2012 | Transect_5D_8_1 | PV271989 | *Cellulomonas shaoxiangyii* |
| 985 | 5D_9 | 2012 | Transect_5D_9 | PV271990 | *Streptomyces cinereoruber* |
| 986 | 6A_1 | 2012 | Transect_6A_1 | PV271991 | *Arthrobacter pokkalii* |
| 987 | 6A_2 | 2012 | Transect_6A_2 | PV271992 | *Pseudarthrobacter equi* |
| 988 | 6A_3 | 2012 | Transect_6A_3 | PV271993 | *Bacillus paramycoides* |
| 989 | 6A_5 | 2012 | Transect_6A_5 | PV271994 | *Metabacillus niabensis* |
| 990 | 6C_11 | 2012 | Transect_6C_11 | PV271995 | *Peribacillus frigoritolerans* |
| 991 | 6C_13 | 2012 | Transect_6C_13 | MG897083 | *Agrococcus jenensis* |
| 992 | 6C_14 | 2012 | Transect_6C_14 | MG897102 | *Kocuria polaris* |
| 993 | 6C_15 | 2012 | Transect_6C_15 | MG897101 | *Kocuria sediminis* |
| 994 | 6C_6 | 2012 | Transect_6C_6 | PV271996 | *Kocuria sediminis* |
| 995 | 6C_7 | 2012 | Transect_6C_7 | PV271997 | *Devosia aurantiaca* |
| 996 | 6C_9 | 2012 | Transect_6C_9 | PV271998 | *Peribacillus frigoritolerans* |
| 997 | 6D_1 | 2012 | Transect_6D_1 | PV271999 | *Aeromonas veronii* |
| 998 | 6D_10 | 2012 | Transect_6D_10 | PV272000 | *Kocuria polaris* |
| 999 | 6D_11 | 2012 | Transect_6D_11 | MG897064 | *Cellulosimicrobium cellulans* |
| 1000 | 6D_12_1 | 2012 | Transect_6D_12_1 | MG897062 | *Cellulosimicrobium aquatile* |
| 1001 | 6D_12_2 | 2012 | Transect_6D_12_2 | MG897063 | *Cellulosimicrobium cellulans* |
| 1002 | 6D_13 | 2012 | Transect_6D_13 | MG897114 | *Brevibacillus formosus* |
| 1003 | 6D_2 | 2012 | Transect_6D_2 | PV272001 | *Pseudarthrobacter phenanthrenivorans* |
| 1004 | 6D_4 | 2012 | Transect_6D_4 | PV272003 | *Roseomonas aestuarii* |
| 1005 | 6D_5 | 2012 | Transect_6D_5 | PV272004 | *Bacillus inaquosorum* |
| 1006 | 6D_6 | 2012 | Transect_6D_6 | MG897116 | *Paenarthrobacter nitroguajacolicus* |
| 1007 | 6D_7 | 2012 | Transect_6D_7 | PV272005 | *Bacillus inaquosorum* |
| 1008 | 6D_8 | 2012 | Transect_6D_8 | PV272006 | *Cellulosimicrobium cellulans* |
| 1009 | 6D_9 | 2012 | Transect_6D_9 | PV272007 | *Bacillus licheniformis* |
| 1010 | 9A_1 | 2012 | Transect_9A_1 | PV272008 | *Bacillus licheniformis* |
| 1011 | 9A_10 | 2012 | Transect_9A_10 | PV272009 | *Bacillus licheniformis* |
| 1012 | 9A_11 | 2012 | Transect_9A_11 | PV272010 | *Metabacillus niabensis* |
| 1013 | 9A_12 | 2012 | Transect_9A_12 | PV272011 | *Metabacillus niabensis* |
| 1014 | 9A_14 | 2012 | Transect_9A_14 | MG897089 | *Sutcliffiella deserti* |
| 1015 | 9A_2 | 2012 | Transect_9A_2 | PV272012 | *Bacillus sonorensis* |
| 1016 | 9A_3 | 2012 | Transect_9A_3 | PV272013 | *Metabacillus niabensis* |
| 1017 | 9A_4 | 2012 | Transect_9A_4 | PV272014 | *Metabacillus niabensis* |
| 1018 | 9A_5 | 2012 | Transect_9A_5 | PV272015 | *Bacillus aequororis* |
| 1019 | 9A_6 | 2012 | Transect_9A_6 | PV272016 | *Bacillus licheniformis* |
| 1020 | 9A_7 | 2012 | Transect_9A_7 | PV272017 | *Microvirga arabica* |
| 1021 | 9A_8 | 2012 | Transect_9A_8 | PV272018 | *Bacillus licheniformis* |
| 1022 | 9A_9 | 2012 | Transect_9A_9 | MG897076 | *Bacillus licheniformis* |
| 1023 | 9B_10 | 2012 | Transect_9B_10 | MG897065 | *Arthrobacter sedimenti* |
| 1024 | 9B_11 | 2012 | Transect_9B_11 | MG897060 | *Metabacillus schmidteae* |
| 1025 | 9B_12_1 | 2012 | Transect_9B_12_1 | MG897066 | *Brevibacillus formosus* |
| 1026 | 9B_12_2 | 2012 | Transect_9B_12_2 | MG897067 | *Brevibacillus formosus* |
| 1027 | 9B_2 | 2012 | Transect_9B_2 | PV272019 | *Planococcus halotolerans* |
| 1028 | 9B_3 | 2012 | Transect_9B_3 | PV272020 | *Bacillus licheniformis* |
| 1029 | 9B_4 | 2012 | Transect_9B_4 | PV272021 | *Metabacillus niabensis* |
| 1030 | 9B_5 | 2012 | Transect_9B_5 | PV272022 | *Paenibacillus popilliae* |
| 1031 | 9B_6 | 2012 | Transect_9B_6 | PV272023 | *Peribacillus frigoritolerans* |
| 1032 | 9B_7 | 2012 | Transect_9B_7 | PV272024 | *Methylorubrum extorquens* |
| 1033 | 9B_8 | 2012 | Transect_9B_8 | PV272025 | *Kocuria rosea* |
| 1034 | 9B_9 | 2012 | Transect_9B_9 | MG897061 | *Kocuria rosea* |
| 1035 | 9C_10 | 2012 | Transect_9C_10 | PV272026 | *Bacillus spizizenii* |
| 1036 | 9C_12 | 2012 | Transect_9C_12 | PV272027 | *Metabacillus crassostreae* |
| 1037 | 9C_13 | 2012 | Transect_9C_13 | PV272028 | *Kocuria polaris* |
| 1038 | 9C_14 | 2012 | Transect_9C_14 | MG897100 | *Cesiribacter roseus* |
| 1039 | 9C_15 | 2012 | Transect_9C_15 | MG897099 | *Methylorubrum extorquens* |
| 1040 | 9C_2 | 2012 | Transect_9C_2 | PV272029 | *Metabacillus niabensis* |
| 1041 | 9C_3 | 2012 | Transect_9C_3 | PV272030 | *Bacillus licheniformis* |
| 1042 | 9C_4 | 2012 | Transect_9C_4 | MG897122 | *Bacillus sonorensis* |
| 1043 | 9C_5 | 2012 | Transect_9C_5 | PV272031 | *Bacillus spizizenii* |
| 1044 | 9C_5_1 | 2012 | Transect_9C_5_1 | PV272032 | *Bacillus stercoris* |
| 1045 | 9C_6 | 2012 | Transect_9C_6 | PV272033 | *Bacillus spizizenii* |
| 1046 | 9C_8 | 2012 | Transect_9C_8 | PV272034 | *Bacillus inaquosorum* |
| 1047 | 9C_9 | 2012 | Transect_9C_9 | PV272035 | *Bacillus inaquosorum* |
| 1048 | 9D_1 | 2012 | Transect_9D_1 | PV272036 | *Bacillus licheniformis* |
| 1049 | 9D_2 | 2012 | Transect_9D_2 | PV272037 | *Bacillus spizizenii* |
| 1050 | 9D_3 | 2012 | Transect_9D_3 | PV272038 | *Bacillus spizizenii* |
| 1051 | 9D_4 | 2012 | Transect_9D_4 | PV272039 | *Bacillus spizizenii* |
| 1052 | 9D_6 | 2012 | Transect_9D_6 | PV272040 | *Paenibacillus thiaminolyticus* |
| 1053 | 24_1.1_p1775 | 2014 | LI 7 | MG897041 | *Hoeflea olei* |
| 1054 | 25_1.2_p1775 | 2014 | LI 7 | MG897054 | *Rheinheimera muenzenbergensis* |
| 1055 | 10_2.1_p1775 | 2014 | LI 7 | MG897032 | *Brevundimonas bacteroides* |
| 1056 | 26_2.3_p1775 | 2014 | LI 7 | MG897056 | *Phenylobacterium koreense* |
| 1057 | 11_2.5_p1775 | 2014 | LI 7 | MG897036 | *Brevundimonas bacteroides* |
| 1058 | 6_4.1_p1775 | 2014 | LI 7 | MG897052 | *Peteryoungia ipomoeae* |
| 1059 | 13_4.3_p1775 | 2014 | LI 7 | MG897038 | *Pararheinheimera chironomi* |
| 1060 | 22_4.5_p1775 | 2014 | LI 7 | MG897043 | *Peteryoungia ipomoeae* |
| 1061 | 23_5.4_p1775 | 2014 | LI 7 | PV272041 | *Pseudomonas peli* |
| 1062 | 7_6.2_p1775 | 2014 | LI 7 | MG897055 | *Peteryoungia ipomoeae* |
| 1063 | 78_8.3_p1775 | 2014 | LI 7 | MG897047 | *Bacillus licheniformis* |
| 1064 | 12_9.2_p1775 | 2014 | LI 7 | MG897037 | *Brevibacillus reuszeri* |
| 1065 | 4_H1.1_p1775 | 2014 | LI 7 | PV272042 | *Metabacillus flavus* |
| 1066 | 8_H1.1_p1775 | 2014 | LI 7 | MG897031 | *Metabacillus flavus* |
| 1067 | 1_H1.3_p1775 | 2014 | LI 7 | MG897023 | *Metabacillus litoralis* |
| 1068 | 3_H1.4_p1775 | 2014 | LI 7 | MG897058 | *Bacillus licheniformis* |
| 1069 | 2_H1.5_p1775 | 2014 | LI 7 | MG897022 | *Bacillus mesophilum* |
| 1070 | 76_H1.6_p1775 | 2014 | LI 7 | MG897018 | *Brevibacillus formosus* |
| 1071 | 82_H1.7_p1775 | 2014 | LI 7 | MG897017 | *Bacillus tequilensis* |
| 1072 | 16_H1.8_p1775 | 2014 | LI 7 | MG897048 | *Brevibacillus formosus* |
| 1073 | 14_H10.2_p1775 | 2014 | LI 7 | MG897045 | *Bacillus aequororis* |
| 1074 | 87_H10.2_p1775 | 2014 | LI 7 | MG897035 | *Bacillus licheniformis* |
| 1075 | 5_H2.3_p1775 | 2014 | LI 7 | MG897050 | *Bacillus pumilus* |
| 1076 | 75_H2.4_p1775 | 2014 | LI 7 | MG897033 | *Rossellomorea vietnamensis* |
| 1077 | 90_H2.6_p1775 | 2014 | LI 7 | MG897026 | *Rossellomorea vietnamensis* |
| 1078 | 18_H3.3_p1775 | 2014 | LI 7 | MG897039 | *Metabacillus crassostreae* |
| 1079 | 17_H3.6_p1775 | 2014 | LI 7 | MG897040 | *Bacillus paramycoides* |
| 1080 | 86_H4.1_p1775 | 2014 | LI 7 | MG897034 | *Bacillus licheniformis* |
| 1081 | 81_H4.3_p1775 | 2014 | LI 7 | MG897016 | *Metabacillus flavus* |
| 1082 | 89_H4.6_p1775 | 2014 | LI 7 | MG897029 | *Bacillus thuringiensis* |
| 1083 | 80_H5.3_p1775 | 2014 | LI 7 | MG897020 | *Bacillus licheniformis* |
| 1084 | 93_H5.4_p1775 | 2014 | LI 7 | MG897057 | *Brevibacillus formosus* |
| 1085 | 19_H5.6_p1775 | 2014 | LI 7 | MG897021 | *Bacillus mesophilum* |
| 1086 | 95_H8.1_p1775 | 2014 | LI 7 | MG897053 | *Paenisporosarcina indica* |
| 1087 | 85_H8.2_p1775 | 2014 | LI 7 | MG897019 | *Bacillus aequororis* |
| 1088 | 84_H8.4_p1775 | 2014 | LI 7 | MG897025 | *Bacillus inaquosorum* |
| 1089 | 77_H8.6_p1775 | 2014 | LI 7 | MG897046 | *Peteryoungia ipomoeae* |
| 1090 | 15_H9.1_p1775 | 2014 | LI 7 | MG897044 | *Metabacillus crassostreae* |
| 1091 | 94_H9.2_p1775 | 2014 | LI 7 | MG897051 | *Metabacillus flavus* |
| 1092 | 83_H9.4_p1775 | 2014 | LI 7 | MG897024 | *Bacillus mesophilum* |
| 1093 | 21_H9.5_p1775 | 2014 | LI 7 | MG897042 | *Bacillus licheniformis* |
| 1094 | 79_H9.6_p1775 | 2014 | LI 7 | MG897049 | *Bacillus paramycoides* |
| 1095 | 10_2019_3_E1_outside_soil_dir | 2019 | In | PV272043 | *Pontibacter salisaro* |
| 1096 | 10_2019_5_E1_outside_soil_dir | 2019 | In | PV272044 | *Planococcus chinensis* |
| 1097 | 10_2019_7_E1_outside_soil_dir | 2019 | In | PV272045 | *Priestia megaterium* |
| 1098 | 10_2019_8_E1_outside_soil_dir | 2019 | In | PV272046 | *Bacillus licheniformis* |
| 1099 | 10_2019_9_E1_soil_dir | 2019 | In | PV272047 | *Rossellomorea vietnamensis* |
| 1100 | 10_2019_11_E1_inside_soil_dir | 2019 | In | PV272048 | *Rossellomorea marisflavi* |
| 1101 | 10_2019_12_E1_outside_soil_dir | 2019 | In | PV272049 | *Bacillus halotolerans* |
| 1102 | 10_2019_13_E1_outside_soil_dir | 2019 | In | PV272050 | *Bacillus vallismortis* |
| 1103 | 10_2019_14_E1+agua_outside_soil_dir | 2019 | In | PV272051 | *Bacillus licheniformis* |
| 1104 | 10_2019_15_E1-agua_soil_dir | 2019 | In | PV272052 | *Bacillus halotolerans* |
| 1105 | 10_2019_16_E1+agua_soil_dir | 2019 | In | PV272053 | *Bacillus licheniformis* |
| 1106 | 10_2019_17_E1+agua_soil_dir | 2019 | In | PV272054 | *Rossellomorea aquimaris* |
| 1107 | 10_2019_18_E1+agua_soil_dir | 2019 | In | PV272055 | *Bacillus proteolyticus* |
| 1108 | 10_2019_19_E1+agua_soil_dir | 2019 | In | PV272056 | *Bacillus halotolerans* |
| 1109 | 10_2019_20_E1+agua_soil_dir | 2019 | In | PQ578922 | *Metabacillus indicus* |
| 1110 | 10_2019_22_E1+agua_soil_hot | 2019 | In | PV272057 | *Bacillus vallismortis* |
| 1111 | 10_2019_24_E1+agua_soil_dir | 2019 | In | PV272058 | *Kocuria himachalensis* |
| 1112 | 10_2019_25_E1+agua_soil_dir | 2019 | In | PV272059 | *Bacillus halotolerans* |
| 1113 | 10_2019_26_E1+agua_soil_dir | 2019 | In | PV272060 | *Bacillus licheniformis* |
| 1114 | 10_2019_27_E1+agua_soil_dir | 2019 | In | PV272061 | *Rossellomorea vietnamensis* |
| 1115 | 10_2019_28_E1+agua_soil_dir | 2019 | In | PV272062 | *Staphylococcus epidermidis* |
| 1116 | 10_2019_29_E1+agua_outside_soil_dir | 2019 | In | PV272063 | *Micrococcus luteus* |
| 1117 | 10_2019_30_E1+agua_soil_dir | 2019 | In | PV272064 | *Bacillus stercoris* |
| 1118 | 10_2019_31_2_soil_dir | 2019 | LI2 | PV272065 | *Pannonibacter carbonis* |
| 1119 | 10_2019_32_2_soil_dir | 2019 | LI2 | PV272066 | *Bacillus sonorensis* |
| 1120 | 10_2019_33_2_soil_dir | 2019 | LI2 | PV272067 | *Bacillus paramycoides* |
| 1121 | 10_2019_35_2_soil_dir | 2019 | LI2 | PV272068 | *Bacillus licheniformis* |
| 1122 | 10_2019_38_2_soil_dir | 2019 | LI2 | PV272069 | *Bacillus halotolerans* |
| 1123 | 10_2019_40_2_soil_dir | 2019 | LI2 | PV272070 | *Bacillus pakistanensis* |
| 1124 | 10_2019_42_manantial2_soil_dir | 2019 | Manantial2 | PV272071 | *Planomicrobium okeanokoites* |
| 1125 | 10_2019_43_manantial3_soil_dir | 2019 | Manantial3 | PV272072 | *Micrococcus luteus* |
| 1126 | 10_2019_44_manantial4_soil_dir | 2019 | Manantial4 | PV272073 | *Mesobacillus jeotgali* |
| 1127 | 10_2019_45_manantial5_soil_dir | 2019 | Manantial5 | PV272074 | *Bacillus stercoris* |
| 1128 | 10_2019_46_manantial6_soil_dir | 2019 | Manantial6 | PV272075 | *Bacillus vallismortis* |
| 1129 | 10_2019_47_manantial7_soil_dir | 2019 | Manantial7 | PV272076 | *Bacillus licheniformis* |
| 1130 | 10_2019_48_manantial8_soil_dir | 2019 | Manantial8 | PV272077 | *Pseudothioclava arenosa* |
| 1131 | 10_2019_49_manantial9_soil_dir | 2019 | Manantial9 | PV272078 | *Devosia albogilva* |
| 1132 | 10_2019_50_manantial10_soil_dir | 2019 | Manantial10 | PV272079 | *Bacillus vallismortis* |
| 1133 | 10_2019_52_manantial12_soil_dir | 2019 | Manantial12 | PV272080 | *Bacillus zhangzhouensis* |
| 1134 | 10_2019_53_manantial13_soil_dir | 2019 | Manantial13 | PV272081 | *Gordonia terrae* |
| 1135 | 10_2019_54_manantial14_soil_dir | 2019 | Manantial14 | PV272082 | *Pseudarthrobacter oxydans* |
| 1136 | 10_2019_57_manantial17_soil_dir | 2019 | Manantial17 | PV272083 | *Bacillus proteolyticus* |
| 1137 | 10_2019_59_manantial19_soil_dir | 2019 | Manantial19 | PV272084 | *Gordonia hongkongensis* |
| 1138 | 10_2019_61_4_soil_dir | 2019 | LI4 | PV272085 | *Staphylococcus epidermidis* |
| 1139 | 10_2019_62_4_soil_dir | 2019 | LI4 | PV272086 | *Bacillus proteolyticus* |
| 1140 | 10_2019_64_4_soil_dir | 2019 | LI4 | PV272087 | *Staphylococcus epidermidis* |
| 1141 | 10_2019_65_4_soil_dir | 2019 | LI4 | PQ578923 | *Bacillus proteolyticus* |
| 1142 | 10_2019_66_4_soil_dir | 2019 | LI4 | PV272088 | *Paracoccus speluncae* |
| 1143 | 10_2019_67_4_soil_dir | 2019 | LI4 | PV272089 | *Bacillus halotolerans* |
| 1144 | 10_2019_68_5_soil_dir | 2019 | LI5 | PV272090 | *Erythrobacter dokdonensis* |
| 1145 | 10_2019_71_5_soil_dir | 2019 | LI5 | PV272091 | *Pannonibacter carbonis* |
| 1146 | 10_2019_73_5_soil_dir | 2019 | LI5 | PV272092 | *Oceanibaculum indicum P24* |
| 1147 | 10_2019_74_5_soil_dir | 2019 | LI5 | PV272093 | *Rossellomorea vietnamensis* |
| 1148 | 10_2019_77_5_soil_dir | 2019 | LI5 | PV272094 | *Bacillus vallismortis* |
| 1149 | 10_2019_78_5_soil_dir | 2019 | LI5 | PV272095 | *Kocuria sediminis* |
| 1150 | 10_2019_79_5_soil_dir | 2019 | LI5 | PV272096 | *Sutcliffiella zhanjiangensis* |
| 1151 | 10_2019_80_5_soil_dir | 2019 | LI5 | PV272097 | *Metabacillus litoralis* |
| 1152 | 10_2019_81_5_soil_dir | 2019 | LI5 | PV272098 | *Agrococcus lahaulensis* |
| 1153 | 10_2019_82_5_soil_dir | 2019 | LI5 | PV272099 | *Roseivivax halodurans* |
| 1154 | 10_2019_84_5_soil_dir | 2019 | LI5 | PV272100 | *Staphylococcus epidermidis* |
| 1155 | 10_2019_85_5_soil_dir | 2019 | LI5 | PV272101 | *Sutcliffiella deserti* |
| 1156 | 10_2019_87_6_soil_dir | 2019 | LI6 | PV272102 | *Staphylococcus epidermidis* |
| 1157 | 10_2019_88_6_soil_dir | 2019 | LI6 | PV272103 | *Bacillus licheniformis* |
| 1158 | 10_2019_89_6_soil_dir | 2019 | LI6 | PV272104 | *Bacillus proteolyticus* |
| 1159 | 10_2019_90_6_soil_dir | 2019 | LI6 | PV272105 | *Bacillus proteolyticus* |
| 1160 | 10_2019_91_6_soil_dir | 2019 | LI6 | PV272106 | *Bacillus aequororis* |
| 1161 | 10_2019_93_6_soil_dir | 2019 | LI6 | PV272107 | *Agrococcus lahaulensis* |
| 1162 | 10_2019_94_6_soil_dir | 2019 | LI6 | PV272108 | *Rossellomorea vietnamensis* |
| 1163 | 10_2019_95_6_soil_dir | 2019 | LI6 | PV272109 | *Pseudarthrobacter polychromogenes* |
| 1164 | 10_2019_98_6.5_soil_dir | 2019 | LI6 | PQ578924 | *Marinobacter orientalis* |
| 1165 | 10_2019_99_6.5_soil_dir | 2019 | LI6 | PV272110 | *Paracoccus aerius* |
| 1166 | 10_2019_100_6.5_soil_dir | 2019 | LI6 | PV272111 | *Staphylococcus epidermidis* |
| 1167 | 10_2019_102_6.5_soil_dir | 2019 | LI6 | PV272112 | *Bacillus proteolyticus* |
| 1168 | 10_2019_104_6.5_soil_dir | 2019 | LI6 | PV272113 | *Paracoccus aerius* |
| 1169 | 10_2019_105_7_soil_dir | 2019 | LI7 | PV272114 | *Bacillus paramycoides* |
| 1170 | 10_2019_106_7_soil_dir | 2019 | LI7 | PV272115 | *Bacillus proteolyticus* |
| 1171 | 10_2019_107_7_soil_hot | 2019 | LI7 | PV272116 | *Bacillus sonorensis* |
| 1172 | 10_2019_109_7_soil_dir | 2019 | LI7 | PV272117 | *Bacillus proteolyticus* |
| 1173 | 10_2019_110_7_soil_dir | 2019 | LI7 | PV272118 | *Bacillus halotolerans* |
| 1174 | 10_2019_111_7_soil_dir | 2019 | LI7 | PV272119 | *Bacillus sonorensis* |
| 1175 | 10_2019_113_7.5_soil_dir | 2019 | LI7 | PV272120 | *Bacillus aequororis* |
| 1176 | 10_2019_114_7.5_soil_dir | 2019 | LI7 | PV272121 | *Bacillus halotolerans* |
| 1177 | 10_2019_115_7.5_soil_dir | 2019 | LI7 | PV272122 | *Bacillus paramycoides* |
| 1178 | 10_2019_116_7.5_soil_dir | 2019 | LI7 | PV272123 | *Bacillus aequororis* |
| 1179 | 10_2019_118_7.5_soil_dir | 2019 | LI7 | PV272124 | *Bacillus subtilis* |
| 1180 | 10_2019_123_8_soil_dir | 2019 | LI8 | PV272125 | *Terribacillus goriensis* |
| 1181 | 10_2019_124_8_soil_dir | 2019 | LI8 | PV272126 | *Erythrobacter dokdonensis* |
| 1182 | 10_2019_126_8_soil_dir | 2019 | LI8 | PV272127 | *Bacillus halotolerans* |
| 1183 | 10_2019_127_8_soil_dir | 2019 | LI8 | PV272128 | *Aquimonas voraii* |
| 1184 | 10_2019_129_8_soil_dir | 2019 | LI8 | PV272129 | *Jeotgalibacillus alimentarius* |
| 1185 | 10_2019_130_8_soil_dir | 2019 | LI8 | PV272130 | *Pannonibacter carbonis* |
| 1186 | 10_2019_131_8_soil_dir | 2019 | LI8 | PV272131 | *Bacillus safensis* |
| 1187 | 10_2019_132_8_soil_dir | 2019 | LI8 | PV272132 | *Priestia aryabhattai B8W22* |
| 1188 | 10_2019_134_manantial2_soil_dir | 2019 | Manantial2 | PV272133 | *Pseudomonas ceruminis* |
| 1189 | 10_2019_135_manantial3_soil_dir | 2019 | Manantial3 | PV272134 | *Mumia flava* |
| 1190 | 10_2019_136_manantial4_soil_dir | 2019 | Manantial4 | PV272135 | *Bacillus zanthoxyli* |
| 1191 | 10_2019_137_manantial5_soil_dir | 2019 | Manantial5 | PV272136 | *Bacillus licheniformis* |
| 1192 | 10_2019_138_manantial6_soil_dir | 2019 | Manantial6 | PV272137 | *Bacillus licheniformis* |
| 1193 | 10_2019_139_manantial7_soil_dir | 2019 | Manantial7 | PV272138 | *Bacillus licheniformis* |
| 1194 | 10_2019_140_manantial8_soil_dir | 2019 | Manantial8 | PV272139 | *Bacillus licheniformis* |
| 1195 | 10_2019_142_manantiarl2_soil_dir | 2019 | Manantial2 | PV272140 | *Bacillus licheniformis* |
| 1196 | 10_2019_143_manantiarl3_soil_dir | 2019 | Manantial3 | PV272141 | *Jeotgalibacillus marinus* |
| 1197 | 10_2019_144_manantiarl4_soil_dir | 2019 | Manantial4 | PV272142 | *Priestia aryabhattai B8W22* |
| 1198 | 10_2019_145_manantial5_soil_dir | 2019 | Manantial5 | PV272143 | *Rossellomorea aquimaris* |
| 1199 | 10_2019_146_manantial6_soil_hot | 2019 | Manantial6 | PV272144 | *Bacillus proteolyticus* |
| 1200 | 10_2019_147_manantial7_soil_hot | 2019 | Manantial7 | PV272145 | *Aeromonas veronii* |
| 1201 | 10_2019_148_manantial8_soil_hot | 2019 | Manantial8 | PV272146 | *Rossellomorea vietnamensis* |
| 1202 | 10_2019_149_manantial9_soil_hot | 2019 | Manantial9 | PV272147 | *Aeromonas veronii* |
| 1203 | 10_2019_151_manantial11_soil_dir | 2019 | Manantial11 | PV272148 | *Metabacillus malikii* |
| 1204 | 10_2019_152_manantial12_soil_dir | 2019 | Manantial12 | PV272149 | *Bacillus proteolyticus* |
| 1205 | 10_2019_153_manantial13_soil_dir | 2019 | Manantial13 | PV272150 | *Aeromonas veronii* |
| 1206 | 10_2019_154_manantial14_soil_dir | 2019 | Manantial14 | PV272151 | *Bacillus proteolyticus* |
| 1207 | 10_2019_155_manantial15_soil_dir | 2019 | Manantial15 | PV272152 | *Bacillus subtilis* |
| 1208 | 10_2019_156_manantial16_soil_dir | 2019 | Manantial16 | PV272153 | *Aeromonas veronii* |
| 1209 | 10_2019_158_manantial18_soil_dir | 2019 | Manantial18 | PV272154 | *Bacillus pakistanensis* |
| 1210 | 10_2019_160_sitio3_soil_dir | 2019 | LI3 | PV272155 | *Qipengyuania algicida* |
| 1211 | 10_2019_162_sitio5_soil_dir | 2019 | LI5 | PV272156 | *Arthrobacter pascens* |
| 1212 | 10_2019_165_sitio3_soil_dir | 2019 | LI3 | PV272157 | *Aeromonas salmonicida* |
| 1213 | 10_2019_167_sitio3_soil_dir | 2019 | LI3 | PV272158 | *Salipiger manganoxidans* |
| 1214 | 10_2019_168_sitio7_soil_dir | 2019 | LI7 | PV272159 | *Bacillus vallismortis* |
| 1215 | 10_2019_169_sitio5_soil_dir | 2019 | LI5 | PQ578921 | *Pannonibacter carbonis* |
| 1216 | 10_2010_170_sitio5_soil_dir | 2019 | LI5 | PV272160 | *Aquimonas voraii* |
| 1217 | 10_2019_172_sitio5_soil_hot | 2019 | LI5 | PV272161 | *Bacillus aequororis* |
| 1218 | 10_2019_173_sitio5_soil_hot | 2019 | LI5 | PV272162 | *Aeromicrobium stalagmiti* |
| 1219 | 10_2019_174_sitio5_soil_hot | 2019 | LI5 | PV272163 | *Bacillus atrophaeus* |
| 1220 | 10_2019_176_sitio6_soil_dir | 2019 | LI6 | PV272164 | *Aeromonas veronii* |
| 1221 | 10_2019_177_sitio6_soil_dir | 2019 | LI6 | PV272165 | *Bacillus proteolyticus* |
| 1222 | 10_2019_178_sitio6_soil_dir | 2019 | LI6 | PV272166 | *Bacillus proteolyticus* |
| 1223 | 10_2019_179_sitio6_soil_dir | 2019 | LI6 | PV272167 | *Bacillus proteolyticus* |
| 1224 | 10_2019_180_sitio6_soil_dir | 2019 | LI6 | PV272168 | *Pannonibacter phragmitetus* |
| 1225 | 10_2019_181_sitio6_soil_hot | 2019 | LI6 | PV272169 | *Brevibacillus formosus* |
| 1226 | 10_2019_183_sitio6_soil_hot | 2019 | LI6 | PV272170 | *Bacillus licheniformis* |
| 1227 | 10_2019_184_sitio6_soil_dir | 2019 | LI6 | PV272171 | *Metabacillus sediminilitoris* |
| 1228 | 10_2019_185_sitio6_soil_dir | 2019 | LI6 | PV272172 | *Bacillus halotolerans* |
| 1229 | 10_2019_188_sitio6_soil_dir | 2019 | LI6 | PV272173 | *Staphylococcus epidermidis* |
| 1230 | 10_2019_189_sitio6_soil_dir | 2019 | LI6 | PV272174 | *Metabacillus crassostreae* |
| 1231 | 10_2019_190_sitio6_soil_dir | 2019 | LI6 | PV272175 | *Aeromicrobium stalagmiti* |
| 1232 | 10_2019_191_sitio6_soil_dir | 2019 | LI6 | PV272176 | *Bacillus licheniformis* |
| 1233 | 10_2019_192_sitio7_soil_hot | 2019 | LI7 | PV272177 | *Bacillus sonorensis* |
| 1234 | 10_2019_193_sitio7_soil_hot | 2019 | LI7 | PV272178 | *Brevibacillus agri* |
| 1235 | 10_2019_194_sitio7_soil_hot | 2019 | LI7 | PV272179 | *Rossellomorea vietnamensis* |
| 1236 | 10_2019_195_sitio7_soil_dir | 2019 | LI7 | PV272180 | *Rhizobium wuzhouense* |
| 1237 | 10_2019_197_sitio7_soil_dir | 2019 | LI7 | PV272181 | *Bacillus vallismortis* |
| 1238 | 10_2019_199_sitio7.5_soil_dir | 2019 | LI7 | PV272182 | *Bacillus vallismortis* |
| 1239 | 10_2019_200_sitio7.5_soil_dir | 2019 | LI7 | PV272183 | *Rhizobium rosettiformans W3* |
| 1240 | 10_2019_202_sitio7.5_soil_dir | 2019 | LI7 | PV272184 | *Bacillus stercoris* |
| 1241 | 10_2019_204_sitio7.5_soil_dir | 2019 | LI7 | PV272185 | *Rhizobium wuzhouense* |
| 1242 | 10_2019_205_sitio7.5_soil_hot | 2019 | LI7 | PV272186 | *Bacillus halotolerans* |
| 1243 | 10_2019_207_sitio7.5_soil_hot | 2019 | LI7 | PV272187 | *Rossellomorea vietnamensis* |
| 1244 | 10_2019_208_sitio7.5_soil_hot | 2019 | LI7 | PV272188 | *Rossellomorea vietnamensis* |
| 1245 | 10_2019_209_sitio7.5_soil_dir | 2019 | LI7 | PV272189 | *Bacillus stercoris* |
| 1246 | 10_2019_210_sitio8_soil_hot | 2019 | LI8 | PV272191 | *Bacillus halotolerans* |
| 1247 | 10_2019_212_sitio8_soil_hot | 2019 | LI8 | PV272192 | *Cytobacillus firmus* |
| 1248 | 10_2019_213_sitio8_soil_dir | 2019 | LI8 | PV272193 | *Pannonibacter indicus* |
| 1249 | 10_2019_214_sitio8_soil_dir | 2019 | LI8 | PV272194 | *Pannonibacter indicus* |
| 1250 | 10_2019_215_sitio8_soil_dir | 2019 | LI8 | PV272195 | *Bacillus licheniformis* |
| 1251 | 10_2019_217_sitio8_soil_dir | 2019 | LI8 | PV272196 | *Novosphingobium lindaniclasticum* |
| 1252 | 10_2019_222_sitio8_soil_dir | 2019 | LI8 | PV272197 | *Fictibacillus nanhaiensis* |
| 1253 | 10_2019_223_sitio8_soil_dir | 2019 | LI8 | PV272198 | *Bacillus aequororis* |
| 1254 | 10_2019_101_6.5_soil_dir | 2019 | LI6 | PV272199 | *Rossellomorea vietnamensis* |
| 1255 | 10_2019_101_2_6.5_soil_dir | 2019 | LI6 | PV272200 | *Roseomonas hellenica* |
| 1256 | 02_2012_9_s3_sed_dir_D08.ab1 | 2022 | s3 | PV272201 | *Pseudomonas leptonychotis* |
| 1257 | 02_2012_20_s2_sed_dir_B11.ab1 | 2022 | s2 | PV272202 | *Bacillus aequororis* |
| 1258 | 02_2012_35_e2_sed_dir_H09.ab1 | 2022 | e2 | PV272203 | *Rheinheimera lutimaris* |
| 1259 | 02_2012_39_e2_sed_dir_A08.ab1 | 2022 | e2 | PV272204 | *Tabrizicola sediminis* |
| 1260 | 02_2012_40_e1_sed_dir_H07.ab1 | 2022 | e1 | PV272205 | *Jeotgalibacillus marinus* |
| 1261 | 02_2012_44_e1_sed_dir_A09.ab1 | 2022 | e1 | PV272206 | *Arenimonas caeni* |
| 1262 | 02_2012_46_e1_sed_dir_C11.ab1 | 2022 | e1 | PV272207 | *Rossellomorea vietnamensis* |
| 1263 | 02_2012_79_l1_sed_dir_F09.ab1 | 2022 | Li1 | PV272216 | *Exiguobacterium aurantiacum* |
| 1264 | 02_2012_84_s1_sed_dir_D07.ab1 | 2022 | s1 | PV272217 | *Pararheinheimera chironomi* |
| 1265 | 1_mar_22 | 2022 | LI3 | PV272218 | *Bacillus halotolerans* |
| 1266 | 2_mar_22 | 2022 | LI3 | PV272219 | *Bacillus massiliglaciei* |
| 1267 | 6_mar_22 | 2022 | LI3 | PV272220 | *Bacillus zhangzhouensis* |
| 1268 | 8_mar_22 | 2022 | LI3 | PV272221 | *Arthrobacter pokkalii* |
| 1269 | 9_mar_22 | 2022 | LI3 | PV272222 | *Sutcliffiella zhanjiangensis* |
| 1270 | 10_mar_22_2 | 2022 | LI3 | PV272223 | *Sutcliffiella deserti* |
| 1271 | 10_mar_22_1 | 2022 | LI3 | PV272224 | *Bacillus tianshenii* |
| 1272 | 11_mar_22 | 2022 | LI3 | PV272225 | *Bacillus aequororis* |
| 1273 | 12_mar_22_1 | 2022 | LI3 | PV272226 | *Bacillus mesophilum* |
| 1274 | 13_mar_22 | 2022 | LI3 | PV272227 | *Bacillus licheniformis* |
| 1275 | 14_mar_22_2 | 2022 | LI3 | PV272228 | *Bacillus licheniformis* |
| 1276 | 14_mar_22_1 | 2022 | LI3 | PV272229 | *Bacillus licheniformis* |
| 1277 | 15_mar_22 | 2022 | LI3 | PV272230 | *Bacillus licheniformis* |
| 1278 | 16_mar_22 | 2022 | LI3 | PV272231 | *Bacillus licheniformis* |
| 1279 | 18_mar_22 | 2022 | LI3 | PV272232 | *Bacillus pumilus* |
| 1280 | 19_mar_22 | 2022 | LI3 | PV272233 | *Bacillus aequororis* |
| 1281 | 21_mar_22 | 2022 | LI3 | PV272234 | *Bacillus licheniformis* |
| 1282 | 22_mar_22 | 2022 | LI3 | PV272235 | *Bacillus licheniformis* |
| 1283 | 23_mar_22 | 2022 | LI3 | PV272236 | *Bacillus licheniformis* |
| 1284 | 26_mar_22 | 2022 | LI3 | PV272237 | *Kytococcus sedentarius* |
| 1285 | 27_mar_22 | 2022 | LI3 | PV272238 | *Bacillus vallismortis* |
| 1286 | 28_mar_22 | 2022 | LI3 | PV272239 | *Bacillus fengqiuensis* |
| 1287 | 30_mar_22 | 2022 | LI3 | PV272240 | *Pontibacter aurantiacus* |
| 1288 | 32_mar_22 | 2022 | LI3 | PV272241 | *Bacillus timonensis* |
| 1289 | 33_mar_22 | 2022 | LI3 | PV272242 | *Bacillus zhangzhouensis* |
| 1290 | 34_mar_22 | 2022 | E1 | PV272243 | *Shewanella putrefaciens* |
| 1291 | 35_mar_22 | 2022 | E1 | PV272244 | *Aeromonas salmonicida* |
| 1292 | 36_mar_22_1 | 2022 | E1 | PV272245 | *Bacillus australimaris* |
| 1293 | 36_mar_22_2 | 2022 | E1 | PV272246 | *Bacillus pumilus* |
| 1294 | 38_mar_22 | 2022 | E1 | PV272247 | *Rhizobium rosettiformans W3* |
| 1295 | 39_mar_22 | 2022 | E1 | PV272248 | *Pedobacter duraquae* |
| 1296 | 40_mar_22 | 2022 | E1 | PV272249 | *Cytobacillus oceanisediminis* |
| 1297 | 41_mar_22 | 2022 | E1 | PV272250 | *Bacillus licheniformis* |
| 1298 | 42_mar_22 | 2022 | E1 | PV272251 | *Bacillus aequororis* |
| 1299 | 43_mar_22 | 2022 | E2 | PV272252 | *Bacillus zhangzhouensis* |
| 1300 | 45_mar_22 | 2022 | E2 | PV272253 | *Shewanella putrefaciens* |
| 1301 | 46_mar_22 | 2022 | E2 | PV272254 | *Bacillus zhangzhouensis* |
| 1302 | 47_mar_22_2 | 2022 | E2 | PV272255 | *Mesobacillus jeotgali* |
| 1303 | 47_mar_22_1 | 2022 | E2 | PV272256 | *Mesobacillus jeotgali* |
| 1304 | 48_mar_22_2 | 2022 | E2 | PV272257 | *Bacillus mesophilum* |
| 1305 | 48_mar_22_1 | 2022 | E2 | PV272258 | *Bacillus mesophilum* |
| 1306 | 50_mar_22 | 2022 | E2 | PV272259 | *Bacillus licheniformis* |
| 1307 | 51_mar_22 | 2022 | E2 | PV272260 | *Bacillus paramycoides* |
| 1308 | 52_mar_22 | 2022 | E2 | PV272261 | *Bacillus stratosphericus* |
| 1309 | 53_mar_22 | 2022 | E2 | PV272262 | *Rheinheimera riviphila* |
| 1310 | 54_mar_22 | 2022 | E2 | PV272263 | *Bacillus zhangzhouensis* |
| 1311 | 55_mar_22 | 2022 | E2 | PV272264 | *Bacillus aequororis* |
| 1312 | 56_mar_22_2 | 2022 | E2 | PV272265 | *Bacillus spizizenii* |
| 1313 | 56_mar_22_1 | 2022 | E2 | PV272266 | *Bacillus spizizenii* |
| 1314 | 57_mar_22 | 2022 | E2 | PV272267 | *Bacillus safensis* |
| 1315 | 59_mar_22 | 2022 | E1 | PV272268 | *Bacillus zhangzhouensis* |
| 1316 | 60_mar_22 | 2022 | E1 | PV272269 | *Bacillus stratosphericus* |
| 1317 | 61_mar_22 | 2022 | E1 | PV272270 | *Shewanella putrefaciens* |
| 1318 | 63_mar_22 | 2022 | E1 | PV272271 | *Bacillus stratosphericus* |
| 1319 | 64_mar_22 | 2022 | E1 | PV272272 | *Bacillus fengqiuensis* |
| 1320 | 67_mar_22_1 | 2022 | LI1 | PV272273 | *Bacillus inaquosorum* |
| 1321 | 68_mar_22 | 2022 | LI1 | PV272274 | *Bacillus paramycoides* |
| 1322 | 69_mar_22 | 2022 | LI1 | PV272275 | *Paenarthrobacter nitroguajacolicus* |
| 1323 | 70_mar_22_2 | 2022 | LI1 | PV272276 | *Bacillus safensis* |
| 1324 | 70_mar_22_1 | 2022 | LI1 | PV272277 | *Bacillus pumilus* |
| 1325 | 72_mar_22 | 2022 | LI1 | PV272278 | *Priestia flexa* |
| 1326 | 73_mar_22 | 2022 | LI1 | PV272279 | *Bacillus paramycoides* |
| 1327 | 75_mar_22_1 | 2022 | LI1 | PV272280 | *Rossellomorea vietnamensis* |
| 1328 | 76_mar_22 | 2022 | LI1 | PV272281 | *Bacillus pakistanensis* |
| 1329 | 77_mar_22_1 | 2022 | LI1 | PV272282 | *Rossellomorea vietnamensis* |
| 1330 | 79_mar_22_3 | 2022 | LI1 | PV272283 | *Pontibacter silvestris* |
| 1331 | 79_mar_22_2 | 2022 | LI1 | PV272284 | *Pontibacter xinjiangensis* |
| 1332 | 79_mar_22_1 | 2022 | LI1 | PV272285 | *Pontibacter xinjiangensis* |
| 1333 | 80_mar_22 | 2022 | LI1 | PV272286 | *Bacillus thuringiensis* |
| 1334 | 82_mar_22 | 2022 | LI1 | PV272287 | *Bacillus thuringiensis* |
| 1335 | 83_mar_22_2 | 2022 | LI1 | PV272288 | *Bacillus proteolyticus* |
| 1336 | 83_mar_22_1 | 2022 | LI1 | PV272289 | *Pedobacter duraquae* |
| 1337 | 84_mar_22 | 2022 | LI1 | PV272290 | *Bacillus mesophilum* |
| 1338 | 86_mar_22 | 2022 | LI1 | PV272292 | *Sphingomonas desiccabilis* |
| 1339 | 88_mar_22 | 2022 | LI1 | PV272293 | *Pontibacter ummariensis* |
| 1340 | 90_mar_22 | 2022 | LI1 | PV272294 | *Bacillus proteolyticus* |
| 1341 | 95_mar_22_2 | 2022 | LI5 | PV272295 | *Oceanobacillus limi* |
| 1342 | 95_mar_22_1 | 2022 | LI5 | PV272296 | *Bacillus aequororis* |
| 1343 | 96_mar_22 | 2022 | LI5 | PV272297 | *Sutcliffiella halmapala* |
| 1344 | 98_mar_22 | 2022 | LI5 | PV272298 | *Kocuria aegyptia* |
| 1345 | 99_mar_22 | 2022 | LI5 | PV272299 | *Bacillus paramycoides* |
| 1346 | 100_mar_22 | 2022 | LI5 | PV272300 | *Rossellomorea vietnamensis* |
| 1347 | 103_mar_22 | 2022 | LI5 | PV272301 | *Rossellomorea vietnamensis* |
| 1348 | 104_mar_22 | 2022 | LI5 | PV272302 | *Brevibacillus formosus* |
| 1349 | 105_mar_22_1 | 2022 | LI5 | PV272303 | *Rossellomorea aquimaris* |
| 1350 | 105_mar_22_2 | 2022 | LI5 | PV272304 | *Rossellomorea aquimaris* |
| 1351 | 106_mar_22_1 | 2022 | LI5 | PV272305 | *Bacillus paramycoides* |
| 1352 | 107_mar_22_1 | 2022 | LI5 | PV272306 | *Bacillus licheniformis* |
| 1353 | 108_mar_22_2 | 2022 | LI5 | PV272307 | *Rossellomorea vietnamensis* |
| 1354 | 109_mar_22 | 2022 | LI5 | PV272308 | *Bacillus proteolyticus* |
| 1355 | 110_mar_22 | 2022 | LI5 | PV272309 | *Bacillus paramycoides* |
| 1356 | 111_mar_22 | 2022 | LI5 | PV272310 | *Bacillus licheniformis* |
| 1357 | 112_mar_22 | 2022 | LI5 | PV272311 | *Bacillus aequororis* |
| 1358 | 114_mar_22 | 2022 | LI5 | PV272312 | *Metabacillus sediminilitoris* |
| 1359 | 115_mar_22 | 2022 | LI5 | PV272313 | *Metabacillus crassostreae* |
| 1360 | 116_mar_22 | 2022 | LI5 | PV272314 | *Bacillus sonorensis* |
| 1361 | 117_mar_22 | 2022 | LI5 | PV272315 | *Metabacillus litoralis* |
| 1362 | 119_mar_22 | 2022 | LI6 | PV272316 | *Brevibacillus gelatini* |
| 1363 | 121_mar_22 | 2022 | LI6 | PV272317 | *Bacillus pakistanensis* |
| 1364 | 122_mar_22_1 | 2022 | LI6 | PV272318 | *Solibacillus silvestris* |
| 1365 | 124_mar_22 | 2022 | LI6 | PV272319 | *Bacillus mesophilum* |
| 1366 | 125_mar_22_1 | 2022 | LI6 | PV272320 | *Bacillus licheniformis* |
| 1367 | 126_mar_22 | 2022 | LI6 | PV272321 | *Rossellomorea vietnamensis* |
| 1368 | 127_mar_22 | 2022 | LI6 | PV272322 | *Bacillus aequororis* |
| 1369 | 128_mar_22 | 2022 | LI6 | PV272323 | *Rossellomorea vietnamensis* |
| 1370 | 129_mar_22 | 2022 | LI6 | PV272324 | *Bacillus sonorensis* |
| 1371 | 130_mar_22_1 | 2022 | LI6 | PV272325 | *Bacillus licheniformis* |
| 1372 | 131_mar_22 | 2022 | LI6 | PV272326 | *Brevibacillus formosus* |
| 1373 | 132_mar_22 | 2022 | LI6 | PV272327 | *Bacillus tropicus* |
| 1374 | 133_mar_22 | 2022 | LI6 | PV272328 | *Sutcliffiella deserti* |
| 1375 | 134_mar_22 | 2022 | LI6 | PV272329 | *Rossellomorea vietnamensis* |
| 1376 | 143_mar_22 | 2022 | LI7 | PV272330 | *Bacillus licheniformis* |
| 1377 | 145_mar_22 | 2022 | LI7 | PV272331 | *Bacillus thuringiensis* |
| 1378 | 147_mar_22 | 2022 | LI7 | PV272332 | *Bacillus aequororis* |
| 1379 | 148_mar_22_1 | 2022 | LI7 | PV272333 | *Bacillus aequororis* |
| 1380 | 151_mar_22_1 | 2022 | LI8 | PV272334 | *Microvirga roseola* |
| 1381 | 156_mar_22 | 2022 | LI8 | PV272335 | *Paracoccus aerius* |
| 1382 | 158_mar_22 | 2022 | LI8 | PV272336 | *Arthrobacter bussei* |
| 1383 | 162_mar_22 | 2022 | LI8 | PV272337 | *Massilia aurea* |
| 1384 | 165_mar_22 | 2022 | LI8 | PV272338 | *Arthrobacter nitrophenolicus* |
| 1385 | 174_mar_22 | 2022 | LI8 | PV272339 | *Bacillus licheniformis* |
| 1386 | 196_mar_22 | 2022 | LI8 | PV272340 | *Bacillus pakistanensis* |
| 1387 | 197_mar_22 | 2022 | LI8 | PV272341 | *Kocuria sediminis* |
| 1388 | 203_mar_22 | 2022 | LI8 | PV272342 | *Bacillus licheniformis* |
| 1389 | 225_mar_22_1 | 2022 | LI4 | PV272343 | *Bacillus paramycoides* |
| 1390 | 228_mar_22 | 2022 | LI4 | PV272344 | *Devosia oryziradicis* |
| 1391 | 229_mar_22_1 | 2022 | LI4 | PV272345 | *Ornithinibacillus contaminans* |
| 1392 | 229_mar_22_2 | 2022 | LI4 | PV272346 | *Sphingopyxis ginsengisoli* |
| 1393 | 230_mar_22 | 2022 | LI4 | PV272347 | *Bacillus licheniformis* |
| 1394 | 231_mar_22 | 2022 | LI4 | PV272348 | *Kocuria oceani* |
| 1395 | 232_mar_22 | 2022 | LI4 | PV272349 | *Rossellomorea vietnamensis* |
| 1396 | 234_mar_22 | 2022 | LI4 | PV272350 | *Bacillus aequororis* |
| 1397 | 236_mar_22_1 | 2022 | LI4 | PV272351 | *Rossellomorea vietnamensis* |
| 1398 | 237_mar_22 | 2022 | LI4 | PV272352 | *Cytobacillus luteolus* |
| 1399 | 245_mar_22 | 2022 | LI5 | PV272353 | *Bacillus aequororis* |
| 1400 | 255_mar_22 | 2022 | LI5 | PV272354 | *Paenibacillus lautus* |
| 1401 | 256_mar_22 | 2022 | LI5 | PV272355 | *Bacillus haikouensis* |
| 1402 | 260_mar_22 | 2022 | LI5 | PV272356 | *Sphingopyxis ginsengisoli* |
| 1403 | 262_mar_22 | 2022 | LI5 | PV272357 | *Bacillus licheniformis* |
| 1404 | 263_mar_22 | 2022 | LI5 | PV272358 | *Oceanobacillus manasiensis* |
| 1405 | 268_mar_22 | 2022 | LI5 | PV272359 | *Terribacillus goriensis* |
| 1406 | 270_mar_22 | 2022 | LI5 | PV272360 | *Caenispirillum bisanense* |
| 1407 | 271_mar_22_1 | 2022 | LI5 | PV272361 | *Bacillus zhangzhouensis* |
| 1408 | 272_mar_22_1 | 2022 | LI2 | PV272362 | *Bacillus cereus* |
| 1409 | 273_mar_22 | 2022 | LI2 | PV272363 | *Bacillus paramycoides* |
| 1410 | 274_mar_22 | 2022 | LI2 | PV272364 | *Rossellomorea vietnamensis* |
| 1411 | 275_mar_22 | 2022 | LI2 | PV272365 | *Brevibacillus formosus* |
| 1412 | 276_mar_22 | 2022 | LI2 | PV272366 | *Bacillus licheniformis* |
| 1413 | 279_mar_22 | 2022 | LI2 | PV272367 | *Bacillus paramycoides* |
| 1414 | 283_mar_22 | 2022 | LI2 | PV272368 | *Bacillus paramycoides* |
| 1415 | 288_mar_22 | 2022 | LI6 | PV272369 | *Bacillus pakistanensis* |
| 1416 | 10_2019_34_2_soil_dir | 2022 | LI2 | PV272370 | *Mesobacillus zeae* |
| 1417 | 10_2019_69_5_soil_dir | 2022 | LI5 | PV272371 | *Erythrobacter dokdonensis* |
| 1418 | 10_2019_70_5_soil_dir | 2022 | LI5 | PV272372 | *Bacillus proteolyticus* |
| 1419 | 10_2019_198_sitio7_soil_dir | 2022 | LI7 | PV272373 | *Rhizobium wuzhouense* |

| **Supplementary Table 8.** Relative and absolute abundances (n) of culturable bacterial genera isolated from the Churince hydrological system. | | | |
| --- | --- | --- | --- |
|  | **Ecological Phase** | | |
| **Genus** | **With_water** | **Transition** | **Desiccation** |
| *Bacillus* | 67.45% (317) | 32.37% (202) | 50.15% (163) |
| *Rossellomorea* | 13.62% (64) | 2.72% (17) | 8.0% (26) |
| *Metabacillus* | 2.13% (10) | 5.13% (32) | 2.46% (8) |
| *Aeromonas* | 0.0% (0) | 6.25% (39) | 2.15% (7) |
| *Rheinheimera* | 0.0% (0) | 5.45% (34) | 0.62% (2) |
| *Kocuria* | 1.06% (5) | 3.04% (19) | 1.54% (5) |
| *Staphylococcus* | 3.19% (15) | 0.0% (0) | 2.15% (7) |
| *Brevibacillus* | 0.0% (0) | 3.37% (21) | 1.85% (6) |
| *Jeotgalibacillus* | 1.49% (7) | 2.56% (16) | 0.92% (3) |
| *Cytobacillus* | 1.91% (9) | 1.44% (9) | 0.92% (3) |
| *Pseudomonas* | 0.21% (1) | 2.56% (16) | 0.62% (2) |
| *Paracoccus* | 0.21% (1) | 1.76% (11) | 1.23% (4) |
| *Sutcliffiella* | 0.43% (2) | 0.8% (5) | 1.85% (6) |
| *Peribacillus* | 1.7% (8) | 1.12% (7) | 0.0% (0) |
| *Planococcus* | 1.06% (5) | 1.44% (9) | 0.31% (1) |
| *Pannonibacter* | 0.0% (0) | 0.64% (4) | 2.15% (7) |
| *Exiguobacterium* | 0.43% (2) | 1.92% (12) | 0.31% (1) |
| *Arthrobacter* | 0.43% (2) | 0.8% (5) | 1.23% (4) |
| *Pontibacter* | 0.0% (0) | 0.48% (3) | 1.85% (6) |
| *Priestia* | 0.64% (3) | 0.32% (2) | 1.23% (4) |
| *Erythrobacter* | 0.0% (0) | 1.12% (7) | 0.92% (3) |
| *Mesobacillus* | 0.43% (2) | 0.16% (1) | 1.23% (4) |
| *Paenibacillus* | 0.0% (0) | 1.44% (9) | 0.31% (1) |
| *Rhizobium* | 0.0% (0) | 0.16% (1) | 1.54% (5) |
| *Shewanella* | 0.0% (0) | 0.64% (4) | 0.92% (3) |
| *Acinetobacter* | 0.0% (0) | 1.44% (9) | 0.0% (0) |
| *Brevundimonas* | 0.0% (0) | 1.44% (9) | 0.0% (0) |
| *Agrococcus* | 0.0% (0) | 0.64% (4) | 0.62% (2) |
| *Micrococcus* | 0.43% (2) | 0.16% (1) | 0.62% (2) |
| *Halomonas* | 0.0% (0) | 1.12% (7) | 0.0% (0) |
| *Fictibacillus* | 0.64% (3) | 0.16% (1) | 0.31% (1) |
| *Devosia* | 0.0% (0) | 0.32% (2) | 0.62% (2) |
| *Pseudarthrobacter* | 0.0% (0) | 0.32% (2) | 0.62% (2) |
| *Streptomyces* | 0.0% (0) | 0.8% (5) | 0.0% (0) |
| *Algoriphagus* | 0.0% (0) | 0.8% (5) | 0.0% (0) |
| *Paenarthrobacter* | 0.0% (0) | 0.48% (3) | 0.31% (1) |
| *Pararheinheimera* | 0.0% (0) | 0.48% (3) | 0.31% (1) |
| *Oceanobacillus* | 0.0% (0) | 0.16% (1) | 0.62% (2) |
| *Corynebacterium* | 0.64% (3) | 0.0% (0) | 0.0% (0) |
| *Cellulosimicrobium* | 0.0% (0) | 0.64% (4) | 0.0% (0) |
| *Microbacterium* | 0.0% (0) | 0.64% (4) | 0.0% (0) |
| *Bowmanella* | 0.0% (0) | 0.64% (4) | 0.0% (0) |
| *Tistrella* | 0.0% (0) | 0.64% (4) | 0.0% (0) |
| *Peteryoungia* | 0.0% (0) | 0.64% (4) | 0.0% (0) |
| *Microvirga* | 0.0% (0) | 0.32% (2) | 0.31% (1) |
| *Sphingomonas* | 0.0% (0) | 0.32% (2) | 0.31% (1) |
| *Gordonia* | 0.0% (0) | 0.0% (0) | 0.62% (2) |
| *Pedobacter* | 0.0% (0) | 0.0% (0) | 0.62% (2) |
| *Aeromicrobium* | 0.0% (0) | 0.0% (0) | 0.62% (2) |
| *Aquimonas* | 0.0% (0) | 0.0% (0) | 0.62% (2) |
| *Terribacillus* | 0.0% (0) | 0.0% (0) | 0.62% (2) |
| *Sphingopyxis* | 0.0% (0) | 0.0% (0) | 0.62% (2) |
| *Citricoccus* | 0.21% (1) | 0.32% (2) | 0.0% (0) |
| *Alkalihalobacillus* | 0.0% (0) | 0.48% (3) | 0.0% (0) |
| *Pseudidiomarina* | 0.0% (0) | 0.48% (3) | 0.0% (0) |
| *Uncultured* | 0.0% (0) | 0.48% (3) | 0.0% (0) |
| *Solibacillus* | 0.0% (0) | 0.16% (1) | 0.31% (1) |
| *Marinobacter* | 0.0% (0) | 0.16% (1) | 0.31% (1) |
| *Arenimonas* | 0.0% (0) | 0.16% (1) | 0.31% (1) |
| *Tabrizicola* | 0.0% (0) | 0.16% (1) | 0.31% (1) |
| *Qipengyuania* | 0.0% (0) | 0.16% (1) | 0.31% (1) |
| *Roseomonas* | 0.0% (0) | 0.16% (1) | 0.31% (1) |
| *Caenispirillum* | 0.0% (0) | 0.16% (1) | 0.31% (1) |
| *Planomicrobium* | 0.0% (0) | 0.16% (1) | 0.31% (1) |
| *Dietzia* | 0.43% (2) | 0.0% (0) | 0.0% (0) |
| *Lysinibacillus* | 0.43% (2) | 0.0% (0) | 0.0% (0) |
| *Brachybacterium* | 0.43% (2) | 0.0% (0) | 0.0% (0) |
| *Domibacillus* | 0.21% (1) | 0.16% (1) | 0.0% (0) |
| *Hoeflea* | 0.0% (0) | 0.32% (2) | 0.0% (0) |
| *Alphaproteobacteria* | 0.0% (0) | 0.32% (2) | 0.0% (0) |
| *Rubribacterium* | 0.0% (0) | 0.32% (2) | 0.0% (0) |
| *Methylorubrum* | 0.0% (0) | 0.32% (2) | 0.0% (0) |
| *Vibrio* | 0.0% (0) | 0.32% (2) | 0.0% (0) |
| *Robertmurraya* | 0.0% (0) | 0.32% (2) | 0.0% (0) |
| *Brevibacterium* | 0.0% (0) | 0.32% (2) | 0.0% (0) |
| *Mumia* | 0.0% (0) | 0.0% (0) | 0.31% (1) |
| *Novosphingobium* | 0.0% (0) | 0.0% (0) | 0.31% (1) |
| *Ornithinibacillus* | 0.0% (0) | 0.0% (0) | 0.31% (1) |
| *Pseudothioclava* | 0.0% (0) | 0.0% (0) | 0.31% (1) |
| *Kytococcus* | 0.0% (0) | 0.0% (0) | 0.31% (1) |
| *Massilia* | 0.0% (0) | 0.0% (0) | 0.31% (1) |
| *Oceanibaculum* | 0.0% (0) | 0.0% (0) | 0.31% (1) |
| *Salipiger* | 0.0% (0) | 0.0% (0) | 0.31% (1) |
| *Roseivivax* | 0.0% (0) | 0.0% (0) | 0.31% (1) |
| *Thalassobacillus* | 0.21% (1) | 0.0% (0) | 0.0% (0) |
| *Marinococcus* | 0.0% (0) | 0.16% (1) | 0.0% (0) |
| *Chryseomicrobium* | 0.0% (0) | 0.16% (1) | 0.0% (0) |
| *Kosakonia* | 0.0% (0) | 0.16% (1) | 0.0% (0) |
| *Gemmobacter* | 0.0% (0) | 0.16% (1) | 0.0% (0) |
| *Hyphomicrobiales* | 0.0% (0) | 0.16% (1) | 0.0% (0) |
| *Cellulomonas* | 0.0% (0) | 0.16% (1) | 0.0% (0) |
| *Agrobacterium* | 0.0% (0) | 0.16% (1) | 0.0% (0) |
| *Arsukibacterium* | 0.0% (0) | 0.16% (1) | 0.0% (0) |
| *Bacterium* | 0.0% (0) | 0.16% (1) | 0.0% (0) |
| *Aerococcus* | 0.0% (0) | 0.16% (1) | 0.0% (0) |
| *Cesiribacter* | 0.0% (0) | 0.16% (1) | 0.0% (0) |
| *Citrobacter* | 0.0% (0) | 0.16% (1) | 0.0% (0) |
| *Enterobacter* | 0.0% (0) | 0.16% (1) | 0.0% (0) |
| *Phenylobacterium* | 0.0% (0) | 0.16% (1) | 0.0% (0) |
| *Photobacterium* | 0.0% (0) | 0.16% (1) | 0.0% (0) |
| *Paenisporosarcina* | 0.0% (0) | 0.16% (1) | 0.0% (0) |
| *Pelagibacterium* | 0.0% (0) | 0.16% (1) | 0.0% (0) |
| *Muricauda* | 0.0% (0) | 0.16% (1) | 0.0% (0) |
| *Nocardioides* | 0.0% (0) | 0.16% (1) | 0.0% (0) |
| *Neobacillus* | 0.0% (0) | 0.16% (1) | 0.0% (0) |
| *Nesterenkonia* | 0.0% (0) | 0.16% (1) | 0.0% (0) |
| *Metapseudomonas* | 0.0% (0) | 0.16% (1) | 0.0% (0) |
| *Saccharothrix* | 0.0% (0) | 0.16% (1) | 0.0% (0) |
| *Rhodobacterales* | 0.0% (0) | 0.16% (1) | 0.0% (0) |
| *Salinarimonas* | 0.0% (0) | 0.16% (1) | 0.0% (0) |
| *Sphingobacterium* | 0.0% (0) | 0.16% (1) | 0.0% (0) |
| *Sphingobium* | 0.0% (0) | 0.16% (1) | 0.0% (0) |
| *Virgibacillus* | 0.0% (0) | 0.16% (1) | 0.0% (0) |
| *Zobellella* | 0.0% (0) | 0.16% (1) | 0.0% (0) |

| **Supplementary Table 9.** Relative and absolute abundances (n) of *Bacillus* spp. across ecological phases defined by water availability. | | | |
| --- | --- | --- | --- |
|  | **Ecological Phase** | | |
| ***Bacillus* species** | **With water** | **Transition** | **Desiccation** |
| *B. licheniformis* | 5.99% (19) | 22.77% (46) | 21.47% (35) |
| *B. aequororis* | 20.19% (64) | 2.97% (6) | 10.43% (17) |
| *B. paramycoides* | 14.51% (46) | 4.95% (10) | 7.98% (13) |
| *B. mesophilum* | 17.98% (57) | 3.47% (7) | 3.07% (5) |
| *Bacillus. sp* | 0.0% (0) | 22.77% (46) | 0.0% (0) |
| *B. proteolyticus* | 5.99% (19) | 1.49% (3) | 11.66% (19) |
| *B. thuringiensis* | 10.41% (33) | 2.97% (6) | 1.84% (3) |
| *B. inaquosorum* | 1.89% (6) | 11.39% (23) | 0.61% (1) |
| *B. zhangzhouensis* | 5.68% (18) | 0.99% (2) | 4.91% (8) |
| *B. spizizenii* | 0.32% (1) | 9.9% (20) | 1.23% (2) |
| *B. pakistanensis* | 5.36% (17) | 1.49% (3) | 3.68% (6) |
| *B. halotolerans* | 0.0% (0) | 0.0% (0) | 7.98% (13) |
| *B. stratosphericus* | 5.68% (18) | 0.0% (0) | 1.84% (3) |
| *B. vallismortis* | 0.32% (1) | 0.5% (1) | 5.52% (9) |
| *B. stercoris* | 1.26% (4) | 0.99% (2) | 2.45% (4) |
| *B. sonorensis* | 0.0% (0) | 0.99% (2) | 3.68% (6) |
| *B. safensis* | 1.58% (5) | 0.0% (0) | 1.84% (3) |
| *B. pumilus* | 0.63% (2) | 0.5% (1) | 1.84% (3) |
| *B. aerophilus* | 0.0% (0) | 2.48% (5) | 0.0% (0) |
| *B. cabrialesii* | 0.0% (0) | 2.48% (5) | 0.0% (0) |
| *B. coahuilensis* m4-4 | 0.0% (0) | 1.98% (4) | 0.0% (0) |
| *B. paranthracis* | 1.26% (4) | 0.0% (0) | 0.0% (0) |
| *B. subtilis* | 0.0% (0) | 0.0% (0) | 1.23% (2) |
| *B. fengqiuensis* | 0.0% (0) | 0.0% (0) | 1.23% (2) |
| *B. tropicus* | 0.0% (0) | 0.5% (1) | 0.61% (1) |
| *B. zanthoxyli* | 0.0% (0) | 0.5% (1) | 0.61% (1) |
| *B. tianshenii* | 0.0% (0) | 0.5% (1) | 0.61% (1) |
| *B. songklensis* | 0.0% (0) | 0.99% (2) | 0.0% (0) |
| *B. timonensis* | 0.0% (0) | 0.0% (0) | 0.61% (1) |
| *B. cereus* | 0.0% (0) | 0.0% (0) | 0.61% (1) |
| *B. atrophaeus* | 0.0% (0) | 0.0% (0) | 0.61% (1) |
| *B. haikouensis* | 0.0% (0) | 0.0% (0) | 0.61% (1) |
| *B. australimaris* | 0.0% (0) | 0.0% (0) | 0.61% (1) |
| *B. massiliglaciei* | 0.0% (0) | 0.0% (0) | 0.61% (1) |
| *B. gaemokensis* | 0.0% (0) | 0.5% (1) | 0.0% (0) |
| *B. coreaensis* | 0.0% (0) | 0.5% (1) | 0.0% (0) |
| *B. mojavensis* | 0.0% (0) | 0.5% (1) | 0.0% (0) |
| *B. tequilensis* | 0.0% (0) | 0.5% (1) | 0.0% (0) |
| *B. yapensis* | 0.0% (0) | 0.5% (1) | 0.0% (0) |
| *B. mobilis* | 0.32% (1) | 0.0% (0) | 0.0% (0) |
| *B. hominis* | 0.32% (1) | 0.0% (0) | 0.0% (0) |
| *B. wiedmannii* | 0.32% (1) | 0.0% (0) | 0.0% (0) |

| **Supplementary Table  10.** Kruskal–Wallis test of *Bacillus* spp. across ecological phases defined by water availability. | | | |
| --- | --- | --- | --- |
| **Taxonomy** | **Kruskal_H** | **p_value** | **Interpretation** |
| *Bacillus paramycoides* | 11.891 | 0.0026 | Significative |
| *Bacillus licheniformis* | 10.355 | 0.0056 | Significative |
| *Bacillus aequororis* | 9.633 | 0.0081 | Significative |
| *Bacillus mesophilum* | 7.403 | 0.0247 | Significative |
| *Bacillus pakistanensis* | 7.001 | 0.0302 | Significative |
| *Bacillus inaquosorum* | 6.944 | 0.0311 | Significative |
| *Bacillus pumilus* | 4 | 0.1353 | Not significative |
| *Bacillus spizizenii* | 3.918 | 0.141 | Not significative |
| *Bacillus proteolyticus* | 3.511 | 0.1728 | Not significative |
| *Bacillus safensis* | 0.75 | 0.3865 | Not significative |
| *Bacillus sonorensis* | 0.5 | 0.4795 | Not significative |
| *Bacillus thuringiensis* | 1.464 | 0.481 | Not significative |
| *Bacillus stercoris* | 1.333 | 0.5134 | Not significative |
| *Bacillus vallismortis* | 0.762 | 0.6832 | Not significative |
| *Bacillus zhangzhouensis* | 0.489 | 0.783 | Not significative |
| *Bacillus stratosphericus* | 0.019 | 0.8908 | Not significative |
| *Bacillus coahuilensis* m4-4 |  |  | Only in transition |
| *Bacillus australimaris* |  |  | Only in Desiccation |
| *Bacillus fengqiuensis* |  |  | Only in Desiccation |
| *Bacillus halotolerans* |  |  | Only in Desiccation |
| *Bacillus tequilensis* |  |  | Only in Transition |
| *Bacillus sp* |  |  | Only in Transition |
| *Bacillus cereus* |  |  | Only in Desiccation |
| *Bacillus paranthracis* |  |  | Only in With_Water |
| *Bacillus massiliglaciei* |  |  | Only in Desiccation |
| *Bacillus tianshenii* |  |  | Error to calculate |
| *Bacillus timonensis* |  |  | Only in Desiccation |
| *Bacillus cabrialesii* |  |  | Only in Transition |
| *Bacillus atrophaeus* |  |  | Only in Desiccation |
| *Bacillus haikouensis* |  |  | Only in Desiccation |
| *Bacillus tropicus* |  |  | Error to calculate |
| *Bacillus gaemokensis* |  |  | Only in Transition |
| *Bacillus hominis* |  |  | Just in With_Water |
| *Bacillus mobilis* |  |  | Just in With_Water |
| *Bacillus subtilis* |  |  | Just in Desiccation |
| *Bacillus wiedmannii* |  |  | Only in With_Water |
| *Bacillus zanthoxyli* |  |  | Error to calculate |
| *Bacillus songklensis* |  |  | Only in Transition |
| *Bacillus aerophilus* |  |  | Only in Transition |
| *Bacillus coreaensis* |  |  | Only in Transition |
| *Bacillus mojavensis* |  |  | Only in Transition |
| *Bacillus yapensis* |  |  | Only in Transition |

| **Supplementary Table 11.** Colony counts per phenotype from mesocosm experiments under heat-treated (spore-enriched) and non-heated (intact community) conditions. | | | | | | | | | |
| --- | --- | --- | --- | --- | --- | --- | --- | --- | --- |
| **Phenotype** | **Sample** | **Mesocosm treatment** | **Time** | **Plating Treatment** | **Dilution** | **Colonies** | **CFU/mL** | **Total Colonies** | **Total CFU/mL** |
| Medium white | IL4 - 1 | Untreated | 0 | Direct | 1 | 10 | 3.03E+03 | 36 | 1.09E+04 |
| Medium yellow | IL4 - 1 | Untreated | 0 | Direct | 1 | 1 | 3.03E+02 |  |  |
| Small yellow | IL4 - 1 | Untreated | 0 | Direct | 1 | 1 | 4.04E+02 |  |  |
| Small white | IL4 - 1 | Untreated | 0 | Direct | 1 | 18 | 5.35E+03 |  |  |
| Translucent yellow | IL4 - 1 | Untreated | 0 | Direct | 1 | 6 | 1.82E+03 |  |  |
| Medium white | IL4 - 1 | Untreated | 0 | Heat | 0 | 2 | 7.07E+01 | 3 | 1.01E+02 |
| Medium yellow | IL4 - 1 | Untreated | 0 | Heat | 0 | 0 | 1.01E+01 |  |  |
| Small melons | IL4 - 1 | Untreated | 0 | Heat | 0 | 1 | 2.02E+01 |  |  |
| Small yellow | IL4 - 2 | Untreated | 0 | Direct | 1 | 1 | 4.04E+02 | 13 | 4.04E+03 |
| Small melons | IL4 - 2 | Untreated | 0 | Direct | 1 | 0 | 1.01E+02 |  |  |
| Medium white | IL4 - 2 | Untreated | 0 | Direct | 1 | 5 | 1.52E+03 |  |  |
| Small white | IL4 - 2 | Untreated | 0 | Direct | 1 | 5 | 1.52E+03 |  |  |
| Medium translucent | IL4 - 2 | Untreated | 0 | Direct | 1 | 0 | 1.01E+02 |  |  |
| Translucent yellow | IL4 - 2 | Untreated | 0 | Direct | 1 | 1 | 4.04E+02 |  |  |
| Medium white | IL4 - 2 | Untreated | 0 | Heat | 0 | 2 | 7.07E+01 | 4 | 1.11E+02 |
| Pink | IL4 - 2 | Untreated | 0 | Heat | 0 | 0 | 1.01E+01 |  |  |
| Medium yellow | IL4 - 2 | Untreated | 0 | Heat | 0 | 0 | 1.01E+01 |  |  |
| Small white | IL4 - 2 | Untreated | 0 | Heat | 0 | 1 | 2.02E+01 |  |  |
| Medium pink | IL4 - 3 | Untreated | 0 | Direct | 0 | 0 | 1.01E+01 | 66 | 2.00E+03 |
| Medium yellow | IL4 - 3 | Untreated | 0 | Direct | 0 | 2 | 7.07E+01 |  |  |
| Small yellow | IL4 - 3 | Untreated | 0 | Direct | 0 | 16 | 4.85E+02 |  |  |
| Small melons | IL4 - 3 | Untreated | 0 | Direct | 0 | 1 | 2.02E+01 |  |  |
| Medium white | IL4 - 3 | Untreated | 0 | Direct | 0 | 17 | 5.25E+02 |  |  |
| Small white | IL4 - 3 | Untreated | 0 | Direct | 0 | 19 | 5.76E+02 |  |  |
| Translucent yellow | IL4 - 3 | Untreated | 0 | Direct | 0 | 10 | 3.13E+02 |  |  |
| Medium white | IL4 - 3 | Untreated | 0 | Heat | 0 | 1 | 3.03E+01 | 3 | 9.09E+01 |
| Small white | IL4 - 3 | Untreated | 0 | Heat | 0 | 2 | 6.06E+01 |  |  |
| Medium white | IL5 - 1 | Untreated | 0 | Direct | 0 | 35 | 1.05E+03 | 147 | 4.46E+03 |
| Medium pink | IL5 - 1 | Untreated | 0 | Direct | 0 | 1 | 2.02E+01 |  |  |
| Medium yellow | IL5 - 1 | Untreated | 0 | Direct | 0 | 4 | 1.21E+02 |  |  |
| Small yellow | IL5 - 1 | Untreated | 0 | Direct | 0 | 10 | 2.93E+02 |  |  |
| Small melons | IL5 - 1 | Untreated | 0 | Direct | 0 | 0 | 1.01E+01 |  |  |
| Small white | IL5 - 1 | Untreated | 0 | Direct | 0 | 96 | 2.90E+03 |  |  |
| Translucent yellow | IL5 - 1 | Untreated | 0 | Direct | 0 | 2 | 7.07E+01 |  |  |
| Medium white | IL5 - 1 | Untreated | 0 | Heat | 0 | 2 | 7.07E+01 | 5 | 1.41E+02 |
| Medium yellow | IL5 - 1 | Untreated | 0 | Heat | 0 | 0 | 1.01E+01 |  |  |
| Small yellow | IL5 - 1 | Untreated | 0 | Heat | 0 | 1 | 4.04E+01 |  |  |
| Small white | IL5 - 1 | Untreated | 0 | Heat | 0 | 1 | 2.02E+01 |  |  |
| Medium white | IL5 - 2 | Untreated | 0 | Direct | 1 | 22 | 6.77E+03 | 54 | 1.64E+04 |
| Medium yellow | IL5 - 2 | Untreated | 0 | Direct | 1 | 3 | 8.08E+02 |  |  |
| Small yellow | IL5 - 2 | Untreated | 0 | Direct | 1 | 3 | 9.09E+02 |  |  |
| Small melons | IL5 - 2 | Untreated | 0 | Direct | 1 | 0 | 1.01E+02 |  |  |
| Small white | IL5 - 2 | Untreated | 0 | Direct | 1 | 19 | 5.66E+03 |  |  |
| Translucent yellow | IL5 - 2 | Untreated | 0 | Direct | 1 | 7 | 2.12E+03 |  |  |
| Medium white | IL5 - 3 | Untreated | 0 | Direct | 1 | 22 | 6.57E+03 | 46 | 1.38E+04 |
| Medium yellow | IL5 - 3 | Untreated | 0 | Direct | 1 | 1 | 4.04E+02 |  |  |
| Small yellow | IL5 - 3 | Untreated | 0 | Direct | 1 | 2 | 7.07E+02 |  |  |
| Small melons | IL5 - 3 | Untreated | 0 | Direct | 1 | 1 | 3.03E+02 |  |  |
| Small white | IL5 - 3 | Untreated | 0 | Direct | 1 | 16 | 4.85E+03 |  |  |
| Translucent yellow | IL5 - 3 | Untreated | 0 | Direct | 1 | 3 | 1.01E+03 |  |  |
| Medium yellow | IL5 - 3 | Untreated | 0 | Heat | 0 | 0 | 1.01E+01 | 2 | 5.05E+01 |
| Small melons | IL5 - 3 | Untreated | 0 | Heat | 0 | 0 | 1.01E+01 |  |  |
| Small white | IL5 - 3 | Untreated | 0 | Heat | 0 | 1 | 3.03E+01 |  |  |
| Medium white | IL8 - 1 | Untreated | 0 | Direct | 1 | 43 | 1.31E+04 | 173 | 5.23E+04 |
| Small yellow | IL8 - 1 | Untreated | 0 | Direct | 1 | 4 | 1.11E+03 |  |  |
| Small white | IL8 - 1 | Untreated | 0 | Direct | 1 | 124 | 3.76E+04 |  |  |
| Translucent yellow | IL8 - 1 | Untreated | 0 | Direct | 1 | 2 | 5.05E+02 |  |  |
| Medium white | IL8 - 1 | Untreated | 0 | Heat | 0 | 5 | 1.52E+02 | 9 | 2.63E+02 |
| Small yellow | IL8 - 1 | Untreated | 0 | Heat | 0 | 1 | 2.02E+01 |  |  |
| Small white | IL8 - 1 | Untreated | 0 | Heat | 0 | 3 | 9.09E+01 |  |  |
| Medium yellow | IL8 - 2 | Untreated | 0 | Direct | 2 | 0 | 1.01E+03 | 19 | 5.76E+04 |
| Medium white | IL8 - 2 | Untreated | 0 | Direct | 2 | 10 | 3.03E+04 |  |  |
| Small white | IL8 - 2 | Untreated | 0 | Direct | 2 | 6 | 1.72E+04 |  |  |
| Translucent yellow | IL8 - 2 | Untreated | 0 | Direct | 2 | 3 | 9.09E+03 |  |  |
| Medium pink | IL8 - 2 | Untreated | 0 | Heat | 0 | 5 | 1.62E+02 | 19 | 5.76E+02 |
| Medium yellow | IL8 - 2 | Untreated | 0 | Heat | 0 | 0 | 1.01E+01 |  |  |
| Medium white | IL8 - 2 | Untreated | 0 | Heat | 0 | 4 | 1.21E+02 |  |  |
| Small white | IL8 - 2 | Untreated | 0 | Heat | 0 | 9 | 2.83E+02 |  |  |
| Small yellow | IL8 - 3 | Untreated | 0 | Direct | 2 | 1 | 4.04E+03 | 4 | 1.21E+04 |
| Small melons | IL8 - 3 | Untreated | 0 | Direct | 2 | 0 | 1.01E+03 |  |  |
| Medium white | IL8 - 3 | Untreated | 0 | Direct | 2 | 1 | 4.04E+03 |  |  |
| Small white | IL8 - 3 | Untreated | 0 | Direct | 2 | 1 | 3.03E+03 |  |  |
| Medium pink | IL8 - 3 | Untreated | 0 | Heat | 0 | 14 | 4.14E+02 | 25 | 7.58E+02 |
| Medium yellow | IL8 - 3 | Untreated | 0 | Heat | 0 | 2 | 6.06E+01 |  |  |
| Medium white | IL8 - 3 | Untreated | 0 | Heat | 0 | 4 | 1.21E+02 |  |  |
| Small white | IL8 - 3 | Untreated | 0 | Heat | 0 | 5 | 1.62E+02 |  |  |
| Medium white | IL4 - 1 | Heat | 0 | Direct | 0 | 9 | 2.73E+02 | 19 | 5.76E+02 |
| Medium yellow | IL4 - 1 | Heat | 0 | Direct | 0 | 1 | 3.03E+01 |  |  |
| Small yellow | IL4 - 1 | Heat | 0 | Direct | 0 | 8 | 2.42E+02 |  |  |
| Small white | IL4 - 1 | Heat | 0 | Direct | 0 | 1 | 3.03E+01 |  |  |
| Medium white | IL4 - 1 | Heat | 0 | Heat | 0 | 7 | 2.12E+02 | 16 | 4.85E+02 |
| Medium pink | IL4 - 1 | Heat | 0 | Heat | 0 | 1 | 3.03E+01 |  |  |
| Small yellow | IL4 - 1 | Heat | 0 | Heat | 0 | 3 | 9.09E+01 |  |  |
| Small white | IL4 - 1 | Heat | 0 | Heat | 0 | 5 | 1.52E+02 |  |  |
| Medium pink | IL4 - 2 | Heat | 0 | Direct | 0 | 2 | 6.06E+01 | 3 | 9.09E+01 |
| Translucent yellow | IL4 - 2 | Heat | 0 | Direct | 0 | 1 | 3.03E+01 |  |  |
| Medium white | IL4 - 2 | Heat | 0 | Heat | 1 | 9 | 2.73E+03 | 9 | 2.73E+03 |
| Medium white | IL4 -3 | Heat | 0 | Direct | 0 | 4 | 1.21E+02 | 8 | 2.42E+02 |
| Small yellow | IL4 -3 | Heat | 0 | Direct | 0 | 1 | 3.03E+01 |  |  |
| Small white | IL4 -3 | Heat | 0 | Direct | 0 | 3 | 9.09E+01 |  |  |
| Medium white | IL4 -3 | Heat | 0 | Heat | 0 | 2 | 6.06E+01 | 4 | 1.21E+02 |
| Medium pink | IL4 -3 | Heat | 0 | Heat | 0 | 1 | 3.03E+01 |  |  |
| Small white | IL4 -3 | Heat | 0 | Heat | 0 | 1 | 3.03E+01 |  |  |
| Medium white | IL5 - 1 | Heat | 0 | Direct | 0 | 10 | 3.03E+02 | 73 | 2.21E+03 |
| Medium pink | IL5 - 1 | Heat | 0 | Direct | 0 | 12 | 3.64E+02 |  |  |
| Medium yellow | IL5 - 1 | Heat | 0 | Direct | 0 | 8 | 2.42E+02 |  |  |
| Small yellow | IL5 - 1 | Heat | 0 | Direct | 0 | 25 | 7.58E+02 |  |  |
| Small melons | IL5 - 1 | Heat | 0 | Direct | 0 | 2 | 6.06E+01 |  |  |
| Small white | IL5 - 1 | Heat | 0 | Direct | 0 | 16 | 4.85E+02 |  |  |
| Medium white | IL5 - 1 | Heat | 0 | Heat | 0 | 11 | 3.33E+02 | 49 | 1.48E+03 |
| Medium pink | IL5 - 1 | Heat | 0 | Heat | 0 | 3 | 9.09E+01 |  |  |
| Medium yellow | IL5 - 1 | Heat | 0 | Heat | 0 | 3 | 9.09E+01 |  |  |
| Small yellow | IL5 - 1 | Heat | 0 | Heat | 0 | 14 | 4.24E+02 |  |  |
| Small white | IL5 - 1 | Heat | 0 | Heat | 0 | 18 | 5.45E+02 |  |  |
| Medium pink | IL5 - 2 | Heat | 0 | Direct | 0 | 6 | 1.82E+02 | 84 | 2.55E+03 |
| Medium yellow | IL5 - 2 | Heat | 0 | Direct | 0 | 3 | 9.09E+01 |  |  |
| Small yellow | IL5 - 2 | Heat | 0 | Direct | 0 | 42 | 1.27E+03 |  |  |
| Small melons | IL5 - 2 | Heat | 0 | Direct | 0 | 3 | 9.09E+01 |  |  |
| Medium white | IL5 - 2 | Heat | 0 | Direct | 0 | 4 | 1.21E+02 |  |  |
| Small white | IL5 - 2 | Heat | 0 | Direct | 0 | 26 | 7.88E+02 |  |  |
| Medium white | IL5 - 2 | Heat | 0 | Heat | 0 | 24 | 7.27E+02 | 57 | 1.73E+03 |
| Medium pink | IL5 - 2 | Heat | 0 | Heat | 0 | 3 | 9.09E+01 |  |  |
| Medium yellow | IL5 - 2 | Heat | 0 | Heat | 0 | 3 | 9.09E+01 |  |  |
| Small yellow | IL5 - 2 | Heat | 0 | Heat | 0 | 17 | 5.15E+02 |  |  |
| Small melons | IL5 - 2 | Heat | 0 | Heat | 0 | 1 | 3.03E+01 |  |  |
| Small white | IL5 - 2 | Heat | 0 | Heat | 0 | 9 | 2.73E+02 |  |  |
| Medium white | IL5 - 3 | Heat | 0 | Direct | 0 | 22 | 6.67E+02 | 84 | 2.55E+03 |
| Medium pink | IL5 - 3 | Heat | 0 | Direct | 0 | 4 | 1.21E+02 |  |  |
| Medium yellow | IL5 - 3 | Heat | 0 | Direct | 0 | 7 | 2.12E+02 |  |  |
| Small yellow | IL5 - 3 | Heat | 0 | Direct | 0 | 21 | 6.36E+02 |  |  |
| Small white | IL5 - 3 | Heat | 0 | Direct | 0 | 30 | 9.09E+02 |  |  |
| Medium white | IL5 - 3 | Heat | 0 | Heat | 0 | 10 | 3.03E+02 | 47 | 1.42E+03 |
| Medium pink | IL5 - 3 | Heat | 0 | Heat | 0 | 13 | 3.94E+02 |  |  |
| Medium yellow | IL5 - 3 | Heat | 0 | Heat | 0 | 1 | 3.03E+01 |  |  |
| Small yellow | IL5 - 3 | Heat | 0 | Heat | 0 | 11 | 3.33E+02 |  |  |
| Small melons | IL5 - 3 | Heat | 0 | Heat | 0 | 1 | 3.03E+01 |  |  |
| Small white | IL5 - 3 | Heat | 0 | Heat | 0 | 11 | 3.33E+02 |  |  |
| Medium pink | IL8 - 1 | Heat | 0 | Direct | 0 | 33 | 1.00E+03 | 139 | 4.21E+03 |
| Medium yellow | IL8 - 1 | Heat | 0 | Direct | 0 | 3 | 9.09E+01 |  |  |
| Small yellow | IL8 - 1 | Heat | 0 | Direct | 0 | 17 | 5.15E+02 |  |  |
| Medium white | IL8 - 1 | Heat | 0 | Direct | 0 | 4 | 1.21E+02 |  |  |
| Small white | IL8 - 1 | Heat | 0 | Direct | 0 | 80 | 2.42E+03 |  |  |
| Translucent yellow | IL8 - 1 | Heat | 0 | Direct | 0 | 2 | 6.06E+01 |  |  |
| Medium pink | IL8 - 1 | Heat | 0 | Heat | 0 | 40 | 1.21E+03 | 81 | 2.45E+03 |
| Small yellow | IL8 - 1 | Heat | 0 | Heat | 0 | 1 | 3.03E+01 |  |  |
| Medium white | IL8 - 1 | Heat | 0 | Heat | 0 | 2 | 6.06E+01 |  |  |
| Small white | IL8 - 1 | Heat | 0 | Heat | 0 | 38 | 1.15E+03 |  |  |
| Medium white | IL8 - 2 | Heat | 0 | Direct | 0 | 9 | 2.73E+02 | 73 | 2.21E+03 |
| Medium pink | IL8 - 2 | Heat | 0 | Direct | 0 | 12 | 3.64E+02 |  |  |
| Medium yellow | IL8 - 2 | Heat | 0 | Direct | 0 | 1 | 3.03E+01 |  |  |
| Small yellow | IL8 - 2 | Heat | 0 | Direct | 0 | 7 | 2.12E+02 |  |  |
| Small white | IL8 - 2 | Heat | 0 | Direct | 0 | 44 | 1.33E+03 |  |  |
| Medium white | IL8 - 2 | Heat | 0 | Heat | 0 | 13 | 3.94E+02 | 108 | 3.27E+03 |
| Medium pink | IL8 - 2 | Heat | 0 | Heat | 0 | 34 | 1.03E+03 |  |  |
| Small yellow | IL8 - 2 | Heat | 0 | Heat | 0 | 1 | 3.03E+01 |  |  |
| Small white | IL8 - 2 | Heat | 0 | Heat | 0 | 60 | 1.82E+03 |  |  |
| Medium pink | IL8 - 3 | Heat | 0 | Direct | 0 | 31 | 9.39E+02 | 80 | 2.42E+03 |
| Small yellow | IL8 - 3 | Heat | 0 | Direct | 0 | 7 | 2.12E+02 |  |  |
| Medium white | IL8 - 3 | Heat | 0 | Direct | 0 | 3 | 9.09E+01 |  |  |
| Small white | IL8 - 3 | Heat | 0 | Direct | 0 | 38 | 1.15E+03 |  |  |
| Translucent yellow | IL8 - 3 | Heat | 0 | Direct | 0 | 1 | 3.03E+01 |  |  |
| Medium pink | IL8 - 3 | Heat | 0 | Heat | 0 | 33 | 1.00E+03 | 67 | 2.03E+03 |
| Medium yellow | IL8 - 3 | Heat | 0 | Heat | 0 | 1 | 3.03E+01 |  |  |
| Small white | IL8 - 3 | Heat | 0 | Heat | 0 | 33 | 1.00E+03 |  |  |
| Medium white | IL4 - 1 | Untreated | 1 | Direct | 0 | 8 | 2.42E+02 | 31 | 9.39E+02 |
| Medium pink | IL4 - 1 | Untreated | 1 | Direct | 0 | 1 | 3.03E+01 |  |  |
| Pink | IL4 - 1 | Untreated | 1 | Direct | 0 | 4 | 1.21E+02 |  |  |
| Small yellow | IL4 - 1 | Untreated | 1 | Direct | 0 | 12 | 3.64E+02 |  |  |
| Translucent yellow | IL4 - 1 | Untreated | 1 | Direct | 0 | 6 | 1.82E+02 |  |  |
| Medium white | IL4 - 1 | Untreated | 1 | Heat | 0 | 1 | 3.03E+01 | 1 | 3.03E+01 |
| Medium white | IL4 - 2 | Untreated | 1 | Direct | 1 | 2 | 6.06E+02 | 19 | 5.76E+03 |
| Pink | IL4 - 2 | Untreated | 1 | Direct | 1 | 2 | 6.06E+02 |  |  |
| Small yellow | IL4 - 2 | Untreated | 1 | Direct | 1 | 1 | 3.03E+02 |  |  |
| Small white | IL4 - 2 | Untreated | 1 | Direct | 1 | 1 | 3.03E+02 |  |  |
| Medium translucent | IL4 - 2 | Untreated | 1 | Direct | 1 | 3 | 9.09E+02 |  |  |
| Small translucent | IL4 - 2 | Untreated | 1 | Direct | 1 | 10 | 3.03E+03 |  |  |
| Pink | IL4 - 3 | Untreated | 1 | Direct | 0 | 1 | 3.03E+01 | 45 | 1.36E+03 |
| Medium yellow | IL4 - 3 | Untreated | 1 | Direct | 0 | 20 | 6.06E+02 |  |  |
| Small melons | IL4 - 3 | Untreated | 1 | Direct | 0 | 10 | 3.03E+02 |  |  |
| Medium white | IL4 - 3 | Untreated | 1 | Direct | 0 | 2 | 6.06E+01 |  |  |
| Small white | IL4 - 3 | Untreated | 1 | Direct | 0 | 12 | 3.64E+02 |  |  |
| Small melons | IL4 - 3 | Untreated | 1 | Heat | 0 | 1 | 3.03E+01 | 2 | 6.06E+01 |
| Medium white | IL4 - 3 | Untreated | 1 | Heat | 0 | 1 | 3.03E+01 |  |  |
| Medium white | IL5 - 1 | Untreated | 1 | Direct | 0 | 4 | 1.21E+02 | 156 | 4.73E+03 |
| Small melons | IL5 - 1 | Untreated | 1 | Direct | 0 | 8 | 2.42E+02 |  |  |
| Small white | IL5 - 1 | Untreated | 1 | Direct | 0 | 60 | 1.82E+03 |  |  |
| Large translucent | IL5 - 1 | Untreated | 1 | Direct | 0 | 4 | 1.21E+02 |  |  |
| Medium translucent | IL5 - 1 | Untreated | 1 | Direct | 0 | 4 | 1.21E+02 |  |  |
| Translucent yellow | IL5 - 1 | Untreated | 1 | Direct | 0 | 76 | 2.30E+03 |  |  |
| Pink | IL5 - 1 | Untreated | 1 | Heat | 0 | 1 | 3.03E+01 | 2 | 6.06E+01 |
| Small white | IL5 - 1 | Untreated | 1 | Heat | 0 | 1 | 3.03E+01 |  |  |
| Pink | IL5 - 2 | Untreated | 1 | Direct | 0 | 48 | 1.45E+03 | 144 | 4.36E+03 |
| Small yellow | IL5 - 2 | Untreated | 1 | Direct | 0 | 8 | 2.42E+02 |  |  |
| Small white | IL5 - 2 | Untreated | 1 | Direct | 0 | 16 | 4.85E+02 |  |  |
| Medium translucent | IL5 - 2 | Untreated | 1 | Direct | 0 | 52 | 1.58E+03 |  |  |
| Small translucent | IL5 - 2 | Untreated | 1 | Direct | 0 | 20 | 6.06E+02 |  |  |
| Pink | IL5 - 2 | Untreated | 1 | Heat | 0 | 3 | 9.09E+01 | 9 | 2.73E+02 |
| Medium yellow | IL5 - 2 | Untreated | 1 | Heat | 0 | 2 | 6.06E+01 |  |  |
| Small melons | IL5 - 2 | Untreated | 1 | Heat | 0 | 1 | 3.03E+01 |  |  |
| Small white | IL5 - 2 | Untreated | 1 | Heat | 0 | 1 | 3.03E+01 |  |  |
| Small translucent | IL5 - 2 | Untreated | 1 | Heat | 0 | 2 | 6.06E+01 |  |  |
| Small melons | IL5 - 3 | Untreated | 1 | Direct | 0 | 16 | 4.85E+02 | 116 | 3.52E+03 |
| Small white | IL5 - 3 | Untreated | 1 | Direct | 0 | 28 | 8.48E+02 |  |  |
| Large translucent | IL5 - 3 | Untreated | 1 | Direct | 0 | 4 | 1.21E+02 |  |  |
| Medium translucent | IL5 - 3 | Untreated | 1 | Direct | 0 | 28 | 8.48E+02 |  |  |
| Small translucent | IL5 - 3 | Untreated | 1 | Direct | 0 | 40 | 1.21E+03 |  |  |
| Pink | IL5 - 3 | Untreated | 1 | Heat | 0 | 1 | 3.03E+01 | 1 | 3.03E+01 |
| Medium pink | IL8 - 1 | Untreated | 1 | Direct | 0 | 4 | 1.21E+02 | 252 | 7.64E+03 |
| Small yellow | IL8 - 1 | Untreated | 1 | Direct | 0 | 4 | 1.21E+02 |  |  |
| Medium white | IL8 - 1 | Untreated | 1 | Direct | 0 | 144 | 4.36E+03 |  |  |
| Small white | IL8 - 1 | Untreated | 1 | Direct | 0 | 60 | 1.82E+03 |  |  |
| Medium translucent | IL8 - 1 | Untreated | 1 | Direct | 0 | 40 | 1.21E+03 |  |  |
| Medium white | IL8 - 1 | Untreated | 1 | Heat | 0 | 6 | 1.82E+02 | 8 | 2.42E+02 |
| Small white | IL8 - 1 | Untreated | 1 | Heat | 0 | 2 | 6.06E+01 |  |  |
| Medium white | IL8 - 2 | Untreated | 1 | Direct | 0 | 41 | 1.24E+03 | 157 | 4.76E+03 |
| Medium translucent | IL8 - 2 | Untreated | 1 | Direct | 0 | 52 | 1.58E+03 |  |  |
| Small translucent | IL8 - 2 | Untreated | 1 | Direct | 0 | 64 | 1.94E+03 |  |  |
| Medium pink | IL8 - 2 | Untreated | 1 | Heat | 0 | 2 | 6.06E+01 | 9 | 2.73E+02 |
| Medium yellow | IL8 - 2 | Untreated | 1 | Heat | 0 | 1 | 3.03E+01 |  |  |
| Medium white | IL8 - 2 | Untreated | 1 | Heat | 0 | 4 | 1.21E+02 |  |  |
| Small white | IL8 - 2 | Untreated | 1 | Heat | 0 | 2 | 6.06E+01 |  |  |
| Small melons | IL8 - 3 | Untreated | 1 | Direct | 1 | 15 | 4.55E+03 | 77 | 2.33E+04 |
| Medium white | IL8 - 3 | Untreated | 1 | Direct | 1 | 21 | 6.36E+03 |  |  |
| Small white | IL8 - 3 | Untreated | 1 | Direct | 1 | 6 | 1.82E+03 |  |  |
| Medium translucent | IL8 - 3 | Untreated | 1 | Direct | 1 | 22 | 6.67E+03 |  |  |
| Translucent yellow | IL8 - 3 | Untreated | 1 | Direct | 1 | 13 | 3.94E+03 |  |  |
| Medium yellow | IL8 - 3 | Untreated | 1 | Heat | 0 | 1 | 3.03E+01 | 7 | 2.12E+02 |
| Small yellow | IL8 - 3 | Untreated | 1 | Heat | 0 | 5 | 1.52E+02 |  |  |
| Small white | IL8 - 3 | Untreated | 1 | Heat | 0 | 1 | 3.03E+01 |  |  |
| Pink | IL4 - 1 | Heat | 1 | Direct | 2 | 1 | 3.03E+03 | 41 | 1.24E+05 |
| Medium yellow | IL4 - 1 | Heat | 1 | Direct | 2 | 24 | 7.27E+04 |  |  |
| Small melons | IL4 - 1 | Heat | 1 | Direct | 2 | 9 | 2.73E+04 |  |  |
| Medium white | IL4 - 1 | Heat | 1 | Direct | 2 | 7 | 2.12E+04 |  |  |
| Medium yellow | IL4 - 1 | Heat | 1 | Heat | 2 | 26 | 7.88E+04 | 29 | 8.79E+04 |
| Small melons | IL4 - 1 | Heat | 1 | Heat | 2 | 2 | 6.06E+03 |  |  |
| Medium white | IL4 - 1 | Heat | 1 | Heat | 2 | 1 | 3.03E+03 |  |  |
| Pink | IL4 - 2 | Heat | 1 | Direct | 2 | 1 | 3.03E+03 | 41 | 1.24E+05 |
| Medium yellow | IL4 - 2 | Heat | 1 | Direct | 2 | 24 | 7.27E+04 |  |  |
| Small melons | IL4 - 2 | Heat | 1 | Direct | 2 | 9 | 2.73E+04 |  |  |
| Medium white | IL4 - 2 | Heat | 1 | Direct | 2 | 7 | 2.12E+04 |  |  |
| Medium yellow | IL4 - 2 | Heat | 1 | Heat | 2 | 26 | 7.88E+04 | 29 | 8.79E+04 |
| Small melons | IL4 - 2 | Heat | 1 | Heat | 2 | 2 | 6.06E+03 |  |  |
| Medium white | IL4 - 2 | Heat | 1 | Heat | 2 | 1 | 3.03E+03 |  |  |
| Medium yellow | IL4 -3 | Heat | 1 | Direct | 2 | 88 | 2.67E+05 | 132 | 4.00E+05 |
| Small melons | IL4 -3 | Heat | 1 | Direct | 2 | 8 | 2.42E+04 |  |  |
| Small white | IL4 -3 | Heat | 1 | Direct | 2 | 36 | 1.09E+05 |  |  |
| Medium yellow | IL4 -3 | Heat | 1 | Heat | 2 | 28 | 8.48E+04 | 39 | 1.18E+05 |
| Medium white | IL4 -3 | Heat | 1 | Heat | 2 | 11 | 3.33E+04 |  |  |
| Pink | IL5 - 1 | Heat | 1 | Direct | 2 | 34 | 1.03E+05 | 158 | 4.79E+05 |
| Medium yellow | IL5 - 1 | Heat | 1 | Direct | 2 | 18 | 5.45E+04 |  |  |
| Small yellow | IL5 - 1 | Heat | 1 | Direct | 2 | 64 | 1.94E+05 |  |  |
| Medium white | IL5 - 1 | Heat | 1 | Direct | 2 | 12 | 3.64E+04 |  |  |
| Small white | IL5 - 1 | Heat | 1 | Direct | 2 | 30 | 9.09E+04 |  |  |
| Pink | IL5 - 1 | Heat | 1 | Heat | 1 | 45 | 1.36E+04 | 104 | 3.15E+04 |
| Medium yellow | IL5 - 1 | Heat | 1 | Heat | 1 | 5 | 1.52E+03 |  |  |
| Small yellow | IL5 - 1 | Heat | 1 | Heat | 1 | 29 | 8.79E+03 |  |  |
| Small white | IL5 - 1 | Heat | 1 | Heat | 1 | 25 | 7.58E+03 |  |  |
| Medium white | IL5 - 2 | Heat | 1 | Direct | 2 | 1 | 3.03E+03 | 163 | 4.94E+05 |
| Pink | IL5 - 2 | Heat | 1 | Direct | 2 | 90 | 2.73E+05 |  |  |
| Medium yellow | IL5 - 2 | Heat | 1 | Direct | 2 | 13 | 3.94E+04 |  |  |
| Small yellow | IL5 - 2 | Heat | 1 | Direct | 2 | 37 | 1.12E+05 |  |  |
| Small white | IL5 - 2 | Heat | 1 | Direct | 2 | 22 | 6.67E+04 |  |  |
| Pink | IL5 - 2 | Heat | 1 | Heat | 1 | 9 | 2.73E+03 | 188 | 5.70E+04 |
| Medium yellow | IL5 - 2 | Heat | 1 | Heat | 1 | 9 | 2.73E+03 |  |  |
| Small yellow | IL5 - 2 | Heat | 1 | Heat | 1 | 150 | 4.55E+04 |  |  |
| Small white | IL5 - 2 | Heat | 1 | Heat | 1 | 20 | 6.06E+03 |  |  |
| Medium white | IL5 - 3 | Heat | 1 | Direct | 2 | 2 | 6.06E+03 | 158 | 4.79E+05 |
| Pink | IL5 - 3 | Heat | 1 | Direct | 2 | 76 | 2.30E+05 |  |  |
| Medium yellow | IL5 - 3 | Heat | 1 | Direct | 2 | 11 | 3.33E+04 |  |  |
| Small yellow | IL5 - 3 | Heat | 1 | Direct | 2 | 40 | 1.21E+05 |  |  |
| Small white | IL5 - 3 | Heat | 1 | Direct | 2 | 29 | 8.79E+04 |  |  |
| Medium yellow | IL5 - 3 | Heat | 1 | Heat | 2 | 23 | 6.97E+04 | 30 | 9.09E+04 |
| Small yellow | IL5 - 3 | Heat | 1 | Heat | 2 | 4 | 1.21E+04 |  |  |
| Medium white | IL5 - 3 | Heat | 1 | Heat | 2 | 3 | 9.09E+03 |  |  |
| Medium yellow | IL8 - 1 | Heat | 1 | Direct | 2 | 64 | 1.94E+05 | 80 | 2.42E+05 |
| Small melons | IL8 - 1 | Heat | 1 | Direct | 2 | 2 | 6.06E+03 |  |  |
| Medium white | IL8 - 1 | Heat | 1 | Direct | 2 | 14 | 4.24E+04 |  |  |
| Medium yellow | IL8 - 1 | Heat | 1 | Heat | 2 | 64 | 1.94E+05 | 76 | 2.30E+05 |
| Medium white | IL8 - 1 | Heat | 1 | Heat | 2 | 12 | 3.64E+04 |  |  |
| Medium yellow | IL8 - 2 | Heat | 1 | Direct | 2 | 98 | 2.97E+05 | 138 | 4.18E+05 |
| Medium white | IL8 - 2 | Heat | 1 | Direct | 2 | 16 | 4.85E+04 |  |  |
| Small white | IL8 - 2 | Heat | 1 | Direct | 2 | 24 | 7.27E+04 |  |  |
| Small yellow | IL8 - 2 | Heat | 1 | Heat | 2 | 94 | 2.85E+05 | 106 | 3.21E+05 |
| Small melons | IL8 - 2 | Heat | 1 | Heat | 2 | 2 | 6.06E+03 |  |  |
| Medium white | IL8 - 2 | Heat | 1 | Heat | 2 | 6 | 1.82E+04 |  |  |
| Small white | IL8 - 2 | Heat | 1 | Heat | 2 | 4 | 1.21E+04 |  |  |
| Medium yellow | IL8 - 3 | Heat | 1 | Direct | 2 | 182 | 5.52E+05 | 214 | 6.48E+05 |
| Small melons | IL8 - 3 | Heat | 1 | Direct | 2 | 2 | 6.06E+03 |  |  |
| Medium white | IL8 - 3 | Heat | 1 | Direct | 2 | 28 | 8.48E+04 |  |  |
| Small white | IL8 - 3 | Heat | 1 | Direct | 2 | 2 | 6.06E+03 |  |  |
| Medium yellow | IL8 - 3 | Heat | 1 | Heat | 2 | 76 | 2.30E+05 | 92 | 2.79E+05 |
| Medium white | IL8 - 3 | Heat | 1 | Heat | 2 | 12 | 3.64E+04 |  |  |
| Small white | IL8 - 3 | Heat | 1 | Heat | 2 | 4 | 1.21E+04 |  |  |
| Medium white | IL4 - 1 | Untreated | 2 | Direct | 0 | 2 | 6.06E+01 | 49 | 1.48E+03 |
| Pink | IL4 - 1 | Untreated | 2 | Direct | 0 | 4 | 1.21E+02 |  |  |
| Small yellow | IL4 - 1 | Untreated | 2 | Direct | 0 | 8 | 2.42E+02 |  |  |
| Small white | IL4 - 1 | Untreated | 2 | Direct | 0 | 19 | 5.76E+02 |  |  |
| Translucent yellow | IL4 - 1 | Untreated | 2 | Direct | 0 | 16 | 4.85E+02 |  |  |
| Medium yellow | IL4 - 1 | Untreated | 2 | Heat | 0 | 2 | 6.06E+01 | 4 | 1.21E+02 |
| Small yellow | IL4 - 1 | Untreated | 2 | Heat | 0 | 1 | 3.03E+01 |  |  |
| Medium white | IL4 - 1 | Untreated | 2 | Heat | 0 | 1 | 3.03E+01 |  |  |
| Medium yellow | IL4 - 2 | Untreated | 2 | Direct | 0 | 1 | 3.03E+01 | 79 | 2.39E+03 |
| Small yellow | IL4 - 2 | Untreated | 2 | Direct | 0 | 25 | 7.58E+02 |  |  |
| Small melons | IL4 - 2 | Untreated | 2 | Direct | 0 | 2 | 6.06E+01 |  |  |
| Small white | IL4 - 2 | Untreated | 2 | Direct | 0 | 4 | 1.21E+02 |  |  |
| Translucent yellow | IL4 - 2 | Untreated | 2 | Direct | 0 | 4 | 1.21E+02 |  |  |
| Medium oranges | IL4 - 2 | Untreated | 2 | Direct | 0 | 43 | 1.30E+03 |  |  |
| Medium yellow | IL4 - 2 | Untreated | 2 | Heat | 0 | 1 | 3.03E+01 | 1 | 3.03E+01 |
| Medium white | IL4 - 3 | Untreated | 2 | Direct | 2 | 1 | 3.03E+03 | 2 | 6.06E+03 |
| Translucent yellow | IL4 - 3 | Untreated | 2 | Direct | 2 | 1 | 3.03E+03 |  |  |
| Medium white | IL4 - 3 | Untreated | 2 | Heat | 3 | 1 | 3.03E+04 | 1 | 3.03E+04 |
| Medium white | IL5 - 1 | Untreated | 2 | Direct | 0 | 16 | 4.85E+02 | 67 | 2.03E+03 |
| Medium yellow | IL5 - 1 | Untreated | 2 | Direct | 0 | 1 | 3.03E+01 |  |  |
| Small yellow | IL5 - 1 | Untreated | 2 | Direct | 0 | 1 | 3.03E+01 |  |  |
| Small white | IL5 - 1 | Untreated | 2 | Direct | 0 | 29 | 8.79E+02 |  |  |
| Medium beige | IL5 - 1 | Untreated | 2 | Direct | 0 | 20 | 6.06E+02 |  |  |
| Small yellow | IL5 - 2 | Untreated | 2 | Direct | 0 | 12 | 3.64E+02 | 59 | 1.79E+03 |
| Small melons | IL5 - 2 | Untreated | 2 | Direct | 0 | 4 | 1.21E+02 |  |  |
| Medium white | IL5 - 2 | Untreated | 2 | Direct | 0 | 22 | 6.67E+02 |  |  |
| Small white | IL5 - 2 | Untreated | 2 | Direct | 0 | 16 | 4.85E+02 |  |  |
| Medium translucent | IL5 - 2 | Untreated | 2 | Direct | 0 | 2 | 6.06E+01 |  |  |
| Small translucent | IL5 - 2 | Untreated | 2 | Direct | 0 | 3 | 9.09E+01 |  |  |
| Medium white | IL5 - 2 | Untreated | 2 | Heat | 0 | 4 | 1.21E+02 | 11 | 3.33E+02 |
| Medium yellow | IL5 - 2 | Untreated | 2 | Heat | 0 | 4 | 1.21E+02 |  |  |
| Small white | IL5 - 2 | Untreated | 2 | Heat | 0 | 2 | 6.06E+01 |  |  |
| Medium melons | IL5 - 2 | Untreated | 2 | Heat | 0 | 1 | 3.03E+01 |  |  |
| Medium white | IL5 - 3 | Untreated | 2 | Direct | 0 | 9 | 2.73E+02 | 42 | 1.27E+03 |
| Small yellow | IL5 - 3 | Untreated | 2 | Direct | 0 | 11 | 3.33E+02 |  |  |
| Small melons | IL5 - 3 | Untreated | 2 | Direct | 0 | 4 | 1.21E+02 |  |  |
| Small white | IL5 - 3 | Untreated | 2 | Direct | 0 | 9 | 2.73E+02 |  |  |
| Medium oranges | IL5 - 3 | Untreated | 2 | Direct | 0 | 3 | 9.09E+01 |  |  |
| Medium melons | IL5 - 3 | Untreated | 2 | Direct | 0 | 6 | 1.82E+02 |  |  |
| Medium white | IL5 - 3 | Untreated | 2 | Heat | 0 | 1 | 3.03E+01 | 9 | 2.73E+02 |
| Pink | IL5 - 3 | Untreated | 2 | Heat | 0 | 1 | 3.03E+01 |  |  |
| Medium yellow | IL5 - 3 | Untreated | 2 | Heat | 0 | 2 | 6.06E+01 |  |  |
| Small yellow | IL5 - 3 | Untreated | 2 | Heat | 0 | 3 | 9.09E+01 |  |  |
| Small white | IL5 - 3 | Untreated | 2 | Heat | 0 | 1 | 3.03E+01 |  |  |
| Medium melons | IL5 - 3 | Untreated | 2 | Heat | 0 | 1 | 3.03E+01 |  |  |
| Medium white | IL8 - 1 | Untreated | 2 | Direct | 0 | 15 | 4.55E+02 | 63 | 1.91E+03 |
| Small white | IL8 - 1 | Untreated | 2 | Direct | 0 | 28 | 8.48E+02 |  |  |
| Small beige | IL8 - 1 | Untreated | 2 | Direct | 0 | 16 | 4.85E+02 |  |  |
| Light brown | IL8 - 1 | Untreated | 2 | Direct | 0 | 4 | 1.21E+02 |  |  |
| Medium white | IL8 - 2 | Untreated | 2 | Direct | 0 | 33 | 1.00E+03 | 62 | 1.88E+03 |
| Medium yellow | IL8 - 2 | Untreated | 2 | Direct | 0 | 2 | 6.06E+01 |  |  |
| Small melons | IL8 - 2 | Untreated | 2 | Direct | 0 | 6 | 1.82E+02 |  |  |
| Small white | IL8 - 2 | Untreated | 2 | Direct | 0 | 21 | 6.36E+02 |  |  |
| Medium white | IL8 - 3 | Untreated | 2 | Direct | 0 | 29 | 8.79E+02 | 67 | 2.03E+03 |
| Black | IL8 - 3 | Untreated | 2 | Direct | 0 | 1 | 3.03E+01 |  |  |
| Medium yellow | IL8 - 3 | Untreated | 2 | Direct | 0 | 1 | 3.03E+01 |  |  |
| Small yellow | IL8 - 3 | Untreated | 2 | Direct | 0 | 2 | 6.06E+01 |  |  |
| Small white | IL8 - 3 | Untreated | 2 | Direct | 0 | 33 | 1.00E+03 |  |  |
| Small translucent | IL8 - 3 | Untreated | 2 | Direct | 0 | 1 | 3.03E+01 |  |  |
| Medium white | IL8 - 3 | Untreated | 2 | Heat | 0 | 4 | 1.21E+02 | 8 | 2.42E+02 |
| Medium pink | IL8 - 3 | Untreated | 2 | Heat | 0 | 1 | 3.03E+01 |  |  |
| Pink | IL8 - 3 | Untreated | 2 | Heat | 0 | 1 | 3.03E+01 |  |  |
| Medium yellow | IL8 - 3 | Untreated | 2 | Heat | 0 | 1 | 3.03E+01 |  |  |
| Small white | IL8 - 3 | Untreated | 2 | Heat | 0 | 1 | 3.03E+01 |  |  |
| Medium yellow | IL4 - 1 | Heat | 2 | Direct | 2 | 94 | 2.85E+05 | 110 | 3.33E+05 |
| Medium white | IL4 - 1 | Heat | 2 | Direct | 2 | 14 | 4.24E+04 |  |  |
| Small white | IL4 - 1 | Heat | 2 | Direct | 2 | 2 | 6.06E+03 |  |  |
| Medium white | IL4 - 1 | Heat | 2 | Heat | 2 | 5 | 1.52E+04 | 40 | 1.21E+05 |
| Yellow cottony | IL4 - 1 | Heat | 2 | Heat | 2 | 35 | 1.06E+05 |  |  |
| Pink | IL4 - 2 | Heat | 2 | Direct | 2 | 2 | 6.06E+03 | 172 | 5.21E+05 |
| Small yellow | IL4 - 2 | Heat | 2 | Direct | 2 | 72 | 2.18E+05 |  |  |
| Medium white | IL4 - 2 | Heat | 2 | Direct | 2 | 88 | 2.67E+05 |  |  |
| Small white | IL4 - 2 | Heat | 2 | Direct | 2 | 8 | 2.42E+04 |  |  |
| Beige | IL4 - 2 | Heat | 2 | Direct | 2 | 2 | 6.06E+03 |  |  |
| Medium yellow | IL4 - 2 | Heat | 2 | Heat | 2 | 32 | 9.70E+04 | 76 | 2.30E+05 |
| Small melons | IL4 - 2 | Heat | 2 | Heat | 2 | 2 | 6.06E+03 |  |  |
| Medium white | IL4 - 2 | Heat | 2 | Heat | 2 | 35 | 1.06E+05 |  |  |
| Small white | IL4 - 2 | Heat | 2 | Heat | 2 | 4 | 1.21E+04 |  |  |
| Beige | IL4 - 2 | Heat | 2 | Heat | 2 | 3 | 9.09E+03 |  |  |
| Medium yellow | IL4 -3 | Heat | 2 | Direct | 2 | 90 | 2.73E+05 | 148 | 4.48E+05 |
| Small yellow | IL4 -3 | Heat | 2 | Direct | 2 | 22 | 6.67E+04 |  |  |
| Small white | IL4 -3 | Heat | 2 | Direct | 2 | 36 | 1.09E+05 |  |  |
| Pink | IL4 -3 | Heat | 2 | Heat | 2 | 2 | 6.06E+03 | 70 | 2.12E+05 |
| Small yellow | IL4 -3 | Heat | 2 | Heat | 2 | 52 | 1.58E+05 |  |  |
| Medium white | IL4 -3 | Heat | 2 | Heat | 2 | 14 | 4.24E+04 |  |  |
| Beige | IL4 -3 | Heat | 2 | Heat | 2 | 2 | 6.06E+03 |  |  |
| Pink | IL5 - 1 | Heat | 2 | Direct | 2 | 30 | 9.09E+04 | 87 | 2.64E+05 |
| Medium yellow | IL5 - 1 | Heat | 2 | Direct | 2 | 15 | 4.55E+04 |  |  |
| Small yellow | IL5 - 1 | Heat | 2 | Direct | 2 | 9 | 2.73E+04 |  |  |
| Small white | IL5 - 1 | Heat | 2 | Direct | 2 | 33 | 1.00E+05 |  |  |
| Pink | IL5 - 1 | Heat | 2 | Heat | 2 | 3 | 9.09E+03 | 26 | 7.88E+04 |
| Medium yellow | IL5 - 1 | Heat | 2 | Heat | 2 | 9 | 2.73E+04 |  |  |
| Small yellow | IL5 - 1 | Heat | 2 | Heat | 2 | 11 | 3.33E+04 |  |  |
| Small melons | IL5 - 1 | Heat | 2 | Heat | 2 | 1 | 3.03E+03 |  |  |
| Small white | IL5 - 1 | Heat | 2 | Heat | 2 | 2 | 6.06E+03 |  |  |
| Pink | IL5 - 2 | Heat | 2 | Direct | 2 | 18 | 5.45E+04 | 66 | 2.00E+05 |
| Medium yellow | IL5 - 2 | Heat | 2 | Direct | 2 | 4 | 1.21E+04 |  |  |
| Small yellow | IL5 - 2 | Heat | 2 | Direct | 2 | 23 | 6.97E+04 |  |  |
| Small melons | IL5 - 2 | Heat | 2 | Direct | 2 | 1 | 3.03E+03 |  |  |
| Small white | IL5 - 2 | Heat | 2 | Direct | 2 | 20 | 6.06E+04 |  |  |
| Pink | IL5 - 2 | Heat | 2 | Heat | 2 | 4 | 1.21E+04 | 21 | 6.36E+04 |
| Medium yellow | IL5 - 2 | Heat | 2 | Heat | 2 | 6 | 1.82E+04 |  |  |
| Small yellow | IL5 - 2 | Heat | 2 | Heat | 2 | 10 | 3.03E+04 |  |  |
| Small white | IL5 - 2 | Heat | 2 | Heat | 2 | 1 | 3.03E+03 |  |  |
| Pink | IL5 - 3 | Heat | 2 | Direct | 2 | 17 | 5.15E+04 | 44 | 1.33E+05 |
| Medium yellow | IL5 - 3 | Heat | 2 | Direct | 2 | 4 | 1.21E+04 |  |  |
| Small yellow | IL5 - 3 | Heat | 2 | Direct | 2 | 16 | 4.85E+04 |  |  |
| Small melons | IL5 - 3 | Heat | 2 | Direct | 2 | 1 | 3.03E+03 |  |  |
| Small white | IL5 - 3 | Heat | 2 | Direct | 2 | 4 | 1.21E+04 |  |  |
| Yellow cottony | IL5 - 3 | Heat | 2 | Direct | 2 | 2 | 6.06E+03 |  |  |
| Medium white | IL5 - 3 | Heat | 2 | Heat | 1 | 1 | 3.03E+02 | 75 | 2.27E+04 |
| Pink | IL5 - 3 | Heat | 2 | Heat | 1 | 20 | 6.06E+03 |  |  |
| Medium yellow | IL5 - 3 | Heat | 2 | Heat | 1 | 16 | 4.85E+03 |  |  |
| Small yellow | IL5 - 3 | Heat | 2 | Heat | 1 | 24 | 7.27E+03 |  |  |
| Small white | IL5 - 3 | Heat | 2 | Heat | 1 | 14 | 4.24E+03 |  |  |
| Small yellow | IL8 - 1 | Heat | 2 | Direct | 1 | 124 | 3.76E+04 | 176 | 5.33E+04 |
| Small white | IL8 - 1 | Heat | 2 | Direct | 1 | 52 | 1.58E+04 |  |  |
| Pink | IL8 - 1 | Heat | 2 | Heat | 1 | 3 | 9.09E+02 | 194 | 5.88E+04 |
| Medium yellow | IL8 - 1 | Heat | 2 | Heat | 1 | 2 | 6.06E+02 |  |  |
| Small yellow | IL8 - 1 | Heat | 2 | Heat | 1 | 148 | 4.48E+04 |  |  |
| Medium white | IL8 - 1 | Heat | 2 | Heat | 1 | 1 | 3.03E+02 |  |  |
| Small white | IL8 - 1 | Heat | 2 | Heat | 1 | 40 | 1.21E+04 |  |  |
| Small yellow | IL8 - 2 | Heat | 2 | Direct | 2 | 116 | 3.52E+05 | 144 | 4.36E+05 |
| Small white | IL8 - 2 | Heat | 2 | Direct | 2 | 28 | 8.48E+04 |  |  |
| Small yellow | IL8 - 2 | Heat | 2 | Heat | 2 | 136 | 4.12E+05 | 160 | 4.85E+05 |
| Small white | IL8 - 2 | Heat | 2 | Heat | 2 | 24 | 7.27E+04 |  |  |
| Pink | IL8 - 3 | Heat | 2 | Direct | 2 | 1 | 3.03E+03 | 74 | 2.24E+05 |
| Small yellow | IL8 - 3 | Heat | 2 | Direct | 2 | 65 | 1.97E+05 |  |  |
| Small white | IL8 - 3 | Heat | 2 | Direct | 2 | 8 | 2.42E+04 |  |  |
| Medium pink | IL8 - 3 | Heat | 2 | Heat | 2 | 1 | 3.03E+03 | 97 | 2.94E+05 |
| Pink | IL8 - 3 | Heat | 2 | Heat | 2 | 2 | 6.06E+03 |  |  |
| Small yellow | IL8 - 3 | Heat | 2 | Heat | 2 | 90 | 2.73E+05 |  |  |
| Small white | IL8 - 3 | Heat | 2 | Heat | 2 | 4 | 1.21E+04 |  |  |
| Small white | IL4 - 1 | Untreated | 3 | Direct | 0 | 7 | 2.12E+02 | 7 | 2.12E+02 |
| Small white | IL4 - 1 | Untreated | 3 | Heat | 0 | 1 | 3.03E+01 | 1 | 3.03E+01 |
| Small white | IL4 - 2 | Untreated | 3 | Direct | 0 | 21 | 6.36E+02 | 21 | 6.36E+02 |
| Small white | IL4 - 2 | Untreated | 3 | Heat | 0 | 2 | 6.06E+01 | 2 | 6.06E+01 |
| Small white | IL4 - 3 | Untreated | 3 | Direct | 2 | 1 | 3.03E+03 | 1 | 3.03E+03 |
| Medium white | IL4 - 3 | Untreated | 3 | Heat | 0 | 1 | 3.03E+01 | 2 | 6.06E+01 |
| Small white | IL4 - 3 | Untreated | 3 | Heat | 0 | 1 | 3.03E+01 |  |  |
| Small yellow | IL5 - 1 | Untreated | 3 | Direct | 0 | 6 | 1.82E+02 | 21 | 6.36E+02 |
| Medium white | IL5 - 1 | Untreated | 3 | Direct | 0 | 4 | 1.21E+02 |  |  |
| Small white | IL5 - 1 | Untreated | 3 | Direct | 0 | 11 | 3.33E+02 |  |  |
| Small yellow | IL5 - 1 | Untreated | 3 | Heat | 0 | 3 | 9.09E+01 | 7 | 2.12E+02 |
| Small white | IL5 - 1 | Untreated | 3 | Heat | 0 | 4 | 1.21E+02 |  |  |
| Medium white | IL5 - 2 | Untreated | 3 | Direct | 0 | 1 | 3.03E+01 | 16 | 4.85E+02 |
| Small yellow | IL5 - 2 | Untreated | 3 | Direct | 0 | 2 | 6.06E+01 |  |  |
| Small white | IL5 - 2 | Untreated | 3 | Direct | 0 | 13 | 3.94E+02 |  |  |
| Small yellow | IL5 - 2 | Untreated | 3 | Heat | 0 | 1 | 3.03E+01 | 2 | 6.06E+01 |
| Small white | IL5 - 2 | Untreated | 3 | Heat | 0 | 1 | 3.03E+01 |  |  |
| Medium white | IL5 - 3 | Untreated | 3 | Direct | 0 | 6 | 1.82E+02 | 20 | 6.06E+02 |
| Pink | IL5 - 3 | Untreated | 3 | Direct | 0 | 2 | 6.06E+01 |  |  |
| Small yellow | IL5 - 3 | Untreated | 3 | Direct | 0 | 1 | 3.03E+01 |  |  |
| Small white | IL5 - 3 | Untreated | 3 | Direct | 0 | 11 | 3.33E+02 |  |  |
| Medium white | IL5 - 3 | Untreated | 3 | Heat | 0 | 2 | 6.06E+01 | 33 | 1.00E+03 |
| Small yellow | IL5 - 3 | Untreated | 3 | Heat | 0 | 14 | 4.24E+02 |  |  |
| Small white | IL5 - 3 | Untreated | 3 | Heat | 0 | 17 | 5.15E+02 |  |  |
| Medium yellow | IL8 - 1 | Untreated | 3 | Direct | 0 | 2 | 6.06E+01 | 14 | 4.24E+02 |
| Medium white | IL8 - 1 | Untreated | 3 | Direct | 0 | 4 | 1.21E+02 |  |  |
| Small white | IL8 - 1 | Untreated | 3 | Direct | 0 | 4 | 1.21E+02 |  |  |
| Translucent yellow | IL8 - 1 | Untreated | 3 | Direct | 0 | 4 | 1.21E+02 |  |  |
| Medium white | IL8 - 1 | Untreated | 3 | Heat | 0 | 4 | 1.21E+02 | 10 | 3.03E+02 |
| Medium pink | IL8 - 1 | Untreated | 3 | Heat | 0 | 1 | 3.03E+01 |  |  |
| Pink | IL8 - 1 | Untreated | 3 | Heat | 0 | 1 | 3.03E+01 |  |  |
| Medium yellow | IL8 - 1 | Untreated | 3 | Heat | 0 | 2 | 6.06E+01 |  |  |
| Small melons | IL8 - 1 | Untreated | 3 | Heat | 0 | 2 | 6.06E+01 |  |  |
| Medium white | IL8 - 2 | Untreated | 3 | Direct | 0 | 19 | 5.76E+02 | 55 | 1.67E+03 |
| Small yellow | IL8 - 2 | Untreated | 3 | Direct | 0 | 4 | 1.21E+02 |  |  |
| Small white | IL8 - 2 | Untreated | 3 | Direct | 0 | 32 | 9.70E+02 |  |  |
| Medium white | IL8 - 2 | Untreated | 3 | Heat | 0 | 4 | 1.21E+02 | 8 | 2.42E+02 |
| Small white | IL8 - 2 | Untreated | 3 | Heat | 0 | 4 | 1.21E+02 |  |  |
| Medium white | IL8 - 3 | Untreated | 3 | Direct | 0 | 17 | 5.15E+02 | 43 | 1.30E+03 |
| Medium yellow | IL8 - 3 | Untreated | 3 | Direct | 0 | 3 | 9.09E+01 |  |  |
| Small yellow | IL8 - 3 | Untreated | 3 | Direct | 0 | 1 | 3.03E+01 |  |  |
| Small white | IL8 - 3 | Untreated | 3 | Direct | 0 | 15 | 4.55E+02 |  |  |
| Small translucent | IL8 - 3 | Untreated | 3 | Direct | 0 | 7 | 2.12E+02 |  |  |
| Medium pink | IL8 - 3 | Untreated | 3 | Heat | 0 | 2 | 6.06E+01 | 18 | 5.45E+02 |
| Small yellow | IL8 - 3 | Untreated | 3 | Heat | 0 | 4 | 1.21E+02 |  |  |
| Small melons | IL8 - 3 | Untreated | 3 | Heat | 0 | 4 | 1.21E+02 |  |  |
| Medium white | IL8 - 3 | Untreated | 3 | Heat | 0 | 5 | 1.52E+02 |  |  |
| Small white | IL8 - 3 | Untreated | 3 | Heat | 0 | 3 | 9.09E+01 |  |  |
| Pink | IL4 - 1 | Heat | 3 | Direct | 2 | 5 | 1.52E+04 | 70 | 2.12E+05 |
| Medium yellow | IL4 - 1 | Heat | 3 | Direct | 2 | 4 | 1.21E+04 |  |  |
| Small yellow | IL4 - 1 | Heat | 3 | Direct | 2 | 44 | 1.33E+05 |  |  |
| Small white | IL4 - 1 | Heat | 3 | Direct | 2 | 17 | 5.15E+04 |  |  |
| Pink | IL4 - 1 | Heat | 3 | Heat | 2 | 1 | 3.03E+03 | 58 | 1.76E+05 |
| Small yellow | IL4 - 1 | Heat | 3 | Heat | 2 | 49 | 1.48E+05 |  |  |
| Small melons | IL4 - 1 | Heat | 3 | Heat | 2 | 3 | 9.09E+03 |  |  |
| Small white | IL4 - 1 | Heat | 3 | Heat | 2 | 5 | 1.52E+04 |  |  |
| Pink | IL4 - 2 | Heat | 3 | Direct | 1 | 7 | 2.12E+03 | 170 | 5.15E+04 |
| Medium yellow | IL4 - 2 | Heat | 3 | Direct | 1 | 40 | 1.21E+04 |  |  |
| Small white | IL4 - 2 | Heat | 3 | Direct | 1 | 123 | 3.73E+04 |  |  |
| Medium white | IL4 - 2 | Heat | 3 | Heat | 0 | 1 | 3.03E+01 | 289 | 8.76E+03 |
| Small yellow | IL4 - 2 | Heat | 3 | Heat | 0 | 156 | 4.73E+03 |  |  |
| Small white | IL4 - 2 | Heat | 3 | Heat | 0 | 132 | 4.00E+03 |  |  |
| Medium yellow | IL4 -3 | Heat | 3 | Direct | 2 | 58 | 1.76E+05 | 79 | 2.39E+05 |
| Small white | IL4 -3 | Heat | 3 | Direct | 2 | 21 | 6.36E+04 |  |  |
| Small yellow | IL4 -3 | Heat | 3 | Heat | 1 | 125 | 3.79E+04 | 187 | 5.67E+04 |
| Small white | IL4 -3 | Heat | 3 | Heat | 1 | 62 | 1.88E+04 |  |  |
| Pink | IL5 - 1 | Heat | 3 | Direct | 2 | 56 | 1.70E+05 | 81 | 2.45E+05 |
| Medium yellow | IL5 - 1 | Heat | 3 | Direct | 2 | 5 | 1.52E+04 |  |  |
| Small white | IL5 - 1 | Heat | 3 | Direct | 2 | 20 | 6.06E+04 |  |  |
| Pink | IL5 - 1 | Heat | 3 | Heat | 2 | 32 | 9.70E+04 | 59 | 1.79E+05 |
| Medium yellow | IL5 - 1 | Heat | 3 | Heat | 2 | 5 | 1.52E+04 |  |  |
| Small yellow | IL5 - 1 | Heat | 3 | Heat | 2 | 4 | 1.21E+04 |  |  |
| Small white | IL5 - 1 | Heat | 3 | Heat | 2 | 18 | 5.45E+04 |  |  |
| Pink | IL5 - 2 | Heat | 3 | Direct | 1 | 180 | 5.45E+04 | 253 | 7.67E+04 |
| Small yellow | IL5 - 2 | Heat | 3 | Direct | 1 | 17 | 5.15E+03 |  |  |
| Small white | IL5 - 2 | Heat | 3 | Direct | 1 | 56 | 1.70E+04 |  |  |
| Pink | IL5 - 2 | Heat | 3 | Heat | 1 | 40 | 1.21E+04 | 52 | 1.58E+04 |
| Small yellow | IL5 - 2 | Heat | 3 | Heat | 1 | 8 | 2.42E+03 |  |  |
| Small white | IL5 - 2 | Heat | 3 | Heat | 1 | 4 | 1.21E+03 |  |  |
| Pink | IL5 - 3 | Heat | 3 | Direct | 2 | 69 | 2.09E+05 | 118 | 3.58E+05 |
| Small yellow | IL5 - 3 | Heat | 3 | Direct | 2 | 18 | 5.45E+04 |  |  |
| Small melons | IL5 - 3 | Heat | 3 | Direct | 2 | 2 | 6.06E+03 |  |  |
| Small white | IL5 - 3 | Heat | 3 | Direct | 2 | 29 | 8.79E+04 |  |  |
| Pink | IL5 - 3 | Heat | 3 | Heat | 1 | 40 | 1.21E+04 | 132 | 4.00E+04 |
| Medium yellow | IL5 - 3 | Heat | 3 | Heat | 1 | 8 | 2.42E+03 |  |  |
| Small yellow | IL5 - 3 | Heat | 3 | Heat | 1 | 20 | 6.06E+03 |  |  |
| Small white | IL5 - 3 | Heat | 3 | Heat | 1 | 64 | 1.94E+04 |  |  |
| Small yellow | IL8 - 1 | Heat | 3 | Direct | 1 | 58 | 1.76E+04 | 138 | 4.18E+04 |
| Small melons | IL8 - 1 | Heat | 3 | Direct | 1 | 4 | 1.21E+03 |  |  |
| Small white | IL8 - 1 | Heat | 3 | Direct | 1 | 76 | 2.30E+04 |  |  |
| Small yellow | IL8 - 1 | Heat | 3 | Heat | 1 | 156 | 4.73E+04 | 228 | 6.91E+04 |
| Small white | IL8 - 1 | Heat | 3 | Heat | 1 | 72 | 2.18E+04 |  |  |
| Pink | IL8 - 2 | Heat | 3 | Direct | 1 | 2 | 6.06E+02 | 278 | 8.42E+04 |
| Small yellow | IL8 - 2 | Heat | 3 | Direct | 1 | 228 | 6.91E+04 |  |  |
| Small white | IL8 - 2 | Heat | 3 | Direct | 1 | 48 | 1.45E+04 |  |  |
| Small yellow | IL8 - 2 | Heat | 3 | Heat | 1 | 156 | 4.73E+04 | 228 | 6.91E+04 |
| Small white | IL8 - 2 | Heat | 3 | Heat | 1 | 72 | 2.18E+04 |  |  |
| Small yellow | IL8 - 3 | Heat | 3 | Direct | 1 | 16 | 4.85E+03 | 136 | 4.12E+04 |
| Small white | IL8 - 3 | Heat | 3 | Direct | 1 | 120 | 3.64E+04 |  |  |
| Pink | IL8 - 3 | Heat | 3 | Heat | 1 | 2 | 6.06E+02 | 160 | 4.85E+04 |
| Small yellow | IL8 - 3 | Heat | 3 | Heat | 1 | 12 | 3.64E+03 |  |  |
| Small melons | IL8 - 3 | Heat | 3 | Heat | 1 | 8 | 2.42E+03 |  |  |
| Small white | IL8 - 3 | Heat | 3 | Heat | 1 | 138 | 4.18E+04 |  |  |
| Medium white | IL4 - 1 | Untreated | 4 | Direct | 0 | 1 | 3.03E+01 | 38 | 1.15E+03 |
| Small yellow | IL4 - 1 | Untreated | 4 | Direct | 0 | 2 | 6.06E+01 |  |  |
| Small white | IL4 - 1 | Untreated | 4 | Direct | 0 | 7 | 2.12E+02 |  |  |
| Small translucent | IL4 - 1 | Untreated | 4 | Direct | 0 | 28 | 8.48E+02 |  |  |
| Medium white | IL4 - 1 | Untreated | 4 | Heat | 0 | 4 | 1.21E+02 | 21 | 6.36E+02 |
| Medium yellow | IL4 - 1 | Untreated | 4 | Heat | 0 | 10 | 3.03E+02 |  |  |
| Small white | IL4 - 1 | Untreated | 4 | Heat | 0 | 4 | 1.21E+02 |  |  |
| Small translucent | IL4 - 1 | Untreated | 4 | Heat | 0 | 3 | 9.09E+01 |  |  |
| Medium yellow | IL4 - 2 | Untreated | 4 | Direct | 0 | 1 | 3.03E+01 | 186 | 5.64E+03 |
| Small yellow | IL4 - 2 | Untreated | 4 | Direct | 0 | 1 | 3.03E+01 |  |  |
| Small white | IL4 - 2 | Untreated | 4 | Direct | 0 | 8 | 2.42E+02 |  |  |
| Small translucent | IL4 - 2 | Untreated | 4 | Direct | 0 | 176 | 5.33E+03 |  |  |
| Medium white | IL4 - 2 | Untreated | 4 | Heat | 0 | 1 | 3.03E+01 | 10 | 3.03E+02 |
| Medium translucent | IL4 - 2 | Untreated | 4 | Heat | 0 | 1 | 3.03E+01 |  |  |
| Small translucent | IL4 - 2 | Untreated | 4 | Heat | 0 | 8 | 2.42E+02 |  |  |
| Medium yellow | IL4 - 3 | Untreated | 4 | Direct | 0 | 4 | 1.21E+02 | 52 | 1.58E+03 |
| Medium white | IL4 - 3 | Untreated | 4 | Direct | 0 | 1 | 3.03E+01 |  |  |
| Small white | IL4 - 3 | Untreated | 4 | Direct | 0 | 20 | 6.06E+02 |  |  |
| Medium translucent | IL4 - 3 | Untreated | 4 | Direct | 0 | 1 | 3.03E+01 |  |  |
| Small translucent | IL4 - 3 | Untreated | 4 | Direct | 0 | 19 | 5.76E+02 |  |  |
| Translucent yellow | IL4 - 3 | Untreated | 4 | Direct | 0 | 7 | 2.12E+02 |  |  |
| Medium white | IL4 - 3 | Untreated | 4 | Heat | 0 | 2 | 6.06E+01 | 2 | 6.06E+01 |
| Medium white | IL5 - 1 | Untreated | 4 | Direct | 0 | 8 | 2.42E+02 | 91 | 2.76E+03 |
| Medium yellow | IL5 - 1 | Untreated | 4 | Direct | 0 | 4 | 1.21E+02 |  |  |
| Small melons | IL5 - 1 | Untreated | 4 | Direct | 0 | 2 | 6.06E+01 |  |  |
| Small white | IL5 - 1 | Untreated | 4 | Direct | 0 | 74 | 2.24E+03 |  |  |
| Translucent yellow | IL5 - 1 | Untreated | 4 | Direct | 0 | 3 | 9.09E+01 |  |  |
| Medium white | IL5 - 1 | Untreated | 4 | Heat | 0 | 3 | 9.09E+01 | 25 | 7.58E+02 |
| Medium pink | IL5 - 1 | Untreated | 4 | Heat | 0 | 1 | 3.03E+01 |  |  |
| Pink | IL5 - 1 | Untreated | 4 | Heat | 0 | 1 | 3.03E+01 |  |  |
| Medium yellow | IL5 - 1 | Untreated | 4 | Heat | 0 | 4 | 1.21E+02 |  |  |
| Small yellow | IL5 - 1 | Untreated | 4 | Heat | 0 | 2 | 6.06E+01 |  |  |
| Small white | IL5 - 1 | Untreated | 4 | Heat | 0 | 7 | 2.12E+02 |  |  |
| Small translucent | IL5 - 1 | Untreated | 4 | Heat | 0 | 7 | 2.12E+02 |  |  |
| Medium white | IL5 - 2 | Untreated | 4 | Direct | 0 | 3 | 9.09E+01 | 281 | 8.52E+03 |
| Medium pink | IL5 - 2 | Untreated | 4 | Direct | 0 | 2 | 6.06E+01 |  |  |
| Pink | IL5 - 2 | Untreated | 4 | Direct | 0 | 1 | 3.03E+01 |  |  |
| Small yellow | IL5 - 2 | Untreated | 4 | Direct | 0 | 7 | 2.12E+02 |  |  |
| Small melons | IL5 - 2 | Untreated | 4 | Direct | 0 | 3 | 9.09E+01 |  |  |
| Small white | IL5 - 2 | Untreated | 4 | Direct | 0 | 246 | 7.45E+03 |  |  |
| Small translucent | IL5 - 2 | Untreated | 4 | Direct | 0 | 19 | 5.76E+02 |  |  |
| Medium pink | IL5 - 2 | Untreated | 4 | Heat | 0 | 1 | 3.03E+01 | 27 | 8.18E+02 |
| Medium yellow | IL5 - 2 | Untreated | 4 | Heat | 0 | 6 | 1.82E+02 |  |  |
| Small yellow | IL5 - 2 | Untreated | 4 | Heat | 0 | 8 | 2.42E+02 |  |  |
| Small white | IL5 - 2 | Untreated | 4 | Heat | 0 | 5 | 1.52E+02 |  |  |
| Small translucent | IL5 - 2 | Untreated | 4 | Heat | 0 | 4 | 1.21E+02 |  |  |
| Translucent yellow | IL5 - 2 | Untreated | 4 | Heat | 0 | 3 | 9.09E+01 |  |  |
| Medium white | IL5 - 3 | Untreated | 4 | Direct | 0 | 78 | 2.36E+03 | 123 | 3.73E+03 |
| Medium yellow | IL5 - 3 | Untreated | 4 | Direct | 0 | 4 | 1.21E+02 |  |  |
| Small yellow | IL5 - 3 | Untreated | 4 | Direct | 0 | 9 | 2.73E+02 |  |  |
| Small white | IL5 - 3 | Untreated | 4 | Direct | 0 | 27 | 8.18E+02 |  |  |
| Small translucent | IL5 - 3 | Untreated | 4 | Direct | 0 | 5 | 1.52E+02 |  |  |
| Medium white | IL5 - 3 | Untreated | 4 | Heat | 0 | 3 | 9.09E+01 | 29 | 8.79E+02 |
| Medium pink | IL5 - 3 | Untreated | 4 | Heat | 0 | 2 | 6.06E+01 |  |  |
| Pink | IL5 - 3 | Untreated | 4 | Heat | 0 | 2 | 6.06E+01 |  |  |
| Medium yellow | IL5 - 3 | Untreated | 4 | Heat | 0 | 4 | 1.21E+02 |  |  |
| Small yellow | IL5 - 3 | Untreated | 4 | Heat | 0 | 7 | 2.12E+02 |  |  |
| Small white | IL5 - 3 | Untreated | 4 | Heat | 0 | 7 | 2.12E+02 |  |  |
| Small translucent | IL5 - 3 | Untreated | 4 | Heat | 0 | 1 | 3.03E+01 |  |  |
| Translucent yellow | IL5 - 3 | Untreated | 4 | Heat | 0 | 3 | 9.09E+01 |  |  |
| Medium yellow | IL8 - 1 | Untreated | 4 | Direct | 0 | 5 | 1.52E+02 | 88 | 2.67E+03 |
| Small yellow | IL8 - 1 | Untreated | 4 | Direct | 0 | 3 | 9.09E+01 |  |  |
| Small melons | IL8 - 1 | Untreated | 4 | Direct | 0 | 1 | 3.03E+01 |  |  |
| Medium white | IL8 - 1 | Untreated | 4 | Direct | 0 | 3 | 9.09E+01 |  |  |
| Small white | IL8 - 1 | Untreated | 4 | Direct | 0 | 76 | 2.30E+03 |  |  |
| Medium pink | IL8 - 1 | Untreated | 4 | Heat | 0 | 3 | 9.09E+01 | 17 | 5.15E+02 |
| Small yellow | IL8 - 1 | Untreated | 4 | Heat | 0 | 1 | 3.03E+01 |  |  |
| Medium white | IL8 - 1 | Untreated | 4 | Heat | 0 | 3 | 9.09E+01 |  |  |
| Small white | IL8 - 1 | Untreated | 4 | Heat | 0 | 10 | 3.03E+02 |  |  |
| Medium yellow | IL8 - 2 | Untreated | 4 | Direct | 0 | 4 | 1.21E+02 | 64 | 1.94E+03 |
| Medium white | IL8 - 2 | Untreated | 4 | Direct | 0 | 2 | 6.06E+01 |  |  |
| Small white | IL8 - 2 | Untreated | 4 | Direct | 0 | 47 | 1.42E+03 |  |  |
| Small translucent | IL8 - 2 | Untreated | 4 | Direct | 0 | 9 | 2.73E+02 |  |  |
| Translucent yellow | IL8 - 2 | Untreated | 4 | Direct | 0 | 2 | 6.06E+01 |  |  |
| Pink | IL8 - 2 | Untreated | 4 | Heat | 0 | 1 | 3.03E+01 | 3 | 9.09E+01 |
| Small white | IL8 - 2 | Untreated | 4 | Heat | 0 | 2 | 6.06E+01 |  |  |
| Pink | IL8 - 3 | Untreated | 4 | Direct | 0 | 2 | 6.06E+01 | 48 | 1.45E+03 |
| Medium yellow | IL8 - 3 | Untreated | 4 | Direct | 0 | 2 | 6.06E+01 |  |  |
| Small white | IL8 - 3 | Untreated | 4 | Direct | 0 | 44 | 1.33E+03 |  |  |
| Medium pink | IL8 - 3 | Untreated | 4 | Heat | 0 | 6 | 1.82E+02 | 97 | 2.94E+03 |
| Medium yellow | IL8 - 3 | Untreated | 4 | Heat | 0 | 1 | 3.03E+01 |  |  |
| Medium white | IL8 - 3 | Untreated | 4 | Heat | 0 | 1 | 3.03E+01 |  |  |
| Small white | IL8 - 3 | Untreated | 4 | Heat | 0 | 87 | 2.64E+03 |  |  |
| Translucent yellow | IL8 - 3 | Untreated | 4 | Heat | 0 | 2 | 6.06E+01 |  |  |
| Medium yellow | IL4 - 1 | Heat | 4 | Direct | 3 | 10 | 3.03E+05 | 18 | 5.45E+05 |
| Medium white | IL4 - 1 | Heat | 4 | Direct | 3 | 3 | 9.09E+04 |  |  |
| Small white | IL4 - 1 | Heat | 4 | Direct | 3 | 5 | 1.52E+05 |  |  |
| Medium yellow | IL4 - 1 | Heat | 4 | Heat | 3 | 13 | 3.94E+05 | 22 | 6.67E+05 |
| Medium white | IL4 - 1 | Heat | 4 | Heat | 3 | 3 | 9.09E+04 |  |  |
| Small white | IL4 - 1 | Heat | 4 | Heat | 3 | 5 | 1.52E+05 |  |  |
| Yellow cottony | IL4 - 1 | Heat | 4 | Heat | 3 | 1 | 3.03E+04 |  |  |
| Pink | IL4 - 2 | Heat | 4 | Direct | 2 | 2 | 6.06E+03 | 169 | 5.12E+05 |
| Medium yellow | IL4 - 2 | Heat | 4 | Direct | 2 | 3 | 9.09E+03 |  |  |
| Small yellow | IL4 - 2 | Heat | 4 | Direct | 2 | 96 | 2.91E+05 |  |  |
| Medium white | IL4 - 2 | Heat | 4 | Direct | 2 | 7 | 2.12E+04 |  |  |
| Small white | IL4 - 2 | Heat | 4 | Direct | 2 | 60 | 1.82E+05 |  |  |
| Small translucent | IL4 - 2 | Heat | 4 | Direct | 2 | 1 | 3.03E+03 |  |  |
| Pink | IL4 - 2 | Heat | 4 | Heat | 2 | 2 | 6.06E+03 | 173 | 5.24E+05 |
| Medium yellow | IL4 - 2 | Heat | 4 | Heat | 2 | 71 | 2.15E+05 |  |  |
| Small melons | IL4 - 2 | Heat | 4 | Heat | 2 | 2 | 6.06E+03 |  |  |
| Medium white | IL4 - 2 | Heat | 4 | Heat | 2 | 18 | 5.45E+04 |  |  |
| Small white | IL4 - 2 | Heat | 4 | Heat | 2 | 74 | 2.24E+05 |  |  |
| Small translucent | IL4 - 2 | Heat | 4 | Heat | 2 | 4 | 1.21E+04 |  |  |
| Yellow cottony | IL4 - 2 | Heat | 4 | Heat | 2 | 2 | 6.06E+03 |  |  |
| Pink | IL4 -3 | Heat | 4 | Direct | 2 | 5 | 1.52E+04 | 154 | 4.67E+05 |
| Medium yellow | IL4 -3 | Heat | 4 | Direct | 2 | 88 | 2.67E+05 |  |  |
| Medium white | IL4 -3 | Heat | 4 | Direct | 2 | 8 | 2.42E+04 |  |  |
| Small white | IL4 -3 | Heat | 4 | Direct | 2 | 49 | 1.48E+05 |  |  |
| Small translucent | IL4 -3 | Heat | 4 | Direct | 2 | 4 | 1.21E+04 |  |  |
| Pink | IL4 -3 | Heat | 4 | Heat | 2 | 4 | 1.21E+04 | 128 | 3.88E+05 |
| Medium yellow | IL4 -3 | Heat | 4 | Heat | 2 | 86 | 2.61E+05 |  |  |
| Small yellow | IL4 -3 | Heat | 4 | Heat | 2 | 5 | 1.52E+04 |  |  |
| Medium white | IL4 -3 | Heat | 4 | Heat | 2 | 19 | 5.76E+04 |  |  |
| Small white | IL4 -3 | Heat | 4 | Heat | 2 | 11 | 3.33E+04 |  |  |
| Small translucent | IL4 -3 | Heat | 4 | Heat | 2 | 3 | 9.09E+03 |  |  |
| Pink | IL5 - 1 | Heat | 4 | Direct | 3 | 12 | 3.64E+05 | 55 | 1.67E+06 |
| Medium yellow | IL5 - 1 | Heat | 4 | Direct | 3 | 19 | 5.76E+05 |  |  |
| Medium white | IL5 - 1 | Heat | 4 | Direct | 3 | 16 | 4.85E+05 |  |  |
| Small translucent | IL5 - 1 | Heat | 4 | Direct | 3 | 4 | 1.21E+05 |  |  |
| Translucent yellow | IL5 - 1 | Heat | 4 | Direct | 3 | 3 | 9.09E+04 |  |  |
| Yellow cottony | IL5 - 1 | Heat | 4 | Direct | 3 | 1 | 3.03E+04 |  |  |
| Pink | IL5 - 1 | Heat | 4 | Heat | 2 | 33 | 1.00E+05 | 104 | 3.15E+05 |
| Medium yellow | IL5 - 1 | Heat | 4 | Heat | 2 | 4 | 1.21E+04 |  |  |
| Small yellow | IL5 - 1 | Heat | 4 | Heat | 2 | 45 | 1.36E+05 |  |  |
| Small white | IL5 - 1 | Heat | 4 | Heat | 2 | 22 | 6.67E+04 |  |  |
| Pink | IL5 - 2 | Heat | 4 | Direct | 2 | 63 | 1.91E+05 | 152 | 4.61E+05 |
| Small yellow | IL5 - 2 | Heat | 4 | Direct | 2 | 47 | 1.42E+05 |  |  |
| Medium white | IL5 - 2 | Heat | 4 | Direct | 2 | 37 | 1.12E+05 |  |  |
| Small translucent | IL5 - 2 | Heat | 4 | Direct | 2 | 5 | 1.52E+04 |  |  |
| Pink | IL5 - 2 | Heat | 4 | Heat | 2 | 40 | 1.21E+05 | 121 | 3.67E+05 |
| Medium yellow | IL5 - 2 | Heat | 4 | Heat | 2 | 7 | 2.12E+04 |  |  |
| Small yellow | IL5 - 2 | Heat | 4 | Heat | 2 | 63 | 1.91E+05 |  |  |
| Small white | IL5 - 2 | Heat | 4 | Heat | 2 | 9 | 2.73E+04 |  |  |
| Yellow cottony | IL5 - 2 | Heat | 4 | Heat | 2 | 2 | 6.06E+03 |  |  |
| Pink | IL5 - 3 | Heat | 4 | Direct | 2 | 89 | 2.70E+05 | 162 | 4.91E+05 |
| Medium yellow | IL5 - 3 | Heat | 4 | Direct | 2 | 9 | 2.73E+04 |  |  |
| Small yellow | IL5 - 3 | Heat | 4 | Direct | 2 | 25 | 7.58E+04 |  |  |
| Small white | IL5 - 3 | Heat | 4 | Direct | 2 | 36 | 1.09E+05 |  |  |
| Translucent yellow | IL5 - 3 | Heat | 4 | Direct | 2 | 3 | 9.09E+03 |  |  |
| Pink | IL5 - 3 | Heat | 4 | Heat | 2 | 90 | 2.73E+05 | 230 | 6.97E+05 |
| Medium yellow | IL5 - 3 | Heat | 4 | Heat | 2 | 18 | 5.45E+04 |  |  |
| Small yellow | IL5 - 3 | Heat | 4 | Heat | 2 | 45 | 1.36E+05 |  |  |
| Medium white | IL5 - 3 | Heat | 4 | Heat | 2 | 58 | 1.76E+05 |  |  |
| Small white | IL5 - 3 | Heat | 4 | Heat | 2 | 19 | 5.76E+04 |  |  |
| Medium white | IL8 - 1 | Heat | 4 | Direct | 2 | 30 | 9.09E+04 | 155 | 4.70E+05 |
| Pink | IL8 - 1 | Heat | 4 | Direct | 2 | 1 | 3.03E+03 |  |  |
| Medium yellow | IL8 - 1 | Heat | 4 | Direct | 2 | 113 | 3.42E+05 |  |  |
| Small yellow | IL8 - 1 | Heat | 4 | Direct | 2 | 1 | 3.03E+03 |  |  |
| Small white | IL8 - 1 | Heat | 4 | Direct | 2 | 3 | 9.09E+03 |  |  |
| Translucent yellow | IL8 - 1 | Heat | 4 | Direct | 2 | 3 | 9.09E+03 |  |  |
| Yellow cottony | IL8 - 1 | Heat | 4 | Direct | 2 | 4 | 1.21E+04 |  |  |
| Medium white | IL8 - 1 | Heat | 4 | Heat | 2 | 19 | 5.76E+04 | 126 | 3.82E+05 |
| Pink | IL8 - 1 | Heat | 4 | Heat | 2 | 4 | 1.21E+04 |  |  |
| Medium yellow | IL8 - 1 | Heat | 4 | Heat | 2 | 98 | 2.97E+05 |  |  |
| Translucent yellow | IL8 - 1 | Heat | 4 | Heat | 2 | 3 | 9.09E+03 |  |  |
| Yellow cottony | IL8 - 1 | Heat | 4 | Heat | 2 | 2 | 6.06E+03 |  |  |
| Medium white | IL8 - 2 | Heat | 4 | Direct | 2 | 29 | 8.79E+04 | 122 | 3.70E+05 |
| Medium yellow | IL8 - 2 | Heat | 4 | Direct | 2 | 59 | 1.79E+05 |  |  |
| Small yellow | IL8 - 2 | Heat | 4 | Direct | 2 | 18 | 5.45E+04 |  |  |
| Small white | IL8 - 2 | Heat | 4 | Direct | 2 | 8 | 2.42E+04 |  |  |
| Small translucent | IL8 - 2 | Heat | 4 | Direct | 2 | 3 | 9.09E+03 |  |  |
| Translucent yellow | IL8 - 2 | Heat | 4 | Direct | 2 | 5 | 1.52E+04 |  |  |
| Medium white | IL8 - 2 | Heat | 4 | Heat | 2 | 29 | 8.79E+04 | 119 | 3.61E+05 |
| Pink | IL8 - 2 | Heat | 4 | Heat | 2 | 1 | 3.03E+03 |  |  |
| Medium yellow | IL8 - 2 | Heat | 4 | Heat | 2 | 83 | 2.52E+05 |  |  |
| Small white | IL8 - 2 | Heat | 4 | Heat | 2 | 3 | 9.09E+03 |  |  |
| Small translucent | IL8 - 2 | Heat | 4 | Heat | 2 | 1 | 3.03E+03 |  |  |
| Translucent yellow | IL8 - 2 | Heat | 4 | Heat | 2 | 2 | 6.06E+03 |  |  |
| Medium white | IL8 - 3 | Heat | 4 | Direct | 2 | 7 | 2.12E+04 | 193 | 5.85E+05 |
| Pink | IL8 - 3 | Heat | 4 | Direct | 2 | 4 | 1.21E+04 |  |  |
| Medium yellow | IL8 - 3 | Heat | 4 | Direct | 2 | 136 | 4.12E+05 |  |  |
| Small yellow | IL8 - 3 | Heat | 4 | Direct | 2 | 2 | 6.06E+03 |  |  |
| Small white | IL8 - 3 | Heat | 4 | Direct | 2 | 44 | 1.33E+05 |  |  |
| Medium white | IL8 - 3 | Heat | 4 | Heat | 2 | 12 | 3.64E+04 | 74 | 2.24E+05 |
| Pink | IL8 - 3 | Heat | 4 | Heat | 2 | 2 | 6.06E+03 |  |  |
| Medium yellow | IL8 - 3 | Heat | 4 | Heat | 2 | 42 | 1.27E+05 |  |  |
| Small yellow | IL8 - 3 | Heat | 4 | Heat | 2 | 6 | 1.82E+04 |  |  |
| Small white | IL8 - 3 | Heat | 4 | Heat | 2 | 6 | 1.82E+04 |  |  |
| Translucent yellow | IL8 - 3 | Heat | 4 | Heat | 2 | 6 | 1.82E+04 |  |  |
| Medium yellow | IL4 - 1 | Untreated | 5 | Direct | 0 | 1 | 3.03E+01 | 10 | 3.03E+02 |
| Small yellow | IL4 - 1 | Untreated | 5 | Direct | 0 | 1 | 3.03E+01 |  |  |
| Small white | IL4 - 1 | Untreated | 5 | Direct | 0 | 8 | 2.42E+02 |  |  |
| Medium pink | IL4 - 1 | Untreated | 5 | Heat | 0 | 1 | 3.03E+01 | 48 | 1.45E+03 |
| Medium yellow | IL4 - 1 | Untreated | 5 | Heat | 0 | 45 | 1.36E+03 |  |  |
| Small yellow | IL4 - 1 | Untreated | 5 | Heat | 0 | 2 | 6.06E+01 |  |  |
| Medium yellow | IL4 - 2 | Untreated | 5 | Direct | 0 | 2 | 6.06E+01 | 34 | 1.03E+03 |
| Small yellow | IL4 - 2 | Untreated | 5 | Direct | 0 | 1 | 3.03E+01 |  |  |
| Medium white | IL4 - 2 | Untreated | 5 | Direct | 0 | 9 | 2.73E+02 |  |  |
| Small white | IL4 - 2 | Untreated | 5 | Direct | 0 | 8 | 2.42E+02 |  |  |
| Small translucent | IL4 - 2 | Untreated | 5 | Direct | 0 | 14 | 4.24E+02 |  |  |
| Medium pink | IL4 - 2 | Untreated | 5 | Heat | 0 | 1 | 3.03E+01 | 1 | 3.03E+01 |
| Medium yellow | IL4 - 3 | Untreated | 5 | Direct | 0 | 1 | 3.03E+01 | 21 | 6.36E+02 |
| Small yellow | IL4 - 3 | Untreated | 5 | Direct | 0 | 3 | 9.09E+01 |  |  |
| Small translucent | IL4 - 3 | Untreated | 5 | Direct | 0 | 14 | 4.24E+02 |  |  |
| Translucent yellow | IL4 - 3 | Untreated | 5 | Direct | 0 | 3 | 9.09E+01 |  |  |
| Medium white | IL4 - 3 | Untreated | 5 | Heat | 0 | 2 | 6.06E+01 | 2 | 6.06E+01 |
| Medium white | IL5 - 1 | Untreated | 5 | Direct | 0 | 18 | 5.45E+02 | 125 | 3.79E+03 |
| Pink | IL5 - 1 | Untreated | 5 | Direct | 0 | 1 | 3.03E+01 |  |  |
| Medium yellow | IL5 - 1 | Untreated | 5 | Direct | 0 | 12 | 3.64E+02 |  |  |
| Small yellow | IL5 - 1 | Untreated | 5 | Direct | 0 | 10 | 3.03E+02 |  |  |
| Small white | IL5 - 1 | Untreated | 5 | Direct | 0 | 13 | 3.94E+02 |  |  |
| Small translucent | IL5 - 1 | Untreated | 5 | Direct | 0 | 71 | 2.15E+03 |  |  |
| Medium yellow | IL5 - 1 | Untreated | 5 | Heat | 0 | 4 | 1.21E+02 | 26 | 7.88E+02 |
| Medium white | IL5 - 1 | Untreated | 5 | Heat | 0 | 7 | 2.12E+02 |  |  |
| Small white | IL5 - 1 | Untreated | 5 | Heat | 0 | 15 | 4.55E+02 |  |  |
| Medium yellow | IL5 - 2 | Untreated | 5 | Direct | 0 | 6 | 1.82E+02 | 156 | 4.73E+03 |
| Small yellow | IL5 - 2 | Untreated | 5 | Direct | 0 | 28 | 8.48E+02 |  |  |
| Medium white | IL5 - 2 | Untreated | 5 | Direct | 0 | 7 | 2.12E+02 |  |  |
| Small white | IL5 - 2 | Untreated | 5 | Direct | 0 | 32 | 9.70E+02 |  |  |
| Small translucent | IL5 - 2 | Untreated | 5 | Direct | 0 | 83 | 2.52E+03 |  |  |
| Medium white | IL5 - 2 | Untreated | 5 | Heat | 0 | 15 | 4.55E+02 | 41 | 1.24E+03 |
| Pink | IL5 - 2 | Untreated | 5 | Heat | 0 | 2 | 6.06E+01 |  |  |
| Medium yellow | IL5 - 2 | Untreated | 5 | Heat | 0 | 7 | 2.12E+02 |  |  |
| Small white | IL5 - 2 | Untreated | 5 | Heat | 0 | 17 | 5.15E+02 |  |  |
| Medium yellow | IL5 - 3 | Untreated | 5 | Direct | 1 | 2 | 6.06E+02 | 7 | 2.12E+03 |
| Small yellow | IL5 - 3 | Untreated | 5 | Direct | 1 | 2 | 6.06E+02 |  |  |
| Medium white | IL5 - 3 | Untreated | 5 | Direct | 1 | 1 | 3.03E+02 |  |  |
| Small white | IL5 - 3 | Untreated | 5 | Direct | 1 | 2 | 6.06E+02 |  |  |
| Medium white | IL5 - 3 | Untreated | 5 | Heat | 0 | 12 | 3.64E+02 | 46 | 1.39E+03 |
| Medium pink | IL5 - 3 | Untreated | 5 | Heat | 0 | 2 | 6.06E+01 |  |  |
| Pink | IL5 - 3 | Untreated | 5 | Heat | 0 | 1 | 3.03E+01 |  |  |
| Medium yellow | IL5 - 3 | Untreated | 5 | Heat | 0 | 12 | 3.64E+02 |  |  |
| Small yellow | IL5 - 3 | Untreated | 5 | Heat | 0 | 5 | 1.52E+02 |  |  |
| Small melons | IL5 - 3 | Untreated | 5 | Heat | 0 | 1 | 3.03E+01 |  |  |
| Small white | IL5 - 3 | Untreated | 5 | Heat | 0 | 13 | 3.94E+02 |  |  |
| Medium white | IL8 - 1 | Untreated | 5 | Direct | 0 | 2 | 6.06E+01 | 92 | 2.79E+03 |
| Medium pink | IL8 - 1 | Untreated | 5 | Direct | 0 | 2 | 6.06E+01 |  |  |
| Pink | IL8 - 1 | Untreated | 5 | Direct | 0 | 1 | 3.03E+01 |  |  |
| Small white | IL8 - 1 | Untreated | 5 | Direct | 0 | 37 | 1.12E+03 |  |  |
| Small translucent | IL8 - 1 | Untreated | 5 | Direct | 0 | 50 | 1.52E+03 |  |  |
| Medium pink | IL8 - 1 | Untreated | 5 | Heat | 0 | 3 | 9.09E+01 | 16 | 4.85E+02 |
| Medium yellow | IL8 - 1 | Untreated | 5 | Heat | 0 | 5 | 1.52E+02 |  |  |
| Small melons | IL8 - 1 | Untreated | 5 | Heat | 0 | 1 | 3.03E+01 |  |  |
| Medium white | IL8 - 1 | Untreated | 5 | Heat | 0 | 4 | 1.21E+02 |  |  |
| Small white | IL8 - 1 | Untreated | 5 | Heat | 0 | 2 | 6.06E+01 |  |  |
| Medium translucent | IL8 - 1 | Untreated | 5 | Heat | 0 | 1 | 3.03E+01 |  |  |
| Medium pink | IL8 - 2 | Untreated | 5 | Direct | 0 | 4 | 1.21E+02 | 103 | 3.12E+03 |
| Small white | IL8 - 2 | Untreated | 5 | Direct | 0 | 69 | 2.09E+03 |  |  |
| Small translucent | IL8 - 2 | Untreated | 5 | Direct | 0 | 30 | 9.09E+02 |  |  |
| Medium white | IL8 - 2 | Untreated | 5 | Heat | 0 | 16 | 4.85E+02 | 33 | 1.00E+03 |
| Medium pink | IL8 - 2 | Untreated | 5 | Heat | 0 | 4 | 1.21E+02 |  |  |
| Medium yellow | IL8 - 2 | Untreated | 5 | Heat | 0 | 10 | 3.03E+02 |  |  |
| Small melons | IL8 - 2 | Untreated | 5 | Heat | 0 | 1 | 3.03E+01 |  |  |
| Medium translucent | IL8 - 2 | Untreated | 5 | Heat | 0 | 1 | 3.03E+01 |  |  |
| Medium oranges | IL8 - 2 | Untreated | 5 | Heat | 0 | 1 | 3.03E+01 |  |  |
| Medium white | IL8 - 3 | Untreated | 5 | Direct | 0 | 2 | 6.06E+01 | 153 | 4.64E+03 |
| Medium pink | IL8 - 3 | Untreated | 5 | Direct | 0 | 3 | 9.09E+01 |  |  |
| Small yellow | IL8 - 3 | Untreated | 5 | Direct | 0 | 5 | 1.52E+02 |  |  |
| Small melons | IL8 - 3 | Untreated | 5 | Direct | 0 | 26 | 7.88E+02 |  |  |
| Small white | IL8 - 3 | Untreated | 5 | Direct | 0 | 25 | 7.58E+02 |  |  |
| Small translucent | IL8 - 3 | Untreated | 5 | Direct | 0 | 14 | 4.24E+02 |  |  |
| Medium oranges | IL8 - 3 | Untreated | 5 | Direct | 0 | 61 | 1.85E+03 |  |  |
| Translucent yellow | IL8 - 3 | Untreated | 5 | Direct | 0 | 17 | 5.15E+02 |  |  |
| Medium white | IL8 - 3 | Untreated | 5 | Heat | 0 | 17 | 5.15E+02 | 49 | 1.48E+03 |
| Medium pink | IL8 - 3 | Untreated | 5 | Heat | 0 | 19 | 5.76E+02 |  |  |
| Pink | IL8 - 3 | Untreated | 5 | Heat | 0 | 2 | 6.06E+01 |  |  |
| Medium yellow | IL8 - 3 | Untreated | 5 | Heat | 0 | 8 | 2.42E+02 |  |  |
| Small translucent | IL8 - 3 | Untreated | 5 | Heat | 0 | 3 | 9.09E+01 |  |  |
| Pink | IL4 - 1 | Heat | 5 | Direct | 3 | 1 | 3.03E+04 | 41 | 1.24E+06 |
| Medium yellow | IL4 - 1 | Heat | 5 | Direct | 3 | 20 | 6.06E+05 |  |  |
| Small yellow | IL4 - 1 | Heat | 5 | Direct | 3 | 1 | 3.03E+04 |  |  |
| Medium white | IL4 - 1 | Heat | 5 | Direct | 3 | 9 | 2.73E+05 |  |  |
| Small white | IL4 - 1 | Heat | 5 | Direct | 3 | 9 | 2.73E+05 |  |  |
| Small translucent | IL4 - 1 | Heat | 5 | Direct | 3 | 1 | 3.03E+04 |  |  |
| Medium white | IL4 - 1 | Heat | 5 | Heat | 2 | 14 | 4.24E+04 | 299 | 9.06E+05 |
| Pink | IL4 - 1 | Heat | 5 | Heat | 2 | 8 | 2.42E+04 |  |  |
| Medium yellow | IL4 - 1 | Heat | 5 | Heat | 2 | 92 | 2.79E+05 |  |  |
| Small yellow | IL4 - 1 | Heat | 5 | Heat | 2 | 134 | 4.06E+05 |  |  |
| Small white | IL4 - 1 | Heat | 5 | Heat | 2 | 51 | 1.55E+05 |  |  |
| Pink | IL4 - 2 | Heat | 5 | Direct | 2 | 1 | 3.03E+03 | 107 | 3.24E+05 |
| Medium yellow | IL4 - 2 | Heat | 5 | Direct | 2 | 45 | 1.36E+05 |  |  |
| Small yellow | IL4 - 2 | Heat | 5 | Direct | 2 | 3 | 9.09E+03 |  |  |
| Small melons | IL4 - 2 | Heat | 5 | Direct | 2 | 2 | 6.06E+03 |  |  |
| Medium white | IL4 - 2 | Heat | 5 | Direct | 2 | 19 | 5.76E+04 |  |  |
| Small white | IL4 - 2 | Heat | 5 | Direct | 2 | 34 | 1.03E+05 |  |  |
| Small translucent | IL4 - 2 | Heat | 5 | Direct | 2 | 3 | 9.09E+03 |  |  |
| Pink | IL4 - 2 | Heat | 5 | Heat | 2 | 2 | 6.06E+03 | 82 | 2.48E+05 |
| Medium yellow | IL4 - 2 | Heat | 5 | Heat | 2 | 26 | 7.88E+04 |  |  |
| Small yellow | IL4 - 2 | Heat | 5 | Heat | 2 | 6 | 1.82E+04 |  |  |
| Small melons | IL4 - 2 | Heat | 5 | Heat | 2 | 1 | 3.03E+03 |  |  |
| Medium white | IL4 - 2 | Heat | 5 | Heat | 2 | 37 | 1.12E+05 |  |  |
| Small white | IL4 - 2 | Heat | 5 | Heat | 2 | 10 | 3.03E+04 |  |  |
| Pink | IL4 -3 | Heat | 5 | Direct | 2 | 11 | 3.33E+04 | 136 | 4.12E+05 |
| Medium yellow | IL4 -3 | Heat | 5 | Direct | 2 | 68 | 2.06E+05 |  |  |
| Small yellow | IL4 -3 | Heat | 5 | Direct | 2 | 15 | 4.55E+04 |  |  |
| Medium white | IL4 -3 | Heat | 5 | Direct | 2 | 17 | 5.15E+04 |  |  |
| Small white | IL4 -3 | Heat | 5 | Direct | 2 | 22 | 6.67E+04 |  |  |
| Small translucent | IL4 -3 | Heat | 5 | Direct | 2 | 1 | 3.03E+03 |  |  |
| Translucent yellow | IL4 -3 | Heat | 5 | Direct | 2 | 2 | 6.06E+03 |  |  |
| Pink | IL4 -3 | Heat | 5 | Heat | 2 | 6 | 1.82E+04 | 122 | 3.70E+05 |
| Medium yellow | IL4 -3 | Heat | 5 | Heat | 2 | 12 | 3.64E+04 |  |  |
| Small melons | IL4 -3 | Heat | 5 | Heat | 2 | 2 | 6.06E+03 |  |  |
| Medium white | IL4 -3 | Heat | 5 | Heat | 2 | 43 | 1.30E+05 |  |  |
| Small white | IL4 -3 | Heat | 5 | Heat | 2 | 25 | 7.58E+04 |  |  |
| Large translucent | IL4 -3 | Heat | 5 | Heat | 2 | 34 | 1.03E+05 |  |  |
| Pink | IL5 - 1 | Heat | 5 | Direct | 2 | 62 | 1.88E+05 | 126 | 3.82E+05 |
| Medium yellow | IL5 - 1 | Heat | 5 | Direct | 2 | 3 | 9.09E+03 |  |  |
| Small white | IL5 - 1 | Heat | 5 | Direct | 2 | 61 | 1.85E+05 |  |  |
| Pink | IL5 - 1 | Heat | 5 | Heat | 2 | 22 | 6.67E+04 | 121 | 3.67E+05 |
| Medium yellow | IL5 - 1 | Heat | 5 | Heat | 2 | 3 | 9.09E+03 |  |  |
| Small yellow | IL5 - 1 | Heat | 5 | Heat | 2 | 6 | 1.82E+04 |  |  |
| Small white | IL5 - 1 | Heat | 5 | Heat | 2 | 90 | 2.73E+05 |  |  |
| Pink | IL5 - 2 | Heat | 5 | Direct | 2 | 61 | 1.85E+05 | 107 | 3.24E+05 |
| Medium yellow | IL5 - 2 | Heat | 5 | Direct | 2 | 6 | 1.82E+04 |  |  |
| Small yellow | IL5 - 2 | Heat | 5 | Direct | 2 | 11 | 3.33E+04 |  |  |
| Medium white | IL5 - 2 | Heat | 5 | Direct | 2 | 4 | 1.21E+04 |  |  |
| Small white | IL5 - 2 | Heat | 5 | Direct | 2 | 23 | 6.97E+04 |  |  |
| Small translucent | IL5 - 2 | Heat | 5 | Direct | 2 | 2 | 6.06E+03 |  |  |
| Pink | IL5 - 2 | Heat | 5 | Heat | 2 | 68 | 2.06E+05 | 194 | 5.88E+05 |
| Medium yellow | IL5 - 2 | Heat | 5 | Heat | 2 | 4 | 1.21E+04 |  |  |
| Small yellow | IL5 - 2 | Heat | 5 | Heat | 2 | 13 | 3.94E+04 |  |  |
| Medium white | IL5 - 2 | Heat | 5 | Heat | 2 | 10 | 3.03E+04 |  |  |
| Small white | IL5 - 2 | Heat | 5 | Heat | 2 | 99 | 3.00E+05 |  |  |
| Pink | IL5 - 3 | Heat | 5 | Direct | 3 | 8 | 2.42E+05 | 20 | 6.06E+05 |
| Medium yellow | IL5 - 3 | Heat | 5 | Direct | 3 | 4 | 1.21E+05 |  |  |
| Medium white | IL5 - 3 | Heat | 5 | Direct | 3 | 2 | 6.06E+04 |  |  |
| Small white | IL5 - 3 | Heat | 5 | Direct | 3 | 5 | 1.52E+05 |  |  |
| Translucent yellow | IL5 - 3 | Heat | 5 | Direct | 3 | 1 | 3.03E+04 |  |  |
| Pink | IL5 - 3 | Heat | 5 | Heat | 3 | 9 | 2.73E+05 | 21 | 6.36E+05 |
| Medium yellow | IL5 - 3 | Heat | 5 | Heat | 3 | 1 | 3.03E+04 |  |  |
| Medium white | IL5 - 3 | Heat | 5 | Heat | 3 | 2 | 6.06E+04 |  |  |
| Small white | IL5 - 3 | Heat | 5 | Heat | 3 | 9 | 2.73E+05 |  |  |
| Pink | IL8 - 1 | Heat | 5 | Direct | 2 | 1 | 3.03E+03 | 186 | 5.64E+05 |
| Small yellow | IL8 - 1 | Heat | 5 | Direct | 2 | 129 | 3.91E+05 |  |  |
| Small melons | IL8 - 1 | Heat | 5 | Direct | 2 | 1 | 3.03E+03 |  |  |
| Small white | IL8 - 1 | Heat | 5 | Direct | 2 | 46 | 1.39E+05 |  |  |
| Small translucent | IL8 - 1 | Heat | 5 | Direct | 2 | 7 | 2.12E+04 |  |  |
| Yellow cottony | IL8 - 1 | Heat | 5 | Direct | 2 | 2 | 6.06E+03 |  |  |
| Pink | IL8 - 1 | Heat | 5 | Heat | 2 | 3 | 9.09E+03 | 142 | 4.30E+05 |
| Small yellow | IL8 - 1 | Heat | 5 | Heat | 2 | 117 | 3.55E+05 |  |  |
| Small melons | IL8 - 1 | Heat | 5 | Heat | 2 | 2 | 6.06E+03 |  |  |
| Small white | IL8 - 1 | Heat | 5 | Heat | 2 | 20 | 6.06E+04 |  |  |
| Pink | IL8 - 2 | Heat | 5 | Direct | 2 | 1 | 3.03E+03 | 186 | 5.64E+05 |
| Medium yellow | IL8 - 2 | Heat | 5 | Direct | 2 | 45 | 1.36E+05 |  |  |
| Small yellow | IL8 - 2 | Heat | 5 | Direct | 2 | 104 | 3.15E+05 |  |  |
| Medium white | IL8 - 2 | Heat | 5 | Direct | 2 | 4 | 1.21E+04 |  |  |
| Small white | IL8 - 2 | Heat | 5 | Direct | 2 | 29 | 8.79E+04 |  |  |
| Yellow cottony | IL8 - 2 | Heat | 5 | Direct | 2 | 3 | 9.09E+03 |  |  |
| Pink | IL8 - 2 | Heat | 5 | Heat | 2 | 1 | 3.03E+03 | 128 | 3.88E+05 |
| Medium yellow | IL8 - 2 | Heat | 5 | Heat | 2 | 82 | 2.48E+05 |  |  |
| Small yellow | IL8 - 2 | Heat | 5 | Heat | 2 | 20 | 6.06E+04 |  |  |
| Small melons | IL8 - 2 | Heat | 5 | Heat | 2 | 1 | 3.03E+03 |  |  |
| Medium white | IL8 - 2 | Heat | 5 | Heat | 2 | 11 | 3.33E+04 |  |  |
| Small white | IL8 - 2 | Heat | 5 | Heat | 2 | 7 | 2.12E+04 |  |  |
| Small translucent | IL8 - 2 | Heat | 5 | Heat | 2 | 2 | 6.06E+03 |  |  |
| Yellow cottony | IL8 - 2 | Heat | 5 | Heat | 2 | 3 | 9.09E+03 |  |  |
| Medium oranges | IL8 - 2 | Heat | 5 | Heat | 2 | 1 | 3.03E+03 |  |  |
| Pink | IL8 - 3 | Heat | 5 | Direct | 2 | 1 | 3.03E+03 | 156 | 4.73E+05 |
| Medium yellow | IL8 - 3 | Heat | 5 | Direct | 2 | 75 | 2.27E+05 |  |  |
| Small yellow | IL8 - 3 | Heat | 5 | Direct | 2 | 25 | 7.58E+04 |  |  |
| Small melons | IL8 - 3 | Heat | 5 | Direct | 2 | 2 | 6.06E+03 |  |  |
| Medium white | IL8 - 3 | Heat | 5 | Direct | 2 | 34 | 1.03E+05 |  |  |
| Small white | IL8 - 3 | Heat | 5 | Direct | 2 | 15 | 4.55E+04 |  |  |
| Small translucent | IL8 - 3 | Heat | 5 | Direct | 2 | 4 | 1.21E+04 |  |  |
| Medium white | IL8 - 3 | Heat | 5 | Heat | 2 | 7 | 2.12E+04 | 158 | 4.79E+05 |
| Pink | IL8 - 3 | Heat | 5 | Heat | 2 | 2 | 6.06E+03 |  |  |
| Medium yellow | IL8 - 3 | Heat | 5 | Heat | 2 | 39 | 1.18E+05 |  |  |
| Small yellow | IL8 - 3 | Heat | 5 | Heat | 2 | 104 | 3.15E+05 |  |  |
| Small white | IL8 - 3 | Heat | 5 | Heat | 2 | 6 | 1.82E+04 |  |  |
| Small white | IL4 - 1 | Untreated | 6 | Direct | 0 | 2 | 6.06E+01 | 2 | 6.06E+01 |
| Medium white | IL4 - 1 | Untreated | 6 | Heat | 0 | 1 | 3.03E+01 | 4 | 1.21E+02 |
| Small white | IL4 - 1 | Untreated | 6 | Heat | 0 | 3 | 9.09E+01 |  |  |
| Small yellow | IL4 - 2 | Untreated | 6 | Direct | 0 | 4 | 1.21E+02 | 9 | 2.73E+02 |
| Medium white | IL4 - 2 | Untreated | 6 | Direct | 0 | 2 | 6.06E+01 |  |  |
| Small white | IL4 - 2 | Untreated | 6 | Direct | 0 | 3 | 9.09E+01 |  |  |
| Medium white | IL4 - 2 | Untreated | 6 | Heat | 0 | 2 | 6.06E+01 | 7 | 2.12E+02 |
| Medium pink | IL4 - 2 | Untreated | 6 | Heat | 0 | 1 | 3.03E+01 |  |  |
| Medium yellow | IL4 - 2 | Untreated | 6 | Heat | 0 | 1 | 3.03E+01 |  |  |
| Small melons | IL4 - 2 | Untreated | 6 | Heat | 0 | 1 | 3.03E+01 |  |  |
| Small white | IL4 - 2 | Untreated | 6 | Heat | 0 | 2 | 6.06E+01 |  |  |
| Medium white | IL5 - 1 | Untreated | 6 | Direct | 0 | 1 | 3.03E+01 | 12 | 3.64E+02 |
| Small melons | IL5 - 1 | Untreated | 6 | Direct | 0 | 1 | 3.03E+01 |  |  |
| Small white | IL5 - 1 | Untreated | 6 | Direct | 0 | 2 | 6.06E+01 |  |  |
| Large translucent | IL5 - 1 | Untreated | 6 | Direct | 0 | 2 | 6.06E+01 |  |  |
| Small translucent | IL5 - 1 | Untreated | 6 | Direct | 0 | 4 | 1.21E+02 |  |  |
| Translucent yellow | IL5 - 1 | Untreated | 6 | Direct | 0 | 2 | 6.06E+01 |  |  |
| Medium white | IL5 - 1 | Untreated | 6 | Heat | 0 | 1 | 3.03E+01 | 3 | 9.09E+01 |
| Small white | IL5 - 1 | Untreated | 6 | Heat | 0 | 2 | 6.06E+01 |  |  |
| Medium white | IL5 - 2 | Untreated | 6 | Direct | 1 | 1 | 3.03E+02 | 30 | 9.09E+03 |
| Small translucent | IL5 - 2 | Untreated | 6 | Direct | 1 | 29 | 8.79E+03 |  |  |
| Medium white | IL5 - 2 | Untreated | 6 | Heat | 0 | 3 | 9.09E+01 | 14 | 4.24E+02 |
| Medium pink | IL5 - 2 | Untreated | 6 | Heat | 0 | 1 | 3.03E+01 |  |  |
| Medium yellow | IL5 - 2 | Untreated | 6 | Heat | 0 | 5 | 1.52E+02 |  |  |
| Small white | IL5 - 2 | Untreated | 6 | Heat | 0 | 5 | 1.52E+02 |  |  |
| Medium white | IL5 - 3 | Untreated | 6 | Direct | 0 | 2 | 6.06E+01 | 21 | 6.36E+02 |
| Medium yellow | IL5 - 3 | Untreated | 6 | Direct | 0 | 2 | 6.06E+01 |  |  |
| Small yellow | IL5 - 3 | Untreated | 6 | Direct | 0 | 6 | 1.82E+02 |  |  |
| Small translucent | IL5 - 3 | Untreated | 6 | Direct | 0 | 11 | 3.33E+02 |  |  |
| Medium yellow | IL5 - 3 | Untreated | 6 | Heat | 1 | 1 | 3.03E+02 | 3 | 9.09E+02 |
| Small melons | IL5 - 3 | Untreated | 6 | Heat | 1 | 1 | 3.03E+02 |  |  |
| Medium white | IL5 - 3 | Untreated | 6 | Heat | 1 | 1 | 3.03E+02 |  |  |
| Medium white | IL8 - 1 | Untreated | 6 | Direct | 0 | 10 | 3.03E+02 | 123 | 3.73E+03 |
| Medium pink | IL8 - 1 | Untreated | 6 | Direct | 0 | 1 | 3.03E+01 |  |  |
| Small white | IL8 - 1 | Untreated | 6 | Direct | 0 | 112 | 3.39E+03 |  |  |
| Medium pink | IL8 - 1 | Untreated | 6 | Heat | 0 | 7 | 2.12E+02 | 32 | 9.70E+02 |
| Medium yellow | IL8 - 1 | Untreated | 6 | Heat | 0 | 2 | 6.06E+01 |  |  |
| Small yellow | IL8 - 1 | Untreated | 6 | Heat | 0 | 3 | 9.09E+01 |  |  |
| Small melons | IL8 - 1 | Untreated | 6 | Heat | 0 | 2 | 6.06E+01 |  |  |
| Medium white | IL8 - 1 | Untreated | 6 | Heat | 0 | 10 | 3.03E+02 |  |  |
| Small white | IL8 - 1 | Untreated | 6 | Heat | 0 | 8 | 2.42E+02 |  |  |
| Medium yellow | IL8 - 2 | Untreated | 6 | Direct | 0 | 3 | 9.09E+01 | 4 | 1.21E+02 |
| Medium white | IL8 - 2 | Untreated | 6 | Direct | 0 | 1 | 3.03E+01 |  |  |
| Medium pink | IL8 - 2 | Untreated | 6 | Heat | 0 | 5 | 1.52E+02 | 12 | 3.64E+02 |
| Medium white | IL8 - 2 | Untreated | 6 | Heat | 0 | 7 | 2.12E+02 |  |  |
| Pink | IL8 - 3 | Untreated | 6 | Direct | 2 | 1 | 3.03E+03 | 12 | 3.64E+04 |
| Medium yellow | IL8 - 3 | Untreated | 6 | Direct | 2 | 1 | 3.03E+03 |  |  |
| Medium white | IL8 - 3 | Untreated | 6 | Direct | 2 | 6 | 1.82E+04 |  |  |
| Small white | IL8 - 3 | Untreated | 6 | Direct | 2 | 2 | 6.06E+03 |  |  |
| Light brown | IL8 - 3 | Untreated | 6 | Direct | 2 | 1 | 3.03E+03 |  |  |
| Medium oranges | IL8 - 3 | Untreated | 6 | Direct | 2 | 1 | 3.03E+03 |  |  |
| Medium pink | IL8 - 3 | Untreated | 6 | Heat | 0 | 10 | 3.03E+02 | 14 | 4.24E+02 |
| Medium white | IL8 - 3 | Untreated | 6 | Heat | 0 | 1 | 3.03E+01 |  |  |
| Small white | IL8 - 3 | Untreated | 6 | Heat | 0 | 2 | 6.06E+01 |  |  |
| Light brown | IL8 - 3 | Untreated | 6 | Heat | 0 | 1 | 3.03E+01 |  |  |
| Pink | IL4 - 1 | Heat | 6 | Direct | 1 | 4 | 1.21E+03 | 113 | 3.42E+04 |
| Medium yellow | IL4 - 1 | Heat | 6 | Direct | 1 | 22 | 6.67E+03 |  |  |
| Small yellow | IL4 - 1 | Heat | 6 | Direct | 1 | 68 | 2.06E+04 |  |  |
| Medium white | IL4 - 1 | Heat | 6 | Direct | 1 | 10 | 3.03E+03 |  |  |
| Small white | IL4 - 1 | Heat | 6 | Direct | 1 | 9 | 2.73E+03 |  |  |
| Pink | IL4 - 1 | Heat | 6 | Heat | 1 | 9 | 2.73E+03 | 199 | 6.03E+04 |
| Medium yellow | IL4 - 1 | Heat | 6 | Heat | 1 | 26 | 7.88E+03 |  |  |
| Small yellow | IL4 - 1 | Heat | 6 | Heat | 1 | 149 | 4.52E+04 |  |  |
| Medium white | IL4 - 1 | Heat | 6 | Heat | 1 | 13 | 3.94E+03 |  |  |
| Small white | IL4 - 1 | Heat | 6 | Heat | 1 | 2 | 6.06E+02 |  |  |
| Pink | IL4 - 2 | Heat | 6 | Direct | 2 | 1 | 3.03E+03 | 47 | 1.42E+05 |
| Medium yellow | IL4 - 2 | Heat | 6 | Direct | 2 | 3 | 9.09E+03 |  |  |
| Medium white | IL4 - 2 | Heat | 6 | Direct | 2 | 28 | 8.48E+04 |  |  |
| Small white | IL4 - 2 | Heat | 6 | Direct | 2 | 12 | 3.64E+04 |  |  |
| Small translucent | IL4 - 2 | Heat | 6 | Direct | 2 | 3 | 9.09E+03 |  |  |
| Pink | IL4 - 2 | Heat | 6 | Heat | 1 | 2 | 6.06E+02 | 202 | 6.12E+04 |
| Medium yellow | IL4 - 2 | Heat | 6 | Heat | 1 | 42 | 1.27E+04 |  |  |
| Small yellow | IL4 - 2 | Heat | 6 | Heat | 1 | 45 | 1.36E+04 |  |  |
| Medium white | IL4 - 2 | Heat | 6 | Heat | 1 | 52 | 1.58E+04 |  |  |
| Small white | IL4 - 2 | Heat | 6 | Heat | 1 | 61 | 1.85E+04 |  |  |
| Medium yellow | IL4 -3 | Heat | 6 | Direct | 2 | 89 | 2.70E+05 | 131 | 3.97E+05 |
| Small yellow | IL4 -3 | Heat | 6 | Direct | 2 | 12 | 3.64E+04 |  |  |
| Medium white | IL4 -3 | Heat | 6 | Direct | 2 | 5 | 1.52E+04 |  |  |
| Small white | IL4 -3 | Heat | 6 | Direct | 2 | 25 | 7.58E+04 |  |  |
| Pink | IL4 -3 | Heat | 6 | Heat | 2 | 3 | 9.09E+03 | 110 | 3.33E+05 |
| Medium yellow | IL4 -3 | Heat | 6 | Heat | 2 | 9 | 2.73E+04 |  |  |
| Small yellow | IL4 -3 | Heat | 6 | Heat | 2 | 33 | 1.00E+05 |  |  |
| Small melons | IL4 -3 | Heat | 6 | Heat | 2 | 3 | 9.09E+03 |  |  |
| Medium white | IL4 -3 | Heat | 6 | Heat | 2 | 32 | 9.70E+04 |  |  |
| Small white | IL4 -3 | Heat | 6 | Heat | 2 | 30 | 9.09E+04 |  |  |
| Pink | IL5 - 1 | Heat | 6 | Direct | 2 | 41 | 1.24E+05 | 85 | 2.58E+05 |
| Medium yellow | IL5 - 1 | Heat | 6 | Direct | 2 | 9 | 2.73E+04 |  |  |
| Medium white | IL5 - 1 | Heat | 6 | Direct | 2 | 21 | 6.36E+04 |  |  |
| Small white | IL5 - 1 | Heat | 6 | Direct | 2 | 14 | 4.24E+04 |  |  |
| Pink | IL5 - 1 | Heat | 6 | Heat | 2 | 41 | 1.24E+05 | 136 | 4.12E+05 |
| Medium yellow | IL5 - 1 | Heat | 6 | Heat | 2 | 12 | 3.64E+04 |  |  |
| Small yellow | IL5 - 1 | Heat | 6 | Heat | 2 | 3 | 9.09E+03 |  |  |
| Medium white | IL5 - 1 | Heat | 6 | Heat | 2 | 5 | 1.52E+04 |  |  |
| Small white | IL5 - 1 | Heat | 6 | Heat | 2 | 75 | 2.27E+05 |  |  |
| Pink | IL5 - 2 | Heat | 6 | Direct | 2 | 59 | 1.79E+05 | 129 | 3.91E+05 |
| Medium yellow | IL5 - 2 | Heat | 6 | Direct | 2 | 23 | 6.97E+04 |  |  |
| Small yellow | IL5 - 2 | Heat | 6 | Direct | 2 | 7 | 2.12E+04 |  |  |
| Medium white | IL5 - 2 | Heat | 6 | Direct | 2 | 19 | 5.76E+04 |  |  |
| Small white | IL5 - 2 | Heat | 6 | Direct | 2 | 21 | 6.36E+04 |  |  |
| Pink | IL5 - 2 | Heat | 6 | Heat | 2 | 69 | 2.09E+05 | 159 | 4.82E+05 |
| Medium yellow | IL5 - 2 | Heat | 6 | Heat | 2 | 11 | 3.33E+04 |  |  |
| Medium white | IL5 - 2 | Heat | 6 | Heat | 2 | 1 | 3.03E+03 |  |  |
| Small white | IL5 - 2 | Heat | 6 | Heat | 2 | 78 | 2.36E+05 |  |  |
| Pink | IL5 - 3 | Heat | 6 | Direct | 1 | 63 | 1.91E+04 | 161 | 4.88E+04 |
| Small yellow | IL5 - 3 | Heat | 6 | Direct | 1 | 61 | 1.85E+04 |  |  |
| Medium white | IL5 - 3 | Heat | 6 | Direct | 1 | 9 | 2.73E+03 |  |  |
| Small white | IL5 - 3 | Heat | 6 | Direct | 1 | 28 | 8.48E+03 |  |  |
| Pink | IL5 - 3 | Heat | 6 | Heat | 1 | 76 | 2.30E+04 | 174 | 5.27E+04 |
| Small yellow | IL5 - 3 | Heat | 6 | Heat | 1 | 9 | 2.73E+03 |  |  |
| Small white | IL5 - 3 | Heat | 6 | Heat | 1 | 89 | 2.70E+04 |  |  |
| Medium white | IL8 - 1 | Heat | 6 | Direct | 1 | 38 | 1.15E+04 | 74 | 2.24E+04 |
| Medium yellow | IL8 - 1 | Heat | 6 | Direct | 1 | 22 | 6.67E+03 |  |  |
| Small melons | IL8 - 1 | Heat | 6 | Direct | 1 | 4 | 1.21E+03 |  |  |
| Small white | IL8 - 1 | Heat | 6 | Direct | 1 | 10 | 3.03E+03 |  |  |
| Medium pink | IL8 - 1 | Heat | 6 | Heat | 1 | 1 | 3.03E+02 | 53 | 1.61E+04 |
| Small melons | IL8 - 1 | Heat | 6 | Heat | 1 | 3 | 9.09E+02 |  |  |
| Medium white | IL8 - 1 | Heat | 6 | Heat | 1 | 49 | 1.48E+04 |  |  |
| Medium pink | IL8 - 2 | Heat | 6 | Heat | 1 | 1 | 3.03E+02 | 21 | 6.36E+03 |
| Medium yellow | IL8 - 2 | Heat | 6 | Heat | 1 | 7 | 2.12E+03 |  |  |
| Medium white | IL8 - 2 | Heat | 6 | Heat | 1 | 7 | 2.12E+03 |  |  |
| Small white | IL8 - 2 | Heat | 6 | Heat | 1 | 5 | 1.52E+03 |  |  |
| Small translucent | IL8 - 2 | Heat | 6 | Heat | 1 | 1 | 3.03E+02 |  |  |
| Medium yellow | IL8 - 3 | Heat | 6 | Direct | 2 | 59 | 1.79E+05 | 176 | 5.33E+05 |
| Small yellow | IL8 - 3 | Heat | 6 | Direct | 2 | 5 | 1.52E+04 |  |  |
| Medium white | IL8 - 3 | Heat | 6 | Direct | 2 | 35 | 1.06E+05 |  |  |
| Small white | IL8 - 3 | Heat | 6 | Direct | 2 | 77 | 2.33E+05 |  |  |
| Small yellow | IL8 - 3 | Heat | 6 | Heat | 2 | 19 | 5.76E+04 | 69 | 2.09E+05 |
| Small melons | IL8 - 3 | Heat | 6 | Heat | 2 | 7 | 2.12E+04 |  |  |
| Medium white | IL8 - 3 | Heat | 6 | Heat | 2 | 2 | 6.06E+03 |  |  |
| Small white | IL8 - 3 | Heat | 6 | Heat | 2 | 39 | 1.18E+05 |  |  |
| Medium oranges | IL8 - 3 | Heat | 6 | Heat | 2 | 2 | 6.06E+03 |  |  |
| Medium yellow | IL4 - 1 | Untreated | 7 | Direct | 0 | 4 | 1.21E+02 | 10 | 3.03E+02 |
| Medium white | IL4 - 1 | Untreated | 7 | Direct | 0 | 5 | 1.52E+02 |  |  |
| Small white | IL4 - 1 | Untreated | 7 | Direct | 0 | 1 | 3.03E+01 |  |  |
| Medium pink | IL4 - 1 | Untreated | 7 | Heat | 0 | 1 | 3.03E+01 | 1 | 3.03E+01 |
| Medium pink | IL4 - 2 | Untreated | 7 | Direct | 0 | 7 | 2.12E+02 | 19 | 5.76E+02 |
| Medium yellow | IL4 - 2 | Untreated | 7 | Direct | 0 | 4 | 1.21E+02 |  |  |
| Medium white | IL4 - 2 | Untreated | 7 | Direct | 0 | 1 | 3.03E+01 |  |  |
| Small white | IL4 - 2 | Untreated | 7 | Direct | 0 | 7 | 2.12E+02 |  |  |
| Medium white | IL4 - 3 | Untreated | 7 | Direct | 0 | 2 | 6.06E+01 | 7 | 2.12E+02 |
| Medium yellow | IL4 - 3 | Untreated | 7 | Direct | 0 | 3 | 9.09E+01 |  |  |
| Small yellow | IL4 - 3 | Untreated | 7 | Direct | 0 | 1 | 3.03E+01 |  |  |
| Small melons | IL4 - 3 | Untreated | 7 | Direct | 0 | 1 | 3.03E+01 |  |  |
| Medium yellow | IL5 - 1 | Untreated | 7 | Direct | 0 | 3 | 9.09E+01 | 17 | 5.15E+02 |
| Small melons | IL5 - 1 | Untreated | 7 | Direct | 0 | 2 | 6.06E+01 |  |  |
| Medium white | IL5 - 1 | Untreated | 7 | Direct | 0 | 3 | 9.09E+01 |  |  |
| Small white | IL5 - 1 | Untreated | 7 | Direct | 0 | 6 | 1.82E+02 |  |  |
| Translucent yellow | IL5 - 1 | Untreated | 7 | Direct | 0 | 3 | 9.09E+01 |  |  |
| Small white | IL5 - 1 | Untreated | 7 | Heat | 0 | 2 | 6.06E+01 | 2 | 6.06E+01 |
| Medium white | IL5 - 2 | Untreated | 7 | Direct | 1 | 4 | 1.21E+03 | 10 | 3.03E+03 |
| Small yellow | IL5 - 2 | Untreated | 7 | Direct | 1 | 2 | 6.06E+02 |  |  |
| Small white | IL5 - 2 | Untreated | 7 | Direct | 1 | 4 | 1.21E+03 |  |  |
| Medium white | IL5 - 2 | Untreated | 7 | Heat | 0 | 17 | 5.15E+02 | 23 | 6.97E+02 |
| Pink | IL5 - 2 | Untreated | 7 | Heat | 0 | 2 | 6.06E+01 |  |  |
| Small white | IL5 - 2 | Untreated | 7 | Heat | 0 | 4 | 1.21E+02 |  |  |
| Medium white | IL5 - 3 | Untreated | 7 | Direct | 0 | 2 | 6.06E+01 | 3 | 9.09E+01 |
| Small white | IL5 - 3 | Untreated | 7 | Direct | 0 | 1 | 3.03E+01 |  |  |
| Medium yellow | IL5 - 3 | Untreated | 7 | Heat | 1 | 1 | 3.03E+02 | 2 | 6.06E+02 |
| Medium white | IL5 - 3 | Untreated | 7 | Heat | 1 | 1 | 3.03E+02 |  |  |
| Medium melons | IL8 - 1 | Untreated | 7 | Direct | 0 | 1 | 3.03E+01 | 1 | 3.03E+01 |
| Medium white | IL8 - 1 | Untreated | 7 | Heat | 0 | 8 | 2.42E+02 | 19 | 5.76E+02 |
| Medium yellow | IL8 - 1 | Untreated | 7 | Heat | 0 | 1 | 3.03E+01 |  |  |
| Small yellow | IL8 - 1 | Untreated | 7 | Heat | 0 | 2 | 6.06E+01 |  |  |
| Small white | IL8 - 1 | Untreated | 7 | Heat | 0 | 2 | 6.06E+01 |  |  |
| Medium translucent | IL8 - 1 | Untreated | 7 | Heat | 0 | 6 | 1.82E+02 |  |  |
| Medium pink | IL8 - 2 | Untreated | 7 | Heat | 0 | 3 | 9.09E+01 | 59 | 1.79E+03 |
| Pink | IL8 - 2 | Untreated | 7 | Heat | 0 | 12 | 3.64E+02 |  |  |
| Medium yellow | IL8 - 2 | Untreated | 7 | Heat | 0 | 1 | 3.03E+01 |  |  |
| Small yellow | IL8 - 2 | Untreated | 7 | Heat | 0 | 28 | 8.48E+02 |  |  |
| Medium white | IL8 - 2 | Untreated | 7 | Heat | 0 | 12 | 3.64E+02 |  |  |
| Large translucent | IL8 - 2 | Untreated | 7 | Heat | 0 | 3 | 9.09E+01 |  |  |
| Medium pink | IL8 - 3 | Untreated | 7 | Direct | 0 | 1 | 3.03E+01 | 1 | 3.03E+01 |
| Pink | IL8 - 3 | Untreated | 7 | Heat | 0 | 3 | 9.09E+01 | 9 | 2.73E+02 |
| Small melons | IL8 - 3 | Untreated | 7 | Heat | 0 | 2 | 6.06E+01 |  |  |
| Medium white | IL8 - 3 | Untreated | 7 | Heat | 0 | 2 | 6.06E+01 |  |  |
| Small white | IL8 - 3 | Untreated | 7 | Heat | 0 | 1 | 3.03E+01 |  |  |
| Small translucent | IL8 - 3 | Untreated | 7 | Heat | 0 | 1 | 3.03E+01 |  |  |
| Pink | IL4 - 1 | Heat | 7 | Direct | 2 | 6 | 1.82E+04 | 60 | 1.82E+05 |
| Small yellow | IL4 - 1 | Heat | 7 | Direct | 2 | 16 | 4.85E+04 |  |  |
| Small white | IL4 - 1 | Heat | 7 | Direct | 2 | 38 | 1.15E+05 |  |  |
| Medium white | IL4 - 1 | Heat | 7 | Heat | 2 | 1 | 3.03E+03 | 24 | 7.27E+04 |
| Pink | IL4 - 1 | Heat | 7 | Heat | 2 | 6 | 1.82E+04 |  |  |
| Small yellow | IL4 - 1 | Heat | 7 | Heat | 2 | 3 | 9.09E+03 |  |  |
| Small white | IL4 - 1 | Heat | 7 | Heat | 2 | 14 | 4.24E+04 |  |  |
| Pink | IL4 - 2 | Heat | 7 | Direct | 2 | 3 | 9.09E+03 | 173 | 5.24E+05 |
| Small yellow | IL4 - 2 | Heat | 7 | Direct | 2 | 78 | 2.36E+05 |  |  |
| Small white | IL4 - 2 | Heat | 7 | Direct | 2 | 92 | 2.79E+05 |  |  |
| Pink | IL4 - 2 | Heat | 7 | Heat | 2 | 4 | 1.21E+04 | 185 | 5.61E+05 |
| Small yellow | IL4 - 2 | Heat | 7 | Heat | 2 | 92 | 2.79E+05 |  |  |
| Small white | IL4 - 2 | Heat | 7 | Heat | 2 | 89 | 2.70E+05 |  |  |
| Pink | IL4 -3 | Heat | 7 | Direct | 2 | 9 | 2.73E+04 | 84 | 2.55E+05 |
| Medium yellow | IL4 -3 | Heat | 7 | Direct | 2 | 25 | 7.58E+04 |  |  |
| Small yellow | IL4 -3 | Heat | 7 | Direct | 2 | 14 | 4.24E+04 |  |  |
| Medium white | IL4 -3 | Heat | 7 | Direct | 2 | 4 | 1.21E+04 |  |  |
| Small white | IL4 -3 | Heat | 7 | Direct | 2 | 32 | 9.70E+04 |  |  |
| Pink | IL4 -3 | Heat | 7 | Heat | 2 | 5 | 1.52E+04 | 128 | 3.88E+05 |
| Medium yellow | IL4 -3 | Heat | 7 | Heat | 2 | 38 | 1.15E+05 |  |  |
| Small yellow | IL4 -3 | Heat | 7 | Heat | 2 | 33 | 1.00E+05 |  |  |
| Medium white | IL4 -3 | Heat | 7 | Heat | 2 | 6 | 1.82E+04 |  |  |
| Small white | IL4 -3 | Heat | 7 | Heat | 2 | 46 | 1.39E+05 |  |  |
| Pink | IL5 - 1 | Heat | 7 | Direct | 2 | 55 | 1.67E+05 | 176 | 5.33E+05 |
| Medium yellow | IL5 - 1 | Heat | 7 | Direct | 2 | 3 | 9.09E+03 |  |  |
| Medium white | IL5 - 1 | Heat | 7 | Direct | 2 | 11 | 3.33E+04 |  |  |
| Small white | IL5 - 1 | Heat | 7 | Direct | 2 | 107 | 3.24E+05 |  |  |
| Pink | IL5 - 1 | Heat | 7 | Heat | 2 | 68 | 2.06E+05 | 190 | 5.76E+05 |
| Medium yellow | IL5 - 1 | Heat | 7 | Heat | 2 | 9 | 2.73E+04 |  |  |
| Medium white | IL5 - 1 | Heat | 7 | Heat | 2 | 27 | 8.18E+04 |  |  |
| Small white | IL5 - 1 | Heat | 7 | Heat | 2 | 86 | 2.61E+05 |  |  |
| Pink | IL5 - 2 | Heat | 7 | Direct | 2 | 69 | 2.09E+05 | 117 | 3.55E+05 |
| Medium yellow | IL5 - 2 | Heat | 7 | Direct | 2 | 41 | 1.24E+05 |  |  |
| Small melons | IL5 - 2 | Heat | 7 | Direct | 2 | 1 | 3.03E+03 |  |  |
| Medium white | IL5 - 2 | Heat | 7 | Direct | 2 | 5 | 1.52E+04 |  |  |
| Small white | IL5 - 2 | Heat | 7 | Direct | 2 | 1 | 3.03E+03 |  |  |
| Pink | IL5 - 2 | Heat | 7 | Heat | 2 | 56 | 1.70E+05 | 92 | 2.79E+05 |
| Medium yellow | IL5 - 2 | Heat | 7 | Heat | 2 | 17 | 5.15E+04 |  |  |
| Small yellow | IL5 - 2 | Heat | 7 | Heat | 2 | 2 | 6.06E+03 |  |  |
| Small melons | IL5 - 2 | Heat | 7 | Heat | 2 | 2 | 6.06E+03 |  |  |
| Medium white | IL5 - 2 | Heat | 7 | Heat | 2 | 5 | 1.52E+04 |  |  |
| Small white | IL5 - 2 | Heat | 7 | Heat | 2 | 10 | 3.03E+04 |  |  |
| Pink | IL5 - 3 | Heat | 7 | Direct | 3 | 10 | 3.03E+05 | 29 | 8.79E+05 |
| Medium yellow | IL5 - 3 | Heat | 7 | Direct | 3 | 3 | 9.09E+04 |  |  |
| Small yellow | IL5 - 3 | Heat | 7 | Direct | 3 | 6 | 1.82E+05 |  |  |
| Medium white | IL5 - 3 | Heat | 7 | Direct | 3 | 10 | 3.03E+05 |  |  |
| Pink | IL5 - 3 | Heat | 7 | Heat | 2 | 93 | 2.82E+05 | 204 | 6.18E+05 |
| Medium yellow | IL5 - 3 | Heat | 7 | Heat | 2 | 17 | 5.15E+04 |  |  |
| Small yellow | IL5 - 3 | Heat | 7 | Heat | 2 | 37 | 1.12E+05 |  |  |
| Medium white | IL5 - 3 | Heat | 7 | Heat | 2 | 26 | 7.88E+04 |  |  |
| Small white | IL5 - 3 | Heat | 7 | Heat | 2 | 31 | 9.39E+04 |  |  |
| Pink | IL8 - 1 | Heat | 7 | Direct | 2 | 1 | 3.03E+03 | 116 | 3.52E+05 |
| Medium yellow | IL8 - 1 | Heat | 7 | Direct | 2 | 86 | 2.61E+05 |  |  |
| Small melons | IL8 - 1 | Heat | 7 | Direct | 2 | 2 | 6.06E+03 |  |  |
| Medium white | IL8 - 1 | Heat | 7 | Direct | 2 | 18 | 5.45E+04 |  |  |
| Small white | IL8 - 1 | Heat | 7 | Direct | 2 | 9 | 2.73E+04 |  |  |
| Medium yellow | IL8 - 1 | Heat | 7 | Heat | 2 | 50 | 1.52E+05 | 102 | 3.09E+05 |
| Small melons | IL8 - 1 | Heat | 7 | Heat | 2 | 1 | 3.03E+03 |  |  |
| Medium white | IL8 - 1 | Heat | 7 | Heat | 2 | 14 | 4.24E+04 |  |  |
| Small white | IL8 - 1 | Heat | 7 | Heat | 2 | 34 | 1.03E+05 |  |  |
| Small translucent | IL8 - 1 | Heat | 7 | Heat | 2 | 3 | 9.09E+03 |  |  |
| Medium pink | IL8 - 2 | Heat | 7 | Direct | 2 | 1 | 3.03E+03 | 127 | 3.85E+05 |
| Medium yellow | IL8 - 2 | Heat | 7 | Direct | 2 | 84 | 2.55E+05 |  |  |
| Medium white | IL8 - 2 | Heat | 7 | Direct | 2 | 27 | 8.18E+04 |  |  |
| Small white | IL8 - 2 | Heat | 7 | Direct | 2 | 15 | 4.55E+04 |  |  |
| Pink | IL8 - 2 | Heat | 7 | Heat | 2 | 2 | 6.06E+03 | 166 | 5.03E+05 |
| Medium yellow | IL8 - 2 | Heat | 7 | Heat | 2 | 139 | 4.21E+05 |  |  |
| Medium white | IL8 - 2 | Heat | 7 | Heat | 2 | 25 | 7.58E+04 |  |  |
| Medium yellow | IL8 - 3 | Heat | 7 | Direct | 3 | 14 | 4.24E+05 | 20 | 6.06E+05 |
| Medium white | IL8 - 3 | Heat | 7 | Direct | 3 | 6 | 1.82E+05 |  |  |
| Medium white | IL8 - 3 | Heat | 7 | Heat | 2 | 49 | 1.48E+05 | 137 | 4.15E+05 |
| Pink | IL8 - 3 | Heat | 7 | Heat | 2 | 1 | 3.03E+03 |  |  |
| Medium yellow | IL8 - 3 | Heat | 7 | Heat | 2 | 87 | 2.64E+05 |  |  |
| Medium white | IL4 - 1 | Untreated | 8 | Direct | 0 | 1 | 3.03E+01 | 85 | 2.58E+03 |
| Medium yellow | IL4 - 1 | Untreated | 8 | Direct | 0 | 14 | 4.24E+02 |  |  |
| Small yellow | IL4 - 1 | Untreated | 8 | Direct | 0 | 18 | 5.45E+02 |  |  |
| Small melons | IL4 - 1 | Untreated | 8 | Direct | 0 | 1 | 3.03E+01 |  |  |
| Small white | IL4 - 1 | Untreated | 8 | Direct | 0 | 49 | 1.48E+03 |  |  |
| Medium oranges | IL4 - 1 | Untreated | 8 | Direct | 0 | 2 | 6.06E+01 |  |  |
| Medium white | IL4 - 1 | Untreated | 8 | Heat | 0 | 1 | 3.03E+01 | 23 | 6.97E+02 |
| Medium pink | IL4 - 1 | Untreated | 8 | Heat | 0 | 1 | 3.03E+01 |  |  |
| Medium yellow | IL4 - 1 | Untreated | 8 | Heat | 0 | 18 | 5.45E+02 |  |  |
| Small melons | IL4 - 1 | Untreated | 8 | Heat | 0 | 2 | 6.06E+01 |  |  |
| Small white | IL4 - 1 | Untreated | 8 | Heat | 0 | 1 | 3.03E+01 |  |  |
| Small yellow | IL4 - 2 | Untreated | 8 | Direct | 0 | 3 | 9.09E+01 | 32 | 9.70E+02 |
| Small white | IL4 - 2 | Untreated | 8 | Direct | 0 | 28 | 8.48E+02 |  |  |
| Medium oranges | IL4 - 2 | Untreated | 8 | Direct | 0 | 1 | 3.03E+01 |  |  |
| Medium yellow | IL4 - 2 | Untreated | 8 | Heat | 0 | 2 | 6.06E+01 | 5 | 1.52E+02 |
| Small yellow | IL4 - 2 | Untreated | 8 | Heat | 0 | 2 | 6.06E+01 |  |  |
| Small white | IL4 - 2 | Untreated | 8 | Heat | 0 | 1 | 3.03E+01 |  |  |
| Medium pink | IL4 - 3 | Untreated | 8 | Direct | 0 | 6 | 1.82E+02 | 23 | 6.97E+02 |
| Medium yellow | IL4 - 3 | Untreated | 8 | Direct | 0 | 2 | 6.06E+01 |  |  |
| Small yellow | IL4 - 3 | Untreated | 8 | Direct | 0 | 2 | 6.06E+01 |  |  |
| Small white | IL4 - 3 | Untreated | 8 | Direct | 0 | 13 | 3.94E+02 |  |  |
| Medium white | IL4 - 3 | Untreated | 8 | Heat | 0 | 5 | 1.52E+02 | 17 | 5.15E+02 |
| Medium pink | IL4 - 3 | Untreated | 8 | Heat | 0 | 3 | 9.09E+01 |  |  |
| Medium yellow | IL4 - 3 | Untreated | 8 | Heat | 0 | 4 | 1.21E+02 |  |  |
| Small yellow | IL4 - 3 | Untreated | 8 | Heat | 0 | 1 | 3.03E+01 |  |  |
| Small melons | IL4 - 3 | Untreated | 8 | Heat | 0 | 1 | 3.03E+01 |  |  |
| Small white | IL4 - 3 | Untreated | 8 | Heat | 0 | 3 | 9.09E+01 |  |  |
| Pink | IL5 - 1 | Untreated | 8 | Direct | 0 | 1 | 3.03E+01 | 71 | 2.15E+03 |
| Medium yellow | IL5 - 1 | Untreated | 8 | Direct | 0 | 4 | 1.21E+02 |  |  |
| Small yellow | IL5 - 1 | Untreated | 8 | Direct | 0 | 2 | 6.06E+01 |  |  |
| Medium white | IL5 - 1 | Untreated | 8 | Direct | 0 | 1 | 3.03E+01 |  |  |
| Small white | IL5 - 1 | Untreated | 8 | Direct | 0 | 53 | 1.61E+03 |  |  |
| Small translucent | IL5 - 1 | Untreated | 8 | Direct | 0 | 10 | 3.03E+02 |  |  |
| Medium white | IL5 - 1 | Untreated | 8 | Heat | 0 | 7 | 2.12E+02 | 18 | 5.45E+02 |
| Medium pink | IL5 - 1 | Untreated | 8 | Heat | 0 | 1 | 3.03E+01 |  |  |
| Small melons | IL5 - 1 | Untreated | 8 | Heat | 0 | 1 | 3.03E+01 |  |  |
| Small white | IL5 - 1 | Untreated | 8 | Heat | 0 | 4 | 1.21E+02 |  |  |
| Small translucent | IL5 - 1 | Untreated | 8 | Heat | 0 | 5 | 1.52E+02 |  |  |
| Medium yellow | IL5 - 2 | Untreated | 8 | Direct | 0 | 7 | 2.12E+02 | 170 | 5.15E+03 |
| Small yellow | IL5 - 2 | Untreated | 8 | Direct | 0 | 24 | 7.27E+02 |  |  |
| Small melons | IL5 - 2 | Untreated | 8 | Direct | 0 | 2 | 6.06E+01 |  |  |
| Small white | IL5 - 2 | Untreated | 8 | Direct | 0 | 50 | 1.52E+03 |  |  |
| Small translucent | IL5 - 2 | Untreated | 8 | Direct | 0 | 87 | 2.64E+03 |  |  |
| Medium white | IL5 - 2 | Untreated | 8 | Heat | 0 | 6 | 1.82E+02 | 47 | 1.42E+03 |
| Medium pink | IL5 - 2 | Untreated | 8 | Heat | 0 | 2 | 6.06E+01 |  |  |
| Medium yellow | IL5 - 2 | Untreated | 8 | Heat | 0 | 19 | 5.76E+02 |  |  |
| Small yellow | IL5 - 2 | Untreated | 8 | Heat | 0 | 7 | 2.12E+02 |  |  |
| Small white | IL5 - 2 | Untreated | 8 | Heat | 0 | 13 | 3.94E+02 |  |  |
| Medium white | IL5 - 3 | Untreated | 8 | Direct | 0 | 1 | 3.03E+01 | 190 | 5.76E+03 |
| Medium pink | IL5 - 3 | Untreated | 8 | Direct | 0 | 2 | 6.06E+01 |  |  |
| Medium yellow | IL5 - 3 | Untreated | 8 | Direct | 0 | 47 | 1.42E+03 |  |  |
| Small white | IL5 - 3 | Untreated | 8 | Direct | 0 | 140 | 4.24E+03 |  |  |
| Medium white | IL5 - 3 | Untreated | 8 | Heat | 0 | 4 | 1.21E+02 | 84 | 2.55E+03 |
| Medium pink | IL5 - 3 | Untreated | 8 | Heat | 0 | 2 | 6.06E+01 |  |  |
| Pink | IL5 - 3 | Untreated | 8 | Heat | 0 | 1 | 3.03E+01 |  |  |
| Medium yellow | IL5 - 3 | Untreated | 8 | Heat | 0 | 69 | 2.09E+03 |  |  |
| Small melons | IL5 - 3 | Untreated | 8 | Heat | 0 | 4 | 1.21E+02 |  |  |
| Small white | IL5 - 3 | Untreated | 8 | Heat | 0 | 4 | 1.21E+02 |  |  |
| Medium white | IL8 - 1 | Untreated | 8 | Direct | 0 | 26 | 7.88E+02 | 107 | 3.24E+03 |
| Medium pink | IL8 - 1 | Untreated | 8 | Direct | 0 | 4 | 1.21E+02 |  |  |
| Medium yellow | IL8 - 1 | Untreated | 8 | Direct | 0 | 3 | 9.09E+01 |  |  |
| Small white | IL8 - 1 | Untreated | 8 | Direct | 0 | 35 | 1.06E+03 |  |  |
| Small translucent | IL8 - 1 | Untreated | 8 | Direct | 0 | 39 | 1.18E+03 |  |  |
| Medium pink | IL8 - 1 | Untreated | 8 | Heat | 0 | 13 | 3.94E+02 | 43 | 1.30E+03 |
| Medium yellow | IL8 - 1 | Untreated | 8 | Heat | 0 | 14 | 4.24E+02 |  |  |
| Medium white | IL8 - 1 | Untreated | 8 | Heat | 0 | 9 | 2.73E+02 |  |  |
| Small white | IL8 - 1 | Untreated | 8 | Heat | 0 | 7 | 2.12E+02 |  |  |
| Medium yellow | IL8 - 2 | Untreated | 8 | Direct | 0 | 5 | 1.52E+02 | 71 | 2.15E+03 |
| Medium white | IL8 - 2 | Untreated | 8 | Direct | 0 | 4 | 1.21E+02 |  |  |
| Small white | IL8 - 2 | Untreated | 8 | Direct | 0 | 60 | 1.82E+03 |  |  |
| Medium translucent | IL8 - 2 | Untreated | 8 | Direct | 0 | 2 | 6.06E+01 |  |  |
| Medium pink | IL8 - 2 | Untreated | 8 | Heat | 0 | 3 | 9.09E+01 | 19 | 5.76E+02 |
| Medium yellow | IL8 - 2 | Untreated | 8 | Heat | 0 | 7 | 2.12E+02 |  |  |
| Small white | IL8 - 2 | Untreated | 8 | Heat | 0 | 9 | 2.73E+02 |  |  |
| Medium white | IL8 - 3 | Untreated | 8 | Direct | 0 | 3 | 9.09E+01 | 126 | 3.82E+03 |
| Medium pink | IL8 - 3 | Untreated | 8 | Direct | 0 | 5 | 1.52E+02 |  |  |
| Medium yellow | IL8 - 3 | Untreated | 8 | Direct | 0 | 3 | 9.09E+01 |  |  |
| Small white | IL8 - 3 | Untreated | 8 | Direct | 0 | 75 | 2.27E+03 |  |  |
| Small translucent | IL8 - 3 | Untreated | 8 | Direct | 0 | 40 | 1.21E+03 |  |  |
| Medium white | IL8 - 3 | Untreated | 8 | Heat | 0 | 3 | 9.09E+01 | 22 | 6.67E+02 |
| Medium pink | IL8 - 3 | Untreated | 8 | Heat | 0 | 3 | 9.09E+01 |  |  |
| Medium yellow | IL8 - 3 | Untreated | 8 | Heat | 0 | 4 | 1.21E+02 |  |  |
| Small white | IL8 - 3 | Untreated | 8 | Heat | 0 | 12 | 3.64E+02 |  |  |
| Pink | IL4 - 1 | Heat | 8 | Direct | 2 | 5 | 1.52E+04 | 159 | 4.82E+05 |
| Medium yellow | IL4 - 1 | Heat | 8 | Direct | 2 | 14 | 4.24E+04 |  |  |
| Small yellow | IL4 - 1 | Heat | 8 | Direct | 2 | 102 | 3.09E+05 |  |  |
| Medium white | IL4 - 1 | Heat | 8 | Direct | 2 | 16 | 4.85E+04 |  |  |
| Small white | IL4 - 1 | Heat | 8 | Direct | 2 | 22 | 6.67E+04 |  |  |
| Pink | IL4 - 1 | Heat | 8 | Heat | 2 | 5 | 1.52E+04 | 99 | 3.00E+05 |
| Medium yellow | IL4 - 1 | Heat | 8 | Heat | 2 | 4 | 1.21E+04 |  |  |
| Small yellow | IL4 - 1 | Heat | 8 | Heat | 2 | 48 | 1.45E+05 |  |  |
| Medium white | IL4 - 1 | Heat | 8 | Heat | 2 | 19 | 5.76E+04 |  |  |
| Small white | IL4 - 1 | Heat | 8 | Heat | 2 | 23 | 6.97E+04 |  |  |
| Medium yellow | IL4 - 2 | Heat | 8 | Direct | 2 | 6 | 1.82E+04 | 239 | 7.24E+05 |
| Small yellow | IL4 - 2 | Heat | 8 | Direct | 2 | 112 | 3.39E+05 |  |  |
| Small white | IL4 - 2 | Heat | 8 | Direct | 2 | 121 | 3.67E+05 |  |  |
| Pink | IL4 - 2 | Heat | 8 | Heat | 2 | 2 | 6.06E+03 | 140 | 4.24E+05 |
| Medium yellow | IL4 - 2 | Heat | 8 | Heat | 2 | 42 | 1.27E+05 |  |  |
| Small yellow | IL4 - 2 | Heat | 8 | Heat | 2 | 38 | 1.15E+05 |  |  |
| Small melons | IL4 - 2 | Heat | 8 | Heat | 2 | 1 | 3.03E+03 |  |  |
| Medium white | IL4 - 2 | Heat | 8 | Heat | 2 | 27 | 8.18E+04 |  |  |
| Small white | IL4 - 2 | Heat | 8 | Heat | 2 | 30 | 9.09E+04 |  |  |
| Pink | IL4 -3 | Heat | 8 | Direct | 2 | 5 | 1.52E+04 | 190 | 5.76E+05 |
| Medium yellow | IL4 -3 | Heat | 8 | Direct | 2 | 44 | 1.33E+05 |  |  |
| Small yellow | IL4 -3 | Heat | 8 | Direct | 2 | 67 | 2.03E+05 |  |  |
| Medium white | IL4 -3 | Heat | 8 | Direct | 2 | 44 | 1.33E+05 |  |  |
| Small white | IL4 -3 | Heat | 8 | Direct | 2 | 16 | 4.85E+04 |  |  |
| Medium translucent | IL4 -3 | Heat | 8 | Direct | 2 | 4 | 1.21E+04 |  |  |
| Small translucent | IL4 -3 | Heat | 8 | Direct | 2 | 10 | 3.03E+04 |  |  |
| Pink | IL4 -3 | Heat | 8 | Heat | 2 | 10 | 3.03E+04 | 260 | 7.88E+05 |
| Small yellow | IL4 -3 | Heat | 8 | Heat | 2 | 158 | 4.79E+05 |  |  |
| Medium white | IL4 -3 | Heat | 8 | Heat | 2 | 16 | 4.85E+04 |  |  |
| Small white | IL4 -3 | Heat | 8 | Heat | 2 | 70 | 2.12E+05 |  |  |
| Medium translucent | IL4 -3 | Heat | 8 | Heat | 2 | 4 | 1.21E+04 |  |  |
| Small translucent | IL4 -3 | Heat | 8 | Heat | 2 | 2 | 6.06E+03 |  |  |
| Pink | IL5 - 1 | Heat | 8 | Direct | 2 | 53 | 1.61E+05 | 129 | 3.91E+05 |
| Medium yellow | IL5 - 1 | Heat | 8 | Direct | 2 | 12 | 3.64E+04 |  |  |
| Medium white | IL5 - 1 | Heat | 8 | Direct | 2 | 49 | 1.48E+05 |  |  |
| Small white | IL5 - 1 | Heat | 8 | Direct | 2 | 15 | 4.55E+04 |  |  |
| Pink | IL5 - 1 | Heat | 8 | Heat | 2 | 49 | 1.48E+05 | 148 | 4.48E+05 |
| Medium yellow | IL5 - 1 | Heat | 8 | Heat | 2 | 14 | 4.24E+04 |  |  |
| Medium white | IL5 - 1 | Heat | 8 | Heat | 2 | 24 | 7.27E+04 |  |  |
| Small white | IL5 - 1 | Heat | 8 | Heat | 2 | 59 | 1.79E+05 |  |  |
| Translucent yellow | IL5 - 1 | Heat | 8 | Heat | 2 | 2 | 6.06E+03 |  |  |
| Pink | IL5 - 2 | Heat | 8 | Direct | 2 | 61 | 1.85E+05 | 143 | 4.33E+05 |
| Medium yellow | IL5 - 2 | Heat | 8 | Direct | 2 | 4 | 1.21E+04 |  |  |
| Small yellow | IL5 - 2 | Heat | 8 | Direct | 2 | 9 | 2.73E+04 |  |  |
| Medium white | IL5 - 2 | Heat | 8 | Direct | 2 | 33 | 1.00E+05 |  |  |
| Small white | IL5 - 2 | Heat | 8 | Direct | 2 | 36 | 1.09E+05 |  |  |
| Pink | IL5 - 2 | Heat | 8 | Heat | 2 | 17 | 5.15E+04 | 50 | 1.52E+05 |
| Medium yellow | IL5 - 2 | Heat | 8 | Heat | 2 | 1 | 3.03E+03 |  |  |
| Small yellow | IL5 - 2 | Heat | 8 | Heat | 2 | 1 | 3.03E+03 |  |  |
| Medium white | IL5 - 2 | Heat | 8 | Heat | 2 | 11 | 3.33E+04 |  |  |
| Small white | IL5 - 2 | Heat | 8 | Heat | 2 | 20 | 6.06E+04 |  |  |
| Pink | IL5 - 3 | Heat | 8 | Direct | 2 | 64 | 1.94E+05 | 118 | 3.58E+05 |
| Medium yellow | IL5 - 3 | Heat | 8 | Direct | 2 | 7 | 2.12E+04 |  |  |
| Small yellow | IL5 - 3 | Heat | 8 | Direct | 2 | 8 | 2.42E+04 |  |  |
| Medium white | IL5 - 3 | Heat | 8 | Direct | 2 | 8 | 2.42E+04 |  |  |
| Small white | IL5 - 3 | Heat | 8 | Direct | 2 | 19 | 5.76E+04 |  |  |
| Medium translucent | IL5 - 3 | Heat | 8 | Direct | 2 | 3 | 9.09E+03 |  |  |
| Small translucent | IL5 - 3 | Heat | 8 | Direct | 2 | 9 | 2.73E+04 |  |  |
| Pink | IL5 - 3 | Heat | 8 | Heat | 3 | 3 | 9.09E+04 | 11 | 3.33E+05 |
| Medium yellow | IL5 - 3 | Heat | 8 | Heat | 3 | 5 | 1.52E+05 |  |  |
| Medium white | IL5 - 3 | Heat | 8 | Heat | 3 | 2 | 6.06E+04 |  |  |
| Translucent yellow | IL5 - 3 | Heat | 8 | Heat | 3 | 1 | 3.03E+04 |  |  |
| Pink | IL8 - 1 | Heat | 8 | Direct | 2 | 1 | 3.03E+03 | 222 | 6.73E+05 |
| Small yellow | IL8 - 1 | Heat | 8 | Direct | 2 | 113 | 3.42E+05 |  |  |
| Small white | IL8 - 1 | Heat | 8 | Direct | 2 | 98 | 2.97E+05 |  |  |
| Small translucent | IL8 - 1 | Heat | 8 | Direct | 2 | 10 | 3.03E+04 |  |  |
| Medium yellow | IL8 - 1 | Heat | 8 | Heat | 2 | 39 | 1.18E+05 | 50 | 1.52E+05 |
| Medium white | IL8 - 1 | Heat | 8 | Heat | 2 | 11 | 3.33E+04 |  |  |
| Medium yellow | IL8 - 2 | Heat | 8 | Direct | 2 | 66 | 2.00E+05 | 83 | 2.52E+05 |
| Medium white | IL8 - 2 | Heat | 8 | Direct | 2 | 15 | 4.55E+04 |  |  |
| Small translucent | IL8 - 2 | Heat | 8 | Direct | 2 | 2 | 6.06E+03 |  |  |
| Medium yellow | IL8 - 2 | Heat | 8 | Heat | 2 | 44 | 1.33E+05 | 54 | 1.64E+05 |
| Medium white | IL8 - 2 | Heat | 8 | Heat | 2 | 10 | 3.03E+04 |  |  |
| Pink | IL8 - 3 | Heat | 8 | Direct | 2 | 6 | 1.82E+04 | 183 | 5.55E+05 |
| Small yellow | IL8 - 3 | Heat | 8 | Direct | 2 | 109 | 3.30E+05 |  |  |
| Small melons | IL8 - 3 | Heat | 8 | Direct | 2 | 1 | 3.03E+03 |  |  |
| Small white | IL8 - 3 | Heat | 8 | Direct | 2 | 67 | 2.03E+05 |  |  |
| Pink | IL8 - 3 | Heat | 8 | Heat | 2 | 2 | 6.06E+03 | 66 | 2.00E+05 |
| Medium yellow | IL8 - 3 | Heat | 8 | Heat | 2 | 50 | 1.52E+05 |  |  |
| Small melons | IL8 - 3 | Heat | 8 | Heat | 2 | 1 | 3.03E+03 |  |  |
| Medium white | IL8 - 3 | Heat | 8 | Heat | 2 | 10 | 3.03E+04 |  |  |
| Small white | IL8 - 3 | Heat | 8 | Heat | 2 | 3 | 9.09E+03 |  |  |
| Medium white | IL4 - 1 | Untreated | 15 | Direct | 0 | 10 | 3.03E+02 | 142 | 4.30E+03 |
| Medium pink | IL4 - 1 | Untreated | 15 | Direct | 0 | 3 | 9.09E+01 |  |  |
| Medium yellow | IL4 - 1 | Untreated | 15 | Direct | 0 | 4 | 1.21E+02 |  |  |
| Small yellow | IL4 - 1 | Untreated | 15 | Direct | 0 | 5 | 1.52E+02 |  |  |
| Small white | IL4 - 1 | Untreated | 15 | Direct | 0 | 105 | 3.18E+03 |  |  |
| Small translucent | IL4 - 1 | Untreated | 15 | Direct | 0 | 15 | 4.55E+02 |  |  |
| Medium white | IL4 - 1 | Untreated | 15 | Heat | 0 | 8 | 2.42E+02 | 23 | 6.97E+02 |
| Pink | IL4 - 1 | Untreated | 15 | Heat | 0 | 1 | 3.03E+01 |  |  |
| Medium yellow | IL4 - 1 | Untreated | 15 | Heat | 0 | 7 | 2.12E+02 |  |  |
| Small melons | IL4 - 1 | Untreated | 15 | Heat | 0 | 2 | 6.06E+01 |  |  |
| Small white | IL4 - 1 | Untreated | 15 | Heat | 0 | 5 | 1.52E+02 |  |  |
| Medium yellow | IL4 - 2 | Untreated | 15 | Direct | 1 | 5 | 1.52E+03 | 20 | 6.06E+03 |
| Small white | IL4 - 2 | Untreated | 15 | Direct | 1 | 14 | 4.24E+03 |  |  |
| Small translucent | IL4 - 2 | Untreated | 15 | Direct | 1 | 1 | 3.03E+02 |  |  |
| Medium pink | IL4 - 2 | Untreated | 15 | Heat | 0 | 1 | 3.03E+01 | 43 | 1.30E+03 |
| Small yellow | IL4 - 2 | Untreated | 15 | Heat | 0 | 37 | 1.12E+03 |  |  |
| Small melons | IL4 - 2 | Untreated | 15 | Heat | 0 | 2 | 6.06E+01 |  |  |
| Medium white | IL4 - 2 | Untreated | 15 | Heat | 0 | 1 | 3.03E+01 |  |  |
| Small white | IL4 - 2 | Untreated | 15 | Heat | 0 | 2 | 6.06E+01 |  |  |
| Medium pink | IL4 - 3 | Untreated | 15 | Direct | 0 | 2 | 6.06E+01 | 175 | 5.30E+03 |
| Medium yellow | IL4 - 3 | Untreated | 15 | Direct | 0 | 7 | 2.12E+02 |  |  |
| Small yellow | IL4 - 3 | Untreated | 15 | Direct | 0 | 4 | 1.21E+02 |  |  |
| Medium white | IL4 - 3 | Untreated | 15 | Direct | 0 | 1 | 3.03E+01 |  |  |
| Small white | IL4 - 3 | Untreated | 15 | Direct | 0 | 158 | 4.79E+03 |  |  |
| Small translucent | IL4 - 3 | Untreated | 15 | Direct | 0 | 1 | 3.03E+01 |  |  |
| Translucent yellow | IL4 - 3 | Untreated | 15 | Direct | 0 | 2 | 6.06E+01 |  |  |
| Medium white | IL4 - 3 | Untreated | 15 | Heat | 0 | 5 | 1.52E+02 | 33 | 1.00E+03 |
| Medium pink | IL4 - 3 | Untreated | 15 | Heat | 0 | 2 | 6.06E+01 |  |  |
| Pink | IL4 - 3 | Untreated | 15 | Heat | 0 | 1 | 3.03E+01 |  |  |
| Medium yellow | IL4 - 3 | Untreated | 15 | Heat | 0 | 4 | 1.21E+02 |  |  |
| Small yellow | IL4 - 3 | Untreated | 15 | Heat | 0 | 17 | 5.15E+02 |  |  |
| Small melons | IL4 - 3 | Untreated | 15 | Heat | 0 | 2 | 6.06E+01 |  |  |
| Small translucent | IL4 - 3 | Untreated | 15 | Heat | 0 | 2 | 6.06E+01 |  |  |
| Small yellow | IL5 - 1 | Untreated | 15 | Direct | 2 | 1 | 3.03E+03 | 113 | 3.42E+05 |
| Medium white | IL5 - 1 | Untreated | 15 | Direct | 2 | 1 | 3.03E+03 |  |  |
| Small white | IL5 - 1 | Untreated | 15 | Direct | 2 | 7 | 2.12E+04 |  |  |
| Translucent yellow | IL5 - 1 | Untreated | 15 | Direct | 2 | 104 | 3.15E+05 |  |  |
| Medium white | IL5 - 1 | Untreated | 15 | Heat | 1 | 2 | 6.06E+02 | 79 | 2.39E+04 |
| Small yellow | IL5 - 1 | Untreated | 15 | Heat | 1 | 77 | 2.33E+04 |  |  |
| Medium white | IL5 - 2 | Untreated | 15 | Direct | 1 | 7 | 2.12E+03 | 72 | 2.18E+04 |
| Small yellow | IL5 - 2 | Untreated | 15 | Direct | 1 | 10 | 3.03E+03 |  |  |
| Small white | IL5 - 2 | Untreated | 15 | Direct | 1 | 55 | 1.67E+04 |  |  |
| Medium pink | IL5 - 2 | Untreated | 15 | Heat | 0 | 1 | 3.03E+01 | 56 | 1.70E+03 |
| Small yellow | IL5 - 2 | Untreated | 15 | Heat | 0 | 25 | 7.58E+02 |  |  |
| Small melons | IL5 - 2 | Untreated | 15 | Heat | 0 | 5 | 1.52E+02 |  |  |
| Medium white | IL5 - 2 | Untreated | 15 | Heat | 0 | 1 | 3.03E+01 |  |  |
| Small white | IL5 - 2 | Untreated | 15 | Heat | 0 | 24 | 7.27E+02 |  |  |
| Small white | IL5 - 3 | Untreated | 15 | Direct | 2 | 8 | 2.42E+04 | 8 | 2.42E+04 |
| Medium white | IL5 - 3 | Untreated | 15 | Heat | 0 | 29 | 8.79E+02 | 68 | 2.06E+03 |
| Medium pink | IL5 - 3 | Untreated | 15 | Heat | 0 | 2 | 6.06E+01 |  |  |
| Small yellow | IL5 - 3 | Untreated | 15 | Heat | 0 | 14 | 4.24E+02 |  |  |
| Small white | IL5 - 3 | Untreated | 15 | Heat | 0 | 19 | 5.76E+02 |  |  |
| Small translucent | IL5 - 3 | Untreated | 15 | Heat | 0 | 4 | 1.21E+02 |  |  |
| Medium white | IL8 - 1 | Untreated | 15 | Direct | 0 | 7 | 2.12E+02 | 138 | 4.18E+03 |
| Medium pink | IL8 - 1 | Untreated | 15 | Direct | 0 | 10 | 3.03E+02 |  |  |
| Medium yellow | IL8 - 1 | Untreated | 15 | Direct | 0 | 1 | 3.03E+01 |  |  |
| Small yellow | IL8 - 1 | Untreated | 15 | Direct | 0 | 16 | 4.85E+02 |  |  |
| Small white | IL8 - 1 | Untreated | 15 | Direct | 0 | 104 | 3.15E+03 |  |  |
| Medium pink | IL8 - 1 | Untreated | 15 | Heat | 0 | 5 | 1.52E+02 | 26 | 7.88E+02 |
| Medium yellow | IL8 - 1 | Untreated | 15 | Heat | 0 | 6 | 1.82E+02 |  |  |
| Small yellow | IL8 - 1 | Untreated | 15 | Heat | 0 | 2 | 6.06E+01 |  |  |
| Medium white | IL8 - 1 | Untreated | 15 | Heat | 0 | 7 | 2.12E+02 |  |  |
| Small white | IL8 - 1 | Untreated | 15 | Heat | 0 | 6 | 1.82E+02 |  |  |
| Medium pink | IL8 - 2 | Untreated | 15 | Direct | 0 | 7 | 2.12E+02 | 155 | 4.70E+03 |
| Medium yellow | IL8 - 2 | Untreated | 15 | Direct | 0 | 17 | 5.15E+02 |  |  |
| Medium white | IL8 - 2 | Untreated | 15 | Direct | 0 | 13 | 3.94E+02 |  |  |
| Small white | IL8 - 2 | Untreated | 15 | Direct | 0 | 58 | 1.76E+03 |  |  |
| Small translucent | IL8 - 2 | Untreated | 15 | Direct | 0 | 6 | 1.82E+02 |  |  |
| Translucent yellow | IL8 - 2 | Untreated | 15 | Direct | 0 | 54 | 1.64E+03 |  |  |
| Medium pink | IL8 - 2 | Untreated | 15 | Heat | 0 | 1 | 3.03E+01 | 16 | 4.85E+02 |
| Pink | IL8 - 2 | Untreated | 15 | Heat | 0 | 5 | 1.52E+02 |  |  |
| Medium yellow | IL8 - 2 | Untreated | 15 | Heat | 0 | 2 | 6.06E+01 |  |  |
| Small yellow | IL8 - 2 | Untreated | 15 | Heat | 0 | 2 | 6.06E+01 |  |  |
| Small melons | IL8 - 2 | Untreated | 15 | Heat | 0 | 4 | 1.21E+02 |  |  |
| Medium white | IL8 - 2 | Untreated | 15 | Heat | 0 | 1 | 3.03E+01 |  |  |
| Small white | IL8 - 2 | Untreated | 15 | Heat | 0 | 1 | 3.03E+01 |  |  |
| Medium pink | IL8 - 3 | Untreated | 15 | Direct | 0 | 17 | 5.15E+02 | 93 | 2.82E+03 |
| Medium yellow | IL8 - 3 | Untreated | 15 | Direct | 0 | 14 | 4.24E+02 |  |  |
| Small yellow | IL8 - 3 | Untreated | 15 | Direct | 0 | 5 | 1.52E+02 |  |  |
| Medium white | IL8 - 3 | Untreated | 15 | Direct | 0 | 14 | 4.24E+02 |  |  |
| Small white | IL8 - 3 | Untreated | 15 | Direct | 0 | 31 | 9.39E+02 |  |  |
| Small translucent | IL8 - 3 | Untreated | 15 | Direct | 0 | 12 | 3.64E+02 |  |  |
| Medium pink | IL8 - 3 | Untreated | 15 | Heat | 0 | 10 | 3.03E+02 | 25 | 7.58E+02 |
| Small yellow | IL8 - 3 | Untreated | 15 | Heat | 0 | 12 | 3.64E+02 |  |  |
| Small white | IL8 - 3 | Untreated | 15 | Heat | 0 | 3 | 9.09E+01 |  |  |
| Pink | IL4 - 1 | Heat | 15 | Direct | 3 | 4 | 1.21E+05 | 55 | 1.67E+06 |
| Medium yellow | IL4 - 1 | Heat | 15 | Direct | 3 | 40 | 1.21E+06 |  |  |
| Medium white | IL4 - 1 | Heat | 15 | Direct | 3 | 11 | 3.33E+05 |  |  |
| Medium pink | IL4 - 1 | Heat | 15 | Heat | 2 | 11 | 3.33E+04 | 159 | 4.82E+05 |
| Medium yellow | IL4 - 1 | Heat | 15 | Heat | 2 | 48 | 1.45E+05 |  |  |
| Medium white | IL4 - 1 | Heat | 15 | Heat | 2 | 48 | 1.45E+05 |  |  |
| Medium oranges | IL4 - 1 | Heat | 15 | Heat | 2 | 52 | 1.58E+05 |  |  |
| Small yellow | IL4 - 2 | Heat | 15 | Direct | 2 | 115 | 3.48E+05 | 208 | 6.30E+05 |
| Small white | IL4 - 2 | Heat | 15 | Direct | 2 | 93 | 2.82E+05 |  |  |
| Pink | IL4 - 2 | Heat | 15 | Heat | 2 | 4 | 1.21E+04 | 114 | 3.45E+05 |
| Medium yellow | IL4 - 2 | Heat | 15 | Heat | 2 | 14 | 4.24E+04 |  |  |
| Small yellow | IL4 - 2 | Heat | 15 | Heat | 2 | 8 | 2.42E+04 |  |  |
| Small white | IL4 - 2 | Heat | 15 | Heat | 2 | 88 | 2.67E+05 |  |  |
| Pink | IL4 -3 | Heat | 15 | Direct | 2 | 72 | 2.18E+05 | 200 | 6.06E+05 |
| Medium yellow | IL4 -3 | Heat | 15 | Direct | 2 | 52 | 1.58E+05 |  |  |
| Medium white | IL4 -3 | Heat | 15 | Direct | 2 | 68 | 2.06E+05 |  |  |
| Small white | IL4 -3 | Heat | 15 | Direct | 2 | 8 | 2.42E+04 |  |  |
| Small yellow | IL4 -3 | Heat | 15 | Heat | 2 | 60 | 1.82E+05 | 176 | 5.33E+05 |
| Small white | IL4 -3 | Heat | 15 | Heat | 2 | 32 | 9.70E+04 |  |  |
| Small translucent | IL4 -3 | Heat | 15 | Heat | 2 | 84 | 2.55E+05 |  |  |
| Pink | IL5 - 1 | Heat | 15 | Direct | 3 | 15 | 4.55E+05 | 67 | 2.03E+06 |
| Medium yellow | IL5 - 1 | Heat | 15 | Direct | 3 | 28 | 8.48E+05 |  |  |
| Small white | IL5 - 1 | Heat | 15 | Direct | 3 | 22 | 6.67E+05 |  |  |
| Medium oranges | IL5 - 1 | Heat | 15 | Direct | 3 | 2 | 6.06E+04 |  |  |
| Pink | IL5 - 1 | Heat | 15 | Heat | 3 | 9 | 2.73E+05 | 47 | 1.42E+06 |
| Medium yellow | IL5 - 1 | Heat | 15 | Heat | 3 | 9 | 2.73E+05 |  |  |
| Medium white | IL5 - 1 | Heat | 15 | Heat | 3 | 24 | 7.27E+05 |  |  |
| Small white | IL5 - 1 | Heat | 15 | Heat | 3 | 3 | 9.09E+04 |  |  |
| Medium translucent | IL5 - 1 | Heat | 15 | Heat | 3 | 2 | 6.06E+04 |  |  |
| Pink | IL5 - 2 | Heat | 15 | Direct | 3 | 7 | 2.12E+05 | 39 | 1.18E+06 |
| Medium yellow | IL5 - 2 | Heat | 15 | Direct | 3 | 7 | 2.12E+05 |  |  |
| Medium white | IL5 - 2 | Heat | 15 | Direct | 3 | 13 | 3.94E+05 |  |  |
| Small white | IL5 - 2 | Heat | 15 | Direct | 3 | 12 | 3.64E+05 |  |  |
| Medium white | IL5 - 2 | Heat | 15 | Heat | 2 | 2 | 6.06E+03 | 82 | 2.48E+05 |
| Pink | IL5 - 2 | Heat | 15 | Heat | 2 | 18 | 5.45E+04 |  |  |
| Small yellow | IL5 - 2 | Heat | 15 | Heat | 2 | 36 | 1.09E+05 |  |  |
| Small white | IL5 - 2 | Heat | 15 | Heat | 2 | 26 | 7.88E+04 |  |  |
| Pink | IL5 - 3 | Heat | 15 | Direct | 3 | 2 | 6.06E+04 | 62 | 1.88E+06 |
| Medium yellow | IL5 - 3 | Heat | 15 | Direct | 3 | 16 | 4.85E+05 |  |  |
| Medium white | IL5 - 3 | Heat | 15 | Direct | 3 | 24 | 7.27E+05 |  |  |
| Small white | IL5 - 3 | Heat | 15 | Direct | 3 | 20 | 6.06E+05 |  |  |
| Pink | IL5 - 3 | Heat | 15 | Heat | 2 | 29 | 8.79E+04 | 70 | 2.12E+05 |
| Medium yellow | IL5 - 3 | Heat | 15 | Heat | 2 | 33 | 1.00E+05 |  |  |
| Small white | IL5 - 3 | Heat | 15 | Heat | 2 | 8 | 2.42E+04 |  |  |
| Pink | IL8 - 1 | Heat | 15 | Direct | 2 | 3 | 9.09E+03 | 79 | 2.39E+05 |
| Medium yellow | IL8 - 1 | Heat | 15 | Direct | 2 | 54 | 1.64E+05 |  |  |
| Medium white | IL8 - 1 | Heat | 15 | Direct | 2 | 4 | 1.21E+04 |  |  |
| Small white | IL8 - 1 | Heat | 15 | Direct | 2 | 17 | 5.15E+04 |  |  |
| Medium translucent | IL8 - 1 | Heat | 15 | Direct | 2 | 1 | 3.03E+03 |  |  |
| Pink | IL8 - 1 | Heat | 15 | Heat | 2 | 3 | 9.09E+03 | 81 | 2.45E+05 |
| Medium yellow | IL8 - 1 | Heat | 15 | Heat | 2 | 33 | 1.00E+05 |  |  |
| Small white | IL8 - 1 | Heat | 15 | Heat | 2 | 45 | 1.36E+05 |  |  |
| Small yellow | IL8 - 2 | Heat | 15 | Direct | 2 | 92 | 2.79E+05 | 280 | 8.48E+05 |
| Small white | IL8 - 2 | Heat | 15 | Direct | 2 | 188 | 5.70E+05 |  |  |
| Medium yellow | IL8 - 2 | Heat | 15 | Heat | 2 | 50 | 1.52E+05 | 125 | 3.79E+05 |
| Small yellow | IL8 - 2 | Heat | 15 | Heat | 2 | 10 | 3.03E+04 |  |  |
| Medium white | IL8 - 2 | Heat | 15 | Heat | 2 | 55 | 1.67E+05 |  |  |
| Small white | IL8 - 2 | Heat | 15 | Heat | 2 | 10 | 3.03E+04 |  |  |
| Small yellow | IL8 - 3 | Heat | 15 | Direct | 2 | 83 | 2.52E+05 | 218 | 6.61E+05 |
| Small white | IL8 - 3 | Heat | 15 | Direct | 2 | 55 | 1.67E+05 |  |  |
| Small translucent | IL8 - 3 | Heat | 15 | Direct | 2 | 80 | 2.42E+05 |  |  |
| Medium white | IL8 - 3 | Heat | 15 | Heat | 2 | 34 | 1.03E+05 | 89 | 2.70E+05 |
| Medium yellow | IL8 - 3 | Heat | 15 | Heat | 2 | 5 | 1.52E+04 |  |  |
| Small yellow | IL8 - 3 | Heat | 15 | Heat | 2 | 24 | 7.27E+04 |  |  |
| Small melons | IL8 - 3 | Heat | 15 | Heat | 2 | 1 | 3.03E+03 |  |  |
| Small white | IL8 - 3 | Heat | 15 | Heat | 2 | 25 | 7.58E+04 |  |  |
| Medium yellow | IL4 - 1 | Untreated | 32 | Direct | 0 | 4 | 1.21E+02 | 168 | 5.09E+03 |
| Small yellow | IL4 - 1 | Untreated | 32 | Direct | 0 | 17 | 5.15E+02 |  |  |
| Small white | IL4 - 1 | Untreated | 32 | Direct | 0 | 34 | 1.03E+03 |  |  |
| Medium beige | IL4 - 1 | Untreated | 32 | Direct | 0 | 11 | 3.33E+02 |  |  |
| Small beige | IL4 - 1 | Untreated | 32 | Direct | 0 | 50 | 1.52E+03 |  |  |
| Medium melons | IL4 - 1 | Untreated | 32 | Direct | 0 | 5 | 1.52E+02 |  |  |
| Small melons | IL4 - 1 | Untreated | 32 | Direct | 0 | 16 | 4.85E+02 |  |  |
| Medium oranges | IL4 - 1 | Untreated | 32 | Direct | 0 | 18 | 5.45E+02 |  |  |
| Small red | IL4 - 1 | Untreated | 32 | Direct | 0 | 4 | 1.21E+02 |  |  |
| Small pink | IL4 - 1 | Untreated | 32 | Direct | 0 | 9 | 2.73E+02 |  |  |
| Medium yellow | IL4 - 1 | Untreated | 32 | Heat | 0 | 1 | 3.03E+01 | 3 | 9.09E+01 |
| Medium white | IL4 - 1 | Untreated | 32 | Heat | 0 | 1 | 3.03E+01 |  |  |
| Medium oranges | IL4 - 1 | Untreated | 32 | Heat | 0 | 1 | 3.03E+01 |  |  |
| Medium white | IL4 - 2 | Untreated | 32 | Direct | 0 | 3 | 9.09E+01 | 95 | 2.88E+03 |
| Medium beige | IL4 - 2 | Untreated | 32 | Direct | 0 | 28 | 8.48E+02 |  |  |
| Medium melons | IL4 - 2 | Untreated | 32 | Direct | 0 | 1 | 3.03E+01 |  |  |
| Small melons | IL4 - 2 | Untreated | 32 | Direct | 0 | 54 | 1.64E+03 |  |  |
| Medium oranges | IL4 - 2 | Untreated | 32 | Direct | 0 | 9 | 2.73E+02 |  |  |
| Medium beige | IL4 - 2 | Untreated | 32 | Heat | 0 | 1 | 3.03E+01 | 19 | 5.76E+02 |
| Medium melons | IL4 - 2 | Untreated | 32 | Heat | 0 | 1 | 3.03E+01 |  |  |
| Small melons | IL4 - 2 | Untreated | 32 | Heat | 0 | 1 | 3.03E+01 |  |  |
| Medium oranges | IL4 - 2 | Untreated | 32 | Heat | 0 | 16 | 4.85E+02 |  |  |
| Yellow cottony | IL4 - 3 | Untreated | 32 | Direct | 2 | 19 | 5.76E+04 | 110 | 3.33E+05 |
| Small yellow | IL4 - 3 | Untreated | 32 | Direct | 2 | 48 | 1.45E+05 |  |  |
| Medium white | IL4 - 3 | Untreated | 32 | Direct | 2 | 4 | 1.21E+04 |  |  |
| Small white | IL4 - 3 | Untreated | 32 | Direct | 2 | 6 | 1.82E+04 |  |  |
| Medium beige | IL4 - 3 | Untreated | 32 | Direct | 2 | 2 | 6.06E+03 |  |  |
| Small beige | IL4 - 3 | Untreated | 32 | Direct | 2 | 17 | 5.15E+04 |  |  |
| Small melons | IL4 - 3 | Untreated | 32 | Direct | 2 | 4 | 1.21E+04 |  |  |
| Small red | IL4 - 3 | Untreated | 32 | Direct | 2 | 10 | 3.03E+04 |  |  |
| Medium yellow | IL4 - 3 | Untreated | 32 | Heat | 0 | 1 | 3.03E+01 | 25 | 7.58E+02 |
| Small yellow | IL4 - 3 | Untreated | 32 | Heat | 0 | 17 | 5.15E+02 |  |  |
| Medium white | IL4 - 3 | Untreated | 32 | Heat | 0 | 1 | 3.03E+01 |  |  |
| Beige | IL4 - 3 | Untreated | 32 | Heat | 0 | 4 | 1.21E+02 |  |  |
| Medium melons | IL4 - 3 | Untreated | 32 | Heat | 0 | 1 | 3.03E+01 |  |  |
| Medium oranges | IL4 - 3 | Untreated | 32 | Heat | 0 | 1 | 3.03E+01 |  |  |
| Medium yellow | IL5 - 1 | Untreated | 32 | Direct | 2 | 55 | 1.67E+05 | 274 | 8.30E+05 |
| Small yellow | IL5 - 1 | Untreated | 32 | Direct | 2 | 12 | 3.64E+04 |  |  |
| Small white | IL5 - 1 | Untreated | 32 | Direct | 2 | 7 | 2.12E+04 |  |  |
| Medium beige | IL5 - 1 | Untreated | 32 | Direct | 2 | 1 | 3.03E+03 |  |  |
| Medium melons | IL5 - 1 | Untreated | 32 | Direct | 2 | 14 | 4.24E+04 |  |  |
| Small translucent | IL5 - 1 | Untreated | 32 | Direct | 2 | 67 | 2.03E+05 |  |  |
| Small red | IL5 - 1 | Untreated | 32 | Direct | 2 | 5 | 1.52E+04 |  |  |
| Small pink | IL5 - 1 | Untreated | 32 | Direct | 2 | 105 | 3.18E+05 |  |  |
| Red | IL5 - 1 | Untreated | 32 | Direct | 2 | 8 | 2.42E+04 |  |  |
| Medium yellow | IL5 - 1 | Untreated | 32 | Heat | 0 | 3 | 9.09E+01 | 18 | 5.45E+02 |
| Small yellow | IL5 - 1 | Untreated | 32 | Heat | 0 | 2 | 6.06E+01 |  |  |
| Medium white | IL5 - 1 | Untreated | 32 | Heat | 0 | 3 | 9.09E+01 |  |  |
| Beige | IL5 - 1 | Untreated | 32 | Heat | 0 | 6 | 1.82E+02 |  |  |
| Medium oranges | IL5 - 1 | Untreated | 32 | Heat | 0 | 3 | 9.09E+01 |  |  |
| Pink | IL5 - 1 | Untreated | 32 | Heat | 0 | 1 | 3.03E+01 |  |  |
| Yellow cottony | IL5 - 2 | Untreated | 32 | Direct | 1 | 2 | 6.06E+02 | 75 | 2.27E+04 |
| Small yellow | IL5 - 2 | Untreated | 32 | Direct | 1 | 11 | 3.33E+03 |  |  |
| Medium white | IL5 - 2 | Untreated | 32 | Direct | 1 | 1 | 3.03E+02 |  |  |
| Medium beige | IL5 - 2 | Untreated | 32 | Direct | 1 | 3 | 9.09E+02 |  |  |
| Small beige | IL5 - 2 | Untreated | 32 | Direct | 1 | 11 | 3.33E+03 |  |  |
| Small melons | IL5 - 2 | Untreated | 32 | Direct | 1 | 36 | 1.09E+04 |  |  |
| Medium oranges | IL5 - 2 | Untreated | 32 | Direct | 1 | 7 | 2.12E+03 |  |  |
| Small red | IL5 - 2 | Untreated | 32 | Direct | 1 | 4 | 1.21E+03 |  |  |
| Medium melons | IL5 - 2 | Untreated | 32 | Heat | 0 | 1 | 3.03E+01 | 23 | 6.97E+02 |
| Medium oranges | IL5 - 2 | Untreated | 32 | Heat | 0 | 22 | 6.67E+02 |  |  |
| Medium yellow | IL5 - 3 | Untreated | 32 | Direct | 1 | 3 | 9.09E+02 | 87 | 2.64E+04 |
| Medium white | IL5 - 3 | Untreated | 32 | Direct | 1 | 1 | 3.03E+02 |  |  |
| Small white | IL5 - 3 | Untreated | 32 | Direct | 1 | 4 | 1.21E+03 |  |  |
| Medium beige | IL5 - 3 | Untreated | 32 | Direct | 1 | 48 | 1.45E+04 |  |  |
| Small beige | IL5 - 3 | Untreated | 32 | Direct | 1 | 9 | 2.73E+03 |  |  |
| Medium melons | IL5 - 3 | Untreated | 32 | Direct | 1 | 1 | 3.03E+02 |  |  |
| Small melons | IL5 - 3 | Untreated | 32 | Direct | 1 | 1 | 3.03E+02 |  |  |
| Medium oranges | IL5 - 3 | Untreated | 32 | Direct | 1 | 20 | 6.06E+03 |  |  |
| Medium yellow | IL5 - 3 | Untreated | 32 | Heat | 1 | 2 | 6.06E+02 | 6 | 1.82E+03 |
| Small yellow | IL5 - 3 | Untreated | 32 | Heat | 1 | 1 | 3.03E+02 |  |  |
| Medium oranges | IL5 - 3 | Untreated | 32 | Heat | 1 | 3 | 9.09E+02 |  |  |
| Medium yellow | IL8 - 1 | Untreated | 32 | Direct | 1 | 4 | 1.21E+03 | 54 | 1.64E+04 |
| Medium white | IL8 - 1 | Untreated | 32 | Direct | 1 | 1 | 3.03E+02 |  |  |
| Beige | IL8 - 1 | Untreated | 32 | Direct | 1 | 1 | 3.03E+02 |  |  |
| Medium beige | IL8 - 1 | Untreated | 32 | Direct | 1 | 26 | 7.88E+03 |  |  |
| Medium oranges | IL8 - 1 | Untreated | 32 | Direct | 1 | 3 | 9.09E+02 |  |  |
| Medium translucent | IL8 - 1 | Untreated | 32 | Direct | 1 | 1 | 3.03E+02 |  |  |
| Small red | IL8 - 1 | Untreated | 32 | Direct | 1 | 18 | 5.45E+03 |  |  |
| Beige | IL8 - 1 | Untreated | 32 | Heat | 0 | 2 | 6.06E+01 | 6 | 1.82E+02 |
| Medium oranges | IL8 - 1 | Untreated | 32 | Heat | 0 | 2 | 6.06E+01 |  |  |
| Medium pink | IL8 - 1 | Untreated | 32 | Heat | 0 | 2 | 6.06E+01 |  |  |
| Medium yellow | IL8 - 2 | Untreated | 32 | Direct | 0 | 44 | 1.33E+03 | 108 | 3.27E+03 |
| Medium white | IL8 - 2 | Untreated | 32 | Direct | 0 | 12 | 3.64E+02 |  |  |
| Small white | IL8 - 2 | Untreated | 32 | Direct | 0 | 16 | 4.85E+02 |  |  |
| Medium melons | IL8 - 2 | Untreated | 32 | Direct | 0 | 8 | 2.42E+02 |  |  |
| Small melons | IL8 - 2 | Untreated | 32 | Direct | 0 | 11 | 3.33E+02 |  |  |
| Medium oranges | IL8 - 2 | Untreated | 32 | Direct | 0 | 16 | 4.85E+02 |  |  |
| Medium translucent | IL8 - 2 | Untreated | 32 | Direct | 0 | 1 | 3.03E+01 |  |  |
| Small yellow | IL8 - 2 | Untreated | 32 | Heat | 0 | 21 | 6.36E+02 | 36 | 1.09E+03 |
| Medium white | IL8 - 2 | Untreated | 32 | Heat | 0 | 10 | 3.03E+02 |  |  |
| Small white | IL8 - 2 | Untreated | 32 | Heat | 0 | 1 | 3.03E+01 |  |  |
| Beige | IL8 - 2 | Untreated | 32 | Heat | 0 | 1 | 3.03E+01 |  |  |
| Medium beige | IL8 - 2 | Untreated | 32 | Heat | 0 | 2 | 6.06E+01 |  |  |
| Small melons | IL8 - 2 | Untreated | 32 | Heat | 0 | 1 | 3.03E+01 |  |  |
| Medium yellow | IL8 - 3 | Untreated | 32 | Direct | 0 | 33 | 1.00E+03 | 206 | 6.24E+03 |
| Small yellow | IL8 - 3 | Untreated | 32 | Direct | 0 | 15 | 4.55E+02 |  |  |
| Medium white | IL8 - 3 | Untreated | 32 | Direct | 0 | 14 | 4.24E+02 |  |  |
| Medium beige | IL8 - 3 | Untreated | 32 | Direct | 0 | 63 | 1.91E+03 |  |  |
| Small beige | IL8 - 3 | Untreated | 32 | Direct | 0 | 33 | 1.00E+03 |  |  |
| Medium melons | IL8 - 3 | Untreated | 32 | Direct | 0 | 1 | 3.03E+01 |  |  |
| Medium oranges | IL8 - 3 | Untreated | 32 | Direct | 0 | 30 | 9.09E+02 |  |  |
| Small translucent | IL8 - 3 | Untreated | 32 | Direct | 0 | 17 | 5.15E+02 |  |  |
| Medium beige | IL8 - 3 | Untreated | 32 | Heat | 0 | 4 | 1.21E+02 | 9 | 2.73E+02 |
| Medium oranges | IL8 - 3 | Untreated | 32 | Heat | 0 | 3 | 9.09E+01 |  |  |
| Medium pink | IL8 - 3 | Untreated | 32 | Heat | 0 | 2 | 6.06E+01 |  |  |
| Medium yellow | IL4 - 1 | Heat | 32 | Direct | 2 | 7 | 2.12E+04 | 192 | 5.82E+05 |
| Medium white | IL4 - 1 | Heat | 32 | Direct | 2 | 10 | 3.03E+04 |  |  |
| Medium beige | IL4 - 1 | Heat | 32 | Direct | 2 | 16 | 4.85E+04 |  |  |
| Medium melons | IL4 - 1 | Heat | 32 | Direct | 2 | 24 | 7.27E+04 |  |  |
| Medium oranges | IL4 - 1 | Heat | 32 | Direct | 2 | 128 | 3.88E+05 |  |  |
| Pink | IL4 - 1 | Heat | 32 | Direct | 2 | 6 | 1.82E+04 |  |  |
| Translucent yellow | IL4 - 1 | Heat | 32 | Direct | 2 | 1 | 3.03E+03 |  |  |
| Medium yellow | IL4 - 1 | Heat | 32 | Heat | 2 | 10 | 3.03E+04 | 117 | 3.55E+05 |
| Beige | IL4 - 1 | Heat | 32 | Heat | 2 | 19 | 5.76E+04 |  |  |
| Medium melons | IL4 - 1 | Heat | 32 | Heat | 2 | 4 | 1.21E+04 |  |  |
| Medium oranges | IL4 - 1 | Heat | 32 | Heat | 2 | 80 | 2.42E+05 |  |  |
| Pink | IL4 - 1 | Heat | 32 | Heat | 2 | 4 | 1.21E+04 |  |  |
| Small white | IL4 - 2 | Heat | 32 | Direct | 2 | 18 | 5.45E+04 | 158 | 4.79E+05 |
| Medium beige | IL4 - 2 | Heat | 32 | Direct | 2 | 50 | 1.52E+05 |  |  |
| Medium oranges | IL4 - 2 | Heat | 32 | Direct | 2 | 81 | 2.45E+05 |  |  |
| Pink | IL4 - 2 | Heat | 32 | Direct | 2 | 1 | 3.03E+03 |  |  |
| Medium translucent | IL4 - 2 | Heat | 32 | Direct | 2 | 8 | 2.42E+04 |  |  |
| Medium yellow | IL4 - 2 | Heat | 32 | Heat | 2 | 53 | 1.61E+05 | 90 | 2.73E+05 |
| Medium white | IL4 - 2 | Heat | 32 | Heat | 2 | 1 | 3.03E+03 |  |  |
| Small beige | IL4 - 2 | Heat | 32 | Heat | 2 | 9 | 2.73E+04 |  |  |
| Medium oranges | IL4 - 2 | Heat | 32 | Heat | 2 | 24 | 7.27E+04 |  |  |
| Pink | IL4 - 2 | Heat | 32 | Heat | 2 | 3 | 9.09E+03 |  |  |
| Medium yellow | IL4 -3 | Heat | 32 | Direct | 2 | 4 | 1.21E+04 | 123 | 3.73E+05 |
| Small white | IL4 -3 | Heat | 32 | Direct | 2 | 2 | 6.06E+03 |  |  |
| Medium melons | IL4 -3 | Heat | 32 | Direct | 2 | 6 | 1.82E+04 |  |  |
| Medium oranges | IL4 -3 | Heat | 32 | Direct | 2 | 73 | 2.21E+05 |  |  |
| Pink | IL4 -3 | Heat | 32 | Direct | 2 | 2 | 6.06E+03 |  |  |
| Translucent yellow | IL4 -3 | Heat | 32 | Direct | 2 | 29 | 8.79E+04 |  |  |
| Medium translucent | IL4 -3 | Heat | 32 | Direct | 2 | 7 | 2.12E+04 |  |  |
| Medium yellow | IL4 -3 | Heat | 32 | Heat | 2 | 21 | 6.36E+04 | 107 | 3.24E+05 |
| Beige | IL4 -3 | Heat | 32 | Heat | 2 | 7 | 2.12E+04 |  |  |
| Small beige | IL4 -3 | Heat | 32 | Heat | 2 | 8 | 2.42E+04 |  |  |
| Small melons | IL4 -3 | Heat | 32 | Heat | 2 | 2 | 6.06E+03 |  |  |
| Medium oranges | IL4 -3 | Heat | 32 | Heat | 2 | 63 | 1.91E+05 |  |  |
| Pink | IL4 -3 | Heat | 32 | Heat | 2 | 6 | 1.82E+04 |  |  |
| Medium yellow | IL5 - 1 | Heat | 32 | Direct | 2 | 5 | 1.52E+04 | 72 | 2.18E+05 |
| Medium white | IL5 - 1 | Heat | 32 | Direct | 2 | 10 | 3.03E+04 |  |  |
| Medium beige | IL5 - 1 | Heat | 32 | Direct | 2 | 14 | 4.24E+04 |  |  |
| Small beige | IL5 - 1 | Heat | 32 | Direct | 2 | 5 | 1.52E+04 |  |  |
| Medium melons | IL5 - 1 | Heat | 32 | Direct | 2 | 5 | 1.52E+04 |  |  |
| Medium oranges | IL5 - 1 | Heat | 32 | Direct | 2 | 10 | 3.03E+04 |  |  |
| Pink | IL5 - 1 | Heat | 32 | Direct | 2 | 11 | 3.33E+04 |  |  |
| Medium translucent | IL5 - 1 | Heat | 32 | Direct | 2 | 7 | 2.12E+04 |  |  |
| Translucent beige | IL5 - 1 | Heat | 32 | Direct | 2 | 5 | 1.52E+04 |  |  |
| Medium yellow | IL5 - 1 | Heat | 32 | Heat | 2 | 2 | 6.06E+03 | 21 | 6.36E+04 |
| Beige | IL5 - 1 | Heat | 32 | Heat | 2 | 6 | 1.82E+04 |  |  |
| Medium beige | IL5 - 1 | Heat | 32 | Heat | 2 | 1 | 3.03E+03 |  |  |
| Medium oranges | IL5 - 1 | Heat | 32 | Heat | 2 | 2 | 6.06E+03 |  |  |
| Pink | IL5 - 1 | Heat | 32 | Heat | 2 | 8 | 2.42E+04 |  |  |
| Translucent yellow | IL5 - 1 | Heat | 32 | Heat | 2 | 1 | 3.03E+03 |  |  |
| Large translucent | IL5 - 1 | Heat | 32 | Heat | 2 | 1 | 3.03E+03 |  |  |
| Yellow cottony | IL5 - 2 | Heat | 32 | Direct | 2 | 1 | 3.03E+03 | 113 | 3.42E+05 |
| Medium yellow | IL5 - 2 | Heat | 32 | Direct | 2 | 4 | 1.21E+04 |  |  |
| Medium white | IL5 - 2 | Heat | 32 | Direct | 2 | 3 | 9.09E+03 |  |  |
| Small white | IL5 - 2 | Heat | 32 | Direct | 2 | 5 | 1.52E+04 |  |  |
| Medium beige | IL5 - 2 | Heat | 32 | Direct | 2 | 21 | 6.36E+04 |  |  |
| Small beige | IL5 - 2 | Heat | 32 | Direct | 2 | 18 | 5.45E+04 |  |  |
| Medium melons | IL5 - 2 | Heat | 32 | Direct | 2 | 2 | 6.06E+03 |  |  |
| Medium oranges | IL5 - 2 | Heat | 32 | Direct | 2 | 13 | 3.94E+04 |  |  |
| Pink | IL5 - 2 | Heat | 32 | Direct | 2 | 42 | 1.27E+05 |  |  |
| Medium translucent | IL5 - 2 | Heat | 32 | Direct | 2 | 3 | 9.09E+03 |  |  |
| Small red | IL5 - 2 | Heat | 32 | Direct | 2 | 1 | 3.03E+03 |  |  |
| Medium yellow | IL5 - 2 | Heat | 32 | Heat | 2 | 23 | 6.97E+04 | 130 | 3.94E+05 |
| Beige | IL5 - 2 | Heat | 32 | Heat | 2 | 5 | 1.52E+04 |  |  |
| Medium beige | IL5 - 2 | Heat | 32 | Heat | 2 | 1 | 3.03E+03 |  |  |
| Medium melons | IL5 - 2 | Heat | 32 | Heat | 2 | 6 | 1.82E+04 |  |  |
| Medium oranges | IL5 - 2 | Heat | 32 | Heat | 2 | 95 | 2.88E+05 |  |  |
| Medium yellow | IL5 - 3 | Heat | 32 | Direct | 2 | 9 | 2.73E+04 | 144 | 4.36E+05 |
| Small yellow | IL5 - 3 | Heat | 32 | Direct | 2 | 5 | 1.52E+04 |  |  |
| Small white | IL5 - 3 | Heat | 32 | Direct | 2 | 14 | 4.24E+04 |  |  |
| Beige | IL5 - 3 | Heat | 32 | Direct | 2 | 8 | 2.42E+04 |  |  |
| Medium melons | IL5 - 3 | Heat | 32 | Direct | 2 | 2 | 6.06E+03 |  |  |
| Medium oranges | IL5 - 3 | Heat | 32 | Direct | 2 | 66 | 2.00E+05 |  |  |
| Pink | IL5 - 3 | Heat | 32 | Direct | 2 | 40 | 1.21E+05 |  |  |
| Medium yellow | IL5 - 3 | Heat | 32 | Heat | 2 | 51 | 1.55E+05 | 118 | 3.58E+05 |
| Small white | IL5 - 3 | Heat | 32 | Heat | 2 | 5 | 1.52E+04 |  |  |
| Beige | IL5 - 3 | Heat | 32 | Heat | 2 | 7 | 2.12E+04 |  |  |
| Medium beige | IL5 - 3 | Heat | 32 | Heat | 2 | 6 | 1.82E+04 |  |  |
| Medium oranges | IL5 - 3 | Heat | 32 | Heat | 2 | 14 | 4.24E+04 |  |  |
| Pink | IL5 - 3 | Heat | 32 | Heat | 2 | 35 | 1.06E+05 |  |  |
| Small white | IL8 - 1 | Heat | 32 | Direct | 3 | 5 | 1.52E+05 | 29 | 8.79E+05 |
| Beige | IL8 - 1 | Heat | 32 | Direct | 3 | 4 | 1.21E+05 |  |  |
| Medium melons | IL8 - 1 | Heat | 32 | Direct | 3 | 1 | 3.03E+04 |  |  |
| Medium oranges | IL8 - 1 | Heat | 32 | Direct | 3 | 18 | 5.45E+05 |  |  |
| Pink | IL8 - 1 | Heat | 32 | Direct | 3 | 1 | 3.03E+04 |  |  |
| Medium yellow | IL8 - 1 | Heat | 32 | Heat | 2 | 18 | 5.45E+04 | 67 | 2.03E+05 |
| Small white | IL8 - 1 | Heat | 32 | Heat | 2 | 3 | 9.09E+03 |  |  |
| Beige | IL8 - 1 | Heat | 32 | Heat | 2 | 8 | 2.42E+04 |  |  |
| Medium beige | IL8 - 1 | Heat | 32 | Heat | 2 | 2 | 6.06E+03 |  |  |
| Medium oranges | IL8 - 1 | Heat | 32 | Heat | 2 | 8 | 2.42E+04 |  |  |
| Pink | IL8 - 1 | Heat | 32 | Heat | 2 | 28 | 8.48E+04 |  |  |
| Medium yellow | IL8 - 2 | Heat | 32 | Direct | 2 | 16 | 4.85E+04 | 117 | 3.55E+05 |
| Medium white | IL8 - 2 | Heat | 32 | Direct | 2 | 1 | 3.03E+03 |  |  |
| Small beige | IL8 - 2 | Heat | 32 | Direct | 2 | 20 | 6.06E+04 |  |  |
| Medium melons | IL8 - 2 | Heat | 32 | Direct | 2 | 8 | 2.42E+04 |  |  |
| Medium oranges | IL8 - 2 | Heat | 32 | Direct | 2 | 66 | 2.00E+05 |  |  |
| Medium translucent | IL8 - 2 | Heat | 32 | Direct | 2 | 4 | 1.21E+04 |  |  |
| Small translucent | IL8 - 2 | Heat | 32 | Direct | 2 | 2 | 6.06E+03 |  |  |
| Medium yellow | IL8 - 2 | Heat | 32 | Heat | 2 | 5 | 1.52E+04 | 95 | 2.88E+05 |
| Small white | IL8 - 2 | Heat | 32 | Heat | 2 | 4 | 1.21E+04 |  |  |
| Beige | IL8 - 2 | Heat | 32 | Heat | 2 | 11 | 3.33E+04 |  |  |
| Medium beige | IL8 - 2 | Heat | 32 | Heat | 2 | 2 | 6.06E+03 |  |  |
| Small beige | IL8 - 2 | Heat | 32 | Heat | 2 | 3 | 9.09E+03 |  |  |
| Medium melons | IL8 - 2 | Heat | 32 | Heat | 2 | 1 | 3.03E+03 |  |  |
| Medium oranges | IL8 - 2 | Heat | 32 | Heat | 2 | 67 | 2.03E+05 |  |  |
| Pink | IL8 - 2 | Heat | 32 | Heat | 2 | 2 | 6.06E+03 |  |  |
| Small yellow | IL8 - 3 | Heat | 32 | Direct | 2 | 5 | 1.52E+04 | 149 | 4.52E+05 |
| Medium white | IL8 - 3 | Heat | 32 | Direct | 2 | 3 | 9.09E+03 |  |  |
| Medium beige | IL8 - 3 | Heat | 32 | Direct | 2 | 39 | 1.18E+05 |  |  |
| Small beige | IL8 - 3 | Heat | 32 | Direct | 2 | 10 | 3.03E+04 |  |  |
| Medium melons | IL8 - 3 | Heat | 32 | Direct | 2 | 4 | 1.21E+04 |  |  |
| Medium oranges | IL8 - 3 | Heat | 32 | Direct | 2 | 82 | 2.48E+05 |  |  |
| Pink | IL8 - 3 | Heat | 32 | Direct | 2 | 4 | 1.21E+04 |  |  |
| Medium translucent | IL8 - 3 | Heat | 32 | Direct | 2 | 2 | 6.06E+03 |  |  |
| Medium yellow | IL8 - 3 | Heat | 32 | Heat | 2 | 1 | 3.03E+03 | 91 | 2.76E+05 |
| Small yellow | IL8 - 3 | Heat | 32 | Heat | 2 | 3 | 9.09E+03 |  |  |
| Beige | IL8 - 3 | Heat | 32 | Heat | 2 | 1 | 3.03E+03 |  |  |
| Medium beige | IL8 - 3 | Heat | 32 | Heat | 2 | 15 | 4.55E+04 |  |  |
| Medium melons | IL8 - 3 | Heat | 32 | Heat | 2 | 1 | 3.03E+03 |  |  |
| Small melons | IL8 - 3 | Heat | 32 | Heat | 2 | 1 | 3.03E+03 |  |  |
| Medium oranges | IL8 - 3 | Heat | 32 | Heat | 2 | 68 | 2.06E+05 |  |  |
| Pink | IL8 - 3 | Heat | 32 | Heat | 2 | 1 | 3.03E+03 |  |  |
| Medium yellow | IL4 - 1 | Untreated | 63 | Direct | 1 | 1 | 3.03E+02 | 32 | 9.70E+03 |
| Medium white | IL4 - 1 | Untreated | 63 | Direct | 1 | 12 | 3.64E+03 |  |  |
| Medium melons | IL4 - 1 | Untreated | 63 | Direct | 1 | 2 | 6.06E+02 |  |  |
| Medium oranges | IL4 - 1 | Untreated | 63 | Direct | 1 | 17 | 5.15E+03 |  |  |
| Small white | IL4 - 1 | Untreated | 63 | Heat | 1 | 1 | 3.03E+02 | 1 | 3.03E+02 |
| Small white | IL4 - 2 | Untreated | 63 | Direct | 1 | 45 | 1.36E+04 | 51 | 1.55E+04 |
| Medium oranges | IL4 - 2 | Untreated | 63 | Direct | 1 | 6 | 1.82E+03 |  |  |
| Medium white | IL4 - 2 | Untreated | 63 | Heat | 0 | 1 | 3.03E+01 | 31 | 9.39E+02 |
| Medium melons | IL4 - 2 | Untreated | 63 | Heat | 0 | 3 | 9.09E+01 |  |  |
| Black | IL4 - 2 | Untreated | 63 | Heat | 0 | 27 | 8.18E+02 |  |  |
| Small white | IL4 - 3 | Untreated | 63 | Direct | 1 | 2 | 6.06E+02 | 53 | 1.61E+04 |
| Small melons | IL4 - 3 | Untreated | 63 | Direct | 1 | 2 | 6.06E+02 |  |  |
| Medium oranges | IL4 - 3 | Untreated | 63 | Direct | 1 | 49 | 1.48E+04 |  |  |
| Medium white | IL4 - 3 | Untreated | 63 | Heat | 1 | 4 | 1.21E+03 | 304 | 9.21E+04 |
| Small white | IL4 - 3 | Untreated | 63 | Heat | 1 | 88 | 2.67E+04 |  |  |
| Medium beige | IL4 - 3 | Untreated | 63 | Heat | 1 | 12 | 3.64E+03 |  |  |
| Medium oranges | IL4 - 3 | Untreated | 63 | Heat | 1 | 140 | 4.24E+04 |  |  |
| Small translucent | IL4 - 3 | Untreated | 63 | Heat | 1 | 60 | 1.82E+04 |  |  |
| Small white | IL5 - 1 | Untreated | 63 | Direct | 2 | 47 | 1.42E+05 | 49 | 1.48E+05 |
| Medium oranges | IL5 - 1 | Untreated | 63 | Direct | 2 | 2 | 6.06E+03 |  |  |
| Medium white | IL5 - 1 | Untreated | 63 | Heat | 0 | 1 | 3.03E+01 | 132 | 4.00E+03 |
| Small white | IL5 - 1 | Untreated | 63 | Heat | 0 | 118 | 3.58E+03 |  |  |
| Medium melons | IL5 - 1 | Untreated | 63 | Heat | 0 | 1 | 3.03E+01 |  |  |
| Medium oranges | IL5 - 1 | Untreated | 63 | Heat | 0 | 12 | 3.64E+02 |  |  |
| Medium white | IL5 - 2 | Untreated | 63 | Direct | 1 | 3 | 9.09E+02 | 14 | 4.24E+03 |
| Medium oranges | IL5 - 2 | Untreated | 63 | Direct | 1 | 10 | 3.03E+03 |  |  |
| Pink | IL5 - 2 | Untreated | 63 | Direct | 1 | 1 | 3.03E+02 |  |  |
| Medium oranges | IL5 - 2 | Untreated | 63 | Heat | 1 | 10 | 3.03E+03 | 10 | 3.03E+03 |
| Medium white | IL5 - 3 | Untreated | 63 | Direct | 1 | 4 | 1.21E+03 | 59 | 1.79E+04 |
| Small white | IL5 - 3 | Untreated | 63 | Direct | 1 | 28 | 8.48E+03 |  |  |
| Small melons | IL5 - 3 | Untreated | 63 | Direct | 1 | 1 | 3.03E+02 |  |  |
| Medium oranges | IL5 - 3 | Untreated | 63 | Direct | 1 | 26 | 7.88E+03 |  |  |
| Medium white | IL5 - 3 | Untreated | 63 | Heat | 0 | 5 | 1.52E+02 | 108 | 3.27E+03 |
| Small beige | IL5 - 3 | Untreated | 63 | Heat | 0 | 12 | 3.64E+02 |  |  |
| Medium oranges | IL5 - 3 | Untreated | 63 | Heat | 0 | 88 | 2.67E+03 |  |  |
| Pink | IL5 - 3 | Untreated | 63 | Heat | 0 | 3 | 9.09E+01 |  |  |
| Small white | IL8 - 1 | Untreated | 63 | Direct | 0 | 53 | 1.61E+03 | 63 | 1.91E+03 |
| Medium beige | IL8 - 1 | Untreated | 63 | Direct | 0 | 4 | 1.21E+02 |  |  |
| Medium oranges | IL8 - 1 | Untreated | 63 | Direct | 0 | 5 | 1.52E+02 |  |  |
| Pink | IL8 - 1 | Untreated | 63 | Direct | 0 | 1 | 3.03E+01 |  |  |
| Medium white | IL8 - 1 | Untreated | 63 | Heat | 0 | 1 | 3.03E+01 | 12 | 3.64E+02 |
| Small beige | IL8 - 1 | Untreated | 63 | Heat | 0 | 2 | 6.06E+01 |  |  |
| Medium oranges | IL8 - 1 | Untreated | 63 | Heat | 0 | 5 | 1.52E+02 |  |  |
| Translucent yellow | IL8 - 1 | Untreated | 63 | Heat | 0 | 4 | 1.21E+02 |  |  |
| Small beige | IL8 - 2 | Untreated | 63 | Direct | 1 | 63 | 1.91E+04 | 105 | 3.18E+04 |
| Medium oranges | IL8 - 2 | Untreated | 63 | Direct | 1 | 42 | 1.27E+04 |  |  |
| Medium white | IL8 - 2 | Untreated | 63 | Heat | 0 | 8 | 2.42E+02 | 44 | 1.33E+03 |
| Small melons | IL8 - 2 | Untreated | 63 | Heat | 0 | 13 | 3.94E+02 |  |  |
| Medium oranges | IL8 - 2 | Untreated | 63 | Heat | 0 | 23 | 6.97E+02 |  |  |
| Small white | IL8 - 3 | Untreated | 63 | Direct | 0 | 155 | 4.70E+03 | 267 | 8.09E+03 |
| Medium beige | IL8 - 3 | Untreated | 63 | Direct | 0 | 4 | 1.21E+02 |  |  |
| Small melons | IL8 - 3 | Untreated | 63 | Direct | 0 | 1 | 3.03E+01 |  |  |
| Medium oranges | IL8 - 3 | Untreated | 63 | Direct | 0 | 107 | 3.24E+03 |  |  |
| Small melons | IL8 - 3 | Untreated | 63 | Heat | 0 | 2 | 6.06E+01 | 4 | 1.21E+02 |
| Medium oranges | IL8 - 3 | Untreated | 63 | Heat | 0 | 2 | 6.06E+01 |  |  |
| Medium white | IL4 - 1 | Heat | 63 | Direct | 3 | 9 | 2.73E+05 | 35 | 1.06E+06 |
| Medium oranges | IL4 - 1 | Heat | 63 | Direct | 3 | 26 | 7.88E+05 |  |  |
| Small white | IL4 - 1 | Heat | 63 | Heat | 2 | 24 | 7.27E+04 | 24 | 7.27E+04 |
| Medium yellow | IL4 - 2 | Heat | 63 | Direct | 2 | 52 | 1.58E+05 | 124 | 3.76E+05 |
| Medium white | IL4 - 2 | Heat | 63 | Direct | 2 | 12 | 3.64E+04 |  |  |
| Medium melons | IL4 - 2 | Heat | 63 | Direct | 2 | 36 | 1.09E+05 |  |  |
| Medium oranges | IL4 - 2 | Heat | 63 | Direct | 2 | 24 | 7.27E+04 |  |  |
| Medium yellow | IL4 - 2 | Heat | 63 | Heat | 2 | 24 | 7.27E+04 | 105 | 3.18E+05 |
| Medium white | IL4 - 2 | Heat | 63 | Heat | 2 | 1 | 3.03E+03 |  |  |
| Medium melons | IL4 - 2 | Heat | 63 | Heat | 2 | 52 | 1.58E+05 |  |  |
| Medium oranges | IL4 - 2 | Heat | 63 | Heat | 2 | 24 | 7.27E+04 |  |  |
| Pink | IL4 - 2 | Heat | 63 | Heat | 2 | 4 | 1.21E+04 |  |  |
| Medium yellow | IL4 -3 | Heat | 63 | Direct | 2 | 104 | 3.15E+05 | 164 | 4.97E+05 |
| Medium white | IL4 -3 | Heat | 63 | Direct | 2 | 36 | 1.09E+05 |  |  |
| Medium oranges | IL4 -3 | Heat | 63 | Direct | 2 | 16 | 4.85E+04 |  |  |
| Pink | IL4 -3 | Heat | 63 | Direct | 2 | 8 | 2.42E+04 |  |  |
| Medium yellow | IL4 -3 | Heat | 63 | Heat | 2 | 48 | 1.45E+05 | 164 | 4.97E+05 |
| Medium white | IL4 -3 | Heat | 63 | Heat | 2 | 44 | 1.33E+05 |  |  |
| Medium melons | IL4 -3 | Heat | 63 | Heat | 2 | 40 | 1.21E+05 |  |  |
| Medium oranges | IL4 -3 | Heat | 63 | Heat | 2 | 24 | 7.27E+04 |  |  |
| Pink | IL4 -3 | Heat | 63 | Heat | 2 | 8 | 2.42E+04 |  |  |
| Medium yellow | IL5 - 1 | Heat | 63 | Direct | 3 | 2 | 6.06E+04 | 23 | 6.97E+05 |
| Medium white | IL5 - 1 | Heat | 63 | Direct | 3 | 4 | 1.21E+05 |  |  |
| Medium beige | IL5 - 1 | Heat | 63 | Direct | 3 | 6 | 1.82E+05 |  |  |
| Medium oranges | IL5 - 1 | Heat | 63 | Direct | 3 | 4 | 1.21E+05 |  |  |
| Black | IL5 - 1 | Heat | 63 | Direct | 3 | 5 | 1.52E+05 |  |  |
| Medium translucent | IL5 - 1 | Heat | 63 | Direct | 3 | 2 | 6.06E+04 |  |  |
| Medium yellow | IL5 - 1 | Heat | 63 | Heat | 2 | 44 | 1.33E+05 | 224 | 6.79E+05 |
| Medium white | IL5 - 1 | Heat | 63 | Heat | 2 | 44 | 1.33E+05 |  |  |
| Medium melons | IL5 - 1 | Heat | 63 | Heat | 2 | 64 | 1.94E+05 |  |  |
| Medium oranges | IL5 - 1 | Heat | 63 | Heat | 2 | 16 | 4.85E+04 |  |  |
| Pink | IL5 - 1 | Heat | 63 | Heat | 2 | 44 | 1.33E+05 |  |  |
| Medium translucent | IL5 - 1 | Heat | 63 | Heat | 2 | 12 | 3.64E+04 |  |  |
| Medium yellow | IL5 - 2 | Heat | 63 | Direct | 1 | 36 | 1.09E+04 | 256 | 7.76E+04 |
| Medium melons | IL5 - 2 | Heat | 63 | Direct | 1 | 20 | 6.06E+03 |  |  |
| Medium oranges | IL5 - 2 | Heat | 63 | Direct | 1 | 24 | 7.27E+03 |  |  |
| Black | IL5 - 2 | Heat | 63 | Direct | 1 | 120 | 3.64E+04 |  |  |
| Medium pink | IL5 - 2 | Heat | 63 | Direct | 1 | 56 | 1.70E+04 |  |  |
| Medium yellow | IL5 - 2 | Heat | 63 | Heat | 1 | 16 | 4.85E+03 | 100 | 3.03E+04 |
| Small melons | IL5 - 2 | Heat | 63 | Heat | 1 | 8 | 2.42E+03 |  |  |
| Medium oranges | IL5 - 2 | Heat | 63 | Heat | 1 | 12 | 3.64E+03 |  |  |
| Pink | IL5 - 2 | Heat | 63 | Heat | 1 | 36 | 1.09E+04 |  |  |
| Translucent yellow | IL5 - 2 | Heat | 63 | Heat | 1 | 28 | 8.48E+03 |  |  |
| Yellow cottony | IL5 - 3 | Heat | 63 | Direct | 2 | 16 | 4.85E+04 | 84 | 2.55E+05 |
| Medium white | IL5 - 3 | Heat | 63 | Direct | 2 | 3 | 9.09E+03 |  |  |
| Small melons | IL5 - 3 | Heat | 63 | Direct | 2 | 5 | 1.52E+04 |  |  |
| Pink | IL5 - 3 | Heat | 63 | Direct | 2 | 60 | 1.82E+05 |  |  |
| Medium yellow | IL5 - 3 | Heat | 63 | Heat | 2 | 4 | 1.21E+04 | 16 | 4.85E+04 |
| Medium white | IL5 - 3 | Heat | 63 | Heat | 2 | 2 | 6.06E+03 |  |  |
| Small melons | IL5 - 3 | Heat | 63 | Heat | 2 | 2 | 6.06E+03 |  |  |
| Pink | IL5 - 3 | Heat | 63 | Heat | 2 | 8 | 2.42E+04 |  |  |
| Medium white | IL8 - 1 | Heat | 63 | Direct | 2 | 8 | 2.42E+04 | 276 | 8.36E+05 |
| Beige | IL8 - 1 | Heat | 63 | Direct | 2 | 8 | 2.42E+04 |  |  |
| Medium oranges | IL8 - 1 | Heat | 63 | Direct | 2 | 260 | 7.88E+05 |  |  |
| Yellow cottony | IL8 - 1 | Heat | 63 | Heat | 2 | 2 | 6.06E+03 | 148 | 4.48E+05 |
| Small white | IL8 - 1 | Heat | 63 | Heat | 2 | 8 | 2.42E+04 |  |  |
| Medium oranges | IL8 - 1 | Heat | 63 | Heat | 2 | 136 | 4.12E+05 |  |  |
| Small translucent | IL8 - 1 | Heat | 63 | Heat | 2 | 2 | 6.06E+03 |  |  |
| Beige | IL8 - 2 | Heat | 63 | Direct | 3 | 1 | 3.03E+04 | 39 | 1.18E+06 |
| Medium beige | IL8 - 2 | Heat | 63 | Direct | 3 | 20 | 6.06E+05 |  |  |
| naranja mediana | IL8 - 2 | Heat | 63 | Direct | 3 | 18 | 5.45E+05 |  |  |
| Beige | IL8 - 2 | Heat | 63 | Heat | 2 | 16 | 4.85E+04 | 300 | 9.09E+05 |
| Medium beige | IL8 - 2 | Heat | 63 | Heat | 2 | 8 | 2.42E+04 |  |  |
| Medium oranges | IL8 - 2 | Heat | 63 | Heat | 2 | 276 | 8.36E+05 |  |  |
| Beige | IL8 - 3 | Heat | 63 | Direct | 3 | 6 | 1.82E+05 | 78 | 2.36E+06 |
| Small beige | IL8 - 3 | Heat | 63 | Direct | 3 | 40 | 1.21E+06 |  |  |
| Medium oranges | IL8 - 3 | Heat | 63 | Direct | 3 | 30 | 9.09E+05 |  |  |
| Black | IL8 - 3 | Heat | 63 | Direct | 3 | 2 | 6.06E+04 |  |  |
| Beige | IL8 - 3 | Heat | 63 | Heat | 2 | 76 | 2.30E+05 | 208 | 6.30E+05 |
| Medium oranges | IL8 - 3 | Heat | 63 | Heat | 2 | 132 | 4.00E+05 |  |  |
| Small white | IL4 - 1 | Untreated | 81 | Direct | 1 | 32 | 9.70E+03 | 37 | 1.12E+04 |
| Medium oranges | IL4 - 1 | Untreated | 81 | Direct | 1 | 1 | 3.03E+02 |  |  |
| Medium white | IL4 - 1 | Untreated | 81 | Direct | 1 | 4 | 1.21E+03 |  |  |
| Medium oranges | IL4 - 1 | Untreated | 81 | Heat | 0 | 2 | 6.06E+01 | 2 | 6.06E+01 |
| Small yellow | IL4 - 2 | Untreated | 81 | Direct | 2 | 13 | 3.94E+04 | 86 | 2.61E+05 |
| Medium white | IL4 - 2 | Untreated | 81 | Direct | 2 | 54 | 1.64E+05 |  |  |
| Small white | IL4 - 2 | Untreated | 81 | Direct | 2 | 18 | 5.45E+04 |  |  |
| Medium melons | IL4 - 2 | Untreated | 81 | Direct | 2 | 1 | 3.03E+03 |  |  |
| Medium white | IL4 - 2 | Untreated | 81 | Heat | 0 | 1 | 3.03E+01 | 40 | 1.21E+03 |
| Small white | IL4 - 2 | Untreated | 81 | Heat | 0 | 1 | 3.03E+01 |  |  |
| Black | IL4 - 2 | Untreated | 81 | Heat | 0 | 34 | 1.03E+03 |  |  |
| Medium pink | IL4 - 2 | Untreated | 81 | Heat | 0 | 4 | 1.21E+02 |  |  |
| Medium yellow | IL4 - 3 | Untreated | 81 | Direct | 0 | 6 | 1.82E+02 | 258 | 7.82E+03 |
| Small yellow | IL4 - 3 | Untreated | 81 | Direct | 0 | 236 | 7.15E+03 |  |  |
| Medium melons | IL4 - 3 | Untreated | 81 | Direct | 0 | 3 | 9.09E+01 |  |  |
| Medium oranges | IL4 - 3 | Untreated | 81 | Direct | 0 | 13 | 3.94E+02 |  |  |
| Medium beige | IL4 - 3 | Untreated | 81 | Heat | 0 | 2 | 6.06E+01 | 19 | 5.76E+02 |
| Medium melons | IL4 - 3 | Untreated | 81 | Heat | 0 | 2 | 6.06E+01 |  |  |
| Medium oranges | IL4 - 3 | Untreated | 81 | Heat | 0 | 13 | 3.94E+02 |  |  |
| Medium pink | IL4 - 3 | Untreated | 81 | Heat | 0 | 2 | 6.06E+01 |  |  |
| Small yellow | IL5 - 1 | Untreated | 81 | Direct | 2 | 172 | 5.21E+05 | 180 | 5.45E+05 |
| Small translucent | IL5 - 1 | Untreated | 81 | Direct | 2 | 8 | 2.42E+04 |  |  |
| Medium yellow | IL5 - 1 | Untreated | 81 | Heat | 0 | 2 | 6.06E+01 | 128 | 3.88E+03 |
| Medium white | IL5 - 1 | Untreated | 81 | Heat | 0 | 1 | 3.03E+01 |  |  |
| Small white | IL5 - 1 | Untreated | 81 | Heat | 0 | 121 | 3.67E+03 |  |  |
| Medium melons | IL5 - 1 | Untreated | 81 | Heat | 0 | 2 | 6.06E+01 |  |  |
| Pink | IL5 - 1 | Untreated | 81 | Heat | 0 | 2 | 6.06E+01 |  |  |
| Small yellow | IL5 - 2 | Untreated | 81 | Direct | 1 | 136 | 4.12E+04 | 140 | 4.24E+04 |
| Beige | IL5 - 2 | Untreated | 81 | Direct | 1 | 1 | 3.03E+02 |  |  |
| Medium oranges | IL5 - 2 | Untreated | 81 | Direct | 1 | 3 | 9.09E+02 |  |  |
| Yellow cottony | IL5 - 2 | Untreated | 81 | Heat | 1 | 16 | 4.85E+03 | 40 | 1.21E+04 |
| Beige | IL5 - 2 | Untreated | 81 | Heat | 1 | 1 | 3.03E+02 |  |  |
| Medium oranges | IL5 - 2 | Untreated | 81 | Heat | 1 | 23 | 6.97E+03 |  |  |
| Medium yellow | IL5 - 3 | Untreated | 81 | Direct | 1 | 3 | 9.09E+02 | 109 | 3.30E+04 |
| Medium white | IL5 - 3 | Untreated | 81 | Direct | 1 | 1 | 3.03E+02 |  |  |
| Small white | IL5 - 3 | Untreated | 81 | Direct | 1 | 40 | 1.21E+04 |  |  |
| Medium melons | IL5 - 3 | Untreated | 81 | Direct | 1 | 1 | 3.03E+02 |  |  |
| Small melons | IL5 - 3 | Untreated | 81 | Direct | 1 | 56 | 1.70E+04 |  |  |
| Medium oranges | IL5 - 3 | Untreated | 81 | Direct | 1 | 8 | 2.42E+03 |  |  |
| Medium yellow | IL5 - 3 | Untreated | 81 | Heat | 0 | 11 | 3.33E+02 | 109 | 3.30E+03 |
| Medium white | IL5 - 3 | Untreated | 81 | Heat | 0 | 1 | 3.03E+01 |  |  |
| Beige | IL5 - 3 | Untreated | 81 | Heat | 0 | 1 | 3.03E+01 |  |  |
| Medium beige | IL5 - 3 | Untreated | 81 | Heat | 0 | 2 | 6.06E+01 |  |  |
| Medium melons | IL5 - 3 | Untreated | 81 | Heat | 0 | 3 | 9.09E+01 |  |  |
| Small melons | IL5 - 3 | Untreated | 81 | Heat | 0 | 5 | 1.52E+02 |  |  |
| Medium oranges | IL5 - 3 | Untreated | 81 | Heat | 0 | 86 | 2.61E+03 |  |  |
| Medium white | IL8 - 1 | Untreated | 81 | Direct | 1 | 7 | 2.12E+03 | 291 | 8.82E+04 |
| Small white | IL8 - 1 | Untreated | 81 | Direct | 1 | 53 | 1.61E+04 |  |  |
| Beige | IL8 - 1 | Untreated | 81 | Direct | 1 | 222 | 6.73E+04 |  |  |
| Medium oranges | IL8 - 1 | Untreated | 81 | Direct | 1 | 9 | 2.73E+03 |  |  |
| Small yellow | IL8 - 1 | Untreated | 81 | Heat | 0 | 2 | 6.06E+01 | 8 | 2.42E+02 |
| Medium melons | IL8 - 1 | Untreated | 81 | Heat | 0 | 1 | 3.03E+01 |  |  |
| Small melons | IL8 - 1 | Untreated | 81 | Heat | 0 | 3 | 9.09E+01 |  |  |
| Medium pink | IL8 - 1 | Untreated | 81 | Heat | 0 | 2 | 6.06E+01 |  |  |
| Small white | IL8 - 2 | Untreated | 81 | Direct | 0 | 1 | 3.03E+01 | 10 | 3.03E+02 |
| Medium beige | IL8 - 2 | Untreated | 81 | Direct | 0 | 1 | 3.03E+01 |  |  |
| Small beige | IL8 - 2 | Untreated | 81 | Direct | 0 | 2 | 6.06E+01 |  |  |
| Medium oranges | IL8 - 2 | Untreated | 81 | Direct | 0 | 5 | 1.52E+02 |  |  |
| Medium pink | IL8 - 2 | Untreated | 81 | Direct | 0 | 1 | 3.03E+01 |  |  |
| Medium yellow | IL8 - 2 | Untreated | 81 | Heat | 0 | 1 | 3.03E+01 | 5 | 1.52E+02 |
| Beige | IL8 - 2 | Untreated | 81 | Heat | 0 | 2 | 6.06E+01 |  |  |
| Medium beige | IL8 - 2 | Untreated | 81 | Heat | 0 | 2 | 6.06E+01 |  |  |
| Small yellow | IL8 - 3 | Untreated | 81 | Direct | 0 | 60 | 1.82E+03 | 823 | 2.49E+04 |
| Small white | IL8 - 3 | Untreated | 81 | Direct | 0 | 753 | 2.28E+04 |  |  |
| Small beige | IL8 - 3 | Untreated | 81 | Direct | 0 | 2 | 6.06E+01 |  |  |
| Medium oranges | IL8 - 3 | Untreated | 81 | Direct | 0 | 6 | 1.82E+02 |  |  |
| Medium pink | IL8 - 3 | Untreated | 81 | Direct | 0 | 2 | 6.06E+01 |  |  |
| Medium yellow | IL8 - 3 | Untreated | 81 | Heat | 0 | 1 | 3.03E+01 | 5 | 1.52E+02 |
| Medium oranges | IL8 - 3 | Untreated | 81 | Heat | 0 | 1 | 3.03E+01 |  |  |
| Medium pink | IL8 - 3 | Untreated | 81 | Heat | 0 | 3 | 9.09E+01 |  |  |
| Medium white | IL4 - 1 | Heat | 81 | Direct | 2 | 2 | 6.06E+03 | 50 | 1.52E+05 |
| Beige | IL4 - 1 | Heat | 81 | Direct | 2 | 2 | 6.06E+03 |  |  |
| Small melons | IL4 - 1 | Heat | 81 | Direct | 2 | 1 | 3.03E+03 |  |  |
| Medium oranges | IL4 - 1 | Heat | 81 | Direct | 2 | 36 | 1.09E+05 |  |  |
| Pink | IL4 - 1 | Heat | 81 | Direct | 2 | 9 | 2.73E+04 |  |  |
| Beige | IL4 - 1 | Heat | 81 | Heat | 0 | 4 | 1.21E+02 | 29 | 8.79E+02 |
| Medium melons | IL4 - 1 | Heat | 81 | Heat | 0 | 4 | 1.21E+02 |  |  |
| Small melons | IL4 - 1 | Heat | 81 | Heat | 0 | 2 | 6.06E+01 |  |  |
| Medium oranges | IL4 - 1 | Heat | 81 | Heat | 0 | 8 | 2.42E+02 |  |  |
| Medium pink | IL4 - 1 | Heat | 81 | Heat | 0 | 10 | 3.03E+02 |  |  |
| Medium white | IL4 - 1 | Heat | 81 | Heat | 0 | 1 | 3.03E+01 |  |  |
| Medium white | IL4 - 2 | Heat | 81 | Direct | 3 | 1 | 3.03E+04 | 41 | 1.24E+06 |
| Small beige | IL4 - 2 | Heat | 81 | Direct | 3 | 14 | 4.24E+05 |  |  |
| Medium oranges | IL4 - 2 | Heat | 81 | Direct | 3 | 26 | 7.88E+05 |  |  |
| Small beige | IL4 - 2 | Heat | 81 | Heat | 2 | 88 | 2.67E+05 | 173 | 5.24E+05 |
| Medium oranges | IL4 - 2 | Heat | 81 | Heat | 2 | 84 | 2.55E+05 |  |  |
| Pink | IL4 - 2 | Heat | 81 | Heat | 2 | 1 | 3.03E+03 |  |  |
| Medium yellow | IL4 -3 | Heat | 81 | Direct | 3 | 2 | 6.06E+04 | 40 | 1.21E+06 |
| Small yellow | IL4 -3 | Heat | 81 | Direct | 3 | 2 | 6.06E+04 |  |  |
| Small white | IL4 -3 | Heat | 81 | Direct | 3 | 1 | 3.03E+04 |  |  |
| Beige | IL4 -3 | Heat | 81 | Direct | 3 | 4 | 1.21E+05 |  |  |
| Small melons | IL4 -3 | Heat | 81 | Direct | 3 | 1 | 3.03E+04 |  |  |
| Medium oranges | IL4 -3 | Heat | 81 | Direct | 3 | 26 | 7.88E+05 |  |  |
| Pink | IL4 -3 | Heat | 81 | Direct | 3 | 1 | 3.03E+04 |  |  |
| Medium translucent | IL4 -3 | Heat | 81 | Direct | 3 | 1 | 3.03E+04 |  |  |
| Small translucent | IL4 -3 | Heat | 81 | Direct | 3 | 2 | 6.06E+04 |  |  |
| Small yellow | IL4 -3 | Heat | 81 | Heat | 2 | 16 | 4.85E+04 | 200 | 6.06E+05 |
| Small white | IL4 -3 | Heat | 81 | Heat | 2 | 8 | 2.42E+04 |  |  |
| Small melons | IL4 -3 | Heat | 81 | Heat | 2 | 24 | 7.27E+04 |  |  |
| Medium oranges | IL4 -3 | Heat | 81 | Heat | 2 | 96 | 2.91E+05 |  |  |
| Small translucent | IL4 -3 | Heat | 81 | Heat | 2 | 16 | 4.85E+04 |  |  |
| Red | IL4 -3 | Heat | 81 | Heat | 2 | 40 | 1.21E+05 |  |  |
| Medium yellow | IL5 - 1 | Heat | 81 | Direct | 2 | 1 | 3.03E+03 | 50 | 1.52E+05 |
| Medium white | IL5 - 1 | Heat | 81 | Direct | 2 | 1 | 3.03E+03 |  |  |
| Small white | IL5 - 1 | Heat | 81 | Direct | 2 | 3 | 9.09E+03 |  |  |
| Beige | IL5 - 1 | Heat | 81 | Direct | 2 | 20 | 6.06E+04 |  |  |
| Medium oranges | IL5 - 1 | Heat | 81 | Direct | 2 | 17 | 5.15E+04 |  |  |
| Pink | IL5 - 1 | Heat | 81 | Direct | 2 | 8 | 2.42E+04 |  |  |
| Medium yellow | IL5 - 1 | Heat | 81 | Heat | 2 | 6 | 1.82E+04 | 46 | 1.39E+05 |
| Medium white | IL5 - 1 | Heat | 81 | Heat | 2 | 16 | 4.85E+04 |  |  |
| Beige | IL5 - 1 | Heat | 81 | Heat | 2 | 3 | 9.09E+03 |  |  |
| Medium melons | IL5 - 1 | Heat | 81 | Heat | 2 | 7 | 2.12E+04 |  |  |
| Pink | IL5 - 1 | Heat | 81 | Heat | 2 | 13 | 3.94E+04 |  |  |
| Medium translucent | IL5 - 1 | Heat | 81 | Heat | 2 | 1 | 3.03E+03 |  |  |
| Small white | IL5 - 2 | Heat | 81 | Direct | 2 | 11 | 3.33E+04 | 41 | 1.24E+05 |
| Medium melons | IL5 - 2 | Heat | 81 | Direct | 2 | 5 | 1.52E+04 |  |  |
| Medium oranges | IL5 - 2 | Heat | 81 | Direct | 2 | 11 | 3.33E+04 |  |  |
| Pink | IL5 - 2 | Heat | 81 | Direct | 2 | 14 | 4.24E+04 |  |  |
| Medium yellow | IL5 - 2 | Heat | 81 | Heat | 1 | 16 | 4.85E+03 | 134 | 4.06E+04 |
| Medium white | IL5 - 2 | Heat | 81 | Heat | 1 | 44 | 1.33E+04 |  |  |
| Beige | IL5 - 2 | Heat | 81 | Heat | 1 | 26 | 7.88E+03 |  |  |
| Medium melons | IL5 - 2 | Heat | 81 | Heat | 1 | 8 | 2.42E+03 |  |  |
| Pink | IL5 - 2 | Heat | 81 | Heat | 1 | 40 | 1.21E+04 |  |  |
| Medium yellow | IL5 - 3 | Heat | 81 | Direct | 2 | 1 | 3.03E+03 | 66 | 2.00E+05 |
| Small yellow | IL5 - 3 | Heat | 81 | Direct | 2 | 11 | 3.33E+04 |  |  |
| Beige | IL5 - 3 | Heat | 81 | Direct | 2 | 3 | 9.09E+03 |  |  |
| Medium melons | IL5 - 3 | Heat | 81 | Direct | 2 | 7 | 2.12E+04 |  |  |
| Medium oranges | IL5 - 3 | Heat | 81 | Direct | 2 | 16 | 4.85E+04 |  |  |
| Pink | IL5 - 3 | Heat | 81 | Direct | 2 | 28 | 8.48E+04 |  |  |
| Small yellow | IL5 - 3 | Heat | 81 | Heat | 1 | 20 | 6.06E+03 | 196 | 5.94E+04 |
| Beige | IL5 - 3 | Heat | 81 | Heat | 1 | 16 | 4.85E+03 |  |  |
| Small beige | IL5 - 3 | Heat | 81 | Heat | 1 | 68 | 2.06E+04 |  |  |
| Medium oranges | IL5 - 3 | Heat | 81 | Heat | 1 | 24 | 7.27E+03 |  |  |
| Pink | IL5 - 3 | Heat | 81 | Heat | 1 | 68 | 2.06E+04 |  |  |
| Small yellow | IL8 - 1 | Heat | 81 | Direct | 1 | 2 | 6.06E+02 | 122 | 3.70E+04 |
| Small white | IL8 - 1 | Heat | 81 | Direct | 1 | 10 | 3.03E+03 |  |  |
| Beige | IL8 - 1 | Heat | 81 | Direct | 1 | 6 | 1.82E+03 |  |  |
| Medium melons | IL8 - 1 | Heat | 81 | Direct | 1 | 2 | 6.06E+02 |  |  |
| Medium oranges | IL8 - 1 | Heat | 81 | Direct | 1 | 100 | 3.03E+04 |  |  |
| Pink | IL8 - 1 | Heat | 81 | Direct | 1 | 2 | 6.06E+02 |  |  |
| Small yellow | IL8 - 1 | Heat | 81 | Heat | 1 | 2 | 6.06E+02 | 43 | 1.30E+04 |
| Medium white | IL8 - 1 | Heat | 81 | Heat | 1 | 2 | 6.06E+02 |  |  |
| Small white | IL8 - 1 | Heat | 81 | Heat | 1 | 4 | 1.21E+03 |  |  |
| Medium melons | IL8 - 1 | Heat | 81 | Heat | 1 | 4 | 1.21E+03 |  |  |
| Small melons | IL8 - 1 | Heat | 81 | Heat | 1 | 2 | 6.06E+02 |  |  |
| Medium oranges | IL8 - 1 | Heat | 81 | Heat | 1 | 24 | 7.27E+03 |  |  |
| Pink | IL8 - 1 | Heat | 81 | Heat | 1 | 3 | 9.09E+02 |  |  |
| Small translucent | IL8 - 1 | Heat | 81 | Heat | 1 | 2 | 6.06E+02 |  |  |
| Beige | IL8 - 2 | Heat | 81 | Direct | 3 | 4 | 1.21E+05 | 48 | 1.45E+06 |
| Small beige | IL8 - 2 | Heat | 81 | Direct | 3 | 3 | 9.09E+04 |  |  |
| Medium oranges | IL8 - 2 | Heat | 81 | Direct | 3 | 39 | 1.18E+06 |  |  |
| Pink | IL8 - 2 | Heat | 81 | Direct | 3 | 2 | 6.06E+04 |  |  |
| Small white | IL8 - 2 | Heat | 81 | Heat | 2 | 1 | 3.03E+03 | 67 | 2.03E+05 |
| Beige | IL8 - 2 | Heat | 81 | Heat | 2 | 7 | 2.12E+04 |  |  |
| Small beige | IL8 - 2 | Heat | 81 | Heat | 2 | 7 | 2.12E+04 |  |  |
| Medium melons | IL8 - 2 | Heat | 81 | Heat | 2 | 1 | 3.03E+03 |  |  |
| Small melons | IL8 - 2 | Heat | 81 | Heat | 2 | 2 | 6.06E+03 |  |  |
| Medium oranges | IL8 - 2 | Heat | 81 | Heat | 2 | 48 | 1.45E+05 |  |  |
| Pink | IL8 - 2 | Heat | 81 | Heat | 2 | 1 | 3.03E+03 |  |  |
| Small white | IL8 - 3 | Heat | 81 | Direct | 2 | 13 | 3.94E+04 | 65 | 1.97E+05 |
| Beige | IL8 - 3 | Heat | 81 | Direct | 2 | 15 | 4.55E+04 |  |  |
| Medium beige | IL8 - 3 | Heat | 81 | Direct | 2 | 5 | 1.52E+04 |  |  |
| Medium melons | IL8 - 3 | Heat | 81 | Direct | 2 | 2 | 6.06E+03 |  |  |
| Medium oranges | IL8 - 3 | Heat | 81 | Direct | 2 | 27 | 8.18E+04 |  |  |
| Pink | IL8 - 3 | Heat | 81 | Direct | 2 | 2 | 6.06E+03 |  |  |
| Medium translucent | IL8 - 3 | Heat | 81 | Direct | 2 | 1 | 3.03E+03 |  |  |
| Beige | IL8 - 3 | Heat | 81 | Heat | 2 | 4 | 1.21E+04 | 56 | 1.70E+05 |
| Medium melons | IL8 - 3 | Heat | 81 | Heat | 2 | 12 | 3.64E+04 |  |  |
| Medium oranges | IL8 - 3 | Heat | 81 | Heat | 2 | 30 | 9.09E+04 |  |  |
| Pink | IL8 - 3 | Heat | 81 | Heat | 2 | 4 | 1.21E+04 |  |  |
| Translucent yellow | IL8 - 3 | Heat | 81 | Heat | 2 | 6 | 1.82E+04 |  |  |
| Medium yellow | IL4 - 1 | Untreated | 98 | Direct | 0 | 1 | 3.03E+01 | 14 | 4.24E+02 |
| Small yellow | IL4 - 1 | Untreated | 98 | Direct | 0 | 12 | 3.64E+02 |  |  |
| Pink | IL4 - 1 | Untreated | 98 | Direct | 0 | 1 | 3.03E+01 |  |  |
| Small white | IL4 - 2 | Untreated | 98 | Direct | 1 | 138 | 4.18E+04 | 138 | 4.18E+04 |
| Medium yellow | IL4 - 2 | Untreated | 98 | Heat | 0 | 14 | 4.24E+02 | 20 | 6.06E+02 |
| Small white | IL4 - 2 | Untreated | 98 | Heat | 0 | 1 | 3.03E+01 |  |  |
| Medium melons | IL4 - 2 | Untreated | 98 | Heat | 0 | 1 | 3.03E+01 |  |  |
| Medium oranges | IL4 - 2 | Untreated | 98 | Heat | 0 | 4 | 1.21E+02 |  |  |
| Small yellow | IL4 - 3 | Untreated | 98 | Direct | 0 | 7 | 2.12E+02 | 56 | 1.70E+03 |
| Small white | IL4 - 3 | Untreated | 98 | Direct | 0 | 44 | 1.33E+03 |  |  |
| Medium oranges | IL4 - 3 | Untreated | 98 | Direct | 0 | 5 | 1.52E+02 |  |  |
| Medium yellow | IL4 - 3 | Untreated | 98 | Heat | 0 | 4 | 1.21E+02 | 4 | 1.21E+02 |
| Small yellow | IL5 - 1 | Untreated | 98 | Direct | 3 | 2 | 6.06E+04 | 3 | 9.09E+04 |
| Medium white | IL5 - 1 | Untreated | 98 | Direct | 3 | 1 | 3.03E+04 |  |  |
| Small white | IL5 - 1 | Untreated | 98 | Heat | 0 | 15 | 4.55E+02 | 15 | 4.55E+02 |
| Medium yellow | IL5 - 2 | Untreated | 98 | Direct | 1 | 1 | 3.03E+02 | 3 | 9.09E+02 |
| Small yellow | IL5 - 2 | Untreated | 98 | Direct | 1 | 1 | 3.03E+02 |  |  |
| Medium oranges | IL5 - 2 | Untreated | 98 | Direct | 1 | 1 | 3.03E+02 |  |  |
| Medium oranges | IL5 - 2 | Untreated | 98 | Heat | 0 | 12 | 3.64E+02 | 12 | 3.64E+02 |
| Medium yellow | IL5 - 3 | Untreated | 98 | Direct | 0 | 10 | 3.03E+02 | 279 | 8.45E+03 |
| Small yellow | IL5 - 3 | Untreated | 98 | Direct | 0 | 27 | 8.18E+02 |  |  |
| Medium white | IL5 - 3 | Untreated | 98 | Direct | 0 | 1 | 3.03E+01 |  |  |
| Small white | IL5 - 3 | Untreated | 98 | Direct | 0 | 134 | 4.06E+03 |  |  |
| Small melons | IL5 - 3 | Untreated | 98 | Direct | 0 | 92 | 2.79E+03 |  |  |
| Black | IL5 - 3 | Untreated | 98 | Direct | 0 | 15 | 4.55E+02 |  |  |
| Medium melons | IL5 - 3 | Untreated | 98 | Heat | 0 | 4 | 1.21E+02 | 11 | 3.33E+02 |
| Medium oranges | IL5 - 3 | Untreated | 98 | Heat | 0 | 7 | 2.12E+02 |  |  |
| Small yellow | IL8 - 1 | Untreated | 98 | Direct | 0 | 50 | 1.52E+03 | 230 | 6.97E+03 |
| Small white | IL8 - 1 | Untreated | 98 | Direct | 0 | 146 | 4.42E+03 |  |  |
| Medium oranges | IL8 - 1 | Untreated | 98 | Direct | 0 | 32 | 9.70E+02 |  |  |
| Medium pink | IL8 - 1 | Untreated | 98 | Direct | 0 | 2 | 6.06E+01 |  |  |
| Small yellow | IL8 - 1 | Untreated | 98 | Heat | 0 | 1 | 3.03E+01 | 3 | 9.09E+01 |
| Small white | IL8 - 1 | Untreated | 98 | Heat | 0 | 1 | 3.03E+01 |  |  |
| Medium oranges | IL8 - 1 | Untreated | 98 | Heat | 0 | 1 | 3.03E+01 |  |  |
| Medium yellow | IL8 - 2 | Untreated | 98 | Direct | 0 | 1 | 3.03E+01 | 17 | 5.15E+02 |
| Small yellow | IL8 - 2 | Untreated | 98 | Direct | 0 | 4 | 1.21E+02 |  |  |
| Small white | IL8 - 2 | Untreated | 98 | Direct | 0 | 4 | 1.21E+02 |  |  |
| Medium melons | IL8 - 2 | Untreated | 98 | Direct | 0 | 1 | 3.03E+01 |  |  |
| Translucent yellow | IL8 - 2 | Untreated | 98 | Direct | 0 | 4 | 1.21E+02 |  |  |
| Beige | IL8 - 2 | Untreated | 98 | Direct | 0 | 3 | 9.09E+01 |  |  |
| Medium white | IL8 - 2 | Untreated | 98 | Heat | 0 | 4 | 1.21E+02 | 5 | 1.52E+02 |
| Translucent yellow | IL8 - 2 | Untreated | 98 | Heat | 0 | 1 | 3.03E+01 |  |  |
| Small white | IL8 - 3 | Untreated | 98 | Direct | 1 | 46 | 1.39E+04 | 46 | 1.39E+04 |
| Medium yellow | IL8 - 3 | Untreated | 98 | Heat | 0 | 1 | 3.03E+01 | 6 | 1.82E+02 |
| Medium white | IL8 - 3 | Untreated | 98 | Heat | 0 | 3 | 9.09E+01 |  |  |
| Medium melons | IL8 - 3 | Untreated | 98 | Heat | 0 | 1 | 3.03E+01 |  |  |
| Medium oranges | IL8 - 3 | Untreated | 98 | Heat | 0 | 1 | 3.03E+01 |  |  |
| Medium yellow | IL4 - 1 | Heat | 98 | Direct | 3 | 13 | 3.94E+05 | 55 | 1.67E+06 |
| Beige | IL4 - 1 | Heat | 98 | Direct | 3 | 15 | 4.55E+05 |  |  |
| Small melons | IL4 - 1 | Heat | 98 | Direct | 3 | 3 | 9.09E+04 |  |  |
| Medium oranges | IL4 - 1 | Heat | 98 | Direct | 3 | 22 | 6.67E+05 |  |  |
| Pink | IL4 - 1 | Heat | 98 | Direct | 3 | 2 | 6.06E+04 |  |  |
| Medium yellow | IL4 - 1 | Heat | 98 | Heat | 2 | 78 | 2.36E+05 | 154 | 4.67E+05 |
| Beige | IL4 - 1 | Heat | 98 | Heat | 2 | 24 | 7.27E+04 |  |  |
| Medium melons | IL4 - 1 | Heat | 98 | Heat | 2 | 14 | 4.24E+04 |  |  |
| Medium oranges | IL4 - 1 | Heat | 98 | Heat | 2 | 30 | 9.09E+04 |  |  |
| Pink | IL4 - 1 | Heat | 98 | Heat | 2 | 8 | 2.42E+04 |  |  |
| Medium yellow | IL4 - 2 | Heat | 98 | Direct | 3 | 18 | 5.45E+05 | 46 | 1.39E+06 |
| Beige | IL4 - 2 | Heat | 98 | Direct | 3 | 13 | 3.94E+05 |  |  |
| Medium translucent | IL4 - 2 | Heat | 98 | Direct | 3 | 2 | 6.06E+04 |  |  |
| Medium oranges | IL4 - 2 | Heat | 98 | Direct | 3 | 13 | 3.94E+05 |  |  |
| Medium yellow | IL4 - 2 | Heat | 98 | Heat | 2 | 72 | 2.18E+05 | 130 | 3.94E+05 |
| Medium white | IL4 - 2 | Heat | 98 | Heat | 2 | 1 | 3.03E+03 |  |  |
| Beige | IL4 - 2 | Heat | 98 | Heat | 2 | 28 | 8.48E+04 |  |  |
| Medium oranges | IL4 - 2 | Heat | 98 | Heat | 2 | 20 | 6.06E+04 |  |  |
| Pink | IL4 - 2 | Heat | 98 | Heat | 2 | 3 | 9.09E+03 |  |  |
| Small translucent | IL4 - 2 | Heat | 98 | Heat | 2 | 6 | 1.82E+04 |  |  |
| Small yellow | IL4 -3 | Heat | 98 | Direct | 3 | 81 | 2.45E+06 | 134 | 4.06E+06 |
| Beige | IL4 -3 | Heat | 98 | Direct | 3 | 22 | 6.67E+05 |  |  |
| Medium melons | IL4 -3 | Heat | 98 | Direct | 3 | 2 | 6.06E+04 |  |  |
| Small melons | IL4 -3 | Heat | 98 | Direct | 3 | 2 | 6.06E+04 |  |  |
| Medium oranges | IL4 -3 | Heat | 98 | Direct | 3 | 25 | 7.58E+05 |  |  |
| Pink | IL4 -3 | Heat | 98 | Direct | 3 | 2 | 6.06E+04 |  |  |
| Small yellow | IL4 -3 | Heat | 98 | Heat | 2 | 296 | 8.97E+05 | 339 | 1.03E+06 |
| Beige | IL4 -3 | Heat | 98 | Heat | 2 | 12 | 3.64E+04 |  |  |
| Medium oranges | IL4 -3 | Heat | 98 | Heat | 2 | 28 | 8.48E+04 |  |  |
| Pink | IL4 -3 | Heat | 98 | Heat | 2 | 3 | 9.09E+03 |  |  |
| Medium yellow | IL5 - 1 | Heat | 98 | Direct | 2 | 9 | 2.73E+04 | 86 | 2.61E+05 |
| Small yellow | IL5 - 1 | Heat | 98 | Direct | 2 | 2 | 6.06E+03 |  |  |
| Small white | IL5 - 1 | Heat | 98 | Direct | 2 | 17 | 5.15E+04 |  |  |
| Beige | IL5 - 1 | Heat | 98 | Direct | 2 | 40 | 1.21E+05 |  |  |
| Medium oranges | IL5 - 1 | Heat | 98 | Direct | 2 | 8 | 2.42E+04 |  |  |
| Pink | IL5 - 1 | Heat | 98 | Direct | 2 | 10 | 3.03E+04 |  |  |
| Small yellow | IL5 - 1 | Heat | 98 | Heat | 2 | 19 | 5.76E+04 | 83 | 2.52E+05 |
| Beige | IL5 - 1 | Heat | 98 | Heat | 2 | 55 | 1.67E+05 |  |  |
| Pink | IL5 - 1 | Heat | 98 | Heat | 2 | 6 | 1.82E+04 |  |  |
| Small translucent | IL5 - 1 | Heat | 98 | Heat | 2 | 3 | 9.09E+03 |  |  |
| Medium yellow | IL5 - 2 | Heat | 98 | Direct | 1 | 13 | 3.94E+03 | 72 | 2.18E+04 |
| Beige | IL5 - 2 | Heat | 98 | Direct | 1 | 12 | 3.64E+03 |  |  |
| Small melons | IL5 - 2 | Heat | 98 | Direct | 1 | 1 | 3.03E+02 |  |  |
| Medium oranges | IL5 - 2 | Heat | 98 | Direct | 1 | 10 | 3.03E+03 |  |  |
| Pink | IL5 - 2 | Heat | 98 | Direct | 1 | 15 | 4.55E+03 |  |  |
| Translucent yellow | IL5 - 2 | Heat | 98 | Direct | 1 | 17 | 5.15E+03 |  |  |
| Small translucent | IL5 - 2 | Heat | 98 | Direct | 1 | 4 | 1.21E+03 |  |  |
| Medium yellow | IL5 - 2 | Heat | 98 | Heat | 1 | 18 | 5.45E+03 | 83 | 2.52E+04 |
| Small white | IL5 - 2 | Heat | 98 | Heat | 1 | 6 | 1.82E+03 |  |  |
| Beige | IL5 - 2 | Heat | 98 | Heat | 1 | 11 | 3.33E+03 |  |  |
| Medium oranges | IL5 - 2 | Heat | 98 | Heat | 1 | 4 | 1.21E+03 |  |  |
| Pink | IL5 - 2 | Heat | 98 | Heat | 1 | 15 | 4.55E+03 |  |  |
| Translucent yellow | IL5 - 2 | Heat | 98 | Heat | 1 | 29 | 8.79E+03 |  |  |
| Medium yellow | IL5 - 3 | Heat | 98 | Direct | 2 | 16 | 4.85E+04 | 275 | 8.33E+05 |
| Small yellow | IL5 - 3 | Heat | 98 | Direct | 2 | 162 | 4.91E+05 |  |  |
| Small white | IL5 - 3 | Heat | 98 | Direct | 2 | 16 | 4.85E+04 |  |  |
| Beige | IL5 - 3 | Heat | 98 | Direct | 2 | 24 | 7.27E+04 |  |  |
| Pink | IL5 - 3 | Heat | 98 | Direct | 2 | 42 | 1.27E+05 |  |  |
| Translucent yellow | IL5 - 3 | Heat | 98 | Direct | 2 | 6 | 1.82E+04 |  |  |
| Medium oranges | IL5 - 3 | Heat | 98 | Direct | 2 | 1 | 3.03E+03 |  |  |
| Small translucent | IL5 - 3 | Heat | 98 | Direct | 2 | 8 | 2.42E+04 |  |  |
| Medium yellow | IL5 - 3 | Heat | 98 | Heat | 2 | 11 | 3.33E+04 | 68 | 2.06E+05 |
| Small white | IL5 - 3 | Heat | 98 | Heat | 2 | 12 | 3.64E+04 |  |  |
| Beige | IL5 - 3 | Heat | 98 | Heat | 2 | 13 | 3.94E+04 |  |  |
| Pink | IL5 - 3 | Heat | 98 | Heat | 2 | 32 | 9.70E+04 |  |  |
| Medium yellow | IL8 - 1 | Heat | 98 | Direct | 1 | 3 | 9.09E+02 | 61 | 1.85E+04 |
| Small yellow | IL8 - 1 | Heat | 98 | Direct | 1 | 5 | 1.52E+03 |  |  |
| Medium white | IL8 - 1 | Heat | 98 | Direct | 1 | 3 | 9.09E+02 |  |  |
| Small white | IL8 - 1 | Heat | 98 | Direct | 1 | 10 | 3.03E+03 |  |  |
| Beige | IL8 - 1 | Heat | 98 | Direct | 1 | 20 | 6.06E+03 |  |  |
| Medium melons | IL8 - 1 | Heat | 98 | Direct | 1 | 4 | 1.21E+03 |  |  |
| Pink | IL8 - 1 | Heat | 98 | Direct | 1 | 2 | 6.06E+02 |  |  |
| Translucent yellow | IL8 - 1 | Heat | 98 | Direct | 1 | 14 | 4.24E+03 |  |  |
| Small yellow | IL8 - 1 | Heat | 98 | Heat | 1 | 1 | 3.03E+02 | 21 | 6.36E+03 |
| Small white | IL8 - 1 | Heat | 98 | Heat | 1 | 7 | 2.12E+03 |  |  |
| Beige | IL8 - 1 | Heat | 98 | Heat | 1 | 7 | 2.12E+03 |  |  |
| Medium oranges | IL8 - 1 | Heat | 98 | Heat | 1 | 4 | 1.21E+03 |  |  |
| Translucent yellow | IL8 - 1 | Heat | 98 | Heat | 1 | 1 | 3.03E+02 |  |  |
| Small translucent | IL8 - 1 | Heat | 98 | Heat | 1 | 1 | 3.03E+02 |  |  |
| Small yellow | IL8 - 2 | Heat | 98 | Direct | 2 | 44 | 1.33E+05 | 208 | 6.30E+05 |
| Small white | IL8 - 2 | Heat | 98 | Direct | 2 | 26 | 7.88E+04 |  |  |
| Beige | IL8 - 2 | Heat | 98 | Direct | 2 | 24 | 7.27E+04 |  |  |
| Medium melons | IL8 - 2 | Heat | 98 | Direct | 2 | 12 | 3.64E+04 |  |  |
| Small melons | IL8 - 2 | Heat | 98 | Direct | 2 | 32 | 9.70E+04 |  |  |
| Medium oranges | IL8 - 2 | Heat | 98 | Direct | 2 | 52 | 1.58E+05 |  |  |
| Pink | IL8 - 2 | Heat | 98 | Direct | 2 | 1 | 3.03E+03 |  |  |
| Translucent yellow | IL8 - 2 | Heat | 98 | Direct | 2 | 16 | 4.85E+04 |  |  |
| Medium white | IL8 - 2 | Heat | 98 | Direct | 2 | 1 | 3.03E+03 |  |  |
| Medium yellow | IL8 - 2 | Heat | 98 | Heat | 2 | 23 | 6.97E+04 | 83 | 2.52E+05 |
| Small yellow | IL8 - 2 | Heat | 98 | Heat | 2 | 2 | 6.06E+03 |  |  |
| Small white | IL8 - 2 | Heat | 98 | Heat | 2 | 5 | 1.52E+04 |  |  |
| Small melons | IL8 - 2 | Heat | 98 | Heat | 2 | 1 | 3.03E+03 |  |  |
| Pink | IL8 - 2 | Heat | 98 | Heat | 2 | 1 | 3.03E+03 |  |  |
| Translucent yellow | IL8 - 2 | Heat | 98 | Heat | 2 | 51 | 1.55E+05 |  |  |
| Medium yellow | IL8 - 3 | Heat | 98 | Direct | 2 | 12 | 3.64E+04 | 142 | 4.30E+05 |
| Medium white | IL8 - 3 | Heat | 98 | Direct | 2 | 1 | 3.03E+03 |  |  |
| Small white | IL8 - 3 | Heat | 98 | Direct | 2 | 96 | 2.91E+05 |  |  |
| Beige | IL8 - 3 | Heat | 98 | Direct | 2 | 4 | 1.21E+04 |  |  |
| Medium melons | IL8 - 3 | Heat | 98 | Direct | 2 | 2 | 6.06E+03 |  |  |
| Medium oranges | IL8 - 3 | Heat | 98 | Direct | 2 | 23 | 6.97E+04 |  |  |
| Pink | IL8 - 3 | Heat | 98 | Direct | 2 | 4 | 1.21E+04 |  |  |
| Small yellow | IL8 - 3 | Heat | 98 | Heat | 1 | 6 | 1.82E+03 | 122 | 3.70E+04 |
| Small white | IL8 - 3 | Heat | 98 | Heat | 1 | 4 | 1.21E+03 |  |  |
| Beige | IL8 - 3 | Heat | 98 | Heat | 1 | 16 | 4.85E+03 |  |  |
| Small melons | IL8 - 3 | Heat | 98 | Heat | 1 | 6 | 1.82E+03 |  |  |
| Pink | IL8 - 3 | Heat | 98 | Heat | 1 | 8 | 2.42E+03 |  |  |
| Translucent yellow | IL8 - 3 | Heat | 98 | Heat | 1 | 82 | 2.48E+04 |  |  |
| Mesocosm experiments were established at three sampling sites (IL4, IL5, and IL8), with three mesocosms per site (1–3). One set was left untreated to preserve the intact microbial community, while the other was heat-treated to enrich for heat-resistant endospores. Samples were collected at multiple time points over a 98-week period, and colony diversity based on color and morphology was recorded for all mesocosm replicates. For each sampling time, serial dilutions were plated (33 µL) both directly and after heat treatment (80 °C for 30 min). | | | | | | | | | |

| **Supplementary Table 12**. Resume of colony counts and sporulation percentages from mesocosm experiments under heat-treated (spore-enriched) and non-heated (intact community) conditions. | | | | | | | |
| --- | --- | --- | --- | --- | --- | --- | --- |
| **Site** | **Replica** | **Mesocosm treatment** | **Time** | **Direct** | **Heat** | **Sporulation percent raw** | **Sporulation percent capped** |
| IL4 | R1 | Untreated | 0 | 10909.1 | 101.0 | 0.9 | 0.9 |
| IL4 | R1 | Untreated | 1 | 939.4 | 30.3 | 3.2 | 3.2 |
| IL4 | R1 | Untreated | 2 | 1484.8 | 121.2 | 8.2 | 8.2 |
| IL4 | R1 | Untreated | 3 | 212.1 | 30.3 | 14.3 | 14.3 |
| IL4 | R1 | Untreated | 4 | 1151.5 | 636.4 | 55.3 | 55.3 |
| IL4 | R1 | Untreated | 5 | 303.0 | 1454.5 | 480.0 | 100.0 |
| IL4 | R1 | Untreated | 6 | 60.6 | 121.2 | 200.0 | 100.0 |
| IL4 | R1 | Untreated | 7 | 303.0 | 30.3 | 10.0 | 10.0 |
| IL4 | R1 | Untreated | 8 | 2575.8 | 697.0 | 27.1 | 27.1 |
| IL4 | R1 | Untreated | 15 | 4303.0 | 697.0 | 16.2 | 16.2 |
| IL4 | R1 | Untreated | 32 | 5090.9 | 90.9 | 1.8 | 1.8 |
| IL4 | R1 | Untreated | 63 | 9697.0 | 303.0 | 3.1 | 3.1 |
| IL4 | R1 | Untreated | 81 | 11212.1 | 60.6 | 0.5 | 0.5 |
| IL4 | R1 | Untreated | 98 | 424.2 | 0.0 | 0.0 | 0.0 |
| IL4 | R2 | Untreated | 0 | 4040.4 | 111.1 | 2.8 | 2.8 |
| IL4 | R2 | Untreated | 1 | 5757.6 | 0.0 | 0.0 | 0.0 |
| IL4 | R2 | Untreated | 2 | 2393.9 | 30.3 | 1.3 | 1.3 |
| IL4 | R2 | Untreated | 3 | 636.4 | 60.6 | 9.5 | 9.5 |
| IL4 | R2 | Untreated | 4 | 5636.4 | 303.0 | 5.4 | 5.4 |
| IL4 | R2 | Untreated | 5 | 1030.3 | 30.3 | 2.9 | 2.9 |
| IL4 | R2 | Untreated | 6 | 272.7 | 212.1 | 77.8 | 77.8 |
| IL4 | R2 | Untreated | 7 | 575.8 | 0.0 | 0.0 | 0.0 |
| IL4 | R2 | Untreated | 8 | 969.7 | 151.5 | 15.6 | 15.6 |
| IL4 | R2 | Untreated | 15 | 6060.6 | 1303.0 | 21.5 | 21.5 |
| IL4 | R2 | Untreated | 32 | 2878.8 | 575.8 | 20.0 | 20.0 |
| IL4 | R2 | Untreated | 63 | 15454.5 | 939.4 | 6.1 | 6.1 |
| IL4 | R2 | Untreated | 81 | 260606.1 | 1212.1 | 0.5 | 0.5 |
| IL4 | R2 | Untreated | 98 | 41818.2 | 606.1 | 1.4 | 1.4 |
| IL4 | R3 | Untreated | 0 | 2000.0 | 90.9 | 4.5 | 4.5 |
| IL4 | R3 | Untreated | 1 | 1363.6 | 60.6 | 4.4 | 4.4 |
| IL4 | R3 | Untreated | 2 | 6060.6 | 30303.0 | 500.0 | 100.0 |
| IL4 | R3 | Untreated | 3 | 3030.3 | 60.6 | 2.0 | 2.0 |
| IL4 | R3 | Untreated | 4 | 1575.8 | 60.6 | 3.8 | 3.8 |
| IL4 | R3 | Untreated | 5 | 636.4 | 60.6 | 9.5 | 9.5 |
| IL4 | R3 | Untreated | 7 | 212.1 | 0.0 | 0.0 | 0.0 |
| IL4 | R3 | Untreated | 8 | 697.0 | 515.2 | 73.9 | 73.9 |
| IL4 | R3 | Untreated | 15 | 5303.0 | 1000.0 | 18.9 | 18.9 |
| IL4 | R3 | Untreated | 32 | 333333.3 | 757.6 | 0.2 | 0.2 |
| IL4 | R3 | Untreated | 63 | 16060.6 | 92121.2 | 573.6 | 100.0 |
| IL4 | R3 | Untreated | 81 | 7818.2 | 575.8 | 7.4 | 7.4 |
| IL4 | R3 | Untreated | 98 | 1697.0 | 121.2 | 7.1 | 7.1 |
| IL5 | R1 | Untreated | 0 | 4464.6 | 141.4 | 3.2 | 3.2 |
| IL5 | R1 | Untreated | 1 | 4727.3 | 60.6 | 1.3 | 1.3 |
| IL5 | R1 | Untreated | 2 | 2030.3 | 0.0 | 0.0 | 0.0 |
| IL5 | R1 | Untreated | 3 | 636.4 | 212.1 | 33.3 | 33.3 |
| IL5 | R1 | Untreated | 4 | 2757.6 | 757.6 | 27.5 | 27.5 |
| IL5 | R1 | Untreated | 5 | 3787.9 | 787.9 | 20.8 | 20.8 |
| IL5 | R1 | Untreated | 6 | 363.6 | 90.9 | 25.0 | 25.0 |
| IL5 | R1 | Untreated | 7 | 515.2 | 60.6 | 11.8 | 11.8 |
| IL5 | R1 | Untreated | 8 | 2151.5 | 545.5 | 25.4 | 25.4 |
| IL5 | R1 | Untreated | 15 | 342424.2 | 23939.4 | 7.0 | 7.0 |
| IL5 | R1 | Untreated | 32 | 830303.0 | 545.5 | 0.1 | 0.1 |
| IL5 | R1 | Untreated | 63 | 148484.8 | 4000.0 | 2.7 | 2.7 |
| IL5 | R1 | Untreated | 81 | 545454.5 | 3878.8 | 0.7 | 0.7 |
| IL5 | R1 | Untreated | 98 | 90909.1 | 454.5 | 0.5 | 0.5 |
| IL5 | R2 | Untreated | 0 | 16363.6 | 0.0 | 0.0 | 0.0 |
| IL5 | R2 | Untreated | 1 | 4363.6 | 272.7 | 6.3 | 6.3 |
| IL5 | R2 | Untreated | 2 | 1787.9 | 333.3 | 18.6 | 18.6 |
| IL5 | R2 | Untreated | 3 | 484.8 | 60.6 | 12.5 | 12.5 |
| IL5 | R2 | Untreated | 4 | 8515.2 | 818.2 | 9.6 | 9.6 |
| IL5 | R2 | Untreated | 5 | 4727.3 | 1242.4 | 26.3 | 26.3 |
| IL5 | R2 | Untreated | 6 | 9090.9 | 424.2 | 4.7 | 4.7 |
| IL5 | R2 | Untreated | 7 | 3030.3 | 697.0 | 23.0 | 23.0 |
| IL5 | R2 | Untreated | 8 | 5151.5 | 1424.2 | 27.6 | 27.6 |
| IL5 | R2 | Untreated | 15 | 21818.2 | 1697.0 | 7.8 | 7.8 |
| IL5 | R2 | Untreated | 32 | 22727.3 | 697.0 | 3.1 | 3.1 |
| IL5 | R2 | Untreated | 63 | 4242.4 | 3030.3 | 71.4 | 71.4 |
| IL5 | R2 | Untreated | 81 | 42424.2 | 12121.2 | 28.6 | 28.6 |
| IL5 | R2 | Untreated | 98 | 909.1 | 363.6 | 40.0 | 40.0 |
| IL5 | R3 | Untreated | 0 | 13838.4 | 50.5 | 0.4 | 0.4 |
| IL5 | R3 | Untreated | 1 | 3515.2 | 30.3 | 0.9 | 0.9 |
| IL5 | R3 | Untreated | 2 | 1272.7 | 272.7 | 21.4 | 21.4 |
| IL5 | R3 | Untreated | 3 | 606.1 | 1000.0 | 165.0 | 100.0 |
| IL5 | R3 | Untreated | 4 | 3727.3 | 878.8 | 23.6 | 23.6 |
| IL5 | R3 | Untreated | 5 | 2121.2 | 1393.9 | 65.7 | 65.7 |
| IL5 | R3 | Untreated | 6 | 636.4 | 909.1 | 142.9 | 100.0 |
| IL5 | R3 | Untreated | 7 | 90.9 | 606.1 | 666.7 | 100.0 |
| IL5 | R3 | Untreated | 8 | 5757.6 | 2545.5 | 44.2 | 44.2 |
| IL5 | R3 | Untreated | 15 | 24242.4 | 2060.6 | 8.5 | 8.5 |
| IL5 | R3 | Untreated | 32 | 26363.6 | 1818.2 | 6.9 | 6.9 |
| IL5 | R3 | Untreated | 63 | 17878.8 | 3272.7 | 18.3 | 18.3 |
| IL5 | R3 | Untreated | 81 | 33030.3 | 3303.0 | 10.0 | 10.0 |
| IL5 | R3 | Untreated | 98 | 8454.5 | 333.3 | 3.9 | 3.9 |
| IL8 | R1 | Untreated | 0 | 52323.2 | 262.6 | 0.5 | 0.5 |
| IL8 | R1 | Untreated | 1 | 7636.4 | 242.4 | 3.2 | 3.2 |
| IL8 | R1 | Untreated | 2 | 1909.1 | 0.0 | 0.0 | 0.0 |
| IL8 | R1 | Untreated | 3 | 424.2 | 303.0 | 71.4 | 71.4 |
| IL8 | R1 | Untreated | 4 | 2666.7 | 515.2 | 19.3 | 19.3 |
| IL8 | R1 | Untreated | 5 | 2787.9 | 484.8 | 17.4 | 17.4 |
| IL8 | R1 | Untreated | 6 | 3727.3 | 969.7 | 26.0 | 26.0 |
| IL8 | R1 | Untreated | 7 | 30.3 | 575.8 | 1900.0 | 100.0 |
| IL8 | R1 | Untreated | 8 | 3242.4 | 1303.0 | 40.2 | 40.2 |
| IL8 | R1 | Untreated | 15 | 4181.8 | 787.9 | 18.8 | 18.8 |
| IL8 | R1 | Untreated | 32 | 16363.6 | 181.8 | 1.1 | 1.1 |
| IL8 | R1 | Untreated | 63 | 1909.1 | 363.6 | 19.0 | 19.0 |
| IL8 | R1 | Untreated | 81 | 88181.8 | 242.4 | 0.3 | 0.3 |
| IL8 | R1 | Untreated | 98 | 6969.7 | 90.9 | 1.3 | 1.3 |
| IL8 | R2 | Untreated | 0 | 57575.8 | 575.8 | 1.0 | 1.0 |
| IL8 | R2 | Untreated | 1 | 4757.6 | 272.7 | 5.7 | 5.7 |
| IL8 | R2 | Untreated | 2 | 1878.8 | 0.0 | 0.0 | 0.0 |
| IL8 | R2 | Untreated | 3 | 1666.7 | 242.4 | 14.5 | 14.5 |
| IL8 | R2 | Untreated | 4 | 1939.4 | 90.9 | 4.7 | 4.7 |
| IL8 | R2 | Untreated | 5 | 3121.2 | 1000.0 | 32.0 | 32.0 |
| IL8 | R2 | Untreated | 6 | 121.2 | 363.6 | 300.0 | 100.0 |
| IL8 | R2 | Untreated | 7 | 0.0 | 1787.9 |  |  |
| IL8 | R2 | Untreated | 8 | 2151.5 | 575.8 | 26.8 | 26.8 |
| IL8 | R2 | Untreated | 15 | 4697.0 | 484.8 | 10.3 | 10.3 |
| IL8 | R2 | Untreated | 32 | 3272.7 | 1090.9 | 33.3 | 33.3 |
| IL8 | R2 | Untreated | 63 | 31818.2 | 1333.3 | 4.2 | 4.2 |
| IL8 | R2 | Untreated | 81 | 303.0 | 151.5 | 50.0 | 50.0 |
| IL8 | R2 | Untreated | 98 | 515.2 | 151.5 | 29.4 | 29.4 |
| IL8 | R3 | Untreated | 0 | 12121.2 | 757.6 | 6.3 | 6.3 |
| IL8 | R3 | Untreated | 1 | 23333.3 | 212.1 | 0.9 | 0.9 |
| IL8 | R3 | Untreated | 2 | 2030.3 | 242.4 | 11.9 | 11.9 |
| IL8 | R3 | Untreated | 3 | 1303.0 | 545.5 | 41.9 | 41.9 |
| IL8 | R3 | Untreated | 4 | 1454.5 | 2939.4 | 202.1 | 100.0 |
| IL8 | R3 | Untreated | 5 | 4636.4 | 1484.8 | 32.0 | 32.0 |
| IL8 | R3 | Untreated | 6 | 36363.6 | 424.2 | 1.2 | 1.2 |
| IL8 | R3 | Untreated | 7 | 30.3 | 272.7 | 900.0 | 100.0 |
| IL8 | R3 | Untreated | 8 | 3818.2 | 666.7 | 17.5 | 17.5 |
| IL8 | R3 | Untreated | 15 | 2818.2 | 757.6 | 26.9 | 26.9 |
| IL8 | R3 | Untreated | 32 | 6242.4 | 272.7 | 4.4 | 4.4 |
| IL8 | R3 | Untreated | 63 | 8090.9 | 121.2 | 1.5 | 1.5 |
| IL8 | R3 | Untreated | 81 | 24939.4 | 151.5 | 0.6 | 0.6 |
| IL8 | R3 | Untreated | 98 | 13939.4 | 181.8 | 1.3 | 1.3 |
| IL4 | R1 | Heat | 0 | 575.8 | 484.8 | 84.2 | 84.2 |
| IL4 | R1 | Heat | 1 | 124242.4 | 87878.8 | 70.7 | 70.7 |
| IL4 | R1 | Heat | 2 | 333333.3 | 121212.1 | 36.4 | 36.4 |
| IL4 | R1 | Heat | 3 | 212121.2 | 175757.6 | 82.9 | 82.9 |
| IL4 | R1 | Heat | 4 | 545454.5 | 666666.7 | 122.2 | 100.0 |
| IL4 | R1 | Heat | 5 | 1242424.2 | 906060.6 | 72.9 | 72.9 |
| IL4 | R1 | Heat | 6 | 34242.4 | 60303.0 | 176.1 | 100.0 |
| IL4 | R1 | Heat | 7 | 181818.2 | 72727.3 | 40.0 | 40.0 |
| IL4 | R1 | Heat | 8 | 481818.2 | 300000.0 | 62.3 | 62.3 |
| IL4 | R1 | Heat | 15 | 1666666.7 | 481818.2 | 28.9 | 28.9 |
| IL4 | R1 | Heat | 32 | 581818.2 | 354545.5 | 60.9 | 60.9 |
| IL4 | R1 | Heat | 63 | 1060606.1 | 72727.3 | 6.9 | 6.9 |
| IL4 | R1 | Heat | 81 | 151515.2 | 878.8 | 0.6 | 0.6 |
| IL4 | R1 | Heat | 98 | 1666666.7 | 466666.7 | 28.0 | 28.0 |
| IL4 | R2 | Heat | 0 | 90.9 | 2727.3 | 3000.0 | 100.0 |
| IL4 | R2 | Heat | 1 | 124242.4 | 87878.8 | 70.7 | 70.7 |
| IL4 | R2 | Heat | 2 | 521212.1 | 230303.0 | 44.2 | 44.2 |
| IL4 | R2 | Heat | 3 | 51515.2 | 8757.6 | 17.0 | 17.0 |
| IL4 | R2 | Heat | 4 | 512121.2 | 524242.4 | 102.4 | 100.0 |
| IL4 | R2 | Heat | 5 | 324242.4 | 248484.8 | 76.6 | 76.6 |
| IL4 | R2 | Heat | 6 | 142424.2 | 61212.1 | 43.0 | 43.0 |
| IL4 | R2 | Heat | 7 | 524242.4 | 560606.1 | 106.9 | 100.0 |
| IL4 | R2 | Heat | 8 | 724242.4 | 424242.4 | 58.6 | 58.6 |
| IL4 | R2 | Heat | 15 | 630303.0 | 345454.5 | 54.8 | 54.8 |
| IL4 | R2 | Heat | 32 | 478787.9 | 272727.3 | 57.0 | 57.0 |
| IL4 | R2 | Heat | 63 | 375757.6 | 318181.8 | 84.7 | 84.7 |
| IL4 | R2 | Heat | 81 | 1242424.2 | 524242.4 | 42.2 | 42.2 |
| IL4 | R2 | Heat | 98 | 1393939.4 | 393939.4 | 28.3 | 28.3 |
| IL4 | R3 | Heat | 0 | 242.4 | 121.2 | 50.0 | 50.0 |
| IL4 | R3 | Heat | 1 | 400000.0 | 118181.8 | 29.5 | 29.5 |
| IL4 | R3 | Heat | 2 | 448484.8 | 212121.2 | 47.3 | 47.3 |
| IL4 | R3 | Heat | 3 | 239393.9 | 56666.7 | 23.7 | 23.7 |
| IL4 | R3 | Heat | 4 | 466666.7 | 387878.8 | 83.1 | 83.1 |
| IL4 | R3 | Heat | 5 | 412121.2 | 369697.0 | 89.7 | 89.7 |
| IL4 | R3 | Heat | 6 | 396969.7 | 333333.3 | 84.0 | 84.0 |
| IL4 | R3 | Heat | 7 | 254545.5 | 387878.8 | 152.4 | 100.0 |
| IL4 | R3 | Heat | 8 | 575757.6 | 787878.8 | 136.8 | 100.0 |
| IL4 | R3 | Heat | 15 | 606060.6 | 533333.3 | 88.0 | 88.0 |
| IL4 | R3 | Heat | 32 | 372727.3 | 324242.4 | 87.0 | 87.0 |
| IL4 | R3 | Heat | 63 | 496969.7 | 496969.7 | 100.0 | 100.0 |
| IL4 | R3 | Heat | 81 | 1212121.2 | 606060.6 | 50.0 | 50.0 |
| IL4 | R3 | Heat | 98 | 4060606.1 | 1027272.7 | 25.3 | 25.3 |
| IL5 | R1 | Heat | 0 | 2212.1 | 1484.8 | 67.1 | 67.1 |
| IL5 | R1 | Heat | 1 | 478787.9 | 31515.2 | 6.6 | 6.6 |
| IL5 | R1 | Heat | 2 | 263636.4 | 78787.9 | 29.9 | 29.9 |
| IL5 | R1 | Heat | 3 | 245454.5 | 178787.9 | 72.8 | 72.8 |
| IL5 | R1 | Heat | 4 | 1666666.7 | 315151.5 | 18.9 | 18.9 |
| IL5 | R1 | Heat | 5 | 381818.2 | 366666.7 | 96.0 | 96.0 |
| IL5 | R1 | Heat | 6 | 257575.8 | 412121.2 | 160.0 | 100.0 |
| IL5 | R1 | Heat | 7 | 533333.3 | 575757.6 | 108.0 | 100.0 |
| IL5 | R1 | Heat | 8 | 390909.1 | 448484.8 | 114.7 | 100.0 |
| IL5 | R1 | Heat | 15 | 2030303.0 | 1424242.4 | 70.1 | 70.1 |
| IL5 | R1 | Heat | 32 | 218181.8 | 63636.4 | 29.2 | 29.2 |
| IL5 | R1 | Heat | 63 | 696969.7 | 678787.9 | 97.4 | 97.4 |
| IL5 | R1 | Heat | 81 | 151515.2 | 139393.9 | 92.0 | 92.0 |
| IL5 | R1 | Heat | 98 | 260606.1 | 251515.2 | 96.5 | 96.5 |
| IL5 | R2 | Heat | 0 | 2545.5 | 1727.3 | 67.9 | 67.9 |
| IL5 | R2 | Heat | 1 | 493939.4 | 56969.7 | 11.5 | 11.5 |
| IL5 | R2 | Heat | 2 | 200000.0 | 63636.4 | 31.8 | 31.8 |
| IL5 | R2 | Heat | 3 | 76666.7 | 15757.6 | 20.6 | 20.6 |
| IL5 | R2 | Heat | 4 | 460606.1 | 366666.7 | 79.6 | 79.6 |
| IL5 | R2 | Heat | 5 | 324242.4 | 587878.8 | 181.3 | 100.0 |
| IL5 | R2 | Heat | 6 | 390909.1 | 481818.2 | 123.3 | 100.0 |
| IL5 | R2 | Heat | 7 | 354545.5 | 278787.9 | 78.6 | 78.6 |
| IL5 | R2 | Heat | 8 | 433333.3 | 151515.2 | 35.0 | 35.0 |
| IL5 | R2 | Heat | 15 | 1181818.2 | 248484.8 | 21.0 | 21.0 |
| IL5 | R2 | Heat | 32 | 342424.2 | 393939.4 | 115.0 | 100.0 |
| IL5 | R2 | Heat | 63 | 77575.8 | 30303.0 | 39.1 | 39.1 |
| IL5 | R2 | Heat | 81 | 124242.4 | 40606.1 | 32.7 | 32.7 |
| IL5 | R2 | Heat | 98 | 21818.2 | 25151.5 | 115.3 | 100.0 |
| IL5 | R3 | Heat | 0 | 2545.5 | 1424.2 | 56.0 | 56.0 |
| IL5 | R3 | Heat | 1 | 478787.9 | 90909.1 | 19.0 | 19.0 |
| IL5 | R3 | Heat | 2 | 133333.3 | 22727.3 | 17.0 | 17.0 |
| IL5 | R3 | Heat | 3 | 357575.8 | 40000.0 | 11.2 | 11.2 |
| IL5 | R3 | Heat | 4 | 490909.1 | 696969.7 | 142.0 | 100.0 |
| IL5 | R3 | Heat | 5 | 606060.6 | 636363.6 | 105.0 | 100.0 |
| IL5 | R3 | Heat | 6 | 48787.9 | 52727.3 | 108.1 | 100.0 |
| IL5 | R3 | Heat | 7 | 878787.9 | 618181.8 | 70.3 | 70.3 |
| IL5 | R3 | Heat | 8 | 357575.8 | 333333.3 | 93.2 | 93.2 |
| IL5 | R3 | Heat | 15 | 1878787.9 | 212121.2 | 11.3 | 11.3 |
| IL5 | R3 | Heat | 32 | 436363.6 | 357575.8 | 81.9 | 81.9 |
| IL5 | R3 | Heat | 63 | 254545.5 | 48484.8 | 19.0 | 19.0 |
| IL5 | R3 | Heat | 81 | 200000.0 | 59393.9 | 29.7 | 29.7 |
| IL5 | R3 | Heat | 98 | 833333.3 | 206060.6 | 24.7 | 24.7 |
| IL8 | R1 | Heat | 0 | 4212.1 | 2454.5 | 58.3 | 58.3 |
| IL8 | R1 | Heat | 1 | 242424.2 | 230303.0 | 95.0 | 95.0 |
| IL8 | R1 | Heat | 2 | 53333.3 | 58787.9 | 110.2 | 100.0 |
| IL8 | R1 | Heat | 3 | 41818.2 | 69090.9 | 165.2 | 100.0 |
| IL8 | R1 | Heat | 4 | 469697.0 | 381818.2 | 81.3 | 81.3 |
| IL8 | R1 | Heat | 5 | 563636.4 | 430303.0 | 76.3 | 76.3 |
| IL8 | R1 | Heat | 6 | 22424.2 | 16060.6 | 71.6 | 71.6 |
| IL8 | R1 | Heat | 7 | 351515.2 | 309090.9 | 87.9 | 87.9 |
| IL8 | R1 | Heat | 8 | 672727.3 | 151515.2 | 22.5 | 22.5 |
| IL8 | R1 | Heat | 15 | 239393.9 | 245454.5 | 102.5 | 100.0 |
| IL8 | R1 | Heat | 32 | 878787.9 | 203030.3 | 23.1 | 23.1 |
| IL8 | R1 | Heat | 63 | 836363.6 | 448484.8 | 53.6 | 53.6 |
| IL8 | R1 | Heat | 81 | 36969.7 | 13030.3 | 35.2 | 35.2 |
| IL8 | R1 | Heat | 98 | 18484.8 | 6363.6 | 34.4 | 34.4 |
| IL8 | R2 | Heat | 0 | 2212.1 | 3272.7 | 147.9 | 100.0 |
| IL8 | R2 | Heat | 1 | 418181.8 | 321212.1 | 76.8 | 76.8 |
| IL8 | R2 | Heat | 2 | 436363.6 | 484848.5 | 111.1 | 100.0 |
| IL8 | R2 | Heat | 3 | 84242.4 | 69090.9 | 82.0 | 82.0 |
| IL8 | R2 | Heat | 4 | 369697.0 | 360606.1 | 97.5 | 97.5 |
| IL8 | R2 | Heat | 5 | 563636.4 | 387878.8 | 68.8 | 68.8 |
| IL8 | R2 | Heat | 6 | 0.0 | 6363.6 |  |  |
| IL8 | R2 | Heat | 7 | 384848.5 | 503030.3 | 130.7 | 100.0 |
| IL8 | R2 | Heat | 8 | 251515.2 | 163636.4 | 65.1 | 65.1 |
| IL8 | R2 | Heat | 15 | 848484.8 | 378787.9 | 44.6 | 44.6 |
| IL8 | R2 | Heat | 32 | 354545.5 | 287878.8 | 81.2 | 81.2 |
| IL8 | R2 | Heat | 63 | 1181818.2 | 909090.9 | 76.9 | 76.9 |
| IL8 | R2 | Heat | 81 | 1454545.5 | 203030.3 | 14.0 | 14.0 |
| IL8 | R2 | Heat | 98 | 630303.0 | 251515.2 | 39.9 | 39.9 |
| IL8 | R3 | Heat | 0 | 2424.2 | 2030.3 | 83.8 | 83.8 |
| IL8 | R3 | Heat | 1 | 648484.8 | 278787.9 | 43.0 | 43.0 |
| IL8 | R3 | Heat | 2 | 224242.4 | 293939.4 | 131.1 | 100.0 |
| IL8 | R3 | Heat | 3 | 41212.1 | 48484.8 | 117.6 | 100.0 |
| IL8 | R3 | Heat | 4 | 584848.5 | 224242.4 | 38.3 | 38.3 |
| IL8 | R3 | Heat | 5 | 472727.3 | 478787.9 | 101.3 | 100.0 |
| IL8 | R3 | Heat | 6 | 533333.3 | 209090.9 | 39.2 | 39.2 |
| IL8 | R3 | Heat | 7 | 606060.6 | 415151.5 | 68.5 | 68.5 |
| IL8 | R3 | Heat | 8 | 554545.5 | 200000.0 | 36.1 | 36.1 |
| IL8 | R3 | Heat | 15 | 660606.1 | 269697.0 | 40.8 | 40.8 |
| IL8 | R3 | Heat | 32 | 451515.2 | 275757.6 | 61.1 | 61.1 |
| IL8 | R3 | Heat | 63 | 2363636.4 | 630303.0 | 26.7 | 26.7 |
| IL8 | R3 | Heat | 81 | 196969.7 | 169697.0 | 86.2 | 86.2 |
| IL8 | R3 | Heat | 98 | 430303.0 | 36969.7 | 8.6 | 8.6 |
| Colony counts and total sporulation percentages were calculated for each replicate (1–3) at each site (IL4, IL5, and IL8). Sporulation percentage was calculated by dividing the direct CFU/mL count by the heat-treated CFU/mL count and multiplying by 100. Sporulation values exceeding 100% were capped at 100% and used in the mean sporulation values shown in Supplementary Figures S8 and S9. | | | | | | | |
