# Supplementary material for: Dormancy as a Signature of Microbial Community Disassembly during Hydrological Collapse in a Desert Oasis
Source: Microb Ecol. 2026 Apr 17;89(1):114. doi: 10.1007/s00248-026-02744-z (PMC13212791; doi:10.1007/s00248-026-02744-z)
Supplement: Supplementary file 2 — Supplementary Material 2 [file 248_2026_2744_MOESM2_ESM.pdf]

Supplementary Figures

This document contains 9 supplementary figures

Supplementary Figure 1

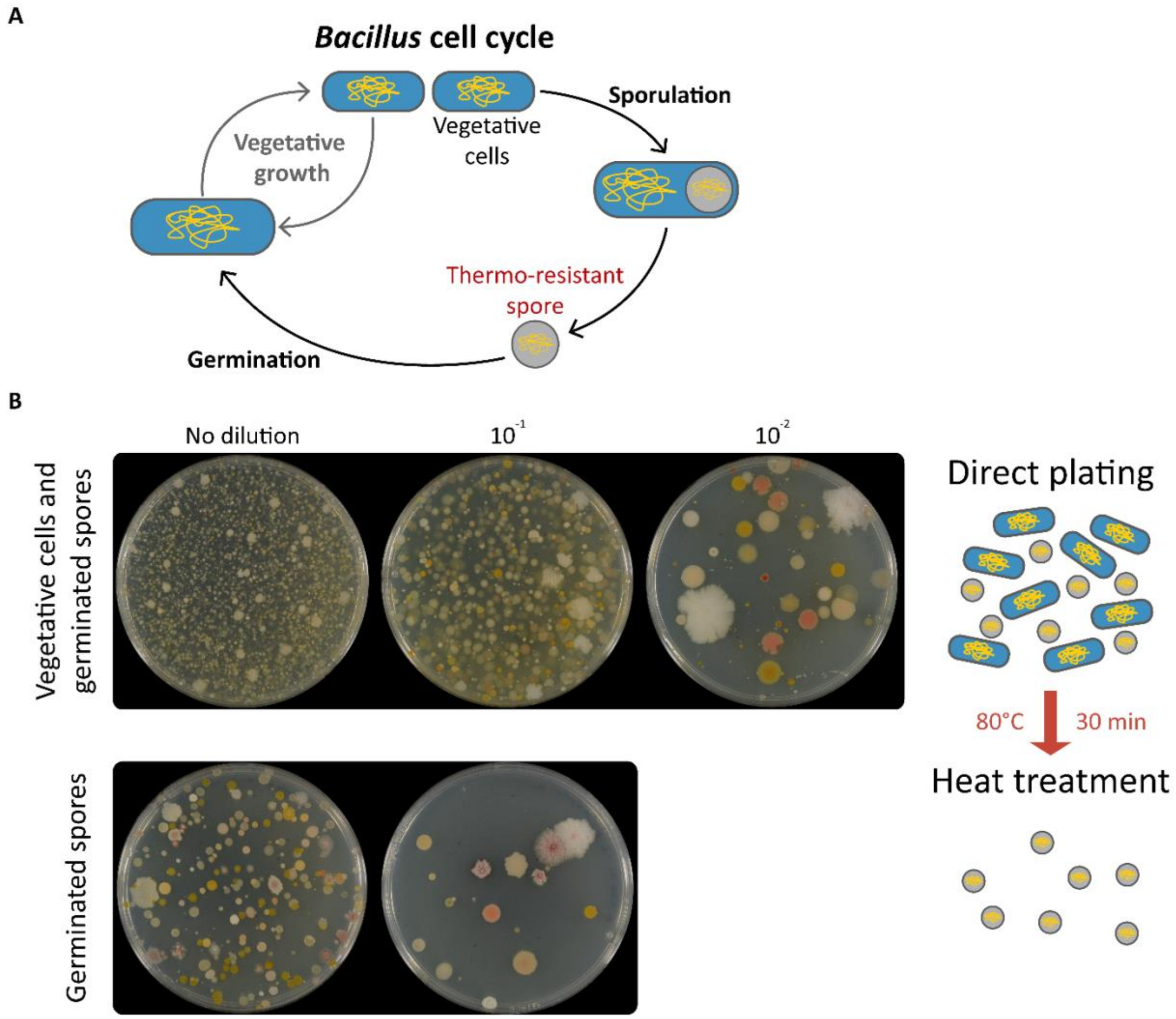

Supplementary Fig. 1. Detection of vegetative cells and spores by direct and heat-treated plating.

Representative colony counts obtained from two cultivation approaches. Direct plating recovers both vegetative cells and spores, thereby reflecting the total viable count. Heat-treated plating (80 °C, 30 min) inactivates vegetative cells, allowing only heat-resistant spores to germinate. In this example, direct plating at the 10^{-3} dilution yielded ~3.1×10^5 CFU, whereas heat-treated plating at the 10^{-1} dilution yielded ~2×10^3 CFU, corresponding to <1% sporulation. Isolates were plated on Marine Medium and incubated at 28 °C for 5 days. The example corresponds to a 2019 sediment sample. A schematic of the *Bacillus* life

cycle is included, showing binary fission under favorable conditions and sporulation under stress.

Supplementary Figure 2

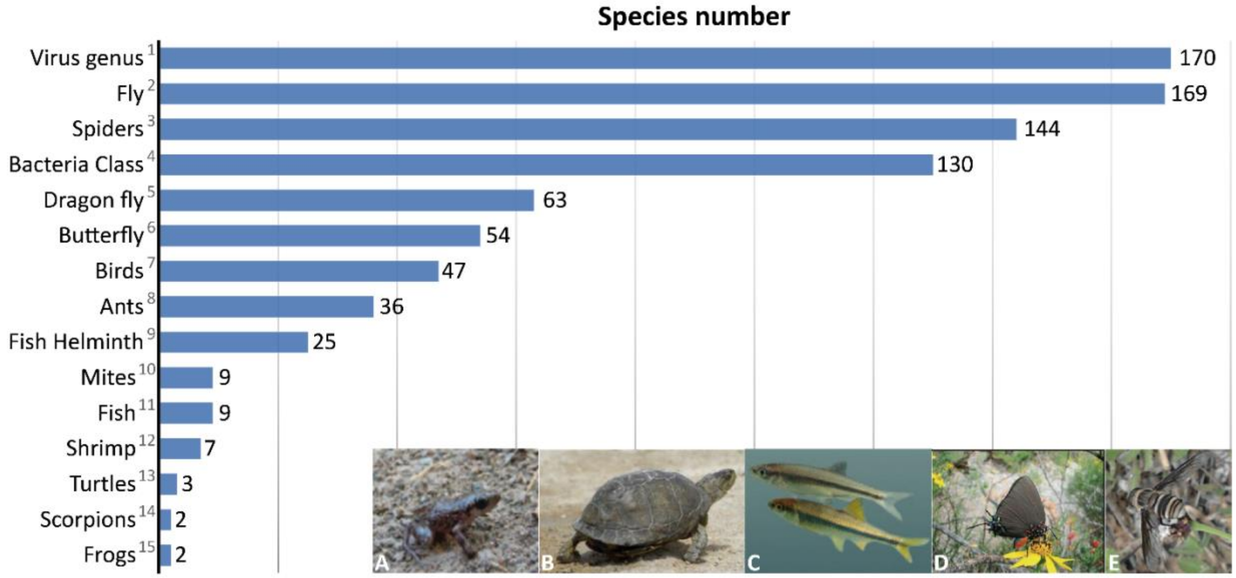

Supplementary Fig. 2. Inventory of endemic biodiversity in the Cuatro Ciénegas Basin (2013).

The chart summarizes the number of species comprising the basin's unique biodiversity. Representative photographs include: (A) *Eleutherodactylus* sp. nov. (frog), (B) T*errapene coahuila* (turtle), (C) *Cyprinella xanthicara* (fish), (D) *Atlides halesus* (butterfly; Cramer), and (E) *Exoprosopa* sp. (bee). A reference is provided to the publication where detailed results on the diversity of viruses, bacteria, mites, fish, frogs, and other organisms were previously reported. References: 1.- Isa, P. *et al*., 2018, 2.- Ávalos-Hernández *et al*., 2019, 3.- Bizuet-Flores *et al*., 2015, 4.- Souza *et al*., 2018, 5.- Ortega-Salas, H., and González-Soriano, E., 2019, 6.- Hernández-Jerónimo *et al*., 2019, 7.- Corcuera *et al*., 2019, 8.- Janda *et al*., 2019, 9.- Pérez-Ponce de León, G., and Aguilar-Aguilar, R., 2019, 10.- Paredes-León, R., 2019, 11.- Espinosa-Pérez, H., and Lambarri-Martínez, C. 2019, 12.- García-Vázquez *et al*., 2022, 13.- García-Vázquez *et al*., 2021, 14.- Francke B., O.F. 2019, 15.- García-Vázquez *et al*., 2019.

Supplementary Figure 3

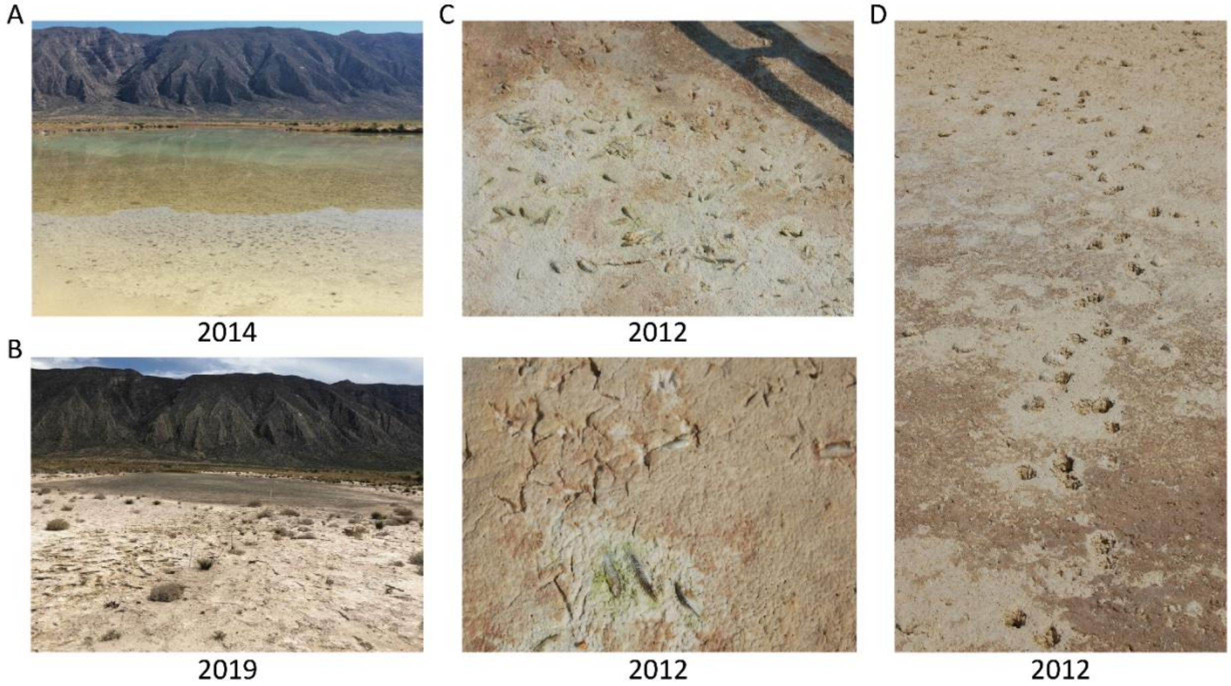

Supplementary Fig. 3. Ecological impacts of desiccation in the Churince Intermediate Lagoon.

Photographs show: (A) surface water still present in 2014; (B) by 2019, water restricted to the lagoon's center; (C) dead fish and bird footprints on the exposed lagoon bed; and (D) turtle tracks indicating individuals followed the receding water and likely perished.

Supplementary Figure 4

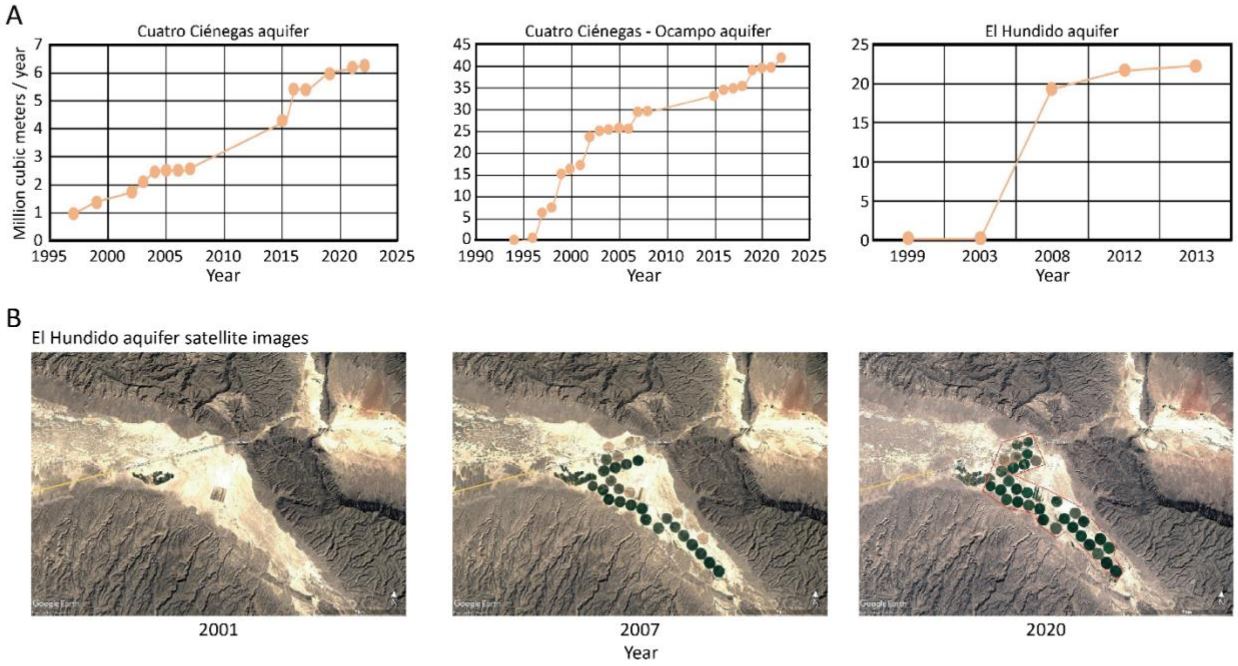

Supplementary Fig. 4. Expansion of alfalfa cultivation and increasing water extraction from Cuatro Ciénegas aquifers.

(A) Annual water consumption for agricultural practices (millions of cubic meters per year) in three aquifers: Cuatro Ciénegas, Ocampo, and El Hundido (data from IMTA). (B) Google Earth images from 2001, 2007, and 2020 showing circular alfalfa fields (rondines) in the Hundido Valley. At these time points, 21 rondines were active in 2007 and 29 in 2020, illustrating the expansion of alfalfa cultivation.

Supplementary Figure 5

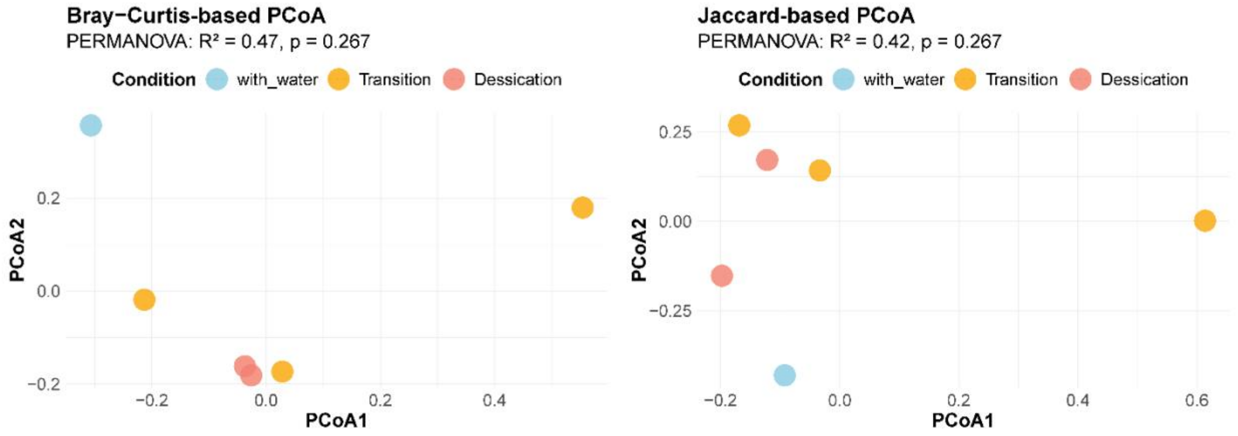

Supplementary Fig. 5. Beta diversity of culturable bacterial communities across ecological phases.

(A) Principal Coordinates Analysis (PCoA) based on Bray–Curtis dissimilarity, illustrating clustering of bacterial communities by ecological phase (With Water, Transition, and Desiccation). PERMANOVA results (R^2 and p-values) are shown. (B) PCoA based on Jaccard dissimilarity, showing community clustering by hydrological condition. PERMANOVA results (R^2 and p-values) are indicated.

Supplementary Figure 6

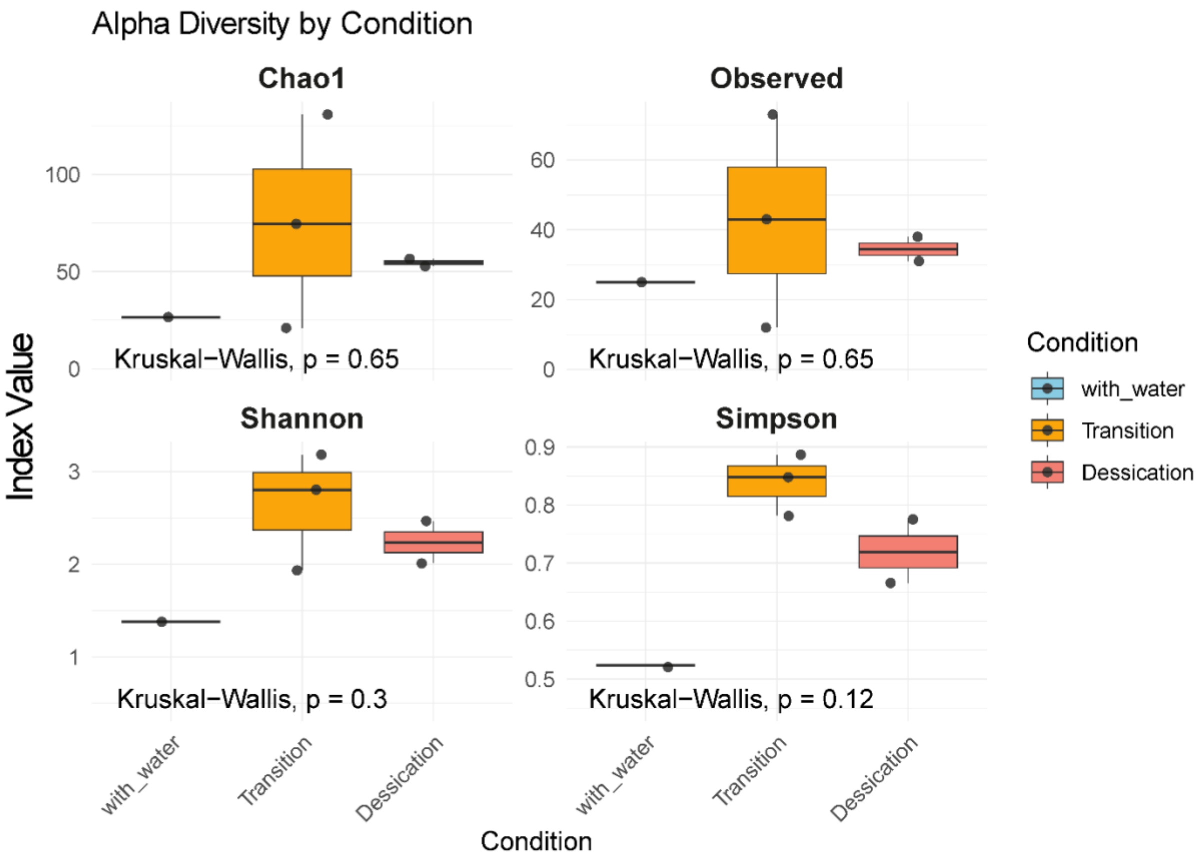

Supplementary Fig. 6. Alpha diversity of culturable bacterial communities across ecological phases.

Box plots show the distribution of alpha diversity indices for bacterial isolates from Churince Intermediate Lagoon sediments across three ecological phases: With Water, Transition, and Desiccation. (A) Chao1 richness estimator. (B) Observed species index. (C) Shannon diversity index. (D) Simpson diversity index. Statistical comparisons among phases were performed using the Kruskal–Wallis test; p-values are shown on each plot.

Supplementary Figure 7

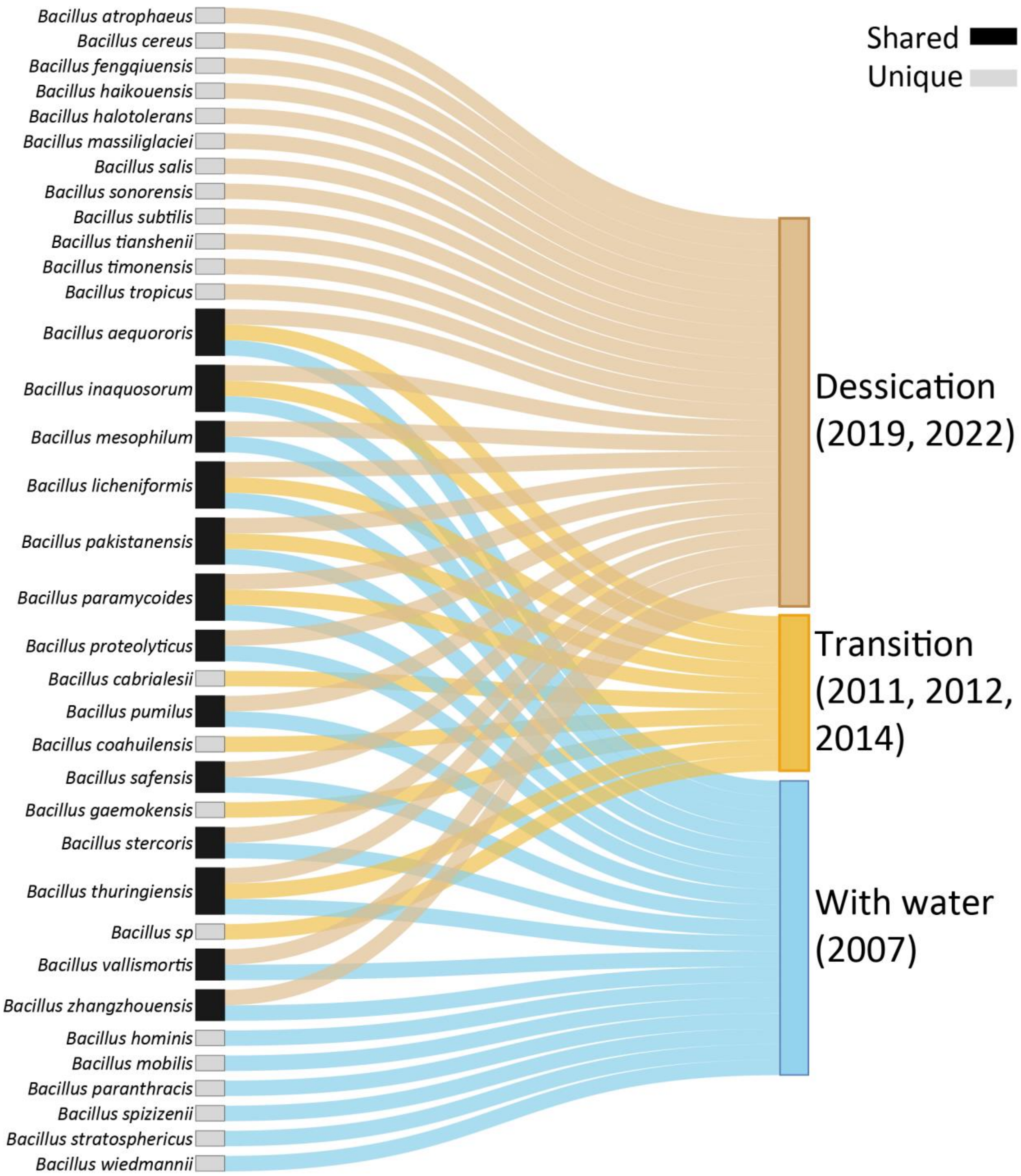

Supplementary Fig. 7. Distribution of culturable *Bacillus* species across ecological phases in sediments of the Churince Intermediate Lagoon. The Sankey diagram illustrates the presence and overlaps of *Bacillus* species identified from culturable isolates collected during three ecological phases: With Water (2007 sampling), Transition

(intermediate desiccation; 2011, 2012, and 2014 sampling), and Desiccation (fully dry; 2019 and 2022 sampling). Grey boxes denote species unique to a single phase, whereas black boxes represent species occurring in at least two phases.

Supplementary Figure 8

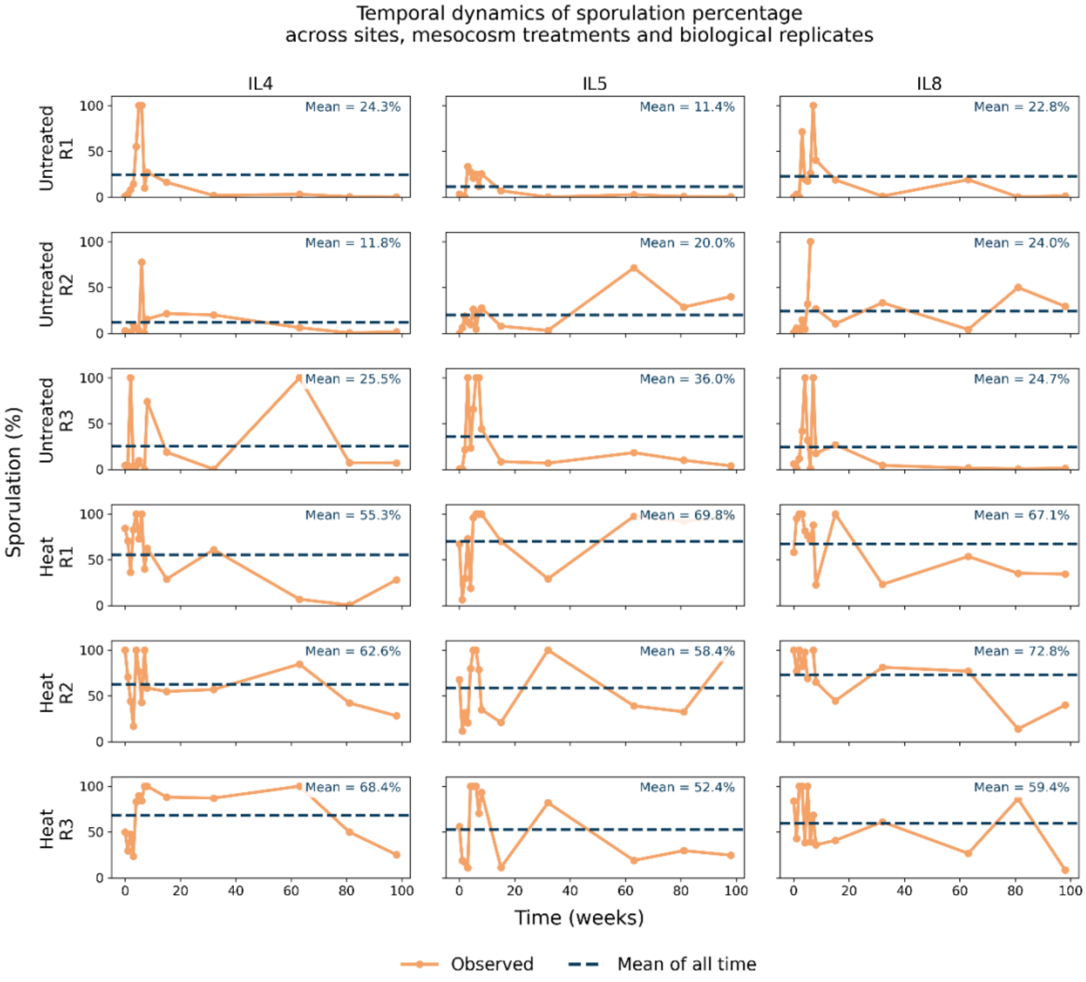

Supplementary Fig. 8. Sporulation dynamics across mesocosms. Line plots show temporal variation in sporulation percentage (%) over time (weeks) for individual mesocosms. Each panel corresponds to a single mesocosm. Orange lines indicate observed values, and dashed gray lines show the mean sporulation level across all time points, with the mean value displayed at the top of each panel. Columns represent samples from sites IL4, IL5, and IL8 (see Fig. 5), each with three independent replicates (rows). The first three show mesocosms initiated without prior heat treatment; the last three shows mesocosms initiated with heat treatment.

Supplementary Figure 9

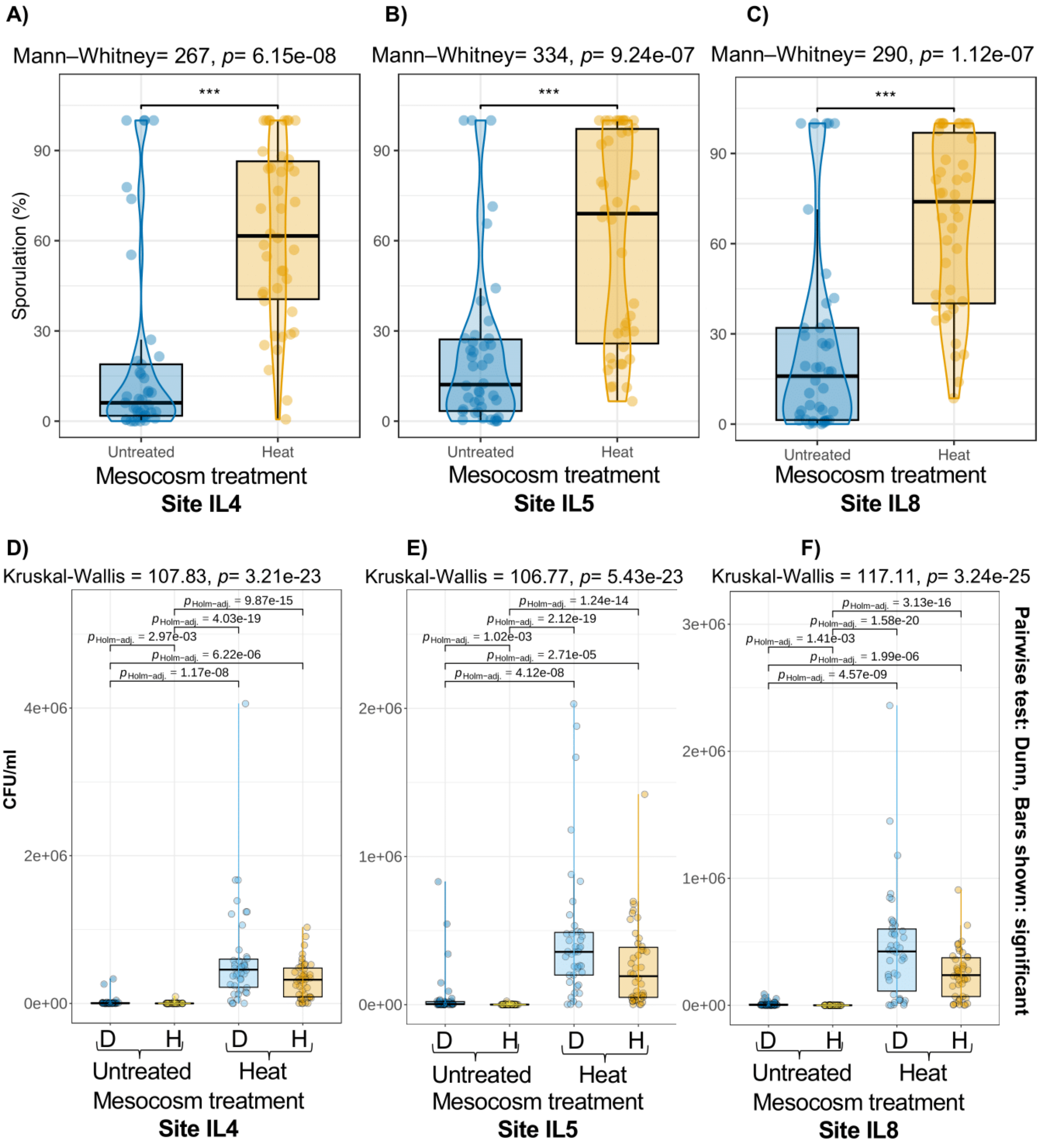

Supplementary Fig. 9 Statistical tests between mesocosm treatments in sporulation and absolute abundance. Panels A–C show sporulation percentages for untreated and heat-treated mesocosms at sites IL4, IL5, and IL8, respectively (Mann–Whitney tests). Panels D–F show CFU/mL for direct (D) and heat-treated (H) fractions at the same sites

(Kruskal–Wallis tests followed by Dunn's post hoc tests with Holm adjustment). Points represent individual observations and boxplots show median and interquartile ranges.
